# Supplementary material for: Identify Candidate Genes Associated with the Weight and Egg Quality Traits in Wenshui Green Shell-Laying Chickens by the Copy Number Variation-Based Genome-Wide Association Study
Source: Vet Sci. 2024 Feb 6;11(2):76. doi: 10.3390/vetsci11020076 (PMC10892766; doi:10.3390/vetsci11020076)
Supplement: Supplementary file 1 [file vetsci-11-00076-s001.zip › Supplementary Table S1.pdf]

Table S1. Number of phenotypes, mean, minimum, maximum, standard deviation and standard error of traits.

trait	Number of individuals examined	maximum	minimum	averages	standard deviation	standard error
30-EW(g)	789	58.90	34.87	46.15	3.25	0.1158
30-ESI	788	1.47	1.17	1.30	0.04	0.0016
40-EW(g)	772	67.92	37.41	49.36	3.84	0.1381
40-ESI	772	1.54	1.11	1.31	0.06	0.0021
40-YC	762	12.60	5.90	8.71	0.67	0.0241
40-EWH(mm)	763	7.00	2.20	3.99	0.85	0.0307
40-SH(mm)	771	0.39	0.22	0.31	0.03	0.0962
40-SS(N/cm2)	769	51.41	13.06	37.53	7.76	0.2799
40-YW(g)	759	20.28	9.86	16.02	1.28	0.0465
40-SW(g)	771	7.02	3.32	5.21	0.56	0.0201
40-EWW(g)	759	44.37	19.97	28.13	2.99	0.1085
40-YR	759	0.40	0.24	0.33	0.02	0.0008
40-SR	771	0.14	0.05	0.11	0.01	0.0004
40-EWR	759	0.66	0.48	0.57	0.03	0.0009
40-EWL	718	137.65	60.02	92.45	10.12	0.3778
40-EWS	718	103.93	51.63	71.86	7.56	0.2822
40-HU	763	86.75	40.03	63.55	8.19	0.2965
BW(g)	809	41.72	25.00	33.34	2.68	0.0941
4-W(g)	813	408.00	174.00	272.10	28.39	0.9957
8-W(g)	785	815.00	320.00	536.38	62.85	2.2433
13-W(g)	808	1,300.00	550.00	932.30	99.24	3.4911
15-W(g)	745	1,500.00	650.00	1,188.52	117.09	4.2899
38-W(g)	832	2,668.00	1,100.00	1,707.98	237.77	8.2433
