# Supplementary material for: Identify Candidate Genes Associated with the Weight and Egg Quality Traits in Wenshui Green Shell-Laying Chickens by the Copy Number Variation-Based Genome-Wide Association Study
Source: Vet Sci. 2024 Feb 6;11(2):76. doi: 10.3390/vetsci11020076 (PMC10892766; doi:10.3390/vetsci11020076)
Supplement: Supplementary file 1 [file vetsci-11-00076-s001.zip › Supplementary Table S2.pdf]

Table S2. Detail information of detected CNVR.

CNVR ID	Chr	Start	End	Size	Description
DEL00000000	chr1	892	1,543	651	Loss
DUP00000138	chr1	126,916	139,367	12,451	Gain
DEL00000207	chr1	243,133	243,439	306	Loss
DUP00000233	chr1	249,688	250,011	323	Gain
DUP00000311	chr1	290,728	354,892	64,164	Mixed
DEL00000504	chr1	455,861	456,033	172	Loss
DEL00000512	chr1	492,481	492,703	222	Loss
DUP00000519	chr1	519,681	519,877	196	Gain
DEL00000525	chr1	528,335	528,442	107	Loss
DEL00000532	chr1	569,535	569,639	104	Loss
DUP00000540	chr1	589,727	590,334	607	Gain
DEL00000563	chr1	740,685	740,895	210	Loss
DUP00000566	chr1	745,074	745,375	301	Gain
DEL00000597	chr1	761,327	761,548	221	Mixed
DUP00000617	chr1	790,558	790,688	130	Gain
DEL00000631	chr1	803,372	803,426	54	Loss
DUP00000633	chr1	809,822	811,090	1,268	Mixed
DEL00000808	chr1	955,646	956,636	990	Loss
DUP00000834	chr1	965,060	965,828	768	Mixed
DUP00000853	chr1	994,376	995,043	667	Gain
DEL00000881	chr1	1,114,496	1,115,153	657	Loss
DEL00000888	chr1	1,135,548	1,136,085	537	Loss
DUP00000890	chr1	1,139,442	1,139,656	214	Gain
DEL00000892	chr1	1,160,833	1,161,182	349	Loss
DUP00000896	chr1	1,236,958	1,237,678	720	Gain
DEL00000904	chr1	1,341,898	1,341,975	77	Loss
DEL00000913	chr1	1,721,750	1,722,606	856	Loss
DEL00000914	chr1	1,756,025	1,756,144	119	Loss
DEL00000916	chr1	1,831,402	1,831,478	76	Loss
DEL00000918	chr1	1,892,717	1,894,912	2,195	Loss
DEL00000938	chr1	2,177,924	2,178,320	396	Loss
DEL00000939	chr1	2,183,040	2,183,253	213	Loss
DEL00000942	chr1	2,263,736	2,264,543	807	Loss
DEL00000945	chr1	2,296,073	2,296,338	265	Loss
DEL00000947	chr1	2,481,957	2,482,777	820	Loss
DEL00000956	chr1	2,654,111	2,654,295	184	Loss
DEL00000959	chr1	2,745,958	2,746,459	501	Loss
DEL00000960	chr1	2,759,061	2,759,157	96	Loss
DEL00000963	chr1	2,861,047	2,861,120	73	Loss
DEL00000966	chr1	3,028,284	3,028,931	647	Loss
DEL00000967	chr1	3,030,621	3,030,785	164	Loss
DEL00000971	chr1	3,096,719	3,096,930	211	Loss
DEL00000973	chr1	3,130,190	3,130,287	97	Loss
DEL00000980	chr1	3,355,087	3,439,927	84,840	Loss
DEL00000996	chr1	3,532,688	3,533,318	630	Loss
DEL00000998	chr1	3,658,564	3,658,978	414	Loss

DEL00000999	chr1	3,666,405	3,666,706	301	Loss
DUP00001001	chr1	3,722,289	3,722,663	374	Gain
DUP00001005	chr1	3,763,086	3,763,192	106	Gain
DEL00001010	chr1	3,933,544	3,933,917	373	Loss
DEL00001013	chr1	3,946,613	3,947,173	560	Loss
DEL00001015	chr1	4,013,201	4,013,458	257	Loss
DEL00001016	chr1	4,059,093	4,059,860	767	Loss
DEL00001018	chr1	4,147,048	4,147,141	93	Loss
DEL00001019	chr1	4,178,857	4,178,987	130	Loss
DEL00001022	chr1	4,241,231	4,241,321	90	Loss
DEL00001025	chr1	4,365,026	4,365,550	524	Loss
DEL00001026	chr1	4,365,785	4,365,850	65	Loss
DUP00001029	chr1	4,451,956	4,452,081	125	Gain
DEL00001030	chr1	4,464,765	4,465,870	1,105	Loss
DEL00001031	chr1	4,490,562	4,490,613	51	Loss
DEL00001034	chr1	4,612,112	4,612,286	174	Loss
DEL00001049	chr1	4,794,550	4,794,666	116	Loss
DEL00001052	chr1	4,860,096	4,860,214	118	Loss
DEL00001053	chr1	4,860,277	4,860,392	115	Loss
DEL00001054	chr1	4,880,381	4,880,564	183	Loss
DEL00001055	chr1	4,891,739	4,892,225	486	Loss
DEL00001058	chr1	5,005,775	5,005,904	129	Loss
DEL00001060	chr1	5,057,109	5,057,231	122	Loss
DEL00001062	chr1	5,119,646	5,120,745	1,099	Loss
DEL00001065	chr1	5,166,443	5,166,539	96	Loss
DEL00001072	chr1	5,447,856	5,447,914	58	Loss
DEL00001075	chr1	5,483,254	5,483,375	121	Loss
DEL00001080	chr1	5,696,402	5,696,997	595	Loss
DEL00001084	chr1	5,795,461	5,795,925	464	Loss
DEL00001088	chr1	5,844,874	5,846,070	1,196	Loss
DEL00001091	chr1	5,859,216	5,859,493	277	Loss
DEL00001092	chr1	5,868,711	5,868,762	51	Loss
DEL00001095	chr1	5,933,799	5,934,237	438	Loss
DEL00001096	chr1	5,980,819	5,980,968	149	Loss
DUP00001108	chr1	6,413,076	6,413,203	127	Gain
DEL00001117	chr1	6,819,299	6,828,675	9,376	Loss
DEL00001118	chr1	6,853,475	6,854,055	580	Loss
DEL00001119	chr1	6,864,345	6,864,963	618	Loss
DEL00001120	chr1	7,036,895	7,037,705	810	Loss
DEL00001121	chr1	7,044,477	7,044,602	125	Loss
DEL00001123	chr1	7,162,152	7,162,605	453	Loss
DEL00001150	chr1	7,327,796	7,328,032	236	Loss
DEL00001151	chr1	7,348,888	7,349,414	526	Loss
DEL00001156	chr1	7,413,551	7,413,728	177	Loss
DEL00001160	chr1	7,489,562	7,489,613	51	Loss
DEL00001163	chr1	7,531,607	7,531,668	61	Loss
DUP00001165	chr1	7,627,826	7,627,907	81	Gain
DEL00001168	chr1	7,667,038	7,667,150	112	Loss
DEL00001172	chr1	7,754,413	7,754,795	382	Loss

DEL00001174	chr1	7,765,039	7,765,229	190	Loss
DEL00001178	chr1	7,875,784	7,875,986	202	Loss
DEL00001185	chr1	7,939,805	7,939,868	63	Loss
DEL00001187	chr1	7,966,536	7,967,080	544	Loss
DEL00001190	chr1	8,011,293	8,011,779	486	Loss
DEL00001194	chr1	8,033,733	8,034,005	272	Loss
DEL00001205	chr1	8,226,627	8,227,043	416	Loss
DEL00001206	chr1	8,228,285	8,229,071	786	Loss
DEL00001208	chr1	8,253,868	8,253,968	100	Loss
DEL00001213	chr1	8,425,965	8,426,482	517	Loss
DEL00001214	chr1	8,428,165	8,435,271	7,106	Loss
DEL00001220	chr1	8,674,754	8,675,255	501	Loss
DEL00001221	chr1	8,683,128	8,683,343	215	Loss
DEL00001222	chr1	8,704,088	8,704,278	190	Loss
DEL00001225	chr1	8,754,965	8,755,029	64	Loss
DEL00001226	chr1	8,769,245	8,770,345	1,100	Loss
DEL00001227	chr1	8,843,011	8,843,192	181	Loss
DEL00001230	chr1	8,920,332	8,920,981	649	Loss
DEL00001232	chr1	8,989,690	8,989,749	59	Loss
DEL00001238	chr1	9,145,387	9,145,445	58	Loss
DEL00001243	chr1	9,272,270	9,272,343	73	Loss
DEL00001244	chr1	9,301,264	9,301,360	96	Loss
DEL00001247	chr1	9,453,577	9,453,664	87	Loss
DEL00001248	chr1	9,481,968	9,482,172	204	Loss
DEL00001253	chr1	9,602,136	9,602,309	173	Loss
DEL00001254	chr1	9,621,627	9,621,695	68	Loss
DEL00001258	chr1	9,644,872	9,645,624	752	Loss
DEL00001262	chr1	9,729,363	9,729,692	329	Loss
DEL00001263	chr1	9,732,588	9,732,752	164	Loss
DEL00001264	chr1	9,780,413	9,781,586	1,173	Loss
DEL00001266	chr1	9,787,887	9,788,389	502	Loss
DEL00001279	chr1	10,133,647	10,134,115	468	Loss
DEL00001280	chr1	10,138,092	10,138,312	220	Loss
DEL00001281	chr1	10,148,100	10,148,177	77	Loss
DEL00001283	chr1	10,193,722	10,194,223	501	Loss
DEL00001287	chr1	10,218,382	10,218,748	366	Loss
DEL00001288	chr1	10,289,405	10,291,596	2,191	Loss
DEL00001289	chr1	10,292,663	10,292,717	54	Loss
DEL00001290	chr1	10,292,968	10,293,547	579	Loss
DEL00001291	chr1	10,295,492	10,295,877	385	Loss
DEL00001292	chr1	10,298,544	10,302,377	3,833	Loss
DEL00001296	chr1	10,315,330	10,315,395	65	Loss
DEL00001298	chr1	10,492,181	10,492,303	122	Loss
DEL00001299	chr1	10,561,567	10,561,679	112	Loss
DEL00001301	chr1	10,624,588	10,624,649	61	Loss
DEL00001302	chr1	10,652,058	10,652,123	65	Loss
DEL00001307	chr1	10,736,167	10,736,447	280	Loss
DEL00001317	chr1	11,016,733	11,016,794	61	Loss
DEL00001323	chr1	11,054,967	11,055,030	63	Loss

DEL00001332	chr1	11,133,717	11,134,243	526	Loss
DEL00001336	chr1	11,264,460	11,264,545	85	Loss
DEL00001338	chr1	11,286,892	11,286,955	63	Loss
DEL00001341	chr1	11,333,247	11,334,203	956	Loss
DEL00001344	chr1	11,469,962	11,472,001	2,039	Loss
DEL00001351	chr1	11,521,252	11,521,687	435	Loss
DEL00001353	chr1	11,537,193	11,537,278	85	Loss
DEL00001355	chr1	11,549,895	11,550,424	529	Loss
DEL00001359	chr1	11,613,187	11,613,298	111	Loss
DEL00001364	chr1	11,733,132	11,733,198	66	Loss
DEL00001365	chr1	11,749,323	11,749,536	213	Loss
DEL00001366	chr1	11,751,850	11,751,931	81	Loss
DEL00001371	chr1	11,786,050	11,786,110	60	Loss
DEL00001374	chr1	11,791,771	11,791,987	216	Loss
DEL00001375	chr1	11,816,259	11,816,343	84	Loss
DEL00001381	chr1	11,897,091	11,897,365	274	Loss
DEL00001384	chr1	11,968,781	11,969,162	381	Loss
DEL00001388	chr1	12,057,971	12,058,039	68	Loss
DEL00001394	chr1	12,109,125	12,109,467	342	Loss
DEL00001402	chr1	12,230,178	12,230,961	783	Loss
DEL00001404	chr1	12,261,230	12,262,466	1,236	Loss
DEL00001405	chr1	12,286,142	12,286,763	621	Loss
DEL00001407	chr1	12,333,468	12,333,527	59	Loss
DEL00001409	chr1	12,334,828	12,335,088	260	Loss
DEL00001410	chr1	12,355,957	12,356,171	214	Loss
DEL00001414	chr1	12,507,207	12,507,763	556	Loss
DEL00001416	chr1	12,512,334	12,512,448	114	Loss
DEL00001418	chr1	12,530,582	12,531,166	584	Loss
DEL00001425	chr1	12,761,463	12,761,632	169	Loss
DEL00001427	chr1	12,783,436	12,783,547	111	Loss
DEL00001428	chr1	12,802,701	12,803,190	489	Loss
DEL00001430	chr1	12,814,702	12,814,786	84	Loss
DEL00001440	chr1	13,016,822	13,017,008	186	Loss
DEL00001442	chr1	13,095,546	13,095,650	104	Loss
DEL00001445	chr1	13,127,448	13,127,521	73	Loss
DEL00001454	chr1	13,189,491	13,189,641	150	Loss
DEL00001485	chr1	13,633,358	13,633,919	561	Loss
DEL00001488	chr1	13,706,975	13,707,099	124	Loss
DUP00001493	chr1	13,830,570	13,830,689	119	Gain
DEL00001500	chr1	13,889,936	13,890,132	196	Loss
DEL00001501	chr1	13,985,399	13,985,883	484	Loss
DEL00001504	chr1	14,049,995	14,050,053	58	Loss
DUP00001509	chr1	14,206,827	14,207,078	251	Gain
DEL00001510	chr1	14,224,516	14,224,571	55	Loss
DEL00001518	chr1	14,437,726	14,437,988	262	Loss
DEL00001519	chr1	14,455,043	14,455,146	103	Loss
DEL00001526	chr1	14,601,449	14,601,571	122	Loss
DEL00001531	chr1	14,649,784	14,650,509	725	Loss
DEL00001534	chr1	14,766,905	14,767,834	929	Loss

DEL00001536	chr1	14,823,834	14,824,395	561	Loss
DEL00001541	chr1	14,942,716	14,942,850	134	Loss
DUP00001543	chr1	14,997,614	15,013,985	16,371	Gain
DEL00001547	chr1	15,109,658	15,109,844	186	Loss
DEL00001551	chr1	15,169,412	15,169,613	201	Loss
DEL00001553	chr1	15,174,117	15,174,487	370	Loss
DEL00001557	chr1	15,242,282	15,242,651	369	Loss
DEL00001558	chr1	15,255,521	15,255,682	161	Loss
DEL00001559	chr1	15,301,794	15,301,848	54	Loss
DEL00001565	chr1	15,354,263	15,354,400	137	Loss
DEL00001567	chr1	15,434,560	15,434,658	98	Loss
DEL00001570	chr1	15,490,476	15,491,210	734	Loss
DEL00001576	chr1	15,597,456	15,598,246	790	Loss
DEL00001577	chr1	15,609,387	15,609,443	56	Loss
DEL00001579	chr1	15,627,450	15,627,557	107	Loss
DEL00001582	chr1	15,743,221	15,747,238	4,017	Loss
DEL00001589	chr1	15,812,607	15,812,677	70	Loss
DEL00001597	chr1	15,970,728	15,970,816	88	Loss
DEL00001606	chr1	16,164,352	16,164,558	206	Loss
DEL00001607	chr1	16,178,434	16,178,949	515	Loss
DEL00001611	chr1	16,238,742	16,238,890	148	Loss
DEL00001615	chr1	16,336,102	16,336,823	721	Loss
DEL00001618	chr1	16,439,427	16,439,561	134	Loss
DUP00001623	chr1	16,636,333	16,636,419	86	Gain
DEL00001625	chr1	16,663,823	16,664,422	599	Loss
DEL00001628	chr1	16,703,608	16,703,931	323	Loss
DEL00001629	chr1	16,704,550	16,704,614	64	Loss
DEL00001630	chr1	16,827,962	16,829,440	1,478	Loss
DEL00001631	chr1	16,830,454	16,841,272	10,818	Loss
DEL00001637	chr1	16,865,198	16,867,033	1,835	Loss
DEL00001640	chr1	16,990,985	16,991,038	53	Loss
DEL00001645	chr1	17,087,603	17,087,798	195	Loss
DEL00001646	chr1	17,151,780	17,152,004	224	Loss
DEL00001647	chr1	17,159,017	17,163,317	4,300	Loss
DEL00001648	chr1	17,186,618	17,187,564	946	Loss
DEL00001650	chr1	17,203,070	17,205,276	2,206	Loss
DEL00001651	chr1	17,223,617	17,223,988	371	Loss
DEL00001654	chr1	17,335,051	17,335,161	110	Loss
DEL00001658	chr1	17,391,102	17,391,901	799	Loss
DEL00001659	chr1	17,431,038	17,431,420	382	Loss
DEL00001660	chr1	17,436,532	17,436,630	98	Loss
DEL00001662	chr1	17,460,897	17,461,132	235	Loss
DEL00001663	chr1	17,482,558	17,482,957	399	Loss
DEL00001666	chr1	17,565,703	17,566,593	890	Loss
DEL00001669	chr1	17,580,838	17,581,168	330	Loss
DEL00001670	chr1	17,589,402	17,589,543	141	Loss
DUP00001673	chr1	17,701,462	17,717,784	16,322	Gain
DEL00001675	chr1	17,741,242	17,741,324	82	Loss
DEL00001680	chr1	17,869,246	17,870,914	1,668	Loss

DEL00001682	chr1	17,878,661	17,879,323	662	Loss
DEL00001721	chr1	17,995,477	17,995,927	450	Loss
DEL00001723	chr1	18,008,340	18,008,578	238	Loss
DEL00001724	chr1	18,042,916	18,043,284	368	Loss
DEL00001730	chr1	18,165,623	18,171,696	6,073	Loss
DEL00001740	chr1	18,377,379	18,378,284	905	Loss
DEL00001746	chr1	18,421,182	18,422,892	1,710	Loss
DEL00001747	chr1	18,457,419	18,458,082	663	Loss
DEL00001752	chr1	18,515,338	18,516,258	920	Loss
DEL00001753	chr1	18,525,904	18,526,143	239	Loss
DEL00001757	chr1	18,548,603	18,548,851	248	Loss
DEL00001758	chr1	18,597,409	18,599,108	1,699	Loss
DEL00001759	chr1	18,697,947	18,698,013	66	Loss
DEL00001760	chr1	18,721,954	18,722,384	430	Loss
DEL00001761	chr1	18,725,998	18,726,845	847	Loss
DEL00001763	chr1	18,739,666	18,740,842	1,176	Loss
DEL00001767	chr1	18,769,249	18,769,385	136	Loss
DEL00001769	chr1	18,790,862	18,791,326	464	Loss
DEL00001770	chr1	18,799,120	18,799,535	415	Loss
DUP00001783	chr1	19,025,298	19,025,442	144	Gain
DEL00001786	chr1	19,147,452	19,147,568	116	Loss
DEL00001787	chr1	19,209,811	19,210,056	245	Loss
DEL00001788	chr1	19,217,686	19,218,314	628	Loss
DEL00001790	chr1	19,241,928	19,241,994	66	Loss
DEL00001796	chr1	19,392,097	19,392,884	787	Loss
DEL00001797	chr1	19,417,083	19,417,554	471	Loss
DEL00001799	chr1	19,499,251	19,499,417	166	Loss
DEL00001802	chr1	19,514,647	19,515,151	504	Loss
DUP00001809	chr1	19,829,190	19,829,385	195	Gain
DEL00001815	chr1	19,872,382	19,873,465	1,083	Loss
DEL00001816	chr1	19,890,878	19,897,421	6,543	Loss
DEL00001821	chr1	20,038,138	20,039,014	876	Loss
DEL00001823	chr1	20,083,605	20,083,959	354	Loss
DEL00001825	chr1	20,099,445	20,099,533	88	Loss
DEL00001827	chr1	20,142,886	20,142,937	51	Loss
DEL00001834	chr1	20,386,961	20,387,729	768	Loss
DEL00001835	chr1	20,397,882	20,399,032	1,150	Loss
DEL00001841	chr1	20,454,154	20,454,483	329	Loss
DEL00001842	chr1	20,517,301	20,517,445	144	Loss
DEL00001849	chr1	20,543,292	20,545,995	2,703	Loss
DEL00001852	chr1	20,668,198	20,668,337	139	Loss
DEL00001853	chr1	20,747,008	20,747,777	769	Loss
DUP00001855	chr1	20,904,502	20,904,592	90	Gain
DEL00001857	chr1	20,929,294	20,929,411	117	Loss
DEL00001860	chr1	21,017,288	21,017,352	64	Loss
DEL00001862	chr1	21,027,912	21,028,144	232	Loss
DEL00001866	chr1	21,155,428	21,155,485	57	Loss
DEL00001868	chr1	21,184,937	21,185,122	185	Loss
DUP00001870	chr1	21,262,844	21,283,488	20,644	Gain

DUP00001872	chr1	21,297,993	21,298,100	107	Gain
DEL00001877	chr1	21,488,966	21,489,607	641	Loss
DEL00001878	chr1	21,511,867	21,512,070	203	Loss
DEL00001880	chr1	21,514,895	21,519,223	4,328	Loss
DEL00001881	chr1	21,532,199	21,532,290	91	Loss
DEL00001885	chr1	21,559,689	21,559,916	227	Loss
DEL00001886	chr1	21,564,780	21,564,903	123	Loss
DEL00001887	chr1	21,581,145	21,581,891	746	Loss
DEL00001888	chr1	21,582,216	21,583,074	858	Loss
DEL00001889	chr1	21,590,176	21,591,304	1,128	Loss
DEL00001895	chr1	21,646,401	21,646,475	74	Loss
DEL00001902	chr1	21,689,995	21,690,211	216	Loss
DEL00001909	chr1	21,761,803	21,762,774	971	Loss
DEL00001911	chr1	21,779,395	21,780,023	628	Loss
DEL00001919	chr1	21,870,439	21,870,531	92	Loss
DEL00001920	chr1	21,880,449	21,880,552	103	Loss
DEL00001925	chr1	21,984,173	21,984,251	78	Loss
DEL00001928	chr1	22,002,497	22,002,591	94	Loss
DEL00001929	chr1	22,005,959	22,006,019	60	Loss
DEL00001931	chr1	22,101,848	22,102,314	466	Loss
DEL00001935	chr1	22,126,918	22,127,640	722	Loss
DEL00001936	chr1	22,192,636	22,193,027	391	Loss
DEL00001937	chr1	22,210,927	22,211,008	81	Loss
DEL00001940	chr1	22,328,280	22,328,508	228	Loss
DEL00001941	chr1	22,358,463	22,358,674	211	Loss
DUP00001942	chr1	22,383,986	22,384,188	202	Gain
DEL00001944	chr1	22,424,072	22,424,449	377	Loss
DEL00001946	chr1	22,425,621	22,426,253	632	Loss
DUP00001948	chr1	22,459,412	22,459,561	149	Gain
DEL00001950	chr1	22,486,770	22,487,647	877	Loss
DEL00001952	chr1	22,510,356	22,511,127	771	Loss
DEL00001953	chr1	22,515,478	22,516,079	601	Loss
DEL00001955	chr1	22,582,521	22,582,592	71	Loss
DEL00001958	chr1	22,618,357	22,618,456	99	Loss
DEL00001959	chr1	22,645,430	22,645,527	97	Loss
DEL00001961	chr1	22,699,896	22,700,079	183	Loss
DEL00001962	chr1	22,732,350	22,732,526	176	Loss
DEL00001965	chr1	22,803,823	22,804,008	185	Loss
DEL00001967	chr1	22,851,739	22,852,627	888	Loss
DEL00001969	chr1	22,855,410	22,855,464	54	Loss
DEL00001971	chr1	22,862,446	22,862,617	171	Loss
DEL00001972	chr1	22,870,984	22,871,954	970	Loss
DEL00001976	chr1	22,924,634	22,925,094	460	Loss
DEL00001977	chr1	22,973,055	22,973,107	52	Loss
DEL00001978	chr1	22,982,371	22,982,925	554	Loss
DEL00001980	chr1	23,030,762	23,031,168	406	Loss
DEL00001983	chr1	23,103,874	23,104,232	358	Loss
DEL00001986	chr1	23,211,251	23,212,507	1,256	Loss
DEL00001987	chr1	23,225,674	23,225,769	95	Loss

DEL00001991	chr1	23,246,809	23,248,350	1,541	Loss
DEL00001992	chr1	23,261,031	23,261,573	542	Loss
DEL00001996	chr1	23,330,300	23,331,241	941	Loss
DEL00001997	chr1	23,337,360	23,337,420	60	Loss
DEL00001998	chr1	23,354,700	23,356,376	1,676	Loss
DEL00001999	chr1	23,360,473	23,360,684	211	Loss
DEL00002003	chr1	23,398,018	23,398,284	266	Loss
DEL00002004	chr1	23,422,902	23,423,487	585	Loss
DEL00002006	chr1	23,425,385	23,425,458	73	Loss
DEL00002011	chr1	23,460,595	23,460,665	70	Loss
DEL00002014	chr1	23,541,671	23,543,251	1,580	Loss
DEL00002016	chr1	23,561,544	23,561,639	95	Loss
DEL00002019	chr1	23,641,700	23,641,815	115	Loss
DEL00002020	chr1	23,647,674	23,647,728	54	Loss
DEL00002021	chr1	23,658,758	23,658,891	133	Loss
DEL00002022	chr1	23,664,180	23,664,305	125	Loss
DEL00002024	chr1	23,712,732	23,713,186	454	Loss
DEL00002026	chr1	23,849,400	23,849,490	90	Loss
DEL00002028	chr1	23,866,928	23,867,109	181	Loss
DEL00002029	chr1	23,870,083	23,870,239	156	Loss
DEL00002031	chr1	23,941,453	23,942,678	1,225	Loss
DEL00002032	chr1	23,969,899	23,971,121	1,222	Loss
DEL00002033	chr1	23,976,155	23,978,336	2,181	Loss
DEL00002034	chr1	23,982,571	23,982,658	87	Loss
DUP00002035	chr1	23,986,402	24,016,655	30,253	Gain
DEL00002036	chr1	24,048,025	24,048,440	415	Loss
DEL00002038	chr1	24,151,715	24,152,773	1,058	Loss
DEL00002042	chr1	24,163,324	24,163,398	74	Loss
DUP00002045	chr1	24,190,562	24,190,697	135	Gain
DEL00002046	chr1	24,192,573	24,192,644	71	Loss
DEL00002049	chr1	24,221,743	24,223,037	1,294	Loss
DEL00002050	chr1	24,223,966	24,224,040	74	Loss
DEL00002053	chr1	24,269,011	24,269,182	171	Loss
DEL00002061	chr1	24,567,371	24,567,573	202	Loss
DEL00002067	chr1	24,702,746	24,702,958	212	Loss
DEL00002069	chr1	24,742,021	24,742,140	119	Loss
DEL00002070	chr1	24,751,974	24,752,794	820	Loss
DEL00002082	chr1	25,035,604	25,035,723	119	Loss
DEL00002083	chr1	25,043,733	25,043,807	74	Loss
DEL00002084	chr1	25,061,505	25,061,566	61	Loss
DEL00002087	chr1	25,130,241	25,130,354	113	Loss
DEL00002090	chr1	25,165,738	25,165,883	145	Loss
DEL00002091	chr1	25,192,973	25,193,025	52	Loss
DEL00002093	chr1	25,306,231	25,306,871	640	Loss
DEL00002094	chr1	25,314,057	25,314,108	51	Loss
DEL00002100	chr1	25,493,123	25,493,243	120	Loss
DEL00002105	chr1	25,867,995	25,868,497	502	Loss
DEL00002106	chr1	25,957,888	25,958,131	243	Loss
DEL00002110	chr1	26,042,017	26,042,153	136	Loss

DEL00002112	chr1	26,048,883	26,048,956	73	Loss
DEL00002113	chr1	26,059,535	26,059,692	157	Loss
DEL00002114	chr1	26,112,874	26,114,327	1,453	Loss
DEL00002117	chr1	26,186,939	26,187,004	65	Loss
DEL00002119	chr1	26,253,262	26,253,472	210	Loss
DEL00002121	chr1	26,260,388	26,260,554	166	Loss
DEL00002123	chr1	26,321,513	26,322,099	586	Loss
DEL00002126	chr1	26,429,127	26,429,295	168	Loss
DEL00002132	chr1	26,513,902	26,514,374	472	Loss
DEL00002134	chr1	26,660,527	26,660,936	409	Loss
DEL00002136	chr1	26,708,798	26,709,283	485	Loss
DEL00002142	chr1	26,850,219	26,850,324	105	Loss
DEL00002143	chr1	26,861,989	26,862,771	782	Loss
DEL00002145	chr1	26,941,261	26,941,357	96	Loss
DEL00002146	chr1	26,945,865	26,945,949	84	Loss
DEL00002148	chr1	27,026,043	27,026,140	97	Loss
DEL00002151	chr1	27,072,638	27,072,813	175	Loss
DEL00002157	chr1	27,087,052	27,087,266	214	Loss
DEL00002161	chr1	27,176,676	27,176,839	163	Loss
DEL00002173	chr1	27,470,697	27,471,068	371	Loss
DEL00002185	chr1	27,727,935	27,728,005	70	Loss
DEL00002186	chr1	27,734,161	27,734,236	75	Loss
DEL00002191	chr1	27,818,009	27,818,169	160	Loss
DEL00002195	chr1	27,937,309	27,937,528	219	Loss
DEL00002197	chr1	27,969,052	27,969,206	154	Loss
DEL00002198	chr1	27,983,063	27,983,187	124	Loss
DEL00002201	chr1	28,040,845	28,043,386	2,541	Loss
DEL00002203	chr1	28,044,664	28,045,402	738	Loss
DEL00002205	chr1	28,071,664	28,074,204	2,540	Loss
DEL00002209	chr1	28,171,497	28,171,585	88	Loss
DEL00002212	chr1	28,226,262	28,226,554	292	Loss
DEL00002215	chr1	28,281,544	28,281,622	78	Loss
DEL00002216	chr1	28,287,651	28,288,281	630	Loss
DEL00002218	chr1	28,313,072	28,313,986	914	Loss
DEL00002219	chr1	28,336,380	28,337,279	899	Loss
DEL00002220	chr1	28,340,604	28,340,716	112	Loss
DEL00002221	chr1	28,434,904	28,434,964	60	Loss
DEL00002226	chr1	28,520,654	28,520,772	118	Loss
DEL00002229	chr1	28,676,830	28,677,218	388	Loss
DEL00002231	chr1	28,925,454	28,925,534	80	Loss
DEL00002237	chr1	29,152,960	29,153,035	75	Loss
DEL00002238	chr1	29,161,833	29,162,248	415	Loss
DEL00002239	chr1	29,178,737	29,178,809	72	Loss
DUP00002245	chr1	29,292,102	29,292,398	296	Gain
DEL00002246	chr1	29,313,454	29,314,008	554	Loss
DEL00002250	chr1	29,452,054	29,452,138	84	Loss
DEL00002251	chr1	29,476,335	29,476,397	62	Loss
DEL00002253	chr1	29,543,374	29,544,127	753	Loss
DEL00002254	chr1	29,551,678	29,552,605	927	Loss

DEL00002255	chr1	29,579,022	29,579,081	59	Loss
DEL00002256	chr1	29,678,175	29,678,646	471	Loss
DEL00002257	chr1	29,692,288	29,692,783	495	Loss
DEL00002267	chr1	30,044,329	30,044,913	584	Loss
DEL00002272	chr1	30,117,106	30,117,803	697	Loss
DEL00002273	chr1	30,163,523	30,163,747	224	Loss
DEL00002274	chr1	30,177,094	30,177,153	59	Loss
DEL00002276	chr1	30,212,737	30,212,866	129	Loss
DEL00002277	chr1	30,271,423	30,271,603	180	Loss
DEL00002279	chr1	30,371,047	30,371,104	57	Loss
DEL00002281	chr1	30,433,811	30,433,978	167	Loss
DUP00002286	chr1	30,564,442	30,564,524	82	Gain
DEL00002290	chr1	30,665,835	30,665,952	117	Loss
DUP00002292	chr1	30,677,983	30,690,100	12,117	Gain
DEL00002295	chr1	30,730,332	30,730,400	68	Loss
DUP00002298	chr1	30,812,168	30,812,252	84	Gain
DEL00002300	chr1	30,850,449	30,852,001	1,552	Loss
DEL00002304	chr1	30,887,678	30,888,117	439	Loss
DEL00002307	chr1	30,917,941	30,918,002	61	Loss
DEL00002318	chr1	31,350,761	31,350,830	69	Loss
DEL00002320	chr1	31,441,583	31,441,764	181	Loss
DEL00002323	chr1	31,504,318	31,506,401	2,083	Loss
DEL00002324	chr1	31,513,947	31,514,040	93	Loss
DEL00002327	chr1	31,608,246	31,608,388	142	Loss
DEL00002328	chr1	31,609,844	31,609,964	120	Loss
DEL00002329	chr1	31,619,613	31,619,682	69	Loss
DEL00002330	chr1	31,623,293	31,623,647	354	Loss
DEL00002334	chr1	31,688,077	31,688,220	143	Loss
DEL00002335	chr1	31,690,737	31,691,384	647	Loss
DEL00002336	chr1	31,731,587	31,731,641	54	Loss
DEL00002338	chr1	31,783,369	31,785,780	2,411	Loss
DEL00002339	chr1	31,814,497	31,818,809	4,312	Loss
DEL00002342	chr1	31,870,415	31,870,545	130	Loss
DEL00002343	chr1	31,875,034	31,875,143	109	Loss
DEL00002344	chr1	31,894,806	31,895,544	738	Loss
DEL00002345	chr1	31,973,755	31,973,919	164	Loss
DUP00002350	chr1	32,116,695	32,135,841	19,146	Mixed
DEL00002354	chr1	32,149,760	32,155,586	5,826	Loss
DEL00002355	chr1	32,161,191	32,161,406	215	Loss
DEL00002356	chr1	32,165,218	32,165,320	102	Loss
DEL00002357	chr1	32,168,380	32,168,981	601	Loss
DEL00002361	chr1	32,262,019	32,262,874	855	Loss
DEL00002362	chr1	32,283,293	32,283,736	443	Loss
DEL00002363	chr1	32,307,530	32,307,679	149	Loss
DEL00002365	chr1	32,401,819	32,402,600	781	Loss
DEL00002366	chr1	32,407,416	32,407,741	325	Loss
DEL00002369	chr1	32,456,145	32,456,711	566	Loss
DEL00002375	chr1	32,498,029	32,499,177	1,148	Loss
DEL00002383	chr1	32,615,758	32,615,831	73	Loss

DEL00002384	chr1	32,623,794	32,633,989	10,195	Loss
DEL00002389	chr1	32,663,265	32,664,563	1,298	Loss
DEL00002399	chr1	32,762,676	32,763,094	418	Loss
DEL00002400	chr1	32,814,750	32,820,584	5,834	Loss
DEL00002407	chr1	33,016,972	33,017,026	54	Loss
DEL00002408	chr1	33,018,847	33,018,914	67	Loss
DEL00002412	chr1	33,157,946	33,158,186	240	Loss
DEL00002413	chr1	33,164,709	33,164,893	184	Loss
DEL00002422	chr1	33,395,187	33,395,264	77	Loss
DEL00002430	chr1	33,600,972	33,601,111	139	Loss
DEL00002435	chr1	33,718,562	33,718,672	110	Loss
DEL00002436	chr1	33,721,440	33,722,103	663	Loss
DEL00002439	chr1	33,881,530	33,881,672	142	Loss
DEL00002443	chr1	33,980,749	33,981,135	386	Loss
DEL00002455	chr1	34,184,391	34,184,503	112	Loss
DEL00002456	chr1	34,283,467	34,284,261	794	Loss
DEL00002457	chr1	34,321,886	34,322,052	166	Loss
DEL00002460	chr1	34,354,992	34,355,124	132	Loss
DEL00002468	chr1	34,606,521	34,606,831	310	Loss
DEL00002469	chr1	34,619,558	34,619,661	103	Loss
DEL00002471	chr1	34,629,194	34,629,396	202	Loss
DEL00002472	chr1	34,644,434	34,644,501	67	Loss
DEL00002474	chr1	34,779,303	34,779,414	111	Loss
DEL00002476	chr1	34,874,391	34,874,478	87	Loss
DEL00002478	chr1	34,907,514	34,907,580	66	Loss
DEL00002482	chr1	34,933,451	34,935,332	1,881	Loss
DEL00002484	chr1	35,093,340	35,093,700	360	Loss
DEL00002487	chr1	35,134,647	35,140,832	6,185	Loss
DEL00002489	chr1	35,193,861	35,194,057	196	Loss
DUP00002492	chr1	35,309,593	35,309,845	252	Gain
DEL00002494	chr1	35,376,287	35,376,519	232	Loss
DEL00002497	chr1	35,435,618	35,435,685	67	Loss
DEL00002503	chr1	35,580,666	35,581,193	527	Loss
DEL00002505	chr1	35,588,209	35,589,000	791	Loss
DEL00002506	chr1	35,595,768	35,595,854	86	Loss
DEL00002512	chr1	35,770,778	35,771,342	564	Loss
DEL00002516	chr1	35,843,033	35,843,237	204	Loss
DEL00002517	chr1	35,854,222	35,854,407	185	Loss
DEL00002520	chr1	35,962,029	35,962,549	520	Loss
DEL00002529	chr1	36,082,570	36,082,675	105	Loss
DEL00002537	chr1	36,358,440	36,358,941	501	Loss
DEL00002540	chr1	36,394,757	36,394,867	110	Loss
DEL00002545	chr1	36,577,858	36,579,053	1,195	Loss
DEL00002546	chr1	36,584,355	36,584,461	106	Loss
DEL00002547	chr1	36,593,307	36,593,390	83	Loss
DEL00002551	chr1	36,630,719	36,630,771	52	Loss
DEL00002552	chr1	36,702,819	36,702,998	179	Loss
DEL00002557	chr1	36,741,489	36,741,979	490	Loss
DEL00002564	chr1	36,899,140	36,899,384	244	Loss

DEL00002565	chr1	36,906,325	36,906,788	463	Loss
DEL00002568	chr1	37,008,915	37,008,987	72	Loss
DEL00002569	chr1	37,118,670	37,118,942	272	Loss
DEL00002570	chr1	37,170,713	37,171,552	839	Loss
DEL00002572	chr1	37,249,637	37,250,919	1,282	Loss
DEL00002574	chr1	37,301,244	37,302,589	1,345	Loss
DEL00002577	chr1	37,404,006	37,404,060	54	Loss
DEL00002578	chr1	37,446,792	37,446,867	75	Loss
DEL00002579	chr1	37,486,656	37,487,596	940	Loss
DEL00002584	chr1	37,603,726	37,604,079	353	Loss
DEL00002587	chr1	37,634,298	37,634,406	108	Loss
DEL00002588	chr1	37,648,844	37,649,001	157	Loss
DEL00002602	chr1	37,918,603	37,918,927	324	Loss
DEL00002604	chr1	37,935,645	37,935,857	212	Loss
DEL00002607	chr1	37,977,331	37,978,819	1,488	Loss
DEL00002609	chr1	37,992,956	37,993,049	93	Loss
DEL00002610	chr1	37,993,202	37,993,378	176	Loss
DEL00002612	chr1	38,074,317	38,074,737	420	Loss
DEL00002613	chr1	38,109,019	38,109,099	80	Loss
DEL00002616	chr1	38,131,825	38,132,132	307	Loss
DEL00002622	chr1	38,272,702	38,273,073	371	Loss
DEL00002627	chr1	38,354,883	38,355,632	749	Loss
DEL00002628	chr1	38,368,548	38,368,783	235	Loss
DEL00002631	chr1	38,395,443	38,396,218	775	Loss
DEL00002632	chr1	38,409,296	38,409,518	222	Loss
DEL00002633	chr1	38,418,422	38,418,538	116	Loss
DEL00002636	chr1	38,497,802	38,497,871	69	Loss
DEL00002637	chr1	38,531,595	38,531,748	153	Loss
DEL00002641	chr1	38,617,530	38,618,026	496	Loss
DEL00002646	chr1	38,758,650	38,758,714	64	Loss
DEL00002648	chr1	38,778,729	38,779,042	313	Loss
DEL00002650	chr1	38,803,032	38,803,846	814	Loss
DEL00002653	chr1	38,918,551	38,918,602	51	Loss
DEL00002655	chr1	38,937,709	38,938,051	342	Loss
DEL00002656	chr1	38,946,502	38,946,561	59	Loss
DEL00002660	chr1	38,987,046	38,987,371	325	Loss
DEL00002661	chr1	39,013,266	39,013,524	258	Loss
DEL00002671	chr1	39,567,993	39,568,104	111	Loss
DEL00002674	chr1	39,677,476	39,677,847	371	Loss
DEL00002676	chr1	39,683,827	39,684,771	944	Loss
DEL00002678	chr1	39,703,295	39,703,512	217	Loss
DEL00002681	chr1	39,821,559	39,821,674	115	Loss
DEL00002682	chr1	39,869,981	39,870,124	143	Loss
DEL00002683	chr1	39,904,399	39,904,453	54	Loss
DEL00002684	chr1	39,918,161	39,918,302	141	Loss
DEL00002685	chr1	39,923,147	39,923,533	386	Loss
DEL00002686	chr1	39,946,522	39,946,597	75	Loss
DEL00002689	chr1	39,980,749	39,980,966	217	Loss
DEL00002690	chr1	39,981,796	39,981,853	57	Loss

DEL00002692	chr1	40,013,813	40,013,869	56	Loss
DEL00002697	chr1	40,098,677	40,100,402	1,725	Loss
DUP00002700	chr1	40,198,822	40,198,960	138	Gain
DEL00002708	chr1	40,332,500	40,333,025	525	Loss
DEL00002712	chr1	40,494,032	40,494,102	70	Loss
DEL00002713	chr1	40,499,688	40,499,775	87	Loss
DEL00002715	chr1	40,507,310	40,507,446	136	Loss
DEL00002716	chr1	40,508,245	40,508,868	623	Loss
DEL00002722	chr1	40,590,306	40,590,357	51	Loss
DEL00002728	chr1	40,726,108	40,726,837	729	Loss
DEL00002729	chr1	40,741,821	40,741,894	73	Loss
DEL00002734	chr1	40,809,187	40,809,474	287	Loss
DEL00002735	chr1	40,850,965	40,851,049	84	Loss
DEL00002741	chr1	41,103,603	41,104,112	509	Loss
DEL00002743	chr1	41,114,193	41,114,767	574	Loss
DEL00002746	chr1	41,222,775	41,224,248	1,473	Loss
DEL00002747	chr1	41,237,270	41,237,576	306	Loss
DEL00002749	chr1	41,297,980	41,298,971	991	Loss
DEL00002756	chr1	41,419,208	41,422,093	2,885	Loss
DEL00002759	chr1	41,457,237	41,457,851	614	Loss
DEL00002762	chr1	41,549,646	41,549,715	69	Loss
DEL00002763	chr1	41,563,526	41,563,643	117	Loss
DEL00002764	chr1	41,573,974	41,574,278	304	Loss
DEL00002766	chr1	41,592,680	41,592,888	208	Loss
DEL00002768	chr1	41,655,057	41,655,273	216	Loss
DEL00002771	chr1	41,895,161	41,896,082	921	Loss
DEL00002772	chr1	41,954,701	41,955,132	431	Loss
DEL00002782	chr1	42,249,613	42,249,794	181	Loss
DEL00002783	chr1	42,263,486	42,263,623	137	Loss
DEL00002784	chr1	42,332,671	42,332,735	64	Loss
DEL00002785	chr1	42,374,712	42,374,807	95	Loss
DEL00002786	chr1	42,375,881	42,382,339	6,458	Loss
DEL00002787	chr1	42,396,065	42,396,276	211	Loss
DEL00002788	chr1	42,424,631	42,425,520	889	Loss
DUP00002789	chr1	42,429,999	42,430,073	74	Gain
DEL00002791	chr1	42,558,143	42,558,203	60	Loss
DEL00002792	chr1	42,653,766	42,653,922	156	Loss
DEL00002795	chr1	42,812,785	42,813,451	666	Loss
DEL00002796	chr1	42,824,184	42,824,250	66	Loss
DEL00002801	chr1	42,975,574	42,975,698	124	Loss
DEL00002802	chr1	42,976,928	42,977,000	72	Loss
DEL00002804	chr1	43,093,508	43,093,563	55	Loss
DEL00002806	chr1	43,210,766	43,211,644	878	Loss
DEL00002813	chr1	43,575,944	43,576,149	205	Loss
DEL00002814	chr1	43,583,016	43,583,257	241	Loss
DEL00002821	chr1	43,844,438	43,844,501	63	Loss
DEL00002825	chr1	43,866,766	43,866,845	79	Loss
DEL00002826	chr1	43,920,387	43,925,486	5,099	Loss
DEL00002828	chr1	43,943,461	43,943,528	67	Loss

DEL00002831	chr1	44,042,789	44,042,976	187	Loss
DEL00002836	chr1	44,182,830	44,183,019	189	Loss
DEL00002841	chr1	44,244,849	44,245,197	348	Loss
DEL00002843	chr1	44,256,539	44,256,827	288	Loss
DEL00002849	chr1	44,390,837	44,390,896	59	Loss
DEL00002853	chr1	44,581,402	44,581,603	201	Loss
DEL00002860	chr1	44,653,983	44,654,087	104	Loss
DEL00002867	chr1	44,815,329	44,815,398	69	Loss
DEL00002871	chr1	44,914,978	44,915,085	107	Loss
DUP00002878	chr1	45,172,495	45,185,841	13,346	Gain
DEL00002880	chr1	45,270,370	45,270,465	95	Loss
DEL00002885	chr1	45,407,572	45,407,760	188	Loss
DEL00002887	chr1	45,419,974	45,420,143	169	Loss
DEL00002892	chr1	45,482,506	45,484,768	2,262	Loss
DEL00002894	chr1	45,500,217	45,500,292	75	Loss
DEL00002897	chr1	45,541,215	45,541,957	742	Loss
DEL00002900	chr1	45,570,707	45,570,970	263	Loss
DEL00002902	chr1	45,634,522	45,634,676	154	Loss
DEL00002905	chr1	45,672,289	45,672,599	310	Loss
DUP00002908	chr1	45,696,580	45,696,931	351	Gain
DEL00002911	chr1	45,730,737	45,731,346	609	Loss
DEL00002923	chr1	45,863,437	45,863,568	131	Loss
DEL00002930	chr1	46,010,905	46,011,136	231	Loss
DEL00002940	chr1	46,158,577	46,158,761	184	Loss
DEL00002941	chr1	46,161,614	46,162,105	491	Loss
DEL00002949	chr1	46,241,653	46,242,017	364	Loss
DEL00002954	chr1	46,262,205	46,262,436	231	Loss
DUP00002956	chr1	46,306,985	46,337,503	30,518	Mixed
DEL00002968	chr1	46,468,697	46,468,782	85	Loss
DEL00002973	chr1	46,576,114	46,576,500	386	Loss
DEL00002978	chr1	46,853,570	46,853,711	141	Loss
DEL00002979	chr1	46,864,901	46,864,991	90	Loss
DEL00002982	chr1	46,874,981	46,875,218	237	Loss
DEL00002985	chr1	46,986,793	46,986,972	179	Loss
DEL00002986	chr1	46,993,245	46,993,373	128	Loss
DEL00002992	chr1	47,154,862	47,156,305	1,443	Loss
DEL00002998	chr1	47,234,931	47,235,838	907	Loss
DEL00003001	chr1	47,303,379	47,303,470	91	Loss
DEL00003005	chr1	47,398,269	47,398,411	142	Loss
DEL00003009	chr1	47,430,132	47,430,680	548	Loss
DEL00003011	chr1	47,447,225	47,447,289	64	Loss
DEL00003012	chr1	47,488,888	47,489,019	131	Loss
DEL00003015	chr1	47,578,411	47,579,054	643	Loss
DEL00003017	chr1	47,704,762	47,705,002	240	Loss
DEL00003018	chr1	47,709,444	47,710,034	590	Loss
DEL00003026	chr1	47,774,172	47,774,280	108	Loss
DEL00003027	chr1	47,792,252	47,792,937	685	Loss
DEL00003036	chr1	47,963,829	47,963,916	87	Loss
DEL00003039	chr1	47,991,956	47,992,240	284	Loss

DEL00003040	chr1	48,003,786	48,004,348	562	Loss
DEL00003045	chr1	48,087,338	48,087,588	250	Loss
DEL00003046	chr1	48,095,507	48,095,608	101	Loss
DEL00003050	chr1	48,166,646	48,166,701	55	Loss
DEL00003053	chr1	48,192,516	48,192,571	55	Loss
DEL00003070	chr1	48,513,451	48,513,682	231	Loss
DEL00003074	chr1	48,563,488	48,563,549	61	Loss
DEL00003076	chr1	48,581,909	48,582,119	210	Loss
DEL00003087	chr1	48,890,752	48,890,829	77	Loss
DEL00003088	chr1	48,897,181	48,897,497	316	Loss
DEL00003089	chr1	48,898,155	48,898,911	756	Loss
DEL00003092	chr1	48,915,306	48,915,400	94	Loss
DEL00003093	chr1	48,917,543	48,917,970	427	Loss
DEL00003095	chr1	48,927,264	48,927,770	506	Loss
DEL00003097	chr1	48,941,052	48,941,186	134	Loss
DEL00003099	chr1	48,963,878	48,964,155	277	Loss
DEL00003101	chr1	48,967,513	48,967,762	249	Loss
DEL00003104	chr1	48,979,299	48,980,600	1,301	Loss
DEL00003106	chr1	49,003,897	49,004,124	227	Loss
DEL00003114	chr1	49,163,890	49,163,995	105	Loss
DEL00003115	chr1	49,197,018	49,197,508	490	Loss
DEL00003118	chr1	49,207,102	49,207,439	337	Loss
DEL00003123	chr1	49,234,684	49,234,743	59	Loss
DEL00003125	chr1	49,246,010	49,247,822	1,812	Loss
DEL00003141	chr1	49,559,054	49,559,788	734	Loss
DEL00003149	chr1	49,726,159	49,726,264	105	Loss
DEL00003154	chr1	49,831,473	49,832,218	745	Loss
DEL00003156	chr1	49,964,541	49,965,064	523	Loss
DEL00003157	chr1	50,054,894	50,054,951	57	Loss
DEL00003162	chr1	50,202,059	50,202,599	540	Loss
DEL00003165	chr1	50,300,065	50,300,288	223	Loss
DEL00003172	chr1	50,500,576	50,500,630	54	Loss
DEL00003183	chr1	50,696,733	50,697,160	427	Loss
DEL00003211	chr1	51,036,879	51,036,954	75	Loss
DEL00003214	chr1	51,192,079	51,192,172	93	Loss
DEL00003218	chr1	51,228,131	51,228,392	261	Loss
DEL00003225	chr1	51,467,367	51,467,507	140	Loss
DEL00003226	chr1	51,605,095	51,605,165	70	Loss
DEL00003230	chr1	51,688,603	51,688,941	338	Loss
DEL00003233	chr1	51,697,112	51,699,401	2,289	Loss
DEL00003243	chr1	51,890,034	51,890,234	200	Loss
DEL00003266	chr1	52,552,866	52,552,926	60	Loss
DEL00003267	chr1	52,565,987	52,566,580	593	Loss
DEL00003272	chr1	52,678,102	52,678,386	284	Loss
DUP00003273	chr1	52,682,037	52,692,527	10,490	Gain
DEL00003277	chr1	52,838,343	52,838,888	545	Loss
DEL00003280	chr1	52,840,614	52,841,214	600	Loss
DEL00003282	chr1	52,847,569	52,847,629	60	Loss
DEL00003285	chr1	52,942,351	52,946,984	4,633	Loss

DEL00003287	chr1	52,951,444	52,951,964	520	Loss
DEL00003292	chr1	53,039,719	53,039,783	64	Loss
DEL00003296	chr1	53,102,482	53,102,557	75	Loss
DEL00003297	chr1	53,157,449	53,157,930	481	Loss
DEL00003300	chr1	53,181,020	53,181,342	322	Loss
DEL00003303	chr1	53,307,440	53,307,690	250	Loss
DEL00003306	chr1	53,415,720	53,416,508	788	Loss
DEL00003307	chr1	53,439,105	53,439,193	88	Loss
DEL00003311	chr1	53,466,090	53,466,160	70	Loss
DEL00003314	chr1	53,505,678	53,505,730	52	Loss
DEL00003316	chr1	53,562,341	53,562,426	85	Loss
DEL00003323	chr1	53,761,223	53,761,476	253	Loss
DEL00003324	chr1	53,765,919	53,765,992	73	Loss
DEL00003328	chr1	53,809,839	53,809,919	80	Loss
DEL00003330	chr1	53,822,265	53,822,474	209	Loss
DEL00003332	chr1	53,842,484	53,843,269	785	Loss
DEL00003335	chr1	53,923,436	53,924,596	1,160	Loss
DEL00003338	chr1	53,976,498	53,976,553	55	Loss
DEL00003344	chr1	54,049,273	54,049,549	276	Loss
DEL00003348	chr1	54,062,587	54,062,641	54	Loss
DEL00003362	chr1	54,350,777	54,350,888	111	Loss
DEL00003364	chr1	54,362,432	54,362,938	506	Loss
DEL00003382	chr1	54,478,018	54,478,267	249	Loss
DEL00003384	chr1	54,496,491	54,496,753	262	Loss
DEL00003387	chr1	54,573,377	54,573,568	191	Loss
DEL00003390	chr1	54,644,183	54,644,272	89	Loss
DEL00003391	chr1	54,653,134	54,653,189	55	Loss
DEL00003395	chr1	54,821,415	54,821,486	71	Loss
DEL00003398	chr1	54,863,891	54,863,970	79	Loss
DEL00003401	chr1	54,891,724	54,893,774	2,050	Loss
DEL00003409	chr1	54,923,802	54,923,886	84	Loss
DEL00003411	chr1	54,985,589	54,985,809	220	Loss
DEL00003417	chr1	55,150,990	55,151,258	268	Loss
DEL00003419	chr1	55,249,810	55,249,888	78	Loss
DUP00003422	chr1	55,329,717	55,329,905	188	Gain
DEL00003423	chr1	55,361,527	55,361,884	357	Loss
DEL00003438	chr1	55,716,895	55,716,947	52	Loss
DEL00003447	chr1	55,899,633	55,899,689	56	Loss
DEL00003448	chr1	55,900,648	55,900,717	69	Loss
DEL00003454	chr1	56,096,434	56,096,602	168	Loss
DEL00003456	chr1	56,148,168	56,148,469	301	Loss
DEL00003461	chr1	56,379,215	56,379,638	423	Loss
DEL00003463	chr1	56,401,925	56,401,991	66	Loss
DEL00003465	chr1	56,453,486	56,453,564	78	Loss
DEL00003466	chr1	56,466,002	56,466,250	248	Loss
DEL00003467	chr1	56,473,354	56,473,436	82	Loss
DEL00003473	chr1	56,637,360	56,637,476	116	Loss
DEL00003475	chr1	56,658,843	56,659,089	246	Loss
DEL00003477	chr1	56,667,807	56,668,139	332	Loss

DEL00003481	chr1	56,786,914	56,787,071	157	Loss
DEL00003484	chr1	56,859,640	56,859,715	75	Loss
DEL00003485	chr1	56,865,335	56,865,399	64	Loss
DEL00003487	chr1	56,954,922	56,957,884	2,962	Loss
DEL00003494	chr1	57,044,056	57,044,152	96	Loss
DUP00003498	chr1	57,060,124	57,060,210	86	Gain
DEL00003499	chr1	57,061,779	57,061,842	63	Loss
DEL00003502	chr1	57,097,996	57,098,074	78	Loss
DEL00003503	chr1	57,105,615	57,105,847	232	Loss
DEL00003524	chr1	57,563,938	57,564,013	75	Loss
DEL00003527	chr1	57,889,717	57,890,743	1,026	Loss
DEL00003534	chr1	57,947,981	57,948,712	731	Loss
DUP00003536	chr1	57,981,231	58,001,511	20,280	Mixed
DEL00003541	chr1	58,068,036	58,068,103	67	Loss
DEL00003543	chr1	58,130,814	58,132,885	2,071	Loss
DEL00003551	chr1	58,339,330	58,339,405	75	Loss
DEL00003552	chr1	58,353,472	58,354,008	536	Loss
DEL00003555	chr1	58,429,313	58,429,636	323	Loss
DEL00003558	chr1	58,478,196	58,478,252	56	Loss
DEL00003561	chr1	58,562,604	58,563,084	480	Loss
DEL00003565	chr1	58,645,969	58,648,036	2,067	Loss
DEL00003566	chr1	58,672,363	58,673,319	956	Loss
DEL00003567	chr1	58,736,204	58,741,778	5,574	Loss
DEL00003568	chr1	58,742,460	58,742,677	217	Loss
DEL00003569	chr1	58,759,976	58,760,028	52	Loss
DEL00003570	chr1	58,766,105	58,766,587	482	Loss
DEL00003571	chr1	58,817,953	58,818,126	173	Loss
DEL00003572	chr1	58,842,831	58,842,882	51	Loss
DEL00003577	chr1	58,960,974	58,961,058	84	Loss
DEL00003578	chr1	58,995,532	58,995,596	64	Loss
DEL00003580	chr1	59,009,332	59,009,451	119	Loss
DEL00003583	chr1	59,079,557	59,079,853	296	Loss
DEL00003584	chr1	59,104,109	59,104,274	165	Loss
DEL00003587	chr1	59,140,518	59,140,634	116	Loss
DEL00003588	chr1	59,143,998	59,144,981	983	Loss
DEL00003592	chr1	59,199,866	59,199,950	84	Loss
DEL00003602	chr1	59,473,422	59,473,991	569	Loss
DEL00003606	chr1	59,562,201	59,562,318	117	Loss
DEL00003609	chr1	59,618,687	59,620,299	1,612	Loss
DEL00003611	chr1	59,630,807	59,630,860	53	Loss
DEL00003612	chr1	59,678,162	59,678,245	83	Loss
DEL00003614	chr1	59,723,089	59,723,400	311	Loss
DEL00003623	chr1	59,944,595	59,944,804	209	Loss
DEL00003624	chr1	59,950,192	59,950,389	197	Loss
DEL00003627	chr1	59,975,346	59,975,411	65	Loss
DEL00003628	chr1	59,975,480	59,976,767	1,287	Loss
DEL00003629	chr1	59,982,398	59,983,012	614	Loss
DEL00003631	chr1	60,034,504	60,034,613	109	Loss
DEL00003634	chr1	60,077,463	60,078,234	771	Loss

DEL00003641	chr1	60,308,095	60,308,148	53	Loss
DEL00003644	chr1	60,326,459	60,326,524	65	Loss
DEL00003646	chr1	60,360,572	60,360,644	72	Loss
DEL00003647	chr1	60,391,546	60,391,606	60	Loss
DEL00003648	chr1	60,408,386	60,408,634	248	Loss
DEL00003649	chr1	60,475,723	60,475,783	60	Loss
DEL00003650	chr1	60,491,239	60,491,530	291	Loss
DEL00003655	chr1	60,752,123	60,752,890	767	Loss
DEL00003656	chr1	60,823,527	60,824,447	920	Loss
DEL00003658	chr1	60,876,169	60,876,246	77	Loss
DEL00003660	chr1	60,984,960	60,985,216	256	Loss
DEL00003664	chr1	61,393,983	61,394,034	51	Loss
DEL00003666	chr1	61,509,632	61,509,695	63	Loss
DEL00003670	chr1	61,610,288	61,610,348	60	Loss
DEL00003671	chr1	61,689,864	61,689,935	71	Loss
DEL00003672	chr1	61,693,485	61,693,550	65	Loss
DEL00003678	chr1	61,881,331	61,881,476	145	Loss
DEL00003680	chr1	61,911,224	61,911,561	337	Loss
DEL00003683	chr1	61,961,979	61,963,158	1,179	Loss
DUP00003697	chr1	62,308,461	62,333,164	24,703	Mixed
DEL00003713	chr1	62,357,610	62,357,693	83	Loss
DEL00003715	chr1	62,399,292	62,399,349	57	Loss
DEL00003718	chr1	62,407,013	62,407,396	383	Loss
DEL00003721	chr1	62,595,909	62,596,086	177	Loss
DEL00003722	chr1	62,635,098	62,635,772	674	Loss
DEL00003729	chr1	62,693,818	62,693,875	57	Loss
DEL00003731	chr1	62,766,426	62,766,789	363	Loss
DEL00003733	chr1	62,804,037	62,804,099	62	Loss
DEL00003735	chr1	62,962,406	62,962,488	82	Loss
DEL00003736	chr1	62,980,592	62,981,412	820	Loss
DEL00003743	chr1	63,361,071	63,361,629	558	Loss
DEL00003745	chr1	63,407,537	63,407,605	68	Loss
DEL00003746	chr1	63,495,660	63,495,948	288	Loss
DEL00003747	chr1	63,554,537	63,554,681	144	Loss
DEL00003752	chr1	63,886,984	63,887,090	106	Loss
DEL00003753	chr1	63,896,947	63,897,522	575	Loss
DEL00003758	chr1	64,174,145	64,174,531	386	Loss
DEL00003759	chr1	64,195,536	64,195,999	463	Loss
DEL00003763	chr1	64,353,609	64,353,699	90	Loss
DEL00003764	chr1	64,385,548	64,385,708	160	Loss
DEL00003770	chr1	64,585,313	64,585,383	70	Loss
DEL00003771	chr1	64,585,714	64,585,806	92	Loss
DEL00003774	chr1	64,697,296	64,697,412	116	Loss
DEL00003778	chr1	64,973,089	64,974,999	1,910	Loss
DEL00003779	chr1	64,991,860	64,992,991	1,131	Loss
DEL00003783	chr1	65,318,201	65,322,405	4,204	Loss
DEL00003794	chr1	65,456,253	65,457,370	1,117	Loss
DEL00003795	chr1	65,471,695	65,472,126	431	Loss
DEL00003797	chr1	65,493,798	65,493,895	97	Loss

DUP00003800	chr1	65,554,131	65,554,291	160	Gain
DEL00003822	chr1	65,901,852	65,902,310	458	Loss
DEL00003829	chr1	66,122,282	66,122,367	85	Loss
DEL00003830	chr1	66,125,570	66,125,725	155	Loss
DEL00003831	chr1	66,145,485	66,146,941	1,456	Loss
DEL00003832	chr1	66,170,902	66,170,979	77	Loss
DEL00003835	chr1	66,192,979	66,193,646	667	Loss
DEL00003848	chr1	66,744,610	66,744,667	57	Loss
DEL00003850	chr1	66,756,255	66,756,355	100	Loss
DEL00003858	chr1	66,900,426	66,902,283	1,857	Loss
DEL00003866	chr1	67,077,709	67,078,149	440	Loss
DEL00003867	chr1	67,102,265	67,102,768	503	Loss
DEL00003870	chr1	67,129,975	67,130,253	278	Loss
DEL00003871	chr1	67,132,007	67,134,182	2,175	Loss
DEL00003873	chr1	67,139,852	67,139,945	93	Loss
DEL00003876	chr1	67,164,473	67,164,589	116	Loss
DEL00003877	chr1	67,190,938	67,193,079	2,141	Loss
DEL00003887	chr1	67,325,625	67,325,730	105	Loss
DEL00003889	chr1	67,363,215	67,363,401	186	Loss
DEL00003890	chr1	67,371,165	67,371,371	206	Loss
DEL00003892	chr1	67,434,935	67,434,987	52	Loss
DEL00003893	chr1	67,438,301	67,449,790	11,489	Loss
DEL00003900	chr1	67,469,952	67,470,043	91	Loss
DEL00003901	chr1	67,557,893	67,557,955	62	Loss
DEL00003903	chr1	67,597,427	67,597,581	154	Loss
DEL00003913	chr1	67,982,690	67,984,582	1,892	Loss
DEL00003914	chr1	68,002,145	68,003,117	972	Loss
DEL00003917	chr1	68,036,509	68,036,564	55	Loss
DEL00003921	chr1	68,084,955	68,085,384	429	Loss
DEL00003923	chr1	68,088,732	68,088,799	67	Loss
DEL00003926	chr1	68,175,260	68,175,374	114	Loss
DEL00003930	chr1	68,305,711	68,306,145	434	Loss
DEL00003934	chr1	68,329,237	68,329,520	283	Loss
DEL00003936	chr1	68,340,848	68,340,995	147	Loss
DEL00003937	chr1	68,365,133	68,365,375	242	Loss
DEL00003939	chr1	68,374,960	68,375,026	66	Loss
DEL00003942	chr1	68,479,105	68,479,700	595	Loss
DEL00003947	chr1	68,558,720	68,559,503	783	Loss
DEL00003949	chr1	68,606,293	68,606,725	432	Loss
DEL00003952	chr1	68,661,866	68,661,919	53	Loss
DEL00003956	chr1	68,721,822	68,722,093	271	Loss
DEL00003957	chr1	68,735,052	68,735,520	468	Loss
DEL00003960	chr1	68,821,922	68,822,487	565	Loss
DEL00003964	chr1	68,887,425	68,887,672	247	Loss
DEL00003970	chr1	69,047,938	69,048,180	242	Loss
DEL00003971	chr1	69,054,743	69,054,961	218	Loss
DEL00003977	chr1	69,101,173	69,101,366	193	Loss
DEL00003978	chr1	69,117,204	69,117,261	57	Loss
DEL00003979	chr1	69,157,070	69,157,184	114	Loss

DEL00003984	chr1	69,308,692	69,308,745	53	Loss
DEL00003986	chr1	69,375,146	69,375,463	317	Loss
DEL00003987	chr1	69,409,156	69,409,314	158	Loss
DEL00003994	chr1	69,543,490	69,544,026	536	Loss
DEL00003999	chr1	69,616,207	69,616,613	406	Loss
DEL00004001	chr1	69,626,896	69,627,079	183	Loss
DEL00004006	chr1	69,665,273	69,665,353	80	Loss
DEL00004007	chr1	69,675,977	69,676,057	80	Loss
DEL00004012	chr1	69,723,648	69,723,826	178	Loss
DEL00004015	chr1	69,725,429	69,725,782	353	Loss
DEL00004022	chr1	69,897,517	69,897,750	233	Loss
DEL00004026	chr1	69,947,880	69,948,211	331	Loss
DEL00004029	chr1	70,033,333	70,033,391	58	Loss
DEL00004032	chr1	70,073,598	70,074,073	475	Loss
DEL00004034	chr1	70,238,122	70,238,327	205	Loss
DEL00004035	chr1	70,263,284	70,263,517	233	Loss
DEL00004036	chr1	70,281,805	70,282,377	572	Loss
DUP00004047	chr1	70,412,994	70,413,297	303	Gain
DEL00004049	chr1	70,459,919	70,460,061	142	Loss
DEL00004050	chr1	70,460,747	70,460,902	155	Loss
DEL00004055	chr1	70,541,129	70,541,969	840	Loss
DEL00004057	chr1	70,626,839	70,627,194	355	Loss
DEL00004058	chr1	70,632,799	70,632,852	53	Loss
DEL00004059	chr1	70,658,953	70,659,090	137	Loss
DEL00004061	chr1	70,731,139	70,731,190	51	Loss
DEL00004064	chr1	70,801,409	70,801,878	469	Loss
DEL00004067	chr1	70,827,694	70,827,769	75	Loss
DUP00004071	chr1	70,892,912	70,893,025	113	Gain
DEL00004087	chr1	71,214,198	71,214,265	67	Loss
DEL00004092	chr1	71,266,146	71,266,211	65	Loss
DEL00004094	chr1	71,392,126	71,393,601	1,475	Loss
DEL00004128	chr1	71,542,943	71,544,122	1,179	Loss
DEL00004132	chr1	71,584,762	71,584,837	75	Loss
DEL00004139	chr1	71,860,495	71,860,940	445	Loss
DEL00004141	chr1	71,927,519	71,928,646	1,127	Loss
DEL00004145	chr1	72,020,007	72,020,103	96	Loss
DEL00004146	chr1	72,068,169	72,068,870	701	Loss
DEL00004150	chr1	72,160,583	72,160,796	213	Loss
DEL00004152	chr1	72,174,819	72,174,967	148	Loss
DEL00004154	chr1	72,241,861	72,241,936	75	Loss
DEL00004155	chr1	72,247,638	72,247,692	54	Loss
DEL00004157	chr1	72,254,952	72,255,290	338	Loss
DEL00004165	chr1	72,428,011	72,428,076	65	Loss
DEL00004167	chr1	72,440,951	72,441,118	167	Loss
DEL00004168	chr1	72,567,085	72,567,146	61	Loss
DEL00004169	chr1	72,622,161	72,622,335	174	Loss
DEL00004170	chr1	72,637,735	72,639,401	1,666	Loss
DEL00004171	chr1	72,747,403	72,750,360	2,957	Loss
DEL00004214	chr1	72,950,235	72,950,476	241	Loss

DEL00004218	chr1	73,049,776	73,049,828	52	Loss
DEL00004226	chr1	73,448,828	73,449,551	723	Loss
DEL00004228	chr1	73,467,235	73,468,241	1,006	Loss
DEL00004229	chr1	73,469,239	73,469,309	70	Loss
DEL00004235	chr1	73,665,905	73,665,972	67	Loss
DEL00004238	chr1	73,694,174	73,694,683	509	Loss
DEL00004246	chr1	73,932,753	73,933,216	463	Loss
DEL00004251	chr1	74,031,396	74,031,594	198	Loss
DEL00004257	chr1	74,450,889	74,451,226	337	Loss
DEL00004267	chr1	75,564,186	75,566,711	2,525	Loss
DEL00004268	chr1	75,636,773	75,636,895	122	Loss
DEL00004271	chr1	75,952,661	75,952,770	109	Loss
DEL00004277	chr1	76,173,297	76,174,569	1,272	Loss
DEL00004278	chr1	76,205,467	76,205,631	164	Loss
DEL00004283	chr1	76,453,944	76,454,770	826	Loss
DEL00004285	chr1	76,501,397	76,501,742	345	Loss
DEL00004286	chr1	76,519,971	76,520,072	101	Loss
DEL00004294	chr1	76,915,615	76,916,729	1,114	Loss
DEL00004302	chr1	77,014,980	77,015,078	98	Loss
DEL00004310	chr1	77,053,154	77,053,276	122	Loss
DEL00004311	chr1	77,059,203	77,059,644	441	Loss
DEL00004313	chr1	77,132,423	77,132,572	149	Loss
DEL00004317	chr1	77,240,071	77,240,203	132	Loss
DEL00004329	chr1	77,742,265	77,742,348	83	Loss
DEL00004333	chr1	77,838,581	77,839,010	429	Loss
DEL00004335	chr1	77,858,607	77,858,681	74	Loss
DEL00004337	chr1	77,960,397	77,960,880	483	Loss
DEL00004348	chr1	78,189,231	78,189,918	687	Loss
DEL00004349	chr1	78,197,665	78,197,718	53	Loss
DEL00004350	chr1	78,200,797	78,200,951	154	Loss
DEL00004405	chr1	78,277,407	78,278,084	677	Loss
DEL00004407	chr1	78,302,206	78,302,295	89	Loss
DEL00004408	chr1	78,313,758	78,314,297	539	Loss
DEL00004411	chr1	78,320,361	78,322,585	2,224	Loss
DEL00004412	chr1	78,332,297	78,333,143	846	Loss
DEL00004413	chr1	78,336,099	78,336,156	57	Loss
DEL00004415	chr1	78,370,241	78,371,877	1,636	Loss
DEL00004416	chr1	78,380,033	78,382,685	2,652	Loss
DEL00004417	chr1	78,385,797	78,391,576	5,779	Loss
DEL00004419	chr1	78,431,326	78,431,784	458	Loss
DEL00004421	chr1	78,460,885	78,462,088	1,203	Loss
DEL00004426	chr1	78,497,891	78,498,882	991	Loss
DEL00004456	chr1	78,537,716	78,539,059	1,343	Loss
DUP00004461	chr1	78,542,026	78,555,032	13,006	Gain
DEL00004486	chr1	78,564,142	78,633,747	69,605	Loss
DEL00004548	chr1	78,741,473	78,741,693	220	Loss
DEL00004550	chr1	78,743,888	78,744,209	321	Loss
DEL00004553	chr1	78,769,553	78,769,622	69	Loss
DEL00004558	chr1	78,799,204	78,799,260	56	Loss

DEL00004560	chr1	78,816,016	78,816,426	410	Loss
DEL00004563	chr1	78,854,421	78,854,717	296	Loss
DEL00004590	chr1	79,046,544	79,046,694	150	Loss
DEL00004597	chr1	79,125,771	79,127,442	1,671	Loss
DEL00004598	chr1	79,133,962	79,134,111	149	Loss
DEL00004602	chr1	79,202,910	79,202,991	81	Loss
DEL00004604	chr1	79,228,923	79,230,067	1,144	Loss
DUP00004610	chr1	79,281,621	79,281,699	78	Gain
DEL00004613	chr1	79,451,512	79,451,648	136	Loss
DEL00004617	chr1	79,527,059	79,527,144	85	Loss
DEL00004619	chr1	79,578,450	79,578,573	123	Loss
DEL00004621	chr1	79,589,922	79,590,092	170	Loss
DEL00004623	chr1	79,681,845	79,682,057	212	Loss
DEL00004624	chr1	79,702,693	79,702,900	207	Loss
DEL00004625	chr1	79,704,313	79,704,405	92	Loss
DEL00004634	chr1	79,899,997	79,900,610	613	Loss
DEL00004637	chr1	80,011,398	80,011,565	167	Loss
DEL00004638	chr1	80,055,304	80,055,563	259	Loss
DEL00004647	chr1	80,169,640	80,169,929	289	Loss
DEL00004652	chr1	80,241,840	80,242,556	716	Loss
DEL00004657	chr1	80,322,130	80,322,367	237	Loss
DEL00004663	chr1	80,370,552	80,370,703	151	Loss
DEL00004672	chr1	80,700,604	80,701,059	455	Loss
DEL00004675	chr1	80,770,548	80,771,315	767	Loss
DEL00004679	chr1	80,939,130	80,939,793	663	Loss
DEL00004683	chr1	80,955,103	80,955,307	204	Loss
DEL00004684	chr1	81,060,531	81,060,785	254	Loss
DEL00004687	chr1	81,070,417	81,070,519	102	Loss
DEL00004688	chr1	81,075,481	81,075,604	123	Loss
DEL00004696	chr1	81,255,753	81,255,982	229	Loss
DEL00004702	chr1	81,348,885	81,349,043	158	Loss
DEL00004703	chr1	81,413,861	81,414,122	261	Loss
DEL00004709	chr1	81,556,050	81,556,105	55	Loss
DEL00004713	chr1	81,643,697	81,643,750	53	Loss
DEL00004720	chr1	81,865,993	81,866,123	130	Loss
DEL00004725	chr1	81,966,498	81,966,690	192	Loss
DEL00004726	chr1	81,987,997	81,988,064	67	Loss
DEL00004728	chr1	82,052,708	82,053,018	310	Loss
DEL00004731	chr1	82,156,939	82,157,029	90	Loss
DEL00004732	chr1	82,201,958	82,202,212	254	Loss
DEL00004737	chr1	82,312,568	82,312,699	131	Loss
DEL00004744	chr1	82,766,472	82,766,980	508	Loss
DEL00004749	chr1	82,848,760	82,848,817	57	Loss
DEL00004752	chr1	82,977,235	82,977,492	257	Loss
DEL00004756	chr1	83,030,073	83,030,135	62	Loss
DEL00004758	chr1	83,174,332	83,174,389	57	Loss
DUP00004765	chr1	83,439,771	83,440,037	266	Gain
DEL00004770	chr1	83,572,613	83,573,241	628	Loss
DUP00004774	chr1	83,580,058	83,584,164	4,106	Mixed

DUP00004779	chr1	83,612,008	83,612,422	414	Gain
DEL00004785	chr1	83,614,449	83,614,899	450	Loss
DEL00004794	chr1	83,617,610	83,617,961	351	Loss
DUP00004800	chr1	83,619,010	83,619,531	521	Gain
DUP00004808	chr1	83,620,082	83,660,434	40,352	Mixed
DUP00004985	chr1	83,665,992	83,671,424	5,432	Gain
DUP00005029	chr1	83,709,367	83,709,690	323	Gain
DUP00005046	chr1	83,718,112	83,718,654	542	Gain
DUP00005106	chr1	83,767,454	83,768,098	644	Gain
DUP00005255	chr1	83,885,405	83,885,828	423	Gain
DUP00005433	chr1	84,025,686	84,026,005	319	Gain
DEL00005444	chr1	84,089,651	84,089,889	238	Loss
DEL00005450	chr1	84,274,972	84,275,984	1,012	Loss
DEL00005459	chr1	84,428,372	84,428,524	152	Loss
DEL00005462	chr1	84,430,986	84,431,233	247	Loss
DEL00005466	chr1	84,578,258	84,578,711	453	Loss
DEL00005467	chr1	84,596,102	84,597,375	1,273	Loss
DEL00005469	chr1	84,638,675	84,638,759	84	Loss
DEL00005470	chr1	84,673,188	84,673,239	51	Loss
DEL00005477	chr1	84,837,416	84,837,472	56	Loss
DEL00005490	chr1	85,049,987	85,050,048	61	Loss
DUP00005494	chr1	85,187,628	85,830,381	642,753	Mixed
DEL00005542	chr1	85,835,510	85,836,189	679	Loss
DUP00005546	chr1	85,907,872	85,917,400	9,528	Gain
DEL00005552	chr1	85,982,332	85,982,395	63	Loss
DEL00005553	chr1	86,018,852	86,019,351	499	Loss
DEL00005555	chr1	86,023,982	86,024,132	150	Loss
DEL00005556	chr1	86,026,583	86,026,645	62	Loss
DEL00005558	chr1	86,041,079	86,041,356	277	Loss
DEL00005563	chr1	86,092,247	86,092,299	52	Loss
DEL00005569	chr1	86,318,931	86,319,283	352	Loss
DEL00005571	chr1	86,322,126	86,322,291	165	Loss
DEL00005572	chr1	86,375,441	86,375,563	122	Loss
DEL00005573	chr1	86,390,100	86,390,185	85	Loss
DEL00005574	chr1	86,428,872	86,429,934	1,062	Loss
DEL00005576	chr1	86,451,543	86,452,078	535	Loss
DEL00005577	chr1	86,471,428	86,471,600	172	Loss
DEL00005578	chr1	86,474,082	86,474,742	660	Loss
DEL00005580	chr1	86,559,951	86,560,110	159	Loss
DEL00005584	chr1	86,600,635	86,602,550	1,915	Loss
DEL00005586	chr1	86,641,108	86,641,219	111	Loss
DEL00005589	chr1	86,800,320	86,804,742	4,422	Loss
DEL00005590	chr1	86,843,312	86,844,698	1,386	Loss
DEL00005591	chr1	86,845,981	86,846,294	313	Loss
DEL00005593	chr1	86,900,186	86,901,326	1,140	Loss
DEL00005594	chr1	87,027,426	87,027,483	57	Loss
DEL00005596	chr1	87,060,007	87,060,088	81	Loss
DEL00005598	chr1	87,144,999	87,145,634	635	Loss
DEL00005602	chr1	87,347,424	87,347,694	270	Loss

DEL00005608	chr1	87,524,966	87,525,859	893	Loss
DEL00005612	chr1	87,577,486	87,577,537	51	Loss
DEL00005616	chr1	87,628,215	87,628,267	52	Loss
DEL00005628	chr1	87,782,553	87,782,958	405	Loss
DEL00005632	chr1	87,870,170	87,871,535	1,365	Loss
DEL00005633	chr1	87,877,029	87,878,575	1,546	Loss
DEL00005634	chr1	87,881,014	87,881,067	53	Loss
DEL00005636	chr1	87,990,646	87,991,429	783	Loss
DEL00005642	chr1	88,070,512	88,070,702	190	Loss
DEL00005650	chr1	88,295,194	88,295,375	181	Loss
DEL00005653	chr1	88,344,917	88,345,044	127	Loss
DEL00005654	chr1	88,406,687	88,419,070	12,383	Loss
DEL00005656	chr1	88,425,702	88,425,793	91	Loss
DEL00005659	chr1	88,470,404	88,471,035	631	Loss
DEL00005662	chr1	88,506,794	88,506,865	71	Loss
DEL00005665	chr1	88,522,387	88,523,005	618	Loss
DEL00005666	chr1	88,567,377	88,567,440	63	Loss
DEL00005668	chr1	88,585,907	88,588,718	2,811	Loss
DEL00005672	chr1	88,630,965	88,631,405	440	Loss
DEL00005673	chr1	88,671,818	88,671,875	57	Loss
DEL00005676	chr1	88,717,228	88,717,350	122	Loss
DEL00005682	chr1	88,823,272	88,823,593	321	Loss
DEL00005685	chr1	88,824,042	88,824,415	373	Loss
DEL00005686	chr1	88,844,304	88,844,547	243	Loss
DUP00005688	chr1	88,893,097	88,893,248	151	Gain
DEL00005690	chr1	88,912,710	88,913,048	338	Loss
DEL00005694	chr1	89,025,434	89,025,542	108	Loss
DEL00005696	chr1	89,074,457	89,074,623	166	Loss
DEL00005697	chr1	89,078,160	89,079,944	1,784	Loss
DEL00005699	chr1	89,185,405	89,185,620	215	Loss
DEL00005700	chr1	89,209,739	89,209,968	229	Loss
DEL00005703	chr1	89,260,321	89,260,820	499	Loss
DEL00005712	chr1	89,447,157	89,447,527	370	Loss
DEL00005714	chr1	89,464,099	89,465,470	1,371	Loss
DEL00005724	chr1	89,646,456	89,646,512	56	Loss
DEL00005725	chr1	89,650,874	89,651,014	140	Loss
DEL00005727	chr1	89,693,770	89,693,832	62	Loss
DEL00005729	chr1	89,750,165	89,750,237	72	Loss
DEL00005731	chr1	89,775,521	89,775,767	246	Loss
DEL00005732	chr1	89,798,820	89,799,700	880	Loss
DEL00005733	chr1	89,803,180	89,803,909	729	Loss
DEL00005740	chr1	90,122,693	90,123,100	407	Loss
DEL00005742	chr1	90,159,611	90,160,123	512	Loss
DEL00005745	chr1	90,172,416	90,172,470	54	Loss
DEL00005749	chr1	90,382,976	90,383,078	102	Loss
DEL00005750	chr1	90,397,483	90,397,956	473	Loss
DEL00005751	chr1	90,397,976	90,398,031	55	Loss
DEL00005752	chr1	90,411,366	90,411,613	247	Loss
DEL00005753	chr1	90,440,272	90,440,325	53	Loss

DEL00005757	chr1	90,505,242	90,505,927	685	Loss
DUP00005759	chr1	90,549,550	90,549,608	58	Gain
DEL00005760	chr1	90,554,302	90,554,367	65	Loss
DEL00005761	chr1	90,559,621	90,559,685	64	Loss
DEL00005764	chr1	90,573,909	90,573,960	51	Loss
DEL00005770	chr1	90,632,286	90,632,479	193	Loss
DEL00005771	chr1	90,635,055	90,635,564	509	Loss
DEL00005774	chr1	90,699,548	90,700,357	809	Loss
DEL00005780	chr1	90,797,988	90,798,364	376	Loss
DEL00005785	chr1	90,914,121	90,914,442	321	Loss
DEL00005786	chr1	90,933,189	90,933,252	63	Loss
DEL00005794	chr1	91,072,766	91,073,196	430	Loss
DEL00005796	chr1	91,097,711	91,097,797	86	Loss
DEL00005797	chr1	91,107,537	91,108,401	864	Loss
DEL00005799	chr1	91,114,009	91,114,147	138	Loss
DEL00005801	chr1	91,128,458	91,131,062	2,604	Loss
DEL00005803	chr1	91,169,750	91,170,236	486	Loss
DEL00005805	chr1	91,180,070	91,180,171	101	Loss
DEL00005807	chr1	91,189,059	91,189,127	68	Loss
DEL00005808	chr1	91,191,027	91,191,791	764	Loss
DEL00005809	chr1	91,216,565	91,217,057	492	Loss
DEL00005811	chr1	91,217,924	91,218,172	248	Loss
DEL00005814	chr1	91,242,165	91,242,368	203	Loss
DEL00005820	chr1	91,294,809	91,295,129	320	Loss
DEL00005821	chr1	91,299,817	91,299,874	57	Loss
DEL00005827	chr1	91,347,027	91,347,348	321	Loss
DEL00005828	chr1	91,350,452	91,350,596	144	Loss
DEL00005830	chr1	91,376,324	91,377,483	1,159	Loss
DEL00005832	chr1	91,446,661	91,446,883	222	Loss
DUP00005834	chr1	91,474,679	91,499,101	24,422	Mixed
DUP00005841	chr1	91,530,671	91,530,853	182	Gain
DEL00005844	chr1	91,577,203	91,578,110	907	Loss
DEL00005845	chr1	91,578,637	91,578,882	245	Loss
DEL00005846	chr1	91,593,261	91,593,916	655	Loss
DEL00005847	chr1	91,596,767	91,597,166	399	Loss
DEL00005854	chr1	91,694,607	91,694,736	129	Loss
DEL00005855	chr1	91,719,093	91,719,450	357	Loss
DEL00005860	chr1	91,761,208	91,761,600	392	Loss
DEL00005863	chr1	91,783,832	91,783,913	81	Loss
DEL00005875	chr1	91,958,643	91,958,697	54	Loss
DEL00005881	chr1	92,013,833	92,013,898	65	Loss
DEL00005888	chr1	92,149,362	92,149,466	104	Loss
DEL00005891	chr1	92,158,184	92,158,237	53	Loss
DEL00005892	chr1	92,160,911	92,161,027	116	Loss
DEL00005898	chr1	92,256,939	92,257,665	726	Loss
DEL00005899	chr1	92,276,116	92,276,608	492	Loss
DEL00005902	chr1	92,290,642	92,290,700	58	Loss
DEL00005912	chr1	92,368,903	92,369,014	111	Loss
DEL00005913	chr1	92,405,211	92,405,287	76	Loss

DEL00005916	chr1	92,411,570	92,411,708	138	Loss
DEL00005918	chr1	92,486,420	92,486,808	388	Loss
DEL00005920	chr1	92,525,490	92,525,601	111	Loss
DEL00005924	chr1	92,555,036	92,561,848	6,812	Loss
DEL00005925	chr1	92,595,275	92,595,742	467	Loss
DEL00005926	chr1	92,616,200	92,618,508	2,308	Loss
DEL00005927	chr1	92,625,430	92,627,375	1,945	Loss
DEL00005929	chr1	92,667,768	92,667,837	69	Loss
DEL00005932	chr1	92,719,426	92,719,808	382	Loss
DEL00005933	chr1	92,728,123	92,728,255	132	Loss
DEL00005940	chr1	92,851,560	92,851,663	103	Loss
DEL00005942	chr1	92,876,355	92,876,427	72	Loss
DEL00005950	chr1	92,987,849	92,988,224	375	Loss
DEL00005953	chr1	93,112,168	93,112,219	51	Loss
DEL00005960	chr1	93,307,634	93,308,539	905	Loss
DEL00005964	chr1	93,340,431	93,343,521	3,090	Loss
DEL00005965	chr1	93,348,684	93,348,759	75	Loss
DEL00005966	chr1	93,351,763	93,352,098	335	Loss
DEL00005971	chr1	93,374,280	93,374,796	516	Loss
DEL00005974	chr1	93,402,332	93,402,892	560	Loss
DUP00005975	chr1	93,410,536	93,410,804	268	Gain
DEL00005977	chr1	93,450,710	93,450,804	94	Loss
DEL00005981	chr1	93,556,300	93,556,536	236	Loss
DEL00005982	chr1	93,558,840	93,562,024	3,184	Loss
DEL00005991	chr1	93,657,897	93,657,956	59	Loss
DEL00005992	chr1	93,674,963	93,675,328	365	Loss
DEL00005999	chr1	93,915,980	93,916,129	149	Loss
DEL00006002	chr1	94,002,017	94,002,229	212	Loss
DEL00006004	chr1	94,040,345	94,040,604	259	Loss
DEL00006016	chr1	94,296,585	94,297,904	1,319	Loss
DEL00006017	chr1	94,305,179	94,305,265	86	Loss
DEL00006019	chr1	94,450,125	94,452,167	2,042	Loss
DEL00006023	chr1	94,569,486	94,571,751	2,265	Loss
DUP00006030	chr1	94,733,765	94,734,081	316	Gain
DEL00006031	chr1	94,747,933	94,748,732	799	Loss
DEL00006034	chr1	94,858,207	94,858,549	342	Loss
DEL00006039	chr1	95,072,966	95,073,021	55	Loss
DEL00006045	chr1	95,160,137	95,160,567	430	Loss
DEL00006047	chr1	95,177,121	95,179,458	2,337	Loss
DEL00006049	chr1	95,194,510	95,195,925	1,415	Loss
DEL00006052	chr1	95,237,682	95,238,631	949	Loss
DEL00006054	chr1	95,364,889	95,379,845	14,956	Loss
DEL00006055	chr1	95,381,575	95,381,735	160	Loss
DEL00006057	chr1	95,591,509	95,593,898	2,389	Loss
DEL00006060	chr1	95,634,623	95,634,872	249	Loss
DEL00006061	chr1	95,645,777	95,646,007	230	Loss
DEL00006062	chr1	95,653,509	95,653,934	425	Loss
DEL00006063	chr1	95,656,517	95,659,023	2,506	Loss
DEL00006065	chr1	95,661,254	95,661,586	332	Loss

DEL00006068	chr1	95,725,626	95,726,062	436	Loss
DEL00006071	chr1	95,780,311	95,782,679	2,368	Loss
DEL00006073	chr1	95,794,860	95,795,191	331	Loss
DEL00006074	chr1	95,799,365	95,800,205	840	Loss
DEL00006078	chr1	95,833,985	95,857,348	23,363	Loss
DEL00006080	chr1	95,915,926	95,918,050	2,124	Loss
DEL00006083	chr1	95,959,036	95,959,214	178	Loss
DEL00006090	chr1	96,019,422	96,019,515	93	Loss
DEL00006092	chr1	96,033,798	96,034,031	233	Loss
DUP00006097	chr1	96,223,330	96,224,017	687	Gain
DEL00006105	chr1	96,311,678	96,312,419	741	Loss
DEL00006115	chr1	96,377,958	96,378,193	235	Loss
DEL00006116	chr1	96,428,066	96,428,171	105	Loss
DEL00006118	chr1	96,475,623	96,482,693	7,070	Loss
DEL00006121	chr1	96,537,083	96,537,942	859	Loss
DEL00006127	chr1	96,694,217	96,694,303	86	Loss
DEL00006128	chr1	96,740,149	96,740,216	67	Loss
DEL00006131	chr1	96,772,606	96,772,684	78	Loss
DEL00006132	chr1	96,803,046	96,804,575	1,529	Loss
DEL00006134	chr1	96,832,893	96,833,313	420	Loss
DEL00006136	chr1	96,851,670	96,856,327	4,657	Loss
DEL00006139	chr1	96,947,962	96,948,226	264	Loss
DEL00006145	chr1	97,295,166	97,295,417	251	Loss
DEL00006147	chr1	97,326,825	97,327,151	326	Loss
DEL00006148	chr1	97,338,407	97,338,944	537	Loss
DEL00006154	chr1	97,674,557	97,675,091	534	Loss
DEL00006164	chr1	97,831,755	97,832,059	304	Loss
DEL00006166	chr1	97,986,412	97,986,477	65	Loss
DEL00006168	chr1	98,030,909	98,032,443	1,534	Loss
DEL00006169	chr1	98,083,954	98,084,876	922	Loss
DEL00006172	chr1	98,396,222	98,397,164	942	Loss
DEL00006173	chr1	98,415,127	98,415,619	492	Loss
DEL00006176	chr1	98,643,428	98,643,590	162	Loss
DEL00006177	chr1	98,651,558	98,651,637	79	Loss
DEL00006179	chr1	98,724,626	98,725,115	489	Loss
DEL00006180	chr1	98,728,841	98,729,942	1,101	Loss
DEL00006181	chr1	98,772,095	98,772,235	140	Loss
DEL00006182	chr1	98,788,773	98,788,974	201	Loss
DEL00006188	chr1	98,904,759	98,905,471	712	Loss
DEL00006189	chr1	98,925,128	98,925,196	68	Loss
DEL00006193	chr1	98,954,061	98,954,127	66	Loss
DEL00006197	chr1	99,077,175	99,077,239	64	Loss
DEL00006206	chr1	99,360,498	99,360,640	142	Loss
DEL00006209	chr1	99,389,335	99,389,803	468	Loss
DEL00006212	chr1	99,464,686	99,464,812	126	Loss
DEL00006215	chr1	99,502,146	99,502,247	101	Loss
DEL00006220	chr1	99,571,875	99,572,485	610	Loss
DEL00006222	chr1	99,641,018	99,641,314	296	Loss
DEL00006225	chr1	99,754,333	99,754,426	93	Loss

DEL00006226	chr1	99,770,305	99,770,356	51	Loss
DEL00006231	chr1	99,997,847	99,998,031	184	Loss
DEL00006235	chr1	100,058,055	100,058,125	70	Loss
DEL00006238	chr1	100,130,528	100,130,658	130	Loss
DEL00006240	chr1	100,182,876	100,183,469	593	Loss
DEL00006242	chr1	100,232,842	100,233,548	706	Loss
DEL00006243	chr1	100,235,134	100,236,039	905	Loss
DEL00006245	chr1	100,245,957	100,246,435	478	Loss
DEL00006247	chr1	100,260,607	100,260,815	208	Loss
DEL00006248	chr1	100,273,378	100,273,520	142	Loss
DEL00006249	chr1	100,304,778	100,306,077	1,299	Loss
DEL00006251	chr1	100,381,984	100,382,138	154	Loss
DEL00006252	chr1	100,411,226	100,411,915	689	Loss
DEL00006254	chr1	100,479,393	100,480,308	915	Loss
DEL00006255	chr1	100,497,206	100,497,429	223	Loss
DEL00006257	chr1	100,514,255	100,514,331	76	Loss
DEL00006260	chr1	100,574,216	100,574,331	115	Loss
DEL00006271	chr1	100,738,005	100,738,061	56	Loss
DEL00006272	chr1	100,741,530	100,741,676	146	Loss
DEL00006273	chr1	100,743,218	100,743,767	549	Loss
DEL00006276	chr1	100,770,692	100,770,829	137	Loss
DEL00006282	chr1	100,818,176	100,820,590	2,414	Loss
DEL00006283	chr1	100,848,652	100,848,748	96	Loss
DEL00006289	chr1	100,906,815	100,906,902	87	Loss
DEL00006290	chr1	100,944,907	100,945,326	419	Loss
DEL00006291	chr1	100,957,372	100,957,514	142	Loss
DEL00006293	chr1	100,960,739	100,961,161	422	Loss
DEL00006294	chr1	101,000,285	101,000,986	701	Loss
DEL00006296	chr1	101,010,051	101,010,227	176	Loss
DEL00006297	chr1	101,055,133	101,055,644	511	Loss
DEL00006298	chr1	101,082,096	101,082,148	52	Loss
DEL00006302	chr1	101,156,985	101,159,555	2,570	Loss
DEL00006306	chr1	101,171,508	101,171,735	227	Loss
DEL00006307	chr1	101,200,308	101,202,302	1,994	Loss
DEL00006310	chr1	101,231,386	101,231,870	484	Loss
DEL00006311	chr1	101,234,388	101,237,248	2,860	Loss
DEL00006312	chr1	101,244,985	101,245,129	144	Loss
DEL00006313	chr1	101,356,512	101,357,007	495	Loss
DEL00006316	chr1	101,480,661	101,480,971	310	Loss
DEL00006321	chr1	101,584,026	101,586,406	2,380	Loss
DEL00006327	chr1	101,703,774	101,705,450	1,676	Loss
DEL00006329	chr1	101,741,079	101,743,498	2,419	Loss
DEL00006333	chr1	102,173,155	102,173,367	212	Loss
DEL00006334	chr1	102,203,835	102,659,666	455,831	Loss
DEL00006360	chr1	102,697,604	102,698,107	503	Loss
DEL00006364	chr1	102,723,677	102,723,806	129	Loss
DEL00006365	chr1	102,774,612	102,774,839	227	Loss
DEL00006371	chr1	102,818,954	102,819,750	796	Loss
DEL00006373	chr1	102,832,537	102,832,990	453	Loss

DEL00006380	chr1	102,952,900	102,953,313	413	Loss
DEL00006385	chr1	102,993,422	102,993,482	60	Loss
DEL00006386	chr1	103,011,042	103,011,216	174	Loss
DEL00006391	chr1	103,086,765	103,090,975	4,210	Loss
DEL00006393	chr1	103,110,400	103,113,532	3,132	Loss
DEL00006394	chr1	103,115,864	103,116,973	1,109	Loss
DEL00006395	chr1	103,130,573	103,135,541	4,968	Loss
DEL00006396	chr1	103,163,631	103,165,079	1,448	Loss
DEL00006397	chr1	103,182,624	103,182,748	124	Loss
DEL00006398	chr1	103,189,178	103,190,773	1,595	Loss
DEL00006401	chr1	103,230,596	103,230,793	197	Loss
DEL00006402	chr1	103,231,415	103,231,468	53	Loss
DUP00006405	chr1	103,319,583	103,319,716	133	Gain
DEL00006406	chr1	103,339,257	103,340,192	935	Loss
DEL00006407	chr1	103,358,139	103,364,223	6,084	Loss
DEL00006410	chr1	103,372,633	103,372,892	259	Loss
DEL00006414	chr1	103,478,187	103,478,264	77	Loss
DEL00006415	chr1	103,480,711	103,481,425	714	Loss
DEL00006417	chr1	103,747,869	103,748,598	729	Loss
DEL00006422	chr1	103,911,994	103,912,049	55	Loss
DEL00006425	chr1	104,025,755	104,025,822	67	Loss
DEL00006432	chr1	104,175,992	104,176,556	564	Loss
DEL00006434	chr1	104,206,530	104,206,640	110	Loss
DEL00006443	chr1	104,391,160	104,391,959	799	Loss
DEL00006449	chr1	104,471,885	104,473,339	1,454	Loss
DEL00006450	chr1	104,473,660	104,473,775	115	Loss
DEL00006451	chr1	104,484,060	104,484,114	54	Loss
DEL00006453	chr1	104,497,482	104,497,894	412	Loss
DEL00006457	chr1	104,535,644	104,535,941	297	Loss
DEL00006460	chr1	104,606,708	104,606,778	70	Loss
DEL00006465	chr1	104,661,014	104,661,238	224	Loss
DEL00006467	chr1	104,721,306	104,721,665	359	Loss
DEL00006469	chr1	104,783,359	104,783,613	254	Loss
DEL00006486	chr1	105,065,036	105,065,150	114	Loss
DEL00006492	chr1	105,097,761	105,097,817	56	Loss
DEL00006497	chr1	105,113,823	105,113,907	84	Loss
DEL00006499	chr1	105,192,514	105,192,571	57	Loss
DEL00006502	chr1	105,219,642	105,219,906	264	Loss
DEL00006504	chr1	105,236,738	105,236,897	159	Loss
DEL00006506	chr1	105,350,500	105,350,997	497	Loss
DEL00006508	chr1	105,351,317	105,352,054	737	Loss
DEL00006509	chr1	105,368,184	105,368,695	511	Loss
DEL00006511	chr1	105,384,827	105,384,927	100	Loss
DEL00006517	chr1	105,463,942	105,464,152	210	Loss
DEL00006523	chr1	105,540,801	105,541,823	1,022	Loss
DEL00006526	chr1	105,618,683	105,618,746	63	Loss
DEL00006535	chr1	105,731,041	105,731,347	306	Loss
DEL00006536	chr1	105,752,534	105,752,667	133	Loss
DEL00006537	chr1	105,772,362	105,772,507	145	Loss

DEL00006541	chr1	105,834,921	105,835,087	166	Loss
DEL00006545	chr1	105,879,106	105,879,339	233	Loss
DEL00006547	chr1	105,941,047	105,941,177	130	Loss
DEL00006548	chr1	105,945,297	105,945,412	115	Loss
DEL00006550	chr1	106,018,263	106,018,811	548	Loss
DEL00006561	chr1	106,199,782	106,200,071	289	Loss
DEL00006563	chr1	106,215,693	106,215,882	189	Loss
DEL00006570	chr1	106,349,000	106,349,097	97	Loss
DEL00006578	chr1	106,569,583	106,570,418	835	Loss
DEL00006579	chr1	106,573,670	106,573,749	79	Loss
DEL00006580	chr1	106,594,226	106,594,305	79	Loss
DEL00006585	chr1	106,661,836	106,662,553	717	Loss
DEL00006591	chr1	106,756,570	106,756,727	157	Loss
DEL00006592	chr1	106,762,536	106,762,699	163	Loss
DEL00006595	chr1	106,890,050	106,890,119	69	Loss
DEL00006596	chr1	106,898,060	106,898,533	473	Loss
DEL00006599	chr1	106,918,722	106,919,003	281	Loss
DEL00006609	chr1	107,182,552	107,183,113	561	Loss
DEL00006610	chr1	107,221,072	107,221,183	111	Loss
DEL00006614	chr1	107,294,494	107,294,616	122	Loss
DEL00006618	chr1	107,424,950	107,425,007	57	Loss
DEL00006624	chr1	107,486,434	107,489,312	2,878	Loss
DEL00006642	chr1	107,965,875	107,966,479	604	Loss
DUP00006645	chr1	108,004,938	108,005,005	67	Gain
DEL00006646	chr1	108,012,113	108,012,226	113	Loss
DEL00006650	chr1	108,086,245	108,086,297	52	Loss
DEL00006653	chr1	108,163,899	108,163,974	75	Loss
DEL00006656	chr1	108,224,591	108,224,645	54	Loss
DEL00006662	chr1	108,270,000	108,270,057	57	Loss
DEL00006664	chr1	108,296,585	108,296,695	110	Loss
DEL00006673	chr1	108,537,358	108,537,566	208	Loss
DEL00006674	chr1	108,555,289	108,555,485	196	Loss
DEL00006675	chr1	108,560,419	108,560,533	114	Loss
DEL00006676	chr1	108,574,581	108,574,870	289	Loss
DEL00006688	chr1	108,924,138	108,924,210	72	Loss
DEL00006690	chr1	108,994,896	108,996,280	1,384	Loss
DEL00006704	chr1	109,350,398	109,350,728	330	Loss
DEL00006705	chr1	109,369,263	109,369,316	53	Loss
DUP00006707	chr1	109,376,041	109,376,229	188	Gain
DEL00006708	chr1	109,376,851	109,376,927	76	Loss
DEL00006709	chr1	109,380,161	109,380,637	476	Loss
DEL00006710	chr1	109,437,857	109,438,064	207	Loss
DEL00006711	chr1	109,447,753	109,447,809	56	Loss
DEL00006725	chr1	109,862,553	109,863,848	1,295	Loss
DEL00006727	chr1	109,941,409	109,941,506	97	Loss
DEL00006728	chr1	109,973,668	109,978,144	4,476	Loss
DEL00006733	chr1	110,107,564	110,108,252	688	Loss
DEL00006734	chr1	110,109,956	110,110,996	1,040	Loss
DEL00006738	chr1	110,232,105	110,232,590	485	Loss

DEL00006740	chr1	110,273,192	110,273,740	548	Loss
DUP00006784	chr1	110,337,896	110,338,433	537	Gain
DEL00006809	chr1	110,483,063	110,483,152	89	Loss
DEL00006811	chr1	110,506,389	110,506,811	422	Loss
DUP00006813	chr1	110,510,087	110,510,168	81	Gain
DEL00006821	chr1	110,746,275	110,746,328	53	Loss
DEL00006832	chr1	111,051,977	111,052,075	98	Loss
DEL00006833	chr1	111,073,443	111,073,643	200	Loss
DEL00006834	chr1	111,079,613	111,079,848	235	Loss
DEL00006835	chr1	111,083,093	111,083,215	122	Loss
DEL00006842	chr1	111,159,922	111,160,014	92	Loss
DEL00006846	chr1	111,300,520	111,300,591	71	Loss
DEL00006847	chr1	111,300,820	111,300,878	58	Loss
DEL00006848	chr1	111,328,791	111,329,222	431	Loss
DEL00006851	chr1	111,354,099	111,354,237	138	Loss
DEL00006858	chr1	111,468,233	111,468,315	82	Loss
DEL00006859	chr1	111,469,287	111,469,497	210	Loss
DEL00006862	chr1	111,538,498	111,538,673	175	Loss
DEL00006865	chr1	111,568,367	111,568,418	51	Loss
DEL00006867	chr1	111,625,174	111,625,236	62	Loss
DEL00006868	chr1	111,745,771	111,745,850	79	Loss
DEL00006873	chr1	111,952,851	111,953,198	347	Loss
DEL00006880	chr1	112,086,681	112,086,860	179	Loss
DEL00006889	chr1	112,235,347	112,236,179	832	Loss
DEL00006890	chr1	112,272,516	112,272,581	65	Loss
DEL00006893	chr1	112,290,509	112,290,774	265	Loss
DEL00006895	chr1	112,387,601	112,387,939	338	Loss
DEL00006897	chr1	112,406,916	112,406,972	56	Loss
DEL00006902	chr1	112,510,901	112,511,714	813	Loss
DEL00006904	chr1	112,558,859	112,559,492	633	Loss
DEL00006909	chr1	112,616,987	112,617,415	428	Loss
DEL00006912	chr1	112,743,672	112,743,767	95	Loss
DEL00006913	chr1	112,761,854	112,761,988	134	Loss
DEL00006923	chr1	112,908,598	112,908,680	82	Loss
DEL00006924	chr1	112,912,459	112,912,525	66	Loss
DEL00006925	chr1	112,923,168	112,923,238	70	Loss
DEL00006926	chr1	112,929,260	112,929,983	723	Loss
DEL00006930	chr1	112,962,466	112,962,577	111	Loss
DEL00006931	chr1	113,008,431	113,008,706	275	Loss
DEL00006933	chr1	113,023,741	113,025,175	1,434	Loss
DUP00006937	chr1	113,043,248	113,043,309	61	Gain
DEL00006941	chr1	113,278,940	113,279,463	523	Loss
DEL00006943	chr1	113,324,907	113,325,727	820	Loss
DEL00006947	chr1	113,354,556	113,354,712	156	Loss
DUP00006950	chr1	113,370,101	113,370,176	75	Gain
DEL00006953	chr1	113,410,601	113,410,878	277	Loss
DEL00006954	chr1	113,418,212	113,422,846	4,634	Loss
DEL00006971	chr1	113,720,233	113,722,201	1,968	Loss
DEL00006974	chr1	113,723,407	113,723,609	202	Loss

DEL00006978	chr1	113,813,682	113,813,773	91	Loss
DEL00006982	chr1	113,951,879	113,951,961	82	Loss
DEL00006986	chr1	114,016,604	114,017,008	404	Loss
DEL00006988	chr1	114,059,791	114,059,910	119	Loss
DEL00006995	chr1	114,181,799	114,181,867	68	Loss
DEL00006999	chr1	114,232,479	114,232,632	153	Loss
DEL00007001	chr1	114,280,420	114,280,526	106	Loss
DEL00007002	chr1	114,295,583	114,295,819	236	Loss
DEL00007014	chr1	114,509,769	114,509,863	94	Loss
DEL00007016	chr1	114,578,575	114,579,051	476	Loss
DEL00007019	chr1	114,635,229	114,635,300	71	Loss
DEL00007020	chr1	114,646,532	114,646,795	263	Loss
DEL00007025	chr1	114,727,851	114,728,899	1,048	Loss
DEL00007026	chr1	114,732,047	114,732,127	80	Loss
DEL00007029	chr1	114,744,308	114,744,449	141	Loss
DEL00007033	chr1	114,771,291	114,771,939	648	Loss
DEL00007042	chr1	115,049,588	115,049,668	80	Loss
DEL00007043	chr1	115,063,103	115,063,162	59	Loss
DEL00007052	chr1	115,154,784	115,154,882	98	Loss
DEL00007061	chr1	115,286,133	115,286,205	72	Loss
DEL00007062	chr1	115,286,821	115,287,340	519	Loss
DEL00007076	chr1	115,426,886	115,427,443	557	Loss
DEL00007078	chr1	115,437,033	115,437,098	65	Loss
DEL00007079	chr1	115,456,157	115,456,558	401	Loss
DUP00007080	chr1	115,473,889	115,473,958	69	Gain
DEL00007081	chr1	115,479,352	115,480,036	684	Loss
DEL00007085	chr1	115,558,125	115,558,321	196	Loss
DEL00007087	chr1	115,575,573	115,575,689	116	Loss
DEL00007089	chr1	115,653,028	115,653,448	420	Loss
DEL00007096	chr1	115,782,643	115,783,279	636	Loss
DEL00007099	chr1	115,793,106	115,794,216	1,110	Loss
DEL00007106	chr1	115,876,460	115,876,740	280	Loss
DEL00007109	chr1	115,964,814	115,965,460	646	Loss
DEL00007110	chr1	115,965,986	115,966,048	62	Loss
DEL00007115	chr1	116,027,704	116,028,195	491	Loss
DEL00007119	chr1	116,062,838	116,062,905	67	Loss
DEL00007124	chr1	116,130,303	116,130,888	585	Loss
DEL00007133	chr1	116,250,356	116,251,166	810	Loss
DUP00007134	chr1	116,256,740	116,256,990	250	Gain
DEL00007142	chr1	116,380,226	116,380,350	124	Loss
DEL00007143	chr1	116,405,527	116,406,566	1,039	Loss
DEL00007148	chr1	116,519,318	116,519,396	78	Loss
DEL00007157	chr1	116,619,254	116,619,615	361	Loss
DEL00007158	chr1	116,626,647	116,626,817	170	Loss
DEL00007159	chr1	116,627,622	116,627,673	51	Loss
DEL00007160	chr1	116,684,466	116,687,331	2,865	Loss
DEL00007163	chr1	116,695,886	116,695,939	53	Loss
DEL00007192	chr1	117,300,307	117,300,890	583	Loss
DEL00007193	chr1	117,307,128	117,307,287	159	Loss

DEL00007195	chr1	117,439,665	117,439,730	65	Loss
DEL00007200	chr1	117,632,967	117,634,042	1,075	Loss
DEL00007204	chr1	117,773,692	117,774,399	707	Loss
DEL00007207	chr1	117,818,026	117,827,840	9,814	Loss
DEL00007211	chr1	117,907,521	117,908,529	1,008	Loss
DEL00007215	chr1	117,927,562	117,930,543	2,981	Loss
DEL00007219	chr1	118,010,835	118,011,527	692	Loss
DEL00007220	chr1	118,031,900	118,031,964	64	Loss
DEL00007221	chr1	118,048,886	118,049,804	918	Loss
DEL00007222	chr1	118,062,275	118,063,732	1,457	Loss
DEL00007225	chr1	118,118,379	118,118,631	252	Loss
DEL00007236	chr1	118,343,361	118,344,283	922	Loss
DEL00007238	chr1	118,373,196	118,374,447	1,251	Loss
DEL00007241	chr1	118,417,191	118,433,800	16,609	Loss
DEL00007242	chr1	118,465,307	118,465,368	61	Loss
DEL00007244	chr1	118,539,385	118,539,942	557	Loss
DUP00007248	chr1	118,575,919	118,578,742	2,823	Mixed
DEL00007252	chr1	118,606,582	118,606,816	234	Loss
DEL00007253	chr1	118,718,417	118,718,519	102	Loss
DUP00007255	chr1	118,739,526	118,739,606	80	Gain
DEL00007261	chr1	118,930,717	118,930,862	145	Loss
DEL00007262	chr1	118,950,288	118,950,728	440	Loss
DEL00007268	chr1	119,129,849	119,130,096	247	Loss
DEL00007269	chr1	119,170,062	119,170,155	93	Loss
DEL00007271	chr1	119,174,731	119,175,128	397	Loss
DEL00007274	chr1	119,219,227	119,219,791	564	Loss
DEL00007280	chr1	119,262,112	119,262,338	226	Loss
DEL00007281	chr1	119,270,184	119,270,287	103	Loss
DEL00007282	chr1	119,278,050	119,280,930	2,880	Loss
DEL00007284	chr1	119,290,522	119,294,086	3,564	Loss
DEL00007289	chr1	119,306,450	119,307,018	568	Loss
DEL00007299	chr1	119,481,596	119,481,653	57	Loss
DEL00007302	chr1	119,601,548	119,602,009	461	Loss
DEL00007303	chr1	119,605,988	119,606,367	379	Loss
DEL00007304	chr1	119,609,204	119,609,299	95	Loss
DEL00007306	chr1	119,643,147	119,643,556	409	Loss
DEL00007309	chr1	119,912,544	119,912,785	241	Loss
DUP00007314	chr1	120,046,530	120,046,595	65	Gain
DEL00007315	chr1	120,086,763	120,086,922	159	Loss
DEL00007318	chr1	120,223,456	120,223,511	55	Loss
DEL00007320	chr1	120,243,980	120,244,084	104	Loss
DEL00007322	chr1	120,256,697	120,257,013	316	Loss
DEL00007328	chr1	120,515,291	120,516,213	922	Loss
DEL00007329	chr1	120,521,745	120,522,818	1,073	Loss
DEL00007332	chr1	120,552,303	120,552,542	239	Loss
DEL00007339	chr1	120,768,635	120,768,969	334	Loss
DEL00007350	chr1	120,909,403	120,909,462	59	Loss
DEL00007351	chr1	120,942,201	120,942,330	129	Loss
DEL00007361	chr1	121,006,848	121,007,440	592	Loss

DEL00007368	chr1	121,068,146	121,068,201	55	Loss
DEL00007369	chr1	121,129,171	121,129,228	57	Loss
DEL00007373	chr1	121,166,480	121,166,877	397	Loss
DEL00007378	chr1	121,194,944	121,195,144	200	Loss
DEL00007380	chr1	121,232,109	121,232,749	640	Loss
DEL00007385	chr1	121,275,147	121,275,318	171	Loss
DEL00007387	chr1	121,307,022	121,307,169	147	Loss
DEL00007389	chr1	121,328,593	121,330,821	2,228	Loss
DEL00007391	chr1	121,373,835	121,374,142	307	Loss
DEL00007400	chr1	121,501,998	121,503,543	1,545	Loss
DEL00007402	chr1	121,538,344	121,538,724	380	Loss
DEL00007403	chr1	121,560,841	121,560,896	55	Loss
DEL00007407	chr1	121,671,391	121,671,450	59	Loss
DEL00007418	chr1	121,795,712	121,795,800	88	Loss
DEL00007431	chr1	121,832,640	121,832,764	124	Loss
DEL00007432	chr1	121,835,033	121,835,323	290	Loss
DEL00007439	chr1	121,924,150	121,924,326	176	Loss
DEL00007446	chr1	122,071,568	122,071,663	95	Loss
DEL00007448	chr1	122,195,120	122,195,210	90	Loss
DEL00007449	chr1	122,282,983	122,284,284	1,301	Loss
DEL00007459	chr1	122,381,240	122,382,464	1,224	Loss
DEL00007463	chr1	122,407,367	122,407,829	462	Loss
DEL00007465	chr1	122,456,459	122,456,567	108	Loss
DEL00007483	chr1	122,608,501	122,608,576	75	Loss
DEL00007491	chr1	122,830,243	122,830,303	60	Loss
DEL00007493	chr1	122,857,515	122,858,217	702	Loss
DEL00007495	chr1	122,896,044	122,896,659	615	Loss
DEL00007505	chr1	123,111,409	123,111,887	478	Loss
DEL00007507	chr1	123,114,624	123,115,993	1,369	Loss
DEL00007516	chr1	123,335,740	123,337,853	2,113	Loss
DEL00007521	chr1	123,472,611	123,473,018	407	Loss
DEL00007525	chr1	123,533,594	123,534,481	887	Loss
DEL00007535	chr1	123,692,933	123,692,992	59	Loss
DEL00007537	chr1	123,746,620	123,746,704	84	Loss
DEL00007538	chr1	123,805,543	123,805,731	188	Loss
DEL00007551	chr1	123,982,664	123,982,744	80	Loss
DEL00007554	chr1	124,018,085	124,018,163	78	Loss
DEL00007555	chr1	124,026,756	124,026,818	62	Loss
DEL00007560	chr1	124,117,270	124,117,323	53	Loss
DEL00007562	chr1	124,156,459	124,156,653	194	Loss
DEL00007570	chr1	124,338,740	124,339,029	289	Loss
DEL00007575	chr1	124,399,591	124,399,722	131	Loss
DEL00007587	chr1	124,507,813	124,507,918	105	Loss
DEL00007589	chr1	124,517,738	124,518,044	306	Loss
DEL00007592	chr1	124,572,748	124,572,896	148	Loss
DEL00007596	chr1	124,636,172	124,636,922	750	Loss
DEL00007598	chr1	124,660,083	124,660,310	227	Loss
DEL00007599	chr1	124,669,710	124,670,605	895	Loss
DEL00007604	chr1	124,712,372	124,712,441	69	Loss

DEL00007606	chr1	124,742,515	124,742,729	214	Loss
DEL00007607	chr1	124,762,735	124,762,803	68	Loss
DEL00007609	chr1	124,830,709	124,831,345	636	Loss
DEL00007616	chr1	124,912,305	124,912,364	59	Loss
DEL00007617	chr1	124,924,032	124,924,202	170	Loss
DEL00007618	chr1	124,929,071	124,929,772	701	Loss
DEL00007633	chr1	125,144,916	125,145,130	214	Loss
DEL00007637	chr1	125,175,887	125,175,983	96	Loss
DEL00007652	chr1	125,290,190	125,304,627	14,437	Loss
DEL00007656	chr1	125,359,500	125,359,953	453	Loss
DEL00007660	chr1	125,493,877	125,494,016	139	Loss
DEL00007662	chr1	125,515,599	125,515,655	56	Loss
DEL00007667	chr1	125,640,452	125,640,605	153	Loss
DEL00007668	chr1	125,643,114	125,643,193	79	Loss
DEL00007669	chr1	125,643,309	125,645,941	2,632	Loss
DEL00007673	chr1	125,703,011	125,703,337	326	Loss
DEL00007674	chr1	125,761,995	125,762,115	120	Loss
DEL00007689	chr1	126,179,315	126,179,594	279	Loss
DEL00007690	chr1	126,271,210	126,271,271	61	Loss
DEL00007695	chr1	126,376,189	126,376,242	53	Loss
DEL00007696	chr1	126,424,195	126,424,293	98	Loss
DEL00007697	chr1	126,456,604	126,457,794	1,190	Loss
DEL00007698	chr1	126,462,806	126,462,913	107	Loss
DEL00007702	chr1	126,571,788	126,572,348	560	Loss
DEL00007710	chr1	126,706,693	126,706,753	60	Loss
DEL00007712	chr1	126,740,489	126,740,686	197	Loss
DEL00007713	chr1	126,741,120	126,741,627	507	Loss
DEL00007715	chr1	126,745,803	126,746,117	314	Loss
DEL00007716	chr1	126,800,332	126,800,393	61	Loss
DUP00007717	chr1	126,871,008	126,877,224	6,216	Mixed
DEL00007720	chr1	126,897,508	126,898,722	1,214	Loss
DEL00007721	chr1	126,901,112	126,901,216	104	Loss
DEL00007724	chr1	126,968,189	126,968,243	54	Loss
DEL00007727	chr1	127,049,935	127,050,751	816	Loss
DEL00007732	chr1	127,139,210	127,140,864	1,654	Loss
DEL00007734	chr1	127,208,232	127,208,307	75	Loss
DEL00007735	chr1	127,269,014	127,269,066	52	Loss
DEL00007736	chr1	127,303,028	127,304,091	1,063	Loss
DEL00007738	chr1	127,553,181	127,553,992	811	Loss
DEL00007740	chr1	127,601,519	127,601,579	60	Loss
DUP00007741	chr1	127,613,374	127,613,513	139	Gain
DEL00007742	chr1	127,613,671	127,615,134	1,463	Loss
DEL00007744	chr1	127,665,421	127,666,022	601	Loss
DEL00007745	chr1	127,688,626	127,688,718	92	Loss
DEL00007747	chr1	127,735,867	127,736,734	867	Loss
DEL00007749	chr1	127,772,228	127,772,293	65	Loss
DEL00007758	chr1	128,159,031	128,162,191	3,160	Loss
DEL00007759	chr1	128,195,185	128,195,243	58	Loss
DEL00007763	chr1	128,267,072	128,268,133	1,061	Loss

DEL00007765	chr1	128,357,781	128,358,098	317	Loss
DEL00007767	chr1	128,414,975	128,415,278	303	Loss
DEL00007768	chr1	128,415,542	128,415,605	63	Loss
DEL00007770	chr1	128,467,668	128,468,865	1,197	Loss
DEL00007778	chr1	128,675,796	128,675,939	143	Loss
DEL00007780	chr1	128,718,807	128,720,125	1,318	Loss
DEL00007781	chr1	128,747,103	128,747,175	72	Loss
DEL00007782	chr1	128,750,398	128,756,723	6,325	Loss
DEL00007784	chr1	128,775,955	128,777,566	1,611	Loss
DEL00007792	chr1	128,877,287	128,878,094	807	Loss
DEL00007794	chr1	128,910,066	128,910,269	203	Loss
DEL00007803	chr1	129,344,618	129,345,097	479	Loss
DUP00007805	chr1	129,347,079	129,347,216	137	Gain
DEL00007806	chr1	129,377,215	129,377,287	72	Loss
DEL00007808	chr1	129,412,332	129,413,119	787	Loss
DEL00007810	chr1	129,419,999	129,421,196	1,197	Loss
DEL00007815	chr1	129,500,884	129,500,952	68	Loss
DEL00007821	chr1	129,708,813	129,716,886	8,073	Loss
DEL00007822	chr1	129,720,442	129,720,520	78	Loss
DEL00007823	chr1	129,732,604	129,733,728	1,124	Loss
DUP00007829	chr1	129,849,095	129,849,186	91	Gain
DEL00007835	chr1	129,984,650	129,984,724	74	Loss
DEL00007837	chr1	130,098,220	130,098,281	61	Loss
DEL00007838	chr1	130,103,277	130,103,852	575	Loss
DEL00007841	chr1	130,154,820	130,157,162	2,342	Loss
DEL00007843	chr1	130,179,113	130,179,192	79	Loss
DEL00007848	chr1	130,405,542	130,405,617	75	Loss
DEL00007851	chr1	130,497,905	130,498,112	207	Loss
DEL00007855	chr1	130,547,195	130,547,843	648	Loss
DEL00007858	chr1	130,761,689	130,761,791	102	Loss
DEL00007859	chr1	130,794,019	130,794,556	537	Loss
DEL00007867	chr1	130,900,252	130,900,346	94	Loss
DEL00007868	chr1	130,910,265	130,910,375	110	Loss
DEL00007870	chr1	131,003,666	131,004,261	595	Loss
DEL00007874	chr1	131,084,482	131,084,543	61	Loss
DEL00007875	chr1	131,085,117	131,085,175	58	Loss
DEL00007878	chr1	131,137,065	131,137,133	68	Loss
DEL00007886	chr1	131,199,387	131,199,588	201	Loss
DEL00007889	chr1	131,238,980	131,239,062	82	Loss
DEL00007896	chr1	131,354,941	131,355,031	90	Loss
DEL00007897	chr1	131,367,692	131,367,823	131	Loss
DEL00007901	chr1	131,593,470	131,593,581	111	Loss
DEL00007902	chr1	131,715,834	131,715,902	68	Loss
DEL00007905	chr1	131,754,244	131,754,384	140	Loss
DEL00007907	chr1	131,835,470	131,836,223	753	Loss
DEL00007910	chr1	131,905,634	131,906,714	1,080	Loss
DEL00007914	chr1	131,999,617	131,999,672	55	Loss
DEL00007915	chr1	132,003,847	132,003,906	59	Loss
DEL00007917	chr1	132,031,649	132,031,957	308	Loss

DEL00007919	chr1	132,032,233	132,033,382	1,149	Loss
DEL00007921	chr1	132,061,144	132,061,266	122	Loss
DEL00007922	chr1	132,083,100	132,083,192	92	Loss
DEL00007930	chr1	132,210,905	132,211,690	785	Loss
DUP00007932	chr1	132,244,294	132,244,410	116	Gain
DEL00007941	chr1	132,430,386	132,430,454	68	Loss
DEL00007944	chr1	132,465,723	132,465,835	112	Loss
DEL00007947	chr1	132,483,689	132,483,759	70	Loss
DEL00007951	chr1	132,605,387	132,605,836	449	Loss
DEL00007955	chr1	132,678,780	132,680,081	1,301	Loss
DEL00007958	chr1	132,774,716	132,774,909	193	Loss
DEL00007960	chr1	132,819,550	132,819,794	244	Loss
DEL00007965	chr1	133,017,477	133,017,721	244	Loss
DEL00007967	chr1	133,074,225	133,074,737	512	Loss
DEL00007970	chr1	133,136,812	133,137,008	196	Loss
DEL00007976	chr1	133,505,494	133,505,597	103	Loss
DEL00007988	chr1	133,770,822	133,770,998	176	Loss
DEL00007990	chr1	133,789,622	133,790,186	564	Loss
DEL00007992	chr1	133,852,878	133,853,017	139	Loss
DEL00007998	chr1	134,003,345	134,003,429	84	Loss
DEL00008000	chr1	134,024,518	134,024,641	123	Loss
DEL00008003	chr1	134,051,406	134,051,731	325	Loss
DEL00008010	chr1	134,255,117	134,255,418	301	Loss
DEL00008011	chr1	134,262,611	134,263,034	423	Loss
DEL00008013	chr1	134,272,852	134,273,504	652	Loss
DEL00008021	chr1	134,383,066	134,383,767	701	Loss
DEL00008029	chr1	134,638,525	134,638,609	84	Loss
DEL00008030	chr1	134,640,608	134,640,848	240	Loss
DUP00008037	chr1	134,681,439	134,681,619	180	Gain
DEL00008040	chr1	134,698,267	134,698,649	382	Loss
DEL00008043	chr1	134,753,966	134,754,609	643	Loss
DEL00008049	chr1	134,885,597	134,886,049	452	Loss
DEL00008054	chr1	135,104,791	135,104,910	119	Loss
DEL00008057	chr1	135,226,541	135,226,632	91	Loss
DEL00008061	chr1	135,335,024	135,335,484	460	Loss
DEL00008062	chr1	135,337,077	135,337,159	82	Loss
DEL00008063	chr1	135,393,796	135,397,467	3,671	Loss
DEL00008064	chr1	135,398,373	135,398,825	452	Loss
DEL00008068	chr1	135,554,205	135,554,813	608	Loss
DEL00008070	chr1	135,571,607	135,571,713	106	Loss
DEL00008072	chr1	135,758,405	135,758,473	68	Loss
DEL00008076	chr1	135,879,968	135,880,020	52	Loss
DEL00008080	chr1	135,922,092	135,922,346	254	Loss
DEL00008083	chr1	135,950,433	135,950,815	382	Loss
DEL00008086	chr1	135,984,527	135,986,036	1,509	Loss
DEL00008089	chr1	136,034,284	136,034,724	440	Loss
DEL00008092	chr1	136,174,359	136,174,636	277	Loss
DEL00008093	chr1	136,190,296	136,190,387	91	Loss
DEL00008095	chr1	136,195,798	136,195,852	54	Loss

DEL00008096	chr1	136,221,299	136,221,361	62	Loss
DEL00008098	chr1	136,232,633	136,232,712	79	Loss
DEL00008100	chr1	136,252,680	136,252,743	63	Loss
DEL00008102	chr1	136,259,013	136,259,311	298	Loss
DEL00008103	chr1	136,288,205	136,289,829	1,624	Loss
DEL00008105	chr1	136,374,558	136,374,747	189	Loss
DEL00008108	chr1	136,422,183	136,422,350	167	Loss
DEL00008109	chr1	136,448,196	136,449,347	1,151	Loss
DEL00008114	chr1	136,614,961	136,615,021	60	Loss
DEL00008115	chr1	136,629,284	136,632,170	2,886	Loss
DEL00008118	chr1	136,647,185	136,647,242	57	Loss
DEL00008119	chr1	136,653,577	136,655,119	1,542	Loss
DEL00008120	chr1	136,665,253	136,665,318	65	Loss
DEL00008124	chr1	136,707,293	136,707,382	89	Loss
DEL00008128	chr1	136,772,133	136,772,433	300	Loss
DEL00008130	chr1	136,790,965	136,793,712	2,747	Loss
DEL00008141	chr1	136,995,366	136,995,585	219	Loss
DEL00008144	chr1	137,088,901	137,089,078	177	Loss
DEL00008157	chr1	137,118,050	137,154,550	36,500	Loss
DUP00008201	chr1	137,165,248	137,225,027	59,779	Gain
DEL00008241	chr1	137,280,058	137,282,660	2,602	Loss
DEL00008245	chr1	137,362,657	137,362,766	109	Loss
DEL00008249	chr1	137,519,659	137,520,394	735	Loss
DEL00008252	chr1	137,649,832	137,650,057	225	Loss
DEL00008254	chr1	137,734,530	137,734,738	208	Loss
DEL00008260	chr1	137,847,154	137,848,299	1,145	Loss
DEL00008263	chr1	137,969,529	137,969,943	414	Loss
DEL00008268	chr1	137,999,496	137,999,590	94	Loss
DEL00008270	chr1	138,018,735	138,018,807	72	Loss
DEL00008272	chr1	138,078,027	138,078,739	712	Loss
DEL00008275	chr1	138,178,085	138,178,169	84	Loss
DEL00008277	chr1	138,203,199	138,203,484	285	Loss
DEL00008281	chr1	138,246,384	138,246,619	235	Loss
DEL00008288	chr1	138,454,046	138,454,189	143	Loss
DEL00008292	chr1	138,572,372	138,572,566	194	Loss
DEL00008293	chr1	138,633,182	138,633,269	87	Loss
DEL00008304	chr1	138,858,349	138,858,680	331	Loss
DEL00008309	chr1	138,940,537	138,940,785	248	Loss
DEL00008310	chr1	138,960,353	138,960,430	77	Loss
DEL00008315	chr1	139,016,454	139,017,804	1,350	Loss
DEL00008317	chr1	139,027,065	139,027,164	99	Loss
DEL00008318	chr1	139,027,593	139,028,403	810	Loss
DEL00008327	chr1	139,288,912	139,289,010	98	Loss
DEL00008336	chr1	139,462,808	139,463,957	1,149	Loss
DEL00008340	chr1	139,655,541	139,656,403	862	Loss
DEL00008343	chr1	139,725,673	139,725,745	72	Loss
DEL00008347	chr1	139,809,272	139,809,585	313	Loss
DEL00008350	chr1	139,877,370	139,877,515	145	Loss
DEL00008353	chr1	139,887,612	139,887,694	82	Loss

DEL00008355	chr1	139,921,754	139,921,862	108	Loss
DEL00008358	chr1	139,995,184	139,995,373	189	Loss
DEL00008359	chr1	140,005,084	140,005,357	273	Loss
DEL00008360	chr1	140,011,227	140,011,465	238	Loss
DEL00008377	chr1	140,340,363	140,340,603	240	Loss
DEL00008379	chr1	140,407,058	140,407,605	547	Loss
DEL00008381	chr1	140,415,197	140,417,143	1,946	Loss
DEL00008383	chr1	140,423,215	140,423,536	321	Loss
DEL00008385	chr1	140,459,805	140,459,883	78	Loss
DEL00008478	chr1	141,288,062	141,288,347	285	Loss
DEL00008479	chr1	141,317,999	141,318,235	236	Loss
DEL00008481	chr1	141,390,635	141,391,753	1,118	Loss
DEL00008483	chr1	141,471,200	141,472,722	1,522	Loss
DEL00008484	chr1	141,514,369	141,514,700	331	Loss
DEL00008485	chr1	141,555,942	141,556,847	905	Loss
DEL00008486	chr1	141,571,327	141,571,458	131	Loss
DEL00008489	chr1	141,665,059	141,665,114	55	Loss
DEL00008492	chr1	141,688,357	141,688,431	74	Loss
DEL00008494	chr1	141,717,331	141,717,514	183	Loss
DEL00008496	chr1	141,730,966	141,732,027	1,061	Loss
DEL00008500	chr1	141,768,563	141,768,621	58	Loss
DEL00008506	chr1	141,831,102	141,831,153	51	Loss
DEL00008510	chr1	141,973,444	141,973,499	55	Loss
DEL00008519	chr1	142,252,072	142,253,266	1,194	Loss
DEL00008520	chr1	142,278,011	142,279,993	1,982	Loss
DEL00008521	chr1	142,297,747	142,298,300	553	Loss
DEL00008524	chr1	142,326,638	142,328,090	1,452	Loss
DEL00008527	chr1	142,339,135	142,340,055	920	Loss
DEL00008528	chr1	142,395,111	142,396,099	988	Loss
DEL00008532	chr1	142,438,911	142,440,023	1,112	Loss
DEL00008537	chr1	142,543,217	142,543,889	672	Loss
DEL00008539	chr1	142,563,932	142,564,178	246	Loss
DUP00008543	chr1	142,635,163	142,635,295	132	Gain
DEL00008546	chr1	142,669,682	142,670,093	411	Loss
DEL00008551	chr1	142,775,240	142,776,203	963	Loss
DEL00008553	chr1	142,777,409	142,777,515	106	Loss
DEL00008583	chr1	142,974,436	142,974,591	155	Loss
DEL00008585	chr1	143,040,242	143,040,304	62	Loss
DEL00008590	chr1	143,192,546	143,192,597	51	Loss
DEL00008591	chr1	143,198,175	143,198,784	609	Loss
DEL00008597	chr1	143,255,862	143,258,145	2,283	Loss
DEL00008598	chr1	143,313,336	143,314,975	1,639	Loss
DEL00008599	chr1	143,321,515	143,322,460	945	Loss
DEL00008608	chr1	143,442,752	143,443,706	954	Loss
DEL00008611	chr1	143,541,004	143,545,643	4,639	Loss
DEL00008613	chr1	143,592,669	143,594,839	2,170	Loss
DEL00008619	chr1	143,766,856	143,766,925	69	Loss
DEL00008620	chr1	143,807,500	143,807,726	226	Loss
DEL00008621	chr1	143,813,299	143,813,698	399	Loss

DEL00008629	chr1	143,994,106	143,994,278	172	Loss
DEL00008636	chr1	144,092,313	144,092,729	416	Loss
DEL00008638	chr1	144,116,291	144,116,734	443	Loss
DEL00008641	chr1	144,134,178	144,134,231	53	Loss
DEL00008642	chr1	144,158,147	144,158,935	788	Loss
DEL00008644	chr1	144,231,527	144,231,611	84	Loss
DEL00008650	chr1	144,376,886	144,377,038	152	Loss
DEL00008651	chr1	144,389,655	144,390,053	398	Loss
DEL00008655	chr1	144,495,352	144,495,882	530	Loss
DEL00008657	chr1	144,496,223	144,496,413	190	Loss
DEL00008660	chr1	144,527,361	144,527,746	385	Loss
DEL00008663	chr1	144,574,218	144,574,360	142	Loss
DEL00008664	chr1	144,619,844	144,619,980	136	Loss
DUP00008672	chr1	144,890,019	144,890,123	104	Gain
DEL00008675	chr1	144,953,584	144,955,319	1,735	Loss
DEL00008678	chr1	145,021,895	145,021,953	58	Loss
DEL00008680	chr1	145,027,681	145,027,822	141	Loss
DEL00008681	chr1	145,033,436	145,033,576	140	Loss
DEL00008683	chr1	145,097,347	145,097,827	480	Loss
DEL00008691	chr1	145,259,016	145,259,297	281	Loss
DEL00008696	chr1	145,317,630	145,317,741	111	Loss
DEL00008702	chr1	145,497,486	145,497,681	195	Loss
DEL00008710	chr1	145,573,572	145,573,623	51	Loss
DEL00008714	chr1	145,656,259	145,656,535	276	Loss
DEL00008718	chr1	145,735,688	145,735,741	53	Loss
DEL00008722	chr1	145,811,709	145,811,768	59	Loss
DEL00008726	chr1	145,887,167	145,887,319	152	Loss
DEL00008727	chr1	145,894,641	145,894,869	228	Loss
DEL00008729	chr1	145,946,372	145,946,974	602	Loss
DEL00008732	chr1	146,208,021	146,208,279	258	Loss
DEL00008734	chr1	146,225,121	146,225,641	520	Loss
DEL00008737	chr1	146,266,359	146,266,463	104	Loss
DEL00008738	chr1	146,305,985	146,306,348	363	Loss
DUP00008739	chr1	146,315,868	146,316,015	147	Gain
DEL00008741	chr1	146,427,591	146,427,745	154	Loss
DEL00008742	chr1	146,431,723	146,432,338	615	Loss
DEL00008747	chr1	146,542,610	146,545,261	2,651	Loss
DEL00008757	chr1	146,670,248	146,670,580	332	Loss
DEL00008765	chr1	146,724,696	146,724,769	73	Loss
DEL00008768	chr1	146,818,271	146,818,742	471	Loss
DEL00008771	chr1	146,819,747	146,820,101	354	Loss
DEL00008774	chr1	146,842,997	146,843,126	129	Loss
DEL00008776	chr1	146,885,146	146,885,594	448	Loss
DEL00008780	chr1	146,935,766	146,936,457	691	Loss
DEL00008787	chr1	147,001,195	147,001,571	376	Loss
DEL00008790	chr1	147,044,891	147,044,959	68	Loss
DEL00008791	chr1	147,109,360	147,109,491	131	Loss
DEL00008794	chr1	147,144,346	147,144,654	308	Loss
DEL00008797	chr1	147,288,351	147,288,423	72	Loss

DEL00008803	chr1	147,436,373	147,436,454	81	Loss
DEL00008813	chr1	147,588,895	147,589,070	175	Loss
DUP00008815	chr1	147,664,174	147,684,522	20,348	Gain
DEL00008816	chr1	147,699,167	147,706,699	7,532	Loss
DEL00008818	chr1	147,767,322	147,771,615	4,293	Loss
DEL00008822	chr1	147,850,648	147,851,138	490	Loss
DEL00008823	chr1	147,874,803	147,875,315	512	Loss
DEL00008831	chr1	147,989,689	147,990,691	1,002	Loss
DEL00008833	chr1	148,014,553	148,014,657	104	Loss
DEL00008834	chr1	148,059,434	148,059,867	433	Loss
DEL00008840	chr1	148,120,131	148,120,696	565	Loss
DEL00008841	chr1	148,150,069	148,150,151	82	Loss
DEL00008843	chr1	148,206,323	148,207,318	995	Loss
DEL00008850	chr1	148,273,118	148,274,047	929	Loss
DEL00008857	chr1	148,325,255	148,328,275	3,020	Loss
DEL00008858	chr1	148,366,174	148,366,862	688	Loss
DEL00008859	chr1	148,460,641	148,460,697	56	Loss
DEL00008860	chr1	148,471,994	148,472,261	267	Loss
DEL00008863	chr1	148,504,361	148,504,649	288	Loss
DEL00008866	chr1	148,511,128	148,511,377	249	Loss
DEL00008868	chr1	148,541,243	148,541,765	522	Loss
DEL00008871	chr1	148,553,619	148,553,939	320	Loss
DEL00008873	chr1	148,578,069	148,579,407	1,338	Loss
DEL00008876	chr1	148,627,311	148,627,536	225	Loss
DEL00008878	chr1	148,646,751	148,647,121	370	Loss
DEL00008879	chr1	148,673,487	148,673,943	456	Loss
DEL00008882	chr1	148,675,098	148,675,758	660	Loss
DEL00008885	chr1	148,767,298	148,767,920	622	Loss
DEL00008887	chr1	148,838,270	148,838,400	130	Loss
DEL00008889	chr1	148,918,822	148,919,131	309	Loss
DEL00008890	chr1	148,927,271	148,927,333	62	Loss
DEL00008891	chr1	148,927,769	148,928,063	294	Loss
DEL00008893	chr1	148,956,564	148,956,683	119	Loss
DEL00008895	chr1	148,993,237	148,993,427	190	Loss
DEL00008898	chr1	149,095,114	149,095,179	65	Loss
DEL00008901	chr1	149,238,045	149,238,179	134	Loss
DEL00008902	chr1	149,293,971	149,294,371	400	Loss
DEL00008903	chr1	149,311,452	149,311,588	136	Loss
DEL00008904	chr1	149,371,872	149,373,215	1,343	Loss
DEL00008910	chr1	149,443,504	149,443,648	144	Loss
DEL00008911	chr1	149,525,048	149,525,276	228	Loss
DEL00008912	chr1	149,525,335	149,525,469	134	Loss
DEL00008924	chr1	149,611,269	149,619,214	7,945	Loss
DEL00008926	chr1	149,654,314	149,654,504	190	Loss
DEL00008937	chr1	149,801,952	149,803,195	1,243	Loss
DEL00008939	chr1	149,886,001	149,890,653	4,652	Loss
DEL00008940	chr1	149,926,346	149,926,829	483	Loss
DEL00008941	chr1	149,926,957	149,927,305	348	Loss
DEL00008942	chr1	149,929,506	149,930,917	1,411	Loss

DEL00008948	chr1	150,050,928	150,050,984	56	Loss
DEL00008953	chr1	150,100,064	150,100,489	425	Loss
DEL00008957	chr1	150,124,581	150,128,446	3,865	Loss
DEL00008958	chr1	150,132,621	150,132,684	63	Loss
DEL00008961	chr1	150,163,505	150,163,572	67	Loss
DEL00008962	chr1	150,212,589	150,216,877	4,288	Loss
DEL00008965	chr1	150,279,415	150,279,955	540	Loss
DEL00008967	chr1	150,320,689	150,321,658	969	Loss
DEL00008968	chr1	150,334,611	150,334,669	58	Loss
DEL00008971	chr1	150,389,251	150,389,618	367	Loss
DEL00008975	chr1	150,491,551	150,491,718	167	Loss
DEL00008981	chr1	150,562,012	150,562,262	250	Loss
DEL00008983	chr1	150,634,057	150,645,898	11,841	Loss
DEL00008984	chr1	150,652,087	150,652,973	886	Loss
DEL00008999	chr1	150,909,617	150,916,308	6,691	Loss
DEL00009007	chr1	150,965,720	150,965,970	250	Loss
DEL00009010	chr1	150,996,238	150,996,801	563	Loss
DEL00009011	chr1	151,012,515	151,014,156	1,641	Loss
DEL00009014	chr1	151,046,231	151,048,547	2,316	Loss
DEL00009018	chr1	151,074,675	151,076,436	1,761	Loss
DEL00009020	chr1	151,101,233	151,102,938	1,705	Loss
DEL00009026	chr1	151,253,201	151,253,262	61	Loss
DEL00009027	chr1	151,299,234	151,299,417	183	Loss
DEL00009028	chr1	151,314,526	151,314,659	133	Loss
DEL00009031	chr1	151,364,896	151,366,565	1,669	Loss
DUP00009032	chr1	151,378,757	151,378,890	133	Gain
DEL00009036	chr1	151,424,865	151,425,018	153	Loss
DEL00009037	chr1	151,437,698	151,444,051	6,353	Loss
DEL00009042	chr1	151,461,741	151,462,055	314	Loss
DEL00009048	chr1	151,491,565	151,491,692	127	Loss
DEL00009056	chr1	151,576,083	151,577,368	1,285	Loss
DEL00009064	chr1	151,671,817	151,672,325	508	Loss
DEL00009066	chr1	151,677,989	151,678,045	56	Loss
DEL00009067	chr1	151,679,778	151,680,738	960	Loss
DEL00009070	chr1	151,701,241	151,702,194	953	Loss
DEL00009071	chr1	151,705,344	151,705,988	644	Loss
DEL00009074	chr1	151,740,546	151,741,649	1,103	Loss
DEL00009082	chr1	151,761,209	151,761,273	64	Loss
DEL00009083	chr1	151,770,088	151,770,298	210	Loss
DEL00009092	chr1	151,890,219	151,890,986	767	Loss
DEL00009094	chr1	151,913,203	151,913,263	60	Loss
DEL00009097	chr1	151,949,731	151,949,967	236	Loss
DEL00009098	chr1	151,964,679	151,964,775	96	Loss
DEL00009100	chr1	151,994,186	151,994,345	159	Loss
DEL00009102	chr1	152,011,799	152,011,878	79	Loss
DEL00009105	chr1	152,069,692	152,069,802	110	Loss
DEL00009110	chr1	152,255,233	152,255,287	54	Loss
DEL00009115	chr1	152,342,245	152,342,609	364	Loss
DEL00009118	chr1	152,360,483	152,361,103	620	Loss

DEL00009119	chr1	152,362,330	152,362,784	454	Loss
DEL00009120	chr1	152,391,990	152,393,487	1,497	Loss
DEL00009122	chr1	152,415,590	152,415,943	353	Loss
DEL00009123	chr1	152,426,019	152,426,326	307	Loss
DEL00009125	chr1	152,442,247	152,442,888	641	Loss
DEL00009127	chr1	152,443,859	152,446,024	2,165	Loss
DEL00009130	chr1	152,452,677	152,452,875	198	Loss
DEL00009133	chr1	152,467,121	152,467,594	473	Loss
DEL00009142	chr1	152,670,572	152,671,411	839	Loss
DEL00009159	chr1	152,784,060	152,784,406	346	Loss
DEL00009163	chr1	152,848,584	152,849,455	871	Loss
DEL00009167	chr1	152,874,554	152,874,683	129	Loss
DEL00009173	chr1	152,928,946	152,929,416	470	Loss
DEL00009174	chr1	152,948,401	152,948,502	101	Loss
DEL00009178	chr1	152,980,977	152,981,120	143	Loss
DEL00009183	chr1	152,997,376	152,999,374	1,998	Loss
DEL00009184	chr1	153,005,604	153,005,710	106	Loss
DEL00009187	chr1	153,017,964	153,020,075	2,111	Loss
DEL00009197	chr1	153,194,859	153,194,959	100	Loss
DEL00009198	chr1	153,198,126	153,199,587	1,461	Loss
DUP00009199	chr1	153,205,029	153,205,088	59	Gain
DEL00009200	chr1	153,209,190	153,209,439	249	Loss
DEL00009202	chr1	153,220,264	153,222,462	2,198	Loss
DUP00009212	chr1	153,299,949	153,300,059	110	Gain
DEL00009215	chr1	153,306,008	153,306,609	601	Loss
DUP00009216	chr1	153,320,766	153,332,011	11,245	Mixed
DEL00009235	chr1	153,555,481	153,560,320	4,839	Loss
DEL00009239	chr1	153,642,228	153,642,295	67	Loss
DEL00009240	chr1	153,644,425	153,644,517	92	Loss
DEL00009242	chr1	153,712,699	153,712,808	109	Loss
DEL00009247	chr1	153,902,307	153,903,704	1,397	Loss
DEL00009248	chr1	153,908,728	153,909,250	522	Loss
DEL00009251	chr1	153,923,071	153,923,365	294	Loss
DEL00009253	chr1	153,949,268	153,949,354	86	Loss
DEL00009273	chr1	154,176,605	154,176,776	171	Loss
DEL00009276	chr1	154,187,742	154,187,828	86	Loss
DEL00009283	chr1	154,206,976	154,207,566	590	Loss
DEL00009284	chr1	154,210,417	154,211,152	735	Loss
DEL00009287	chr1	154,265,290	154,265,566	276	Loss
DEL00009288	chr1	154,309,282	154,309,396	114	Loss
DEL00009298	chr1	154,489,113	154,489,375	262	Loss
DEL00009300	chr1	154,581,221	154,581,280	59	Loss
DEL00009306	chr1	154,640,977	154,641,087	110	Loss
DEL00009307	chr1	154,679,884	154,680,015	131	Loss
DEL00009308	chr1	154,682,903	154,683,038	135	Loss
DEL00009310	chr1	154,683,393	154,685,406	2,013	Loss
DEL00009322	chr1	154,801,742	154,801,814	72	Loss
DEL00009323	chr1	154,837,362	154,837,416	54	Loss
DEL00009325	chr1	154,861,251	154,861,471	220	Loss

DEL00009331	chr1	154,918,731	154,918,904	173	Loss
DEL00009332	chr1	154,957,051	154,960,014	2,963	Loss
DEL00009337	chr1	155,036,374	155,036,522	148	Loss
DEL00009344	chr1	155,080,681	155,080,745	64	Loss
DEL00009345	chr1	155,104,397	155,105,254	857	Loss
DEL00009348	chr1	155,162,105	155,163,377	1,272	Loss
DEL00009351	chr1	155,173,115	155,175,127	2,012	Loss
DEL00009353	chr1	155,207,427	155,210,440	3,013	Loss
DEL00009357	chr1	155,234,887	155,235,145	258	Loss
DEL00009359	chr1	155,257,405	155,257,476	71	Loss
DEL00009364	chr1	155,331,718	155,331,802	84	Loss
DEL00009365	chr1	155,379,019	155,379,081	62	Loss
DEL00009367	chr1	155,409,914	155,409,972	58	Loss
DEL00009387	chr1	155,454,884	155,456,040	1,156	Loss
DEL00009395	chr1	155,565,106	155,566,052	946	Loss
DUP00009406	chr1	155,724,784	155,736,333	11,549	Gain
DEL00009407	chr1	155,765,444	155,768,819	3,375	Loss
DEL00009414	chr1	155,814,542	155,814,604	62	Loss
DEL00009416	chr1	155,828,497	155,828,893	396	Loss
DEL00009417	chr1	155,846,802	155,848,670	1,868	Loss
DEL00009426	chr1	156,019,750	156,019,963	213	Loss
DEL00009431	chr1	156,075,882	156,076,503	621	Loss
DEL00009446	chr1	156,172,838	156,173,168	330	Loss
DEL00009452	chr1	156,316,337	156,316,508	171	Loss
DEL00009460	chr1	156,368,397	156,368,916	519	Loss
DEL00009462	chr1	156,371,417	156,372,240	823	Loss
DEL00009465	chr1	156,387,463	156,388,390	927	Loss
DEL00009467	chr1	156,391,600	156,391,655	55	Loss
DEL00009470	chr1	156,444,209	156,444,269	60	Loss
DEL00009483	chr1	156,489,555	156,489,646	91	Loss
DEL00009488	chr1	156,511,340	156,511,432	92	Loss
DEL00009492	chr1	156,555,921	156,555,997	76	Loss
DEL00009493	chr1	156,591,600	156,591,674	74	Loss
DEL00009496	chr1	156,620,477	156,621,156	679	Loss
DEL00009515	chr1	156,766,850	156,767,073	223	Loss
DEL00009519	chr1	156,797,594	156,803,944	6,350	Loss
DEL00009521	chr1	156,831,802	156,831,889	87	Loss
DEL00009522	chr1	156,846,591	156,846,776	185	Loss
DEL00009529	chr1	156,931,230	156,931,428	198	Loss
DEL00009540	chr1	157,076,843	157,076,933	90	Loss
DUP00009547	chr1	157,168,764	157,168,831	67	Gain
DEL00009552	chr1	157,218,241	157,218,297	56	Loss
DEL00009561	chr1	157,242,615	157,243,257	642	Loss
DEL00009568	chr1	157,261,690	157,261,813	123	Loss
DEL00009585	chr1	157,613,391	157,614,846	1,455	Loss
DEL00009588	chr1	157,631,946	157,636,611	4,665	Loss
DEL00009589	chr1	157,652,548	157,652,686	138	Loss
DEL00009593	chr1	157,745,558	157,876,776	131,218	Loss
DEL00009604	chr1	157,966,876	157,967,112	236	Loss

DEL00009609	chr1	158,018,615	158,018,668	53	Loss
DEL00009617	chr1	158,171,683	158,175,735	4,052	Loss
DEL00009621	chr1	158,195,276	158,195,981	705	Loss
DEL00009624	chr1	158,212,428	158,212,520	92	Loss
DEL00009625	chr1	158,247,733	158,247,965	232	Loss
DEL00009626	chr1	158,283,797	158,283,877	80	Loss
DEL00009628	chr1	158,310,289	158,311,920	1,631	Loss
DEL00009634	chr1	158,349,981	158,350,106	125	Loss
DEL00009638	chr1	158,396,655	158,397,158	503	Loss
DEL00009639	chr1	158,500,685	158,500,755	70	Loss
DEL00009642	chr1	158,600,520	158,602,754	2,234	Loss
DEL00009646	chr1	158,677,817	158,677,938	121	Loss
DEL00009650	chr1	158,704,770	158,705,696	926	Loss
DEL00009653	chr1	158,716,503	158,717,565	1,062	Loss
DEL00009659	chr1	158,777,513	158,777,564	51	Loss
DEL00009663	chr1	158,803,104	158,803,182	78	Loss
DEL00009664	chr1	158,803,608	158,803,704	96	Loss
DEL00009666	chr1	158,844,376	158,846,753	2,377	Loss
DEL00009668	chr1	158,853,745	158,854,385	640	Loss
DEL00009676	chr1	158,903,813	158,905,073	1,260	Loss
DEL00009677	chr1	158,917,778	158,917,964	186	Loss
DEL00009678	chr1	158,925,019	158,926,964	1,945	Loss
DEL00009688	chr1	158,971,631	158,971,728	97	Loss
DEL00009689	chr1	158,975,889	158,976,704	815	Loss
DEL00009702	chr1	159,033,037	159,033,105	68	Loss
DEL00009704	chr1	159,069,771	159,069,913	142	Loss
DEL00009717	chr1	159,243,911	159,245,372	1,461	Loss
DEL00009721	chr1	159,253,755	159,253,818	63	Loss
DEL00009733	chr1	159,360,436	159,360,581	145	Loss
DEL00009735	chr1	159,398,395	159,401,877	3,482	Loss
DEL00009748	chr1	159,547,269	159,550,432	3,163	Loss
DEL00009761	chr1	159,829,627	159,830,311	684	Loss
DEL00009773	chr1	159,954,642	159,955,388	746	Loss
DEL00009775	chr1	160,099,910	160,099,973	63	Loss
DEL00009777	chr1	160,191,101	160,191,986	885	Loss
DEL00009778	chr1	160,291,913	160,291,996	83	Loss
DEL00009781	chr1	160,377,492	160,377,552	60	Loss
DEL00009784	chr1	160,479,737	160,480,636	899	Loss
DEL00009794	chr1	160,668,608	160,669,527	919	Loss
DEL00009795	chr1	160,693,203	160,693,386	183	Loss
DEL00009797	chr1	160,722,999	160,723,055	56	Loss
DEL00009804	chr1	160,891,185	160,892,447	1,262	Loss
DEL00009806	chr1	160,929,668	160,930,061	393	Loss
DEL00009808	chr1	161,014,379	161,016,290	1,911	Loss
DEL00009816	chr1	161,123,476	161,123,892	416	Loss
DEL00009817	chr1	161,185,303	161,186,120	817	Loss
DEL00009818	chr1	161,189,268	161,189,445	177	Loss
DEL00009820	chr1	161,214,551	161,217,375	2,824	Loss
DEL00009822	chr1	161,217,554	161,218,279	725	Loss

DEL00009823	chr1	161,241,439	161,241,724	285	Loss
DEL00009833	chr1	161,271,523	161,272,420	897	Loss
DEL00009834	chr1	161,302,472	161,303,055	583	Loss
DEL00009836	chr1	161,317,299	161,317,762	463	Loss
DEL00009839	chr1	161,376,038	161,376,459	421	Loss
DEL00009842	chr1	161,424,448	161,425,368	920	Loss
DEL00009843	chr1	161,428,826	161,433,480	4,654	Loss
DEL00009848	chr1	161,502,445	161,502,738	293	Loss
DUP00009849	chr1	161,514,924	161,514,987	63	Gain
DUP00009850	chr1	161,583,863	161,592,050	8,187	Gain
DEL00009862	chr1	161,912,705	161,912,757	52	Loss
DEL00009869	chr1	162,214,453	162,215,097	644	Loss
DEL00009872	chr1	162,301,633	162,302,370	737	Loss
DEL00009876	chr1	162,378,122	162,378,183	61	Loss
DEL00009877	chr1	162,398,701	162,399,544	843	Loss
DEL00009880	chr1	162,430,246	162,430,699	453	Loss
DEL00009881	chr1	162,449,808	162,449,864	56	Loss
DEL00009887	chr1	162,507,516	162,507,673	157	Loss
DEL00009888	chr1	162,550,118	162,550,614	496	Loss
DEL00009890	chr1	162,590,621	162,591,015	394	Loss
DEL00009895	chr1	162,622,490	162,629,420	6,930	Loss
DEL00009897	chr1	162,668,995	162,669,609	614	Loss
DEL00009898	chr1	162,671,323	162,671,669	346	Loss
DEL00009903	chr1	162,742,068	162,742,193	125	Loss
DEL00009906	chr1	162,777,233	162,777,301	68	Loss
DEL00009908	chr1	162,800,683	162,801,221	538	Loss
DEL00009909	chr1	162,807,489	162,807,960	471	Loss
DEL00009918	chr1	162,919,205	162,919,278	73	Loss
DEL00009924	chr1	162,993,676	162,994,564	888	Loss
DEL00009925	chr1	162,994,866	162,995,220	354	Loss
DEL00009927	chr1	163,015,694	163,017,201	1,507	Loss
DEL00009933	chr1	163,051,671	163,052,843	1,172	Loss
DEL00009935	chr1	163,136,254	163,136,715	461	Loss
DEL00009942	chr1	163,192,537	163,192,609	72	Loss
DEL00009951	chr1	163,396,122	163,396,646	524	Loss
DEL00009958	chr1	163,677,047	163,677,881	834	Loss
DEL00009966	chr1	163,729,399	163,729,685	286	Loss
DEL00009968	chr1	163,742,390	163,742,471	81	Loss
DEL00009969	chr1	163,812,963	163,814,136	1,173	Loss
DEL00009973	chr1	163,849,613	163,850,500	887	Loss
DEL00009974	chr1	163,864,040	163,865,216	1,176	Loss
DEL00009975	chr1	163,879,848	163,880,494	646	Loss
DUP00009976	chr1	163,907,024	163,909,132	2,108	Gain
DEL00009977	chr1	163,923,294	163,923,356	62	Loss
DEL00009978	chr1	163,964,099	163,964,658	559	Loss
DEL00009980	chr1	163,997,473	163,998,516	1,043	Loss
DEL00009981	chr1	164,007,154	164,007,587	433	Loss
DEL00009983	chr1	164,026,892	164,027,227	335	Loss
DEL00009984	chr1	164,049,406	164,049,537	131	Loss

DEL00010000	chr1	164,113,580	164,114,033	453	Loss
DEL00010005	chr1	164,244,402	164,244,648	246	Loss
DEL00010008	chr1	164,271,113	164,271,593	480	Loss
DEL00010010	chr1	164,353,803	164,354,297	494	Loss
DUP00010014	chr1	164,500,228	164,500,341	113	Gain
DEL00010015	chr1	164,574,962	164,575,182	220	Loss
DEL00010019	chr1	164,648,484	164,649,140	656	Loss
DEL00010021	chr1	164,778,704	164,778,997	293	Loss
DEL00010022	chr1	164,891,570	164,891,983	413	Loss
DEL00010027	chr1	164,931,205	164,938,014	6,809	Loss
DEL00010034	chr1	165,093,517	165,099,726	6,209	Loss
DEL00010035	chr1	165,107,016	165,124,939	17,923	Loss
DEL00010040	chr1	165,129,026	165,129,249	223	Loss
DEL00010053	chr1	165,292,395	165,296,615	4,220	Loss
DEL00010054	chr1	165,365,475	165,366,652	1,177	Loss
DEL00010057	chr1	165,471,358	165,472,915	1,557	Loss
DEL00010066	chr1	165,634,575	165,634,697	122	Loss
DUP00010067	chr1	165,671,101	165,671,238	137	Gain
DEL00010073	chr1	165,836,851	165,837,316	465	Loss
DEL00010075	chr1	165,895,955	165,896,012	57	Loss
DEL00010086	chr1	166,084,083	166,084,143	60	Loss
DEL00010087	chr1	166,098,138	166,098,196	58	Loss
DEL00010093	chr1	166,215,779	166,215,840	61	Loss
DEL00010094	chr1	166,262,097	166,262,202	105	Loss
DEL00010096	chr1	166,299,599	166,300,126	527	Loss
DEL00010100	chr1	166,426,526	166,426,640	114	Loss
DEL00010106	chr1	166,592,248	166,592,317	69	Loss
DEL00010111	chr1	166,665,451	166,665,618	167	Loss
DEL00010117	chr1	166,828,956	166,829,623	667	Loss
DEL00010120	chr1	166,927,390	166,927,998	608	Loss
DEL00010122	chr1	166,955,961	166,956,096	135	Loss
DEL00010124	chr1	167,012,272	167,012,323	51	Loss
DUP00010126	chr1	167,024,257	167,024,356	99	Gain
DEL00010133	chr1	167,188,551	167,189,057	506	Loss
DEL00010135	chr1	167,375,876	167,375,997	121	Loss
DEL00010138	chr1	167,513,240	167,513,718	478	Loss
DEL00010139	chr1	167,553,182	167,553,433	251	Loss
DEL00010140	chr1	167,565,065	167,565,158	93	Loss
DEL00010150	chr1	167,859,897	167,860,169	272	Loss
DEL00010156	chr1	168,014,743	168,014,795	52	Loss
DEL00010164	chr1	168,181,803	168,182,006	203	Loss
DEL00010165	chr1	168,198,068	168,198,284	216	Loss
DEL00010166	chr1	168,198,500	168,198,700	200	Loss
DEL00010179	chr1	168,364,520	168,364,604	84	Loss
DEL00010181	chr1	168,420,970	168,421,182	212	Loss
DEL00010182	chr1	168,442,953	168,443,447	494	Loss
DEL00010189	chr1	168,540,624	168,541,005	381	Loss
DEL00010192	chr1	168,581,057	168,581,121	64	Loss
DEL00010194	chr1	168,805,714	168,806,157	443	Loss

DEL00010198	chr1	168,873,004	168,873,349	345	Loss
DEL00010205	chr1	168,996,457	168,996,665	208	Loss
DEL00010208	chr1	169,033,303	169,033,957	654	Loss
DEL00010212	chr1	169,202,485	169,202,822	337	Loss
DEL00010217	chr1	169,246,606	169,246,970	364	Loss
DEL00010222	chr1	169,358,250	169,358,930	680	Loss
DEL00010226	chr1	169,375,950	169,376,274	324	Loss
DEL00010227	chr1	169,392,824	169,392,883	59	Loss
DEL00010239	chr1	169,536,867	169,536,923	56	Loss
DEL00010241	chr1	169,561,407	169,561,743	336	Loss
DEL00010243	chr1	169,593,430	169,593,741	311	Loss
DEL00010244	chr1	169,608,969	169,609,062	93	Loss
DEL00010250	chr1	169,668,761	169,669,432	671	Loss
DEL00010254	chr1	169,746,599	169,747,045	446	Loss
DEL00010260	chr1	169,862,052	169,862,353	301	Loss
DEL00010261	chr1	169,867,310	169,867,379	69	Loss
DEL00010266	chr1	169,931,656	169,931,770	114	Loss
DUP00010267	chr1	169,940,114	169,940,213	99	Gain
DEL00010268	chr1	169,955,045	169,955,603	558	Loss
DEL00010271	chr1	169,968,910	169,969,019	109	Loss
DEL00010277	chr1	170,139,868	170,140,106	238	Loss
DEL00010281	chr1	170,190,755	170,190,853	98	Loss
DEL00010289	chr1	170,292,507	170,292,562	55	Loss
DEL00010293	chr1	170,368,202	170,368,297	95	Loss
DEL00010297	chr1	170,484,904	170,485,091	187	Loss
DEL00010299	chr1	170,512,054	170,512,237	183	Loss
DEL00010303	chr1	170,663,864	170,664,848	984	Loss
DEL00010305	chr1	170,745,490	170,745,676	186	Loss
DEL00010318	chr1	170,780,248	170,780,380	132	Loss
DEL00010340	chr1	171,112,964	171,113,465	501	Loss
DEL00010341	chr1	171,150,282	171,150,343	61	Loss
DEL00010344	chr1	171,166,645	171,166,869	224	Loss
DEL00010350	chr1	171,214,154	171,214,373	219	Loss
DEL00010357	chr1	171,273,863	171,274,434	571	Loss
DEL00010359	chr1	171,293,248	171,293,304	56	Loss
DEL00010367	chr1	171,343,263	171,343,440	177	Loss
DEL00010368	chr1	171,357,307	171,357,359	52	Loss
DEL00010369	chr1	171,359,067	171,359,191	124	Loss
DEL00010378	chr1	171,454,954	171,455,158	204	Loss
DEL00010385	chr1	171,638,849	171,638,963	114	Loss
DEL00010388	chr1	171,734,142	171,734,358	216	Loss
DEL00010390	chr1	171,763,896	171,763,986	90	Loss
DEL00010391	chr1	171,802,020	171,802,077	57	Loss
DEL00010398	chr1	171,878,093	171,878,284	191	Loss
DEL00010400	chr1	171,930,707	171,930,865	158	Loss
DEL00010410	chr1	172,065,120	172,065,266	146	Loss
DEL00010417	chr1	172,215,880	172,215,971	91	Loss
DEL00010419	chr1	172,227,926	172,228,119	193	Loss
DEL00010420	chr1	172,258,676	172,258,816	140	Loss

DEL00010421	chr1	172,268,060	172,271,864	3,804	Loss
DEL00010427	chr1	172,393,059	172,393,110	51	Loss
DEL00010432	chr1	172,442,121	172,443,362	1,241	Loss
DEL00010436	chr1	172,492,571	172,493,009	438	Loss
DEL00010437	chr1	172,510,352	172,511,085	733	Loss
DEL00010444	chr1	172,619,528	172,620,089	561	Loss
DEL00010449	chr1	172,663,423	172,664,436	1,013	Loss
DEL00010451	chr1	172,718,990	172,719,089	99	Loss
DEL00010452	chr1	172,761,405	172,761,460	55	Loss
DEL00010462	chr1	172,963,799	172,965,419	1,620	Loss
DEL00010463	chr1	172,975,613	172,975,675	62	Loss
DEL00010464	chr1	172,981,450	172,982,217	767	Loss
DEL00010465	chr1	173,005,455	173,007,082	1,627	Loss
DEL00010467	chr1	173,033,879	173,033,974	95	Loss
DEL00010472	chr1	173,058,065	173,058,285	220	Loss
DEL00010473	chr1	173,062,843	173,063,407	564	Loss
DEL00010476	chr1	173,085,743	173,089,832	4,089	Loss
DEL00010477	chr1	173,093,737	173,093,867	130	Loss
DEL00010478	chr1	173,102,489	173,102,646	157	Loss
DEL00010483	chr1	173,169,282	173,170,817	1,535	Loss
DEL00010484	chr1	173,170,826	173,171,643	817	Loss
DEL00010485	chr1	173,184,247	173,185,048	801	Loss
DEL00010486	chr1	173,220,347	173,220,551	204	Loss
DEL00010493	chr1	173,413,188	173,413,620	432	Loss
DEL00010494	chr1	173,484,583	173,484,663	80	Loss
DEL00010499	chr1	173,617,573	173,619,933	2,360	Loss
DEL00010501	chr1	173,641,875	173,642,113	238	Loss
DEL00010503	chr1	173,658,258	173,658,344	86	Loss
DEL00010505	chr1	173,751,049	173,751,129	80	Loss
DEL00010506	chr1	173,761,309	173,763,393	2,084	Loss
DEL00010508	chr1	173,811,622	173,811,711	89	Loss
DUP00010510	chr1	173,887,761	173,894,588	6,827	Gain
DEL00010511	chr1	173,905,280	173,905,333	53	Loss
DEL00010516	chr1	174,061,810	174,061,865	55	Loss
DEL00010518	chr1	174,152,497	174,152,575	78	Loss
DEL00010520	chr1	174,159,540	174,159,600	60	Loss
DEL00010522	chr1	174,192,491	174,192,588	97	Loss
DEL00010523	chr1	174,241,770	174,242,474	704	Loss
DEL00010527	chr1	174,316,134	174,316,267	133	Loss
DEL00010532	chr1	174,379,690	174,380,595	905	Loss
DEL00010534	chr1	174,403,951	174,404,009	58	Loss
DEL00010537	chr1	174,496,689	174,496,752	63	Loss
DEL00010542	chr1	174,643,520	174,643,630	110	Loss
DEL00010545	chr1	174,690,851	174,690,948	97	Loss
DEL00010546	chr1	174,708,350	174,708,670	320	Loss
DEL00010551	chr1	174,759,526	174,759,589	63	Loss
DEL00010553	chr1	174,817,963	174,818,128	165	Loss
DEL00010556	chr1	174,846,299	174,846,353	54	Loss
DEL00010558	chr1	174,854,694	174,855,325	631	Loss

DEL00010559	chr1	174,855,786	174,855,848	62	Loss
DEL00010565	chr1	175,047,957	175,048,658	701	Loss
DEL00010566	chr1	175,097,834	175,098,511	677	Loss
DEL00010575	chr1	175,161,169	175,161,263	94	Loss
DEL00010576	chr1	175,179,384	175,179,435	51	Loss
DEL00010577	chr1	175,182,139	175,182,281	142	Loss
DEL00010579	chr1	175,205,451	175,205,832	381	Loss
DEL00010581	chr1	175,280,036	175,280,598	562	Loss
DEL00010585	chr1	175,330,902	175,330,997	95	Loss
DEL00010590	chr1	175,449,911	175,449,971	60	Loss
DEL00010593	chr1	175,472,994	175,473,062	68	Loss
DEL00010604	chr1	175,650,967	175,651,095	128	Loss
DEL00010607	chr1	175,667,566	175,667,977	411	Loss
DEL00010613	chr1	175,683,859	175,684,043	184	Loss
DEL00010614	chr1	175,720,625	175,721,126	501	Loss
DEL00010615	chr1	175,743,086	175,743,158	72	Loss
DEL00010622	chr1	175,816,013	175,816,151	138	Loss
DEL00010631	chr1	175,930,249	175,930,390	141	Loss
DEL00010632	chr1	175,947,771	175,948,670	899	Loss
DEL00010641	chr1	176,349,072	176,349,317	245	Loss
DEL00010644	chr1	176,384,683	176,385,432	749	Loss
DEL00010649	chr1	176,427,607	176,427,818	211	Loss
DEL00010652	chr1	176,550,031	176,550,279	248	Loss
DEL00010659	chr1	176,620,834	176,620,903	69	Loss
DEL00010660	chr1	176,629,428	176,629,494	66	Loss
DEL00010663	chr1	176,649,837	176,650,051	214	Loss
DEL00010668	chr1	176,900,702	176,900,784	82	Loss
DEL00010670	chr1	176,928,846	176,928,911	65	Loss
DEL00010673	chr1	176,958,126	176,958,265	139	Loss
DEL00010680	chr1	177,035,502	177,035,790	288	Loss
DEL00010681	chr1	177,061,529	177,061,937	408	Loss
DEL00010697	chr1	177,258,382	177,258,438	56	Loss
DEL00010701	chr1	177,303,131	177,303,814	683	Loss
DEL00010704	chr1	177,352,310	177,352,405	95	Loss
DEL00010706	chr1	177,399,064	177,399,695	631	Loss
DEL00010717	chr1	177,511,367	177,512,087	720	Loss
DEL00010725	chr1	177,626,515	177,626,724	209	Loss
DEL00010728	chr1	177,655,968	177,656,450	482	Loss
DEL00010729	chr1	177,707,276	177,708,333	1,057	Loss
DEL00010730	chr1	177,848,575	177,848,630	55	Loss
DEL00010732	chr1	177,911,659	177,911,759	100	Loss
DEL00010734	chr1	177,935,109	177,935,164	55	Loss
DEL00010735	chr1	177,936,728	177,937,851	1,123	Loss
DEL00010737	chr1	178,009,456	178,009,613	157	Loss
DEL00010738	chr1	178,029,000	178,029,089	89	Loss
DEL00010739	chr1	178,033,540	178,033,595	55	Loss
DEL00010740	chr1	178,037,953	178,038,004	51	Loss
DEL00010741	chr1	178,047,805	178,049,986	2,181	Loss
DEL00010771	chr1	178,288,615	178,288,972	357	Loss

DEL00010775	chr1	178,387,556	178,387,674	118	Loss
DEL00010777	chr1	178,402,366	178,402,932	566	Loss
DEL00010778	chr1	178,422,023	178,422,312	289	Loss
DEL00010780	chr1	178,487,776	178,487,840	64	Loss
DEL00010782	chr1	178,504,476	178,504,559	83	Loss
DEL00010784	chr1	178,524,050	178,524,139	89	Loss
DEL00010786	chr1	178,560,676	178,561,478	802	Loss
DEL00010795	chr1	178,647,348	178,647,401	53	Loss
DEL00010805	chr1	178,745,419	178,746,065	646	Loss
DEL00010808	chr1	178,770,386	178,771,152	766	Loss
DEL00010809	chr1	178,782,753	178,782,818	65	Loss
DEL00010812	chr1	178,846,451	178,846,859	408	Loss
DEL00010815	chr1	178,881,384	178,881,494	110	Loss
DEL00010817	chr1	178,907,334	178,907,886	552	Loss
DEL00010825	chr1	179,038,633	179,038,772	139	Loss
DEL00010835	chr1	179,320,402	179,320,820	418	Loss
DEL00010836	chr1	179,353,411	179,354,999	1,588	Loss
DEL00010839	chr1	179,388,215	179,389,579	1,364	Loss
DEL00010848	chr1	179,667,894	179,668,130	236	Loss
DEL00010849	chr1	179,670,668	179,671,098	430	Loss
DEL00010856	chr1	179,719,139	179,719,288	149	Loss
DEL00010858	chr1	179,758,617	179,758,672	55	Loss
DEL00010862	chr1	179,789,877	179,790,837	960	Loss
DEL00010868	chr1	179,881,322	179,881,401	79	Loss
DEL00010870	chr1	179,920,716	179,920,790	74	Loss
DEL00010872	chr1	179,963,153	179,963,238	85	Loss
DEL00010875	chr1	179,990,560	179,991,117	557	Loss
DEL00010877	chr1	180,035,372	180,036,008	636	Loss
DEL00010884	chr1	180,170,252	180,170,755	503	Loss
DEL00010886	chr1	180,177,778	180,177,886	108	Loss
DEL00010888	chr1	180,201,592	180,202,086	494	Loss
DEL00010893	chr1	180,267,569	180,267,671	102	Loss
DEL00010899	chr1	180,556,167	180,556,251	84	Loss
DEL00010903	chr1	180,697,280	180,697,381	101	Loss
DEL00010904	chr1	180,731,990	180,732,080	90	Loss
DEL00010906	chr1	180,794,344	180,794,412	68	Loss
DUP00010910	chr1	180,845,501	180,864,403	18,902	Gain
DEL00010912	chr1	180,871,301	180,872,257	956	Loss
DEL00010925	chr1	181,079,406	181,079,465	59	Loss
DEL00010928	chr1	181,131,046	181,131,306	260	Loss
DEL00010936	chr1	181,315,626	181,319,019	3,393	Loss
DEL00010937	chr1	181,319,970	181,320,177	207	Loss
DEL00010939	chr1	181,357,561	181,358,134	573	Loss
DUP00010943	chr1	181,388,840	181,406,357	17,517	Gain
DEL00010944	chr1	181,411,753	181,411,937	184	Loss
DUP00010948	chr1	181,431,303	181,431,487	184	Gain
DEL00010949	chr1	181,457,264	181,457,318	54	Loss
DEL00010954	chr1	181,535,083	181,535,282	199	Loss
DEL00010955	chr1	181,609,504	181,609,594	90	Loss

DEL00010956	chr1	181,615,999	181,618,217	2,218	Loss
DEL00010963	chr1	181,789,482	181,789,759	277	Loss
DEL00010965	chr1	181,811,465	181,811,706	241	Loss
DEL00010966	chr1	181,821,174	181,821,270	96	Loss
DEL00010967	chr1	181,832,658	181,833,108	450	Loss
DEL00010973	chr1	181,896,505	181,897,035	530	Loss
DEL00010982	chr1	182,161,371	182,162,300	929	Loss
DEL00010983	chr1	182,193,586	182,193,651	65	Loss
DEL00010986	chr1	182,211,196	182,211,317	121	Loss
DEL00010987	chr1	182,219,235	182,220,457	1,222	Loss
DEL00010991	chr1	182,330,019	182,330,230	211	Loss
DEL00010992	chr1	182,335,149	182,335,656	507	Loss
DEL00011000	chr1	182,413,052	182,414,327	1,275	Loss
DEL00011005	chr1	182,554,732	182,555,132	400	Loss
DEL00011012	chr1	182,623,985	182,624,204	219	Loss
DEL00011013	chr1	182,644,316	182,644,596	280	Loss
DEL00011023	chr1	182,759,865	182,763,039	3,174	Loss
DEL00011025	chr1	182,796,249	182,796,636	387	Loss
DEL00011031	chr1	182,915,556	182,916,013	457	Loss
DEL00011035	chr1	183,054,384	183,054,476	92	Loss
DEL00011038	chr1	183,115,522	183,115,731	209	Loss
DEL00011039	chr1	183,116,173	183,116,264	91	Loss
DEL00011044	chr1	183,184,900	183,185,257	357	Loss
DEL00011053	chr1	183,308,797	183,308,862	65	Loss
DUP00011054	chr1	183,317,610	183,332,700	15,090	Gain
DEL00011061	chr1	183,447,374	183,447,640	266	Loss
DEL00011070	chr1	183,764,716	183,764,857	141	Loss
DEL00011071	chr1	183,784,969	183,785,430	461	Loss
DUP00011074	chr1	183,830,803	183,831,345	542	Gain
DEL00011078	chr1	183,874,923	183,875,012	89	Loss
DEL00011080	chr1	183,906,545	183,909,942	3,397	Loss
DEL00011083	chr1	183,957,789	183,958,180	391	Loss
DEL00011086	chr1	183,984,310	183,984,769	459	Loss
DEL00011090	chr1	184,086,740	184,086,839	99	Loss
DEL00011094	chr1	184,243,549	184,248,573	5,024	Loss
DEL00011098	chr1	184,367,881	184,368,254	373	Loss
DEL00011100	chr1	184,395,708	184,395,830	122	Loss
DEL00011101	chr1	184,420,885	184,423,944	3,059	Loss
DEL00011103	chr1	184,483,072	184,483,151	79	Loss
DEL00011104	chr1	184,510,761	184,513,191	2,430	Loss
DEL00011105	chr1	184,539,198	184,539,322	124	Loss
DEL00011106	chr1	184,551,504	184,552,242	738	Loss
DEL00011107	chr1	184,553,623	184,553,693	70	Loss
DEL00011108	chr1	184,578,789	184,578,980	191	Loss
DEL00011109	chr1	184,605,990	184,607,497	1,507	Loss
DEL00011116	chr1	184,631,189	184,631,932	743	Loss
DEL00011117	chr1	184,650,334	184,651,962	1,628	Loss
DEL00011119	chr1	184,672,249	184,672,420	171	Loss
DEL00011121	chr1	184,673,942	184,674,243	301	Loss

DEL00011122	chr1	184,748,897	184,749,208	311	Loss
DEL00011123	chr1	184,749,557	184,749,777	220	Loss
DEL00011124	chr1	184,760,954	184,761,208	254	Loss
DEL00011129	chr1	184,816,571	184,817,207	636	Loss
DUP00011130	chr1	184,823,659	184,837,413	13,754	Gain
DEL00011134	chr1	184,960,031	184,960,179	148	Loss
DEL00011135	chr1	184,988,138	184,988,901	763	Loss
DEL00011136	chr1	185,013,440	185,014,652	1,212	Loss
DEL00011144	chr1	185,205,557	185,205,892	335	Loss
DEL00011147	chr1	185,266,729	185,267,612	883	Loss
DEL00011148	chr1	185,272,158	185,273,212	1,054	Loss
DEL00011149	chr1	185,281,605	185,281,702	97	Loss
DEL00011159	chr1	185,350,825	185,351,028	203	Loss
DEL00011160	chr1	185,391,303	185,391,676	373	Loss
DEL00011163	chr1	185,408,696	185,408,943	247	Loss
DEL00011164	chr1	185,471,095	185,472,184	1,089	Loss
DEL00011165	chr1	185,492,316	185,492,463	147	Loss
DEL00011171	chr1	185,654,808	185,655,270	462	Loss
DEL00011181	chr1	185,853,245	185,853,328	83	Loss
DEL00011182	chr1	185,913,970	185,914,042	72	Loss
DEL00011183	chr1	185,960,891	185,961,001	110	Loss
DEL00011184	chr1	185,967,572	185,967,772	200	Loss
DEL00011186	chr1	186,058,444	186,058,658	214	Loss
DEL00011189	chr1	186,077,105	186,077,995	890	Loss
DEL00011191	chr1	186,088,962	186,089,060	98	Loss
DEL00011194	chr1	186,157,726	186,158,075	349	Loss
DEL00011199	chr1	186,248,524	186,248,704	180	Loss
DEL00011206	chr1	186,447,473	186,447,559	86	Loss
DEL00011209	chr1	186,491,437	186,495,427	3,990	Loss
DEL00011214	chr1	186,523,179	186,523,706	527	Loss
DEL00011217	chr1	186,602,510	186,602,583	73	Loss
DEL00011219	chr1	186,615,134	186,615,585	451	Loss
DEL00011225	chr1	186,756,348	186,756,615	267	Loss
DEL00011231	chr1	186,831,474	186,831,603	129	Loss
DEL00011232	chr1	186,832,221	186,832,332	111	Loss
DEL00011235	chr1	186,853,218	186,853,806	588	Loss
DEL00011236	chr1	186,864,579	186,864,955	376	Loss
DEL00011240	chr1	186,896,033	186,896,107	74	Loss
DEL00011241	chr1	186,905,051	186,905,104	53	Loss
DEL00011242	chr1	186,912,575	186,912,641	66	Loss
DEL00011243	chr1	186,923,715	186,924,602	887	Loss
DEL00011245	chr1	186,947,495	186,948,540	1,045	Loss
DEL00011255	chr1	187,135,113	187,135,361	248	Loss
DEL00011257	chr1	187,150,028	187,150,085	57	Loss
DEL00011259	chr1	187,177,775	187,178,100	325	Loss
DEL00011260	chr1	187,231,959	187,232,645	686	Loss
DEL00011264	chr1	187,384,913	187,385,032	119	Loss
DEL00011270	chr1	187,452,631	187,452,971	340	Loss
DEL00011279	chr1	187,486,699	187,486,837	138	Loss

DEL00011284	chr1	187,523,652	187,523,776	124	Loss
DEL00011288	chr1	187,581,135	187,582,187	1,052	Loss
DEL00011298	chr1	187,643,422	187,649,263	5,841	Loss
DEL00011299	chr1	187,722,318	187,722,461	143	Loss
DEL00011301	chr1	187,764,103	187,764,378	275	Loss
DEL00011304	chr1	187,778,533	187,778,908	375	Loss
DEL00011310	chr1	187,867,765	187,868,154	389	Loss
DEL00011314	chr1	187,973,961	187,975,021	1,060	Loss
DEL00011316	chr1	188,026,892	188,027,025	133	Loss
DEL00011346	chr1	188,065,132	188,065,553	421	Loss
DEL00011348	chr1	188,071,590	188,071,672	82	Loss
DUP00011351	chr1	188,153,178	188,153,368	190	Gain
DEL00011352	chr1	188,154,731	188,155,176	445	Loss
DEL00011357	chr1	188,180,841	188,180,892	51	Loss
DEL00011358	chr1	188,221,802	188,222,356	554	Loss
DEL00011362	chr1	188,263,704	188,265,963	2,259	Loss
DEL00011363	chr1	188,303,073	188,303,166	93	Loss
DEL00011367	chr1	188,334,117	188,334,356	239	Loss
DEL00011369	chr1	188,405,743	188,406,779	1,036	Loss
DEL00011372	chr1	188,456,703	188,456,785	82	Loss
DEL00011376	chr1	188,510,507	188,511,391	884	Loss
DEL00011377	chr1	188,533,978	188,534,620	642	Loss
DEL00011386	chr1	188,690,359	188,690,900	541	Loss
DEL00011388	chr1	188,691,734	188,692,277	543	Loss
DEL00011392	chr1	188,726,572	188,726,642	70	Loss
DEL00011398	chr1	188,853,768	188,853,944	176	Loss
DEL00011406	chr1	188,952,853	188,953,025	172	Loss
DEL00011408	chr1	188,988,475	188,988,585	110	Loss
DEL00011414	chr1	189,129,648	189,129,728	80	Loss
DEL00011418	chr1	189,245,024	189,245,178	154	Loss
DEL00011421	chr1	189,429,797	189,431,048	1,251	Loss
DEL00011424	chr1	189,541,809	189,546,803	4,994	Loss
DEL00011437	chr1	189,843,081	189,843,653	572	Loss
DEL00011438	chr1	189,868,980	189,869,700	720	Loss
DEL00011443	chr1	190,001,219	190,001,386	167	Loss
DEL00011446	chr1	190,138,251	190,138,311	60	Loss
DEL00011447	chr1	190,149,363	190,149,428	65	Loss
DEL00011450	chr1	190,213,830	190,213,909	79	Loss
DEL00011453	chr1	190,241,725	190,241,911	186	Loss
DEL00011455	chr1	190,278,028	190,278,990	962	Loss
DEL00011462	chr1	190,442,953	190,443,010	57	Loss
DEL00011468	chr1	190,462,410	190,462,584	174	Loss
DEL00011470	chr1	190,491,569	190,492,133	564	Loss
DEL00011472	chr1	190,494,068	190,494,231	163	Loss
DEL00011474	chr1	190,589,128	190,589,619	491	Loss
DEL00011476	chr1	190,595,277	190,595,398	121	Loss
DEL00011477	chr1	190,610,541	190,611,493	952	Loss
DEL00011483	chr1	190,863,941	190,864,176	235	Loss
DEL00011494	chr1	191,085,283	191,086,205	922	Loss

DEL00011497	chr1	191,129,118	191,129,195	77	Loss
DEL00011500	chr1	191,173,351	191,173,403	52	Loss
DEL00011502	chr1	191,227,064	191,227,254	190	Loss
DEL00011508	chr1	191,368,113	191,368,465	352	Loss
DEL00011509	chr1	191,401,366	191,402,042	676	Loss
DEL00011512	chr1	191,461,869	191,462,408	539	Loss
DEL00011514	chr1	191,468,293	191,468,345	52	Loss
DEL00011517	chr1	191,481,745	191,482,083	338	Loss
DEL00011520	chr1	191,536,762	191,536,834	72	Loss
DEL00011523	chr1	191,589,268	191,589,412	144	Loss
DEL00011526	chr1	191,802,024	191,802,844	820	Loss
DEL00011528	chr1	191,952,049	191,952,605	556	Loss
DEL00011532	chr1	191,965,793	191,966,849	1,056	Loss
DEL00011533	chr1	191,968,520	191,968,578	58	Loss
DEL00011534	chr1	191,968,871	191,968,931	60	Loss
DEL00011535	chr1	191,981,439	191,981,970	531	Loss
DEL00011540	chr1	192,053,650	192,053,861	211	Loss
DEL00011541	chr1	192,059,401	192,059,510	109	Loss
DEL00011542	chr1	192,073,512	192,075,578	2,066	Loss
DEL00011543	chr1	192,090,517	192,090,971	454	Loss
DEL00011544	chr1	192,101,274	192,101,346	72	Loss
DEL00011545	chr1	192,132,575	192,132,631	56	Loss
DEL00011546	chr1	192,167,405	192,167,473	68	Loss
DEL00011547	chr1	192,219,790	192,227,683	7,893	Loss
DEL00011554	chr1	192,392,603	192,393,368	765	Loss
DEL00011555	chr1	192,406,415	192,406,468	53	Loss
DEL00011556	chr1	192,408,802	192,410,191	1,389	Loss
DEL00011561	chr1	192,501,850	192,502,142	292	Loss
DEL00011569	chr1	192,620,947	192,621,389	442	Loss
DEL00011572	chr1	192,633,869	192,634,464	595	Loss
DEL00011577	chr1	192,691,156	192,691,236	80	Loss
DEL00011587	chr1	192,851,692	192,852,635	943	Loss
DEL00011591	chr1	193,069,533	193,079,501	9,968	Loss
DEL00011593	chr1	193,183,991	193,184,076	85	Loss
DEL00011596	chr1	193,266,733	193,266,874	141	Loss
DEL00011598	chr1	193,394,257	193,394,371	114	Loss
DEL00011616	chr1	193,512,122	193,512,221	99	Loss
DEL00011622	chr1	193,585,708	194,027,066	441,358	Mixed
DEL00011650	chr1	194,096,535	194,098,381	1,846	Loss
DEL00011651	chr1	194,133,962	194,134,482	520	Loss
DEL00011652	chr1	194,165,971	194,166,849	878	Loss
DEL00011655	chr1	194,242,836	194,242,961	125	Loss
DEL00011660	chr1	194,252,796	194,253,228	432	Loss
DEL00011663	chr1	194,291,453	194,291,568	115	Loss
DEL00011669	chr1	194,399,020	194,400,197	1,177	Loss
DEL00011670	chr1	194,449,845	194,449,918	73	Loss
DEL00011674	chr1	194,516,007	194,516,495	488	Loss
DEL00011676	chr1	194,543,141	194,543,196	55	Loss
DEL00011679	chr1	194,602,722	194,602,786	64	Loss

DEL00011680	chr1	194,622,462	194,622,594	132	Loss
DEL00011684	chr1	194,690,922	194,691,070	148	Loss
DEL00011685	chr1	194,715,777	194,715,837	60	Loss
DEL00011690	chr1	194,748,478	194,748,582	104	Loss
DEL00011693	chr1	194,877,702	194,877,757	55	Loss
DEL00011696	chr1	194,932,195	194,932,377	182	Loss
DEL00011698	chr1	194,932,766	194,932,973	207	Loss
DEL00011713	chr1	195,161,734	195,161,820	86	Loss
DEL00011725	chr1	195,332,051	195,332,535	484	Loss
DEL00011727	chr1	195,349,143	195,349,257	114	Loss
DEL00011728	chr1	195,353,967	195,355,947	1,980	Loss
DEL00011746	chr1	195,365,715	195,366,639	924	Loss
DEL00011787	chr1	195,396,518	195,396,574	56	Loss
DEL00011798	chr1	195,440,208	195,440,430	222	Loss
DEL00011827	chr1	195,441,551	195,442,178	627	Loss
DEL00011834	chr1	195,443,078	195,443,135	57	Loss
DEL00011836	chr1	195,443,563	195,444,525	962	Mixed
DUP00011882	chr1	195,482,137	195,484,044	1,907	Mixed
DEL00011933	chr1	195,492,573	195,492,656	83	Loss
DEL00011935	chr1	195,498,443	195,499,163	720	Loss
DEL00011992	chr1	195,527,676	195,529,360	1,684	Loss
DEL00012197	chr1	195,621,501	195,623,193	1,692	Loss
DUP00012236	chr1	195,625,266	195,625,706	440	Mixed
DEL00012255	chr1	195,661,637	195,661,716	79	Loss
DUP00012263	chr1	195,673,385	195,673,997	612	Gain
DEL00012271	chr1	195,684,940	195,685,533	593	Loss
DEL00012299	chr1	195,745,618	195,745,985	367	Loss
DEL00012305	chr1	195,756,818	195,756,888	70	Loss
DEL00012307	chr1	195,799,424	195,799,482	58	Loss
DUP00012380	chr1	195,962,913	195,963,032	119	Gain
DEL00012381	chr1	195,992,061	195,992,346	285	Loss
DEL00012405	chr1	196,110,156	196,116,739	6,583	Loss
DEL00012414	chr1	196,132,552	196,132,727	175	Loss
DEL00012463	chr1	196,325,537	196,326,851	1,314	Loss
DUP00012478	chr1	196,387,938	196,388,579	641	Gain
DEL00012484	chr1	196,393,656	196,393,785	129	Loss
DEL00012492	chr1	196,408,165	196,408,480	315	Loss
DEL00012499	chr1	196,421,449	196,421,554	105	Loss
DUP00012509	chr1	196,448,182	196,448,294	112	Gain
DUP00012510	chr1	196,485,661	196,486,082	421	Gain
DEL00012515	chr1	196,492,756	196,493,649	893	Loss
DUP00012520	chr1	196,501,748	196,502,178	430	Gain
DEL00012534	chr1	196,535,357	196,536,311	954	Loss
DEL00012544	chr1	196,555,649	196,555,758	109	Loss
DEL00012553	chr1	196,565,341	196,565,536	195	Loss
DEL00012599	chr1	196,717,417	196,717,710	293	Loss
DEL00012613	chr1	196,858,765	196,858,816	51	Loss
DEL00012614	chr1	196,879,427	196,881,761	2,334	Loss
DEL00012615	chr1	196,884,337	196,885,568	1,231	Loss

DUP00012618	chr1	196,922,732	196,922,973	241	Gain
DUP00012641	chr1	197,000,283	197,011,953	11,670	Mixed
DEL00012687	chr1	197,079,152	197,079,204	52	Loss
DEL00012689	chr1	197,082,772	197,082,885	113	Loss
DUP00012694	chr1	197,086,863	197,087,420	557	Gain
DEL00012703	chr1	197,110,537	197,110,596	59	Loss
DUP00012712	chr1	197,117,928	197,118,331	403	Gain
DEL00012719	chr1	197,125,358	197,126,105	747	Mixed
DUP00012733	chr1	197,134,214	197,134,753	539	Gain
DEL00012758	chr1	197,200,159	197,200,694	535	Loss
DUP00012774	chr1	197,238,735	197,261,799	23,064	Gain
DEL00012792	chr1	197,294,785	197,295,135	350	Loss
DEL00012797	chr1	197,306,406	197,306,534	128	Loss
DEL00012803	chr1	197,382,762	197,382,816	54	Loss
DEL00012805	chr1	197,393,602	197,393,984	382	Loss
DUP00012806	chr1	197,409,477	197,409,760	283	Mixed
DUP00012814	chr1	197,435,549	197,438,596	3,047	Gain
DEL00012835	chr1	197,461,251	197,462,059	808	Loss
DEL00012843	chr1	197,495,948	197,496,004	56	Loss
DEL00012850	chr1	197,549,985	197,550,042	57	Loss
DEL00012871	chr2	186,494	186,595	101	Loss
DEL00012896	chr2	319,585	320,932	1,347	Loss
DEL00012946	chr2	428,292	428,856	564	Loss
DUP00012947	chr2	450,608	451,096	488	Gain
DEL00012949	chr2	480,741	480,911	170	Loss
DEL00012958	chr2	515,390	515,748	358	Loss
DEL00013010	chr2	591,952	592,019	67	Loss
DEL00013027	chr2	603,128	603,199	71	Loss
DEL00013045	chr2	619,091	619,555	464	Loss
DEL00013063	chr2	644,515	645,457	942	Mixed
DEL00013110	chr2	804,255	804,357	102	Loss
DUP00013136	chr2	903,432	910,658	7,226	Gain
DEL00013147	chr2	982,141	982,465	324	Loss
DEL00013149	chr2	1,000,565	1,005,925	5,360	Loss
DEL00013150	chr2	1,017,970	1,018,694	724	Loss
DEL00013154	chr2	1,116,658	1,116,716	58	Loss
DEL00013165	chr2	1,250,468	1,250,685	217	Loss
DEL00013170	chr2	1,295,319	1,295,397	78	Loss
DEL00013171	chr2	1,317,444	1,317,507	63	Loss
DEL00013176	chr2	1,387,174	1,387,243	69	Loss
DEL00013178	chr2	1,407,425	1,407,827	402	Loss
DEL00013179	chr2	1,474,974	1,475,909	935	Loss
DEL00013184	chr2	1,537,217	1,537,809	592	Loss
DUP00013193	chr2	1,604,949	1,613,945	8,996	Gain
DEL00013209	chr2	1,752,004	1,752,317	313	Loss
DEL00013213	chr2	1,759,330	1,759,468	138	Loss
DEL00013225	chr2	1,923,119	1,923,355	236	Loss
DUP00013227	chr2	1,938,747	1,938,860	113	Gain
DEL00013229	chr2	1,971,478	1,972,050	572	Loss

DEL00013233	chr2	2,085,046	2,085,530	484	Loss
DUP00013242	chr2	2,154,254	2,155,760	1,506	Gain
DEL00013255	chr2	2,287,365	2,287,492	127	Loss
DEL00013270	chr2	2,361,636	2,362,230	594	Loss
DEL00013280	chr2	2,538,182	2,538,420	238	Loss
DEL00013284	chr2	2,601,167	2,601,247	80	Loss
DEL00013286	chr2	2,614,004	2,614,237	233	Loss
DEL00013299	chr2	2,880,025	2,881,137	1,112	Loss
DEL00013301	chr2	2,960,642	2,960,790	148	Loss
DEL00013310	chr2	3,195,351	3,195,783	432	Loss
DEL00013322	chr2	3,328,288	3,328,646	358	Loss
DEL00013324	chr2	3,350,820	3,351,080	260	Loss
DEL00013330	chr2	3,393,912	3,393,969	57	Loss
DEL00013343	chr2	3,510,104	3,510,157	53	Loss
DUP00013351	chr2	3,797,111	3,797,221	110	Gain
DEL00013354	chr2	3,863,749	3,863,853	104	Loss
DEL00013360	chr2	4,038,911	4,039,012	101	Loss
DEL00013367	chr2	4,100,244	4,101,030	786	Loss
DEL00013375	chr2	4,206,764	4,207,121	357	Loss
DEL00013377	chr2	4,212,643	4,212,970	327	Loss
DEL00013378	chr2	4,222,022	4,223,691	1,669	Loss
DEL00013386	chr2	4,294,358	4,294,578	220	Loss
DEL00013388	chr2	4,331,007	4,331,353	346	Loss
DEL00013395	chr2	4,381,219	4,381,365	146	Loss
DEL00013397	chr2	4,447,435	4,447,619	184	Loss
DEL00013399	chr2	4,494,110	4,494,181	71	Loss
DEL00013400	chr2	4,499,235	4,499,755	520	Loss
DEL00013402	chr2	4,525,202	4,525,354	152	Loss
DEL00013405	chr2	4,594,234	4,594,418	184	Loss
DEL00013408	chr2	4,647,165	4,647,333	168	Loss
DEL00013414	chr2	4,792,141	4,805,863	13,722	Loss
DEL00013415	chr2	4,827,767	4,827,935	168	Loss
DEL00013427	chr2	4,914,063	4,914,114	51	Loss
DEL00013435	chr2	5,059,875	5,061,304	1,429	Loss
DEL00013438	chr2	5,147,654	5,147,727	73	Loss
DEL00013445	chr2	5,279,602	5,279,897	295	Loss
DEL00013450	chr2	5,364,429	5,364,501	72	Loss
DEL00013453	chr2	5,441,451	5,441,623	172	Loss
DEL00013457	chr2	5,581,439	5,581,735	296	Loss
DEL00013459	chr2	5,589,549	5,589,641	92	Loss
DEL00013460	chr2	5,605,136	5,605,212	76	Loss
DUP00013461	chr2	5,666,273	5,666,567	294	Gain
DEL00013462	chr2	5,764,900	5,765,399	499	Loss
DEL00013466	chr2	5,786,853	5,786,923	70	Loss
DEL00013468	chr2	5,827,259	5,827,535	276	Loss
DEL00013474	chr2	6,031,763	6,031,859	96	Loss
DEL00013477	chr2	6,125,215	6,125,381	166	Loss
DEL00013478	chr2	6,127,761	6,128,031	270	Loss
DEL00013486	chr2	6,218,586	6,218,637	51	Loss

DEL00013488	chr2	6,275,251	6,275,822	571	Loss
DEL00013489	chr2	6,296,036	6,296,104	68	Loss
DEL00013490	chr2	6,298,408	6,298,555	147	Loss
DEL00013493	chr2	6,325,109	6,325,768	659	Loss
DEL00013495	chr2	6,342,791	6,342,872	81	Loss
DEL00013496	chr2	6,369,273	6,369,328	55	Loss
DEL00013505	chr2	6,513,741	6,513,801	60	Loss
DEL00013507	chr2	6,520,988	6,521,452	464	Loss
DEL00013522	chr2	6,639,793	6,639,877	84	Loss
DEL00013526	chr2	6,671,197	6,671,496	299	Loss
DEL00013528	chr2	6,729,304	6,729,393	89	Loss
DEL00013531	chr2	6,754,574	6,755,174	600	Loss
DEL00013532	chr2	6,787,249	6,787,538	289	Loss
DEL00013538	chr2	6,815,755	6,816,581	826	Loss
DEL00013539	chr2	6,827,593	6,827,775	182	Loss
DEL00013541	chr2	6,899,824	6,900,074	250	Loss
DEL00013544	chr2	6,967,732	6,967,817	85	Loss
DEL00013545	chr2	6,974,639	6,979,298	4,659	Loss
DEL00013547	chr2	7,000,428	7,000,521	93	Loss
DEL00013551	chr2	7,096,211	7,096,322	111	Loss
DEL00013552	chr2	7,119,655	7,119,731	76	Loss
DEL00013555	chr2	7,165,617	7,165,723	106	Loss
DEL00013556	chr2	7,172,000	7,174,332	2,332	Loss
DEL00013558	chr2	7,219,493	7,219,674	181	Loss
DEL00013560	chr2	7,293,491	7,294,010	519	Loss
DEL00013573	chr2	7,563,970	7,566,639	2,669	Loss
DEL00013584	chr2	7,701,594	7,701,922	328	Loss
DEL00013586	chr2	7,722,817	7,723,236	419	Loss
DEL00013593	chr2	7,914,676	7,914,790	114	Loss
DEL00013598	chr2	8,077,847	8,078,049	202	Loss
DEL00013601	chr2	8,165,079	8,166,205	1,126	Loss
DEL00013604	chr2	8,197,159	8,197,465	306	Loss
DEL00013607	chr2	8,238,388	8,238,445	57	Loss
DEL00013611	chr2	8,309,737	8,309,835	98	Loss
DUP00013614	chr2	8,310,692	8,310,788	96	Gain
DEL00013615	chr2	8,317,073	8,317,332	259	Loss
DEL00013625	chr2	8,558,026	8,558,081	55	Loss
DEL00013626	chr2	8,571,618	8,571,774	156	Loss
DEL00013633	chr2	8,698,381	8,698,472	91	Loss
DEL00013634	chr2	8,745,911	8,746,448	537	Loss
DEL00013635	chr2	8,779,435	8,779,523	88	Loss
DEL00013645	chr2	9,018,280	9,019,657	1,377	Loss
DEL00013646	chr2	9,038,816	9,038,893	77	Loss
DEL00013649	chr2	9,076,807	9,077,324	517	Loss
DEL00013650	chr2	9,141,188	9,147,538	6,350	Loss
DEL00013651	chr2	9,167,699	9,168,251	552	Loss
DEL00013653	chr2	9,262,379	9,262,445	66	Loss
DEL00013654	chr2	9,268,209	9,268,424	215	Loss
DEL00013656	chr2	9,311,647	9,312,540	893	Loss

DEL00013661	chr2	9,378,780	9,378,888	108	Loss
DEL00013662	chr2	9,386,834	9,387,335	501	Loss
DEL00013663	chr2	9,388,040	9,388,121	81	Loss
DEL00013665	chr2	9,412,053	9,412,235	182	Loss
DEL00013666	chr2	9,413,870	9,413,968	98	Loss
DEL00013668	chr2	9,449,371	9,449,491	120	Loss
DEL00013669	chr2	9,461,239	9,461,592	353	Loss
DEL00013670	chr2	9,468,936	9,469,022	86	Loss
DEL00013672	chr2	9,480,048	9,480,876	828	Loss
DEL00013678	chr2	9,537,412	9,537,720	308	Loss
DEL00013679	chr2	9,550,854	9,551,405	551	Loss
DEL00013685	chr2	9,615,507	9,616,037	530	Loss
DEL00013687	chr2	9,670,926	9,671,079	153	Loss
DEL00013688	chr2	9,693,738	9,694,269	531	Loss
DEL00013691	chr2	9,767,269	9,767,421	152	Loss
DEL00013698	chr2	9,845,209	9,845,264	55	Loss
DEL00013699	chr2	9,847,725	9,851,478	3,753	Loss
DEL00013709	chr2	9,883,588	9,883,816	228	Loss
DEL00013710	chr2	9,935,228	9,935,505	277	Loss
DEL00013711	chr2	9,947,644	9,947,800	156	Loss
DEL00013722	chr2	10,138,475	10,138,567	92	Loss
DEL00013723	chr2	10,156,251	10,156,846	595	Loss
DEL00013724	chr2	10,199,764	10,199,820	56	Loss
DEL00013725	chr2	10,203,920	10,204,405	485	Loss
DEL00013731	chr2	10,343,137	10,343,260	123	Loss
DEL00013734	chr2	10,382,322	10,382,902	580	Loss
DEL00013741	chr2	10,445,978	10,446,081	103	Loss
DEL00013742	chr2	10,447,624	10,452,352	4,728	Loss
DEL00013744	chr2	10,518,550	10,519,149	599	Loss
DEL00013745	chr2	10,533,474	10,533,706	232	Loss
DEL00013746	chr2	10,576,174	10,576,595	421	Loss
DEL00013749	chr2	10,634,430	10,634,503	73	Loss
DEL00013760	chr2	10,824,605	10,824,826	221	Loss
DEL00013764	chr2	10,918,530	10,918,668	138	Loss
DEL00013766	chr2	10,924,030	10,924,522	492	Loss
DEL00013772	chr2	10,961,939	10,962,176	237	Loss
DEL00013773	chr2	10,972,750	10,974,254	1,504	Loss
DEL00013782	chr2	11,079,550	11,080,510	960	Loss
DEL00013787	chr2	11,141,336	11,141,405	69	Loss
DEL00013789	chr2	11,146,068	11,146,927	859	Loss
DEL00013793	chr2	11,232,667	11,232,831	164	Loss
DEL00013798	chr2	11,355,450	11,356,481	1,031	Loss
DEL00013800	chr2	11,363,396	11,363,736	340	Loss
DEL00013801	chr2	11,364,923	11,365,028	105	Loss
DEL00013807	chr2	11,547,330	11,551,244	3,914	Loss
DEL00013812	chr2	11,674,422	11,674,474	52	Loss
DEL00013816	chr2	11,747,137	11,749,412	2,275	Loss
DEL00013820	chr2	11,833,943	11,838,558	4,615	Loss
DEL00013826	chr2	11,973,871	11,973,936	65	Loss

DEL00013831	chr2	12,062,872	12,064,339	1,467	Loss
DEL00013836	chr2	12,126,226	12,126,326	100	Loss
DEL00013838	chr2	12,196,976	12,197,195	219	Loss
DEL00013839	chr2	12,210,642	12,211,562	920	Loss
DEL00013844	chr2	12,279,965	12,281,013	1,048	Loss
DEL00013845	chr2	12,284,891	12,285,910	1,019	Loss
DEL00013846	chr2	12,289,315	12,289,391	76	Loss
DEL00013851	chr2	12,366,761	12,366,884	123	Loss
DEL00013853	chr2	12,395,739	12,395,905	166	Loss
DEL00013857	chr2	12,417,582	12,417,960	378	Loss
DEL00013859	chr2	12,463,466	12,463,542	76	Loss
DEL00013871	chr2	12,611,124	12,611,242	118	Loss
DEL00013873	chr2	12,629,992	12,630,758	766	Loss
DEL00013875	chr2	12,634,785	12,635,926	1,141	Loss
DEL00013880	chr2	12,739,704	12,739,789	85	Loss
DEL00013891	chr2	12,879,762	12,879,827	65	Loss
DEL00013903	chr2	13,103,903	13,104,123	220	Loss
DEL00013906	chr2	13,125,700	13,125,783	83	Loss
DEL00013907	chr2	13,141,878	13,142,324	446	Loss
DEL00013908	chr2	13,163,559	13,163,641	82	Loss
DEL00013912	chr2	13,186,968	13,187,019	51	Loss
DEL00013913	chr2	13,219,449	13,219,505	56	Loss
DEL00013938	chr2	13,760,743	13,760,851	108	Loss
DEL00013940	chr2	13,880,381	13,880,672	291	Loss
DEL00013942	chr2	14,004,423	14,004,533	110	Loss
DEL00013951	chr2	14,072,304	14,072,559	255	Loss
DEL00013953	chr2	14,084,404	14,084,558	154	Loss
DEL00013958	chr2	14,251,614	14,251,835	221	Loss
DEL00013960	chr2	14,272,016	14,272,430	414	Loss
DEL00013976	chr2	14,674,119	14,674,858	739	Loss
DEL00013979	chr2	14,699,020	14,699,099	79	Loss
DEL00013985	chr2	14,811,273	14,811,496	223	Loss
DEL00013988	chr2	14,883,651	14,883,726	75	Loss
DEL00013991	chr2	14,976,136	14,976,187	51	Loss
DEL00013994	chr2	14,982,518	14,983,035	517	Loss
DEL00013995	chr2	14,986,234	14,986,756	522	Loss
DEL00014002	chr2	15,149,901	15,150,097	196	Loss
DEL00014013	chr2	15,286,854	15,286,988	134	Loss
DEL00014030	chr2	15,593,323	15,593,455	132	Loss
DEL00014043	chr2	15,823,156	15,823,213	57	Loss
DEL00014056	chr2	15,947,975	15,948,036	61	Loss
DEL00014058	chr2	15,973,989	15,974,044	55	Loss
DEL00014060	chr2	16,069,906	16,069,960	54	Loss
DEL00014062	chr2	16,136,362	16,138,509	2,147	Loss
DEL00014065	chr2	16,164,602	16,164,692	90	Loss
DEL00014066	chr2	16,194,165	16,194,661	496	Loss
DEL00014070	chr2	16,224,715	16,224,948	233	Loss
DEL00014075	chr2	16,250,208	16,250,290	82	Loss
DEL00014076	chr2	16,257,685	16,257,740	55	Loss

DEL00014082	chr2	16,387,905	16,387,999	94	Loss
DEL00014085	chr2	16,430,218	16,430,744	526	Loss
DEL00014087	chr2	16,467,647	16,468,987	1,340	Loss
DEL00014092	chr2	16,591,206	16,591,290	84	Loss
DEL00014104	chr2	16,719,481	16,719,556	75	Loss
DEL00014105	chr2	16,735,531	16,735,743	212	Loss
DEL00014109	chr2	17,002,601	17,002,777	176	Loss
DEL00014111	chr2	17,028,373	17,028,920	547	Loss
DEL00014116	chr2	17,189,503	17,189,573	70	Loss
DEL00014119	chr2	17,215,715	17,215,832	117	Loss
DEL00014130	chr2	17,458,575	17,458,885	310	Loss
DUP00014138	chr2	17,602,129	17,606,571	4,442	Gain
DEL00014144	chr2	17,713,837	17,714,055	218	Loss
DEL00014147	chr2	17,786,964	17,787,683	719	Loss
DEL00014160	chr2	17,983,134	17,983,187	53	Loss
DEL00014161	chr2	18,016,806	18,016,885	79	Loss
DEL00014162	chr2	18,027,293	18,027,646	353	Loss
DEL00014165	chr2	18,057,663	18,057,904	241	Loss
DEL00014166	chr2	18,083,379	18,087,419	4,040	Loss
DEL00014167	chr2	18,149,018	18,149,086	68	Loss
DEL00014168	chr2	18,192,326	18,192,404	78	Loss
DEL00014173	chr2	18,270,910	18,271,005	95	Loss
DEL00014174	chr2	18,274,904	18,278,109	3,205	Loss
DEL00014179	chr2	18,335,231	18,336,482	1,251	Loss
DEL00014187	chr2	18,560,008	18,560,088	80	Loss
DEL00014188	chr2	18,560,277	18,560,331	54	Loss
DEL00014193	chr2	18,644,094	18,645,423	1,329	Loss
DEL00014200	chr2	18,762,113	18,762,191	78	Loss
DEL00014208	chr2	18,915,205	18,915,309	104	Loss
DUP00014214	chr2	19,055,667	19,072,178	16,511	Gain
DEL00014218	chr2	19,147,822	19,149,582	1,760	Loss
DEL00014225	chr2	19,203,967	19,204,021	54	Loss
DEL00014227	chr2	19,217,683	19,217,823	140	Loss
DEL00014229	chr2	19,248,416	19,248,816	400	Loss
DEL00014232	chr2	19,280,623	19,281,229	606	Loss
DEL00014241	chr2	19,329,375	19,329,717	342	Loss
DEL00014253	chr2	19,530,135	19,530,299	164	Loss
DEL00014263	chr2	19,720,617	19,720,763	146	Loss
DEL00014264	chr2	19,754,587	19,754,658	71	Loss
DEL00014267	chr2	19,834,266	19,834,372	106	Loss
DEL00014268	chr2	19,834,671	19,835,298	627	Loss
DEL00014271	chr2	19,926,124	19,926,211	87	Loss
DEL00014273	chr2	19,936,868	19,937,075	207	Loss
DEL00014276	chr2	19,984,489	19,984,789	300	Loss
DEL00014279	chr2	20,052,811	20,052,943	132	Loss
DEL00014283	chr2	20,145,972	20,146,709	737	Loss
DEL00014291	chr2	20,226,854	20,227,728	874	Loss
DEL00014293	chr2	20,248,020	20,248,091	71	Loss
DEL00014294	chr2	20,270,025	20,270,208	183	Loss

DEL00014297	chr2	20,296,727	20,296,792	65	Loss
DEL00014302	chr2	20,456,333	20,456,461	128	Loss
DEL00014308	chr2	20,672,783	20,673,439	656	Loss
DEL00014310	chr2	20,709,914	20,710,845	931	Loss
DUP00014311	chr2	20,720,323	20,720,943	620	Gain
DEL00014313	chr2	20,764,017	20,764,538	521	Loss
DEL00014319	chr2	20,882,129	20,882,591	462	Loss
DEL00014320	chr2	20,895,512	20,895,640	128	Loss
DEL00014325	chr2	21,012,678	21,012,921	243	Loss
DEL00014326	chr2	21,025,059	21,025,111	52	Loss
DEL00014333	chr2	21,155,260	21,155,695	435	Loss
DEL00014334	chr2	21,163,785	21,163,942	157	Loss
DEL00014337	chr2	21,189,550	21,192,914	3,364	Loss
DEL00014338	chr2	21,219,004	21,219,494	490	Loss
DEL00014340	chr2	21,250,649	21,250,751	102	Loss
DEL00014344	chr2	21,326,161	21,327,041	880	Loss
DEL00014356	chr2	21,484,110	21,484,183	73	Loss
DEL00014357	chr2	21,488,196	21,488,301	105	Loss
DUP00014358	chr2	21,509,961	21,510,050	89	Gain
DUP00014359	chr2	21,514,757	21,514,841	84	Gain
DEL00014360	chr2	21,536,055	21,536,178	123	Loss
DEL00014363	chr2	21,555,229	21,555,523	294	Loss
DEL00014370	chr2	21,782,483	21,782,657	174	Loss
DEL00014376	chr2	21,841,096	21,841,292	196	Loss
DEL00014378	chr2	21,916,436	21,916,498	62	Loss
DEL00014382	chr2	22,054,327	22,054,390	63	Loss
DEL00014383	chr2	22,066,680	22,066,932	252	Loss
DEL00014386	chr2	22,096,906	22,097,205	299	Loss
DEL00014388	chr2	22,099,141	22,099,234	93	Loss
DUP00014389	chr2	22,118,365	22,118,568	203	Gain
DEL00014397	chr2	22,334,211	22,334,285	74	Loss
DEL00014399	chr2	22,354,379	22,354,442	63	Loss
DEL00014405	chr2	22,480,747	22,480,877	130	Loss
DEL00014410	chr2	22,555,710	22,555,936	226	Loss
DEL00014413	chr2	22,765,797	22,766,486	689	Loss
DEL00014416	chr2	22,785,924	22,786,208	284	Loss
DEL00014421	chr2	22,865,447	22,865,548	101	Loss
DEL00014426	chr2	22,877,250	22,877,564	314	Loss
DEL00014428	chr2	22,919,242	22,919,414	172	Loss
DEL00014432	chr2	22,964,473	22,964,559	86	Loss
DEL00014437	chr2	23,030,544	23,030,667	123	Loss
DEL00014438	chr2	23,072,409	23,072,548	139	Loss
DEL00014442	chr2	23,147,958	23,148,044	86	Loss
DEL00014445	chr2	23,231,596	23,232,058	462	Loss
DEL00014447	chr2	23,280,250	23,280,420	170	Loss
DEL00014454	chr2	23,375,422	23,376,556	1,134	Loss
DEL00014455	chr2	23,387,314	23,387,393	79	Loss
DEL00014464	chr2	23,477,458	23,477,548	90	Loss
DUP00014466	chr2	23,567,055	23,567,131	76	Gain

DEL00014471	chr2	23,708,238	23,709,617	1,379	Loss
DEL00014476	chr2	23,831,366	23,831,436	70	Loss
DEL00014484	chr2	24,081,487	24,081,551	64	Loss
DEL00014485	chr2	24,155,638	24,155,722	84	Loss
DEL00014492	chr2	24,397,731	24,398,101	370	Loss
DEL00014493	chr2	24,448,494	24,448,893	399	Loss
DEL00014495	chr2	24,482,054	24,482,107	53	Loss
DEL00014507	chr2	24,722,234	24,722,404	170	Loss
DEL00014511	chr2	24,746,007	24,746,199	192	Loss
DEL00014512	chr2	24,748,569	24,749,044	475	Loss
DEL00014514	chr2	24,752,222	24,752,522	300	Loss
DEL00014515	chr2	24,775,977	24,776,491	514	Loss
DEL00014518	chr2	24,808,990	24,809,100	110	Loss
DEL00014523	chr2	25,081,249	25,081,636	387	Loss
DEL00014526	chr2	25,098,537	25,098,626	89	Loss
DEL00014533	chr2	25,265,542	25,266,761	1,219	Loss
DEL00014537	chr2	25,353,447	25,353,622	175	Loss
DEL00014542	chr2	25,477,053	25,477,110	57	Loss
DEL00014543	chr2	25,477,169	25,477,729	560	Loss
DEL00014544	chr2	25,537,281	25,538,219	938	Loss
DEL00014546	chr2	25,545,349	25,545,648	299	Loss
DEL00014555	chr2	25,651,893	25,652,498	605	Loss
DEL00014556	chr2	25,689,029	25,689,629	600	Loss
DEL00014557	chr2	25,718,169	25,719,527	1,358	Loss
DEL00014558	chr2	25,744,979	25,746,729	1,750	Loss
DEL00014562	chr2	25,768,115	25,768,172	57	Loss
DEL00014563	chr2	25,796,504	25,796,568	64	Loss
DEL00014564	chr2	25,824,082	25,824,250	168	Loss
DEL00014567	chr2	25,852,644	25,853,203	559	Loss
DEL00014574	chr2	26,038,229	26,039,521	1,292	Loss
DEL00014576	chr2	26,116,458	26,116,528	70	Loss
DEL00014580	chr2	26,145,930	26,146,175	245	Loss
DEL00014583	chr2	26,206,283	26,206,969	686	Loss
DEL00014587	chr2	26,311,888	26,311,968	80	Loss
DEL00014598	chr2	26,597,510	26,597,728	218	Loss
DEL00014602	chr2	26,676,773	26,677,136	363	Loss
DEL00014605	chr2	26,730,853	26,730,963	110	Loss
DEL00014606	chr2	26,752,277	26,752,359	82	Loss
DEL00014607	chr2	26,818,382	26,819,412	1,030	Loss
DEL00014609	chr2	26,846,177	26,846,262	85	Loss
DEL00014631	chr2	27,333,399	27,333,635	236	Loss
DEL00014636	chr2	27,539,113	27,539,214	101	Loss
DEL00014642	chr2	27,571,822	27,571,953	131	Loss
DEL00014646	chr2	27,628,223	27,631,472	3,249	Loss
DEL00014655	chr2	27,954,796	27,955,412	616	Loss
DEL00014660	chr2	28,030,703	28,032,293	1,590	Loss
DEL00014662	chr2	28,096,191	28,098,412	2,221	Loss
DEL00014663	chr2	28,108,369	28,108,449	80	Loss
DUP00014664	chr2	28,132,529	28,132,665	136	Gain

DEL00014668	chr2	28,162,094	28,163,512	1,418	Loss
DEL00014669	chr2	28,167,326	28,167,493	167	Loss
DEL00014671	chr2	28,169,770	28,170,216	446	Loss
DEL00014683	chr2	28,277,227	28,277,380	153	Loss
DEL00014687	chr2	28,328,403	28,328,515	112	Loss
DEL00014692	chr2	28,418,861	28,419,016	155	Loss
DEL00014693	chr2	28,443,350	28,443,454	104	Loss
DEL00014702	chr2	28,650,915	28,652,043	1,128	Loss
DEL00014707	chr2	28,783,595	28,784,213	618	Loss
DEL00014714	chr2	28,953,493	28,953,547	54	Loss
DEL00014717	chr2	28,990,025	28,990,626	601	Loss
DEL00014718	chr2	29,080,416	29,080,584	168	Loss
DEL00014722	chr2	29,151,936	29,152,047	111	Loss
DEL00014723	chr2	29,194,289	29,194,394	105	Loss
DEL00014725	chr2	29,258,750	29,258,898	148	Loss
DEL00014726	chr2	29,330,019	29,330,208	189	Loss
DEL00014727	chr2	29,330,785	29,332,528	1,743	Loss
DEL00014730	chr2	29,408,551	29,408,614	63	Loss
DEL00014741	chr2	29,677,270	29,677,869	599	Loss
DEL00014746	chr2	29,708,060	29,708,243	183	Loss
DEL00014748	chr2	29,785,287	29,785,486	199	Loss
DEL00014754	chr2	29,874,249	29,874,300	51	Loss
DUP00014759	chr2	29,961,690	29,961,872	182	Gain
DEL00014760	chr2	29,967,720	29,969,057	1,337	Loss
DEL00014762	chr2	30,061,559	30,061,704	145	Loss
DEL00014765	chr2	30,161,167	30,161,242	75	Loss
DEL00014771	chr2	30,215,878	30,215,937	59	Loss
DEL00014773	chr2	30,258,437	30,258,925	488	Loss
DEL00014774	chr2	30,265,843	30,265,984	141	Loss
DEL00014778	chr2	30,284,900	30,285,430	530	Loss
DEL00014780	chr2	30,401,723	30,401,849	126	Loss
DEL00014789	chr2	30,529,073	30,529,240	167	Loss
DEL00014791	chr2	30,587,103	30,587,653	550	Loss
DEL00014792	chr2	30,592,004	30,592,166	162	Loss
DEL00014799	chr2	30,635,188	30,635,974	786	Loss
DEL00014802	chr2	30,723,226	30,723,278	52	Loss
DEL00014824	chr2	31,027,934	31,028,039	105	Loss
DEL00014825	chr2	31,041,093	31,041,745	652	Loss
DEL00014829	chr2	31,129,829	31,130,136	307	Loss
DEL00014841	chr2	31,324,195	31,324,570	375	Loss
DEL00014843	chr2	31,365,635	31,366,163	528	Loss
DEL00014844	chr2	31,373,331	31,373,924	593	Loss
DEL00014847	chr2	31,499,796	31,499,949	153	Loss
DEL00014849	chr2	31,525,978	31,526,750	772	Loss
DEL00014859	chr2	31,619,592	31,619,918	326	Loss
DEL00014864	chr2	31,749,313	31,749,420	107	Loss
DEL00014870	chr2	31,991,255	31,991,469	214	Loss
DEL00014871	chr2	31,999,474	31,999,686	212	Loss
DEL00014882	chr2	32,308,186	32,308,266	80	Loss

DEL00014884	chr2	32,381,457	32,381,709	252	Loss
DEL00014899	chr2	32,563,753	32,563,807	54	Loss
DEL00014902	chr2	32,580,914	32,580,968	54	Loss
DEL00014912	chr2	32,761,702	32,761,848	146	Loss
DEL00014916	chr2	32,853,514	32,854,014	500	Loss
DEL00014917	chr2	32,878,525	32,878,673	148	Loss
DEL00014918	chr2	32,919,829	32,920,375	546	Loss
DEL00014928	chr2	33,138,550	33,139,027	477	Loss
DEL00014932	chr2	33,211,602	33,211,665	63	Loss
DEL00014933	chr2	33,216,515	33,216,647	132	Loss
DEL00014941	chr2	33,322,454	33,323,616	1,162	Loss
DEL00014952	chr2	33,666,245	33,666,326	81	Loss
DEL00014959	chr2	33,826,997	33,829,301	2,304	Loss
DEL00014965	chr2	34,036,251	34,036,374	123	Loss
DEL00014968	chr2	34,108,200	34,108,544	344	Loss
DEL00014970	chr2	34,108,683	34,108,738	55	Loss
DEL00014971	chr2	34,129,462	34,129,531	69	Loss
DEL00014976	chr2	34,175,586	34,175,681	95	Loss
DEL00014977	chr2	34,214,413	34,214,844	431	Loss
DEL00014978	chr2	34,253,522	34,253,645	123	Loss
DEL00014980	chr2	34,275,329	34,275,766	437	Loss
DEL00014983	chr2	34,345,211	34,345,644	433	Loss
DEL00014985	chr2	34,380,489	34,380,545	56	Loss
DEL00014989	chr2	34,439,206	34,439,558	352	Loss
DEL00014999	chr2	34,729,087	34,729,256	169	Loss
DEL00015003	chr2	34,898,365	34,898,475	110	Loss
DEL00015008	chr2	35,145,326	35,145,754	428	Loss
DUP00015010	chr2	35,163,651	35,181,458	17,807	Gain
DEL00015011	chr2	35,187,614	35,198,818	11,204	Loss
DEL00015012	chr2	35,226,155	35,226,379	224	Loss
DEL00015013	chr2	35,236,585	35,236,647	62	Loss
DEL00015017	chr2	35,406,158	35,406,244	86	Loss
DEL00015021	chr2	35,519,401	35,520,310	909	Loss
DEL00015024	chr2	35,634,517	35,634,581	64	Loss
DEL00015026	chr2	35,666,652	35,666,746	94	Loss
DEL00015031	chr2	35,760,220	35,760,274	54	Loss
DEL00015033	chr2	35,814,555	35,815,549	994	Loss
DEL00015038	chr2	35,879,754	35,880,089	335	Loss
DEL00015040	chr2	35,884,500	35,885,869	1,369	Loss
DEL00015044	chr2	35,958,908	35,959,742	834	Loss
DEL00015045	chr2	35,972,634	35,973,093	459	Loss
DEL00015047	chr2	35,978,351	35,978,412	61	Loss
DEL00015051	chr2	36,036,416	36,036,759	343	Loss
DEL00015053	chr2	36,060,586	36,060,707	121	Loss
DEL00015054	chr2	36,138,325	36,138,572	247	Loss
DEL00015056	chr2	36,139,340	36,145,685	6,345	Loss
DEL00015058	chr2	36,174,505	36,174,599	94	Loss
DEL00015064	chr2	36,354,233	36,367,802	13,569	Loss
DUP00015066	chr2	36,392,506	36,392,633	127	Gain

DEL00015072	chr2	36,459,120	36,459,321	201	Loss
DEL00015073	chr2	36,500,559	36,501,983	1,424	Loss
DEL00015075	chr2	36,506,612	36,506,896	284	Loss
DEL00015076	chr2	36,585,082	36,585,274	192	Loss
DEL00015077	chr2	36,612,600	36,617,999	5,399	Loss
DEL00015081	chr2	36,655,479	36,655,869	390	Loss
DEL00015084	chr2	36,786,153	36,786,427	274	Loss
DEL00015088	chr2	36,884,359	36,884,618	259	Loss
DEL00015092	chr2	36,930,034	36,930,169	135	Loss
DEL00015093	chr2	36,961,571	36,961,797	226	Loss
DEL00015095	chr2	37,017,660	37,017,717	57	Loss
DEL00015106	chr2	37,377,688	37,377,739	51	Loss
DEL00015107	chr2	37,392,931	37,393,170	239	Loss
DEL00015110	chr2	37,446,978	37,447,098	120	Loss
DEL00015113	chr2	37,475,806	37,475,877	71	Loss
DEL00015118	chr2	37,550,466	37,551,298	832	Loss
DEL00015126	chr2	37,744,424	37,744,534	110	Loss
DEL00015131	chr2	37,807,448	37,809,539	2,091	Loss
DEL00015140	chr2	38,029,683	38,030,344	661	Loss
DEL00015141	chr2	38,082,126	38,083,605	1,479	Loss
DEL00015158	chr2	38,320,090	38,320,154	64	Loss
DEL00015160	chr2	38,371,666	38,371,774	108	Loss
DEL00015161	chr2	38,382,062	38,382,312	250	Loss
DEL00015164	chr2	38,507,968	38,508,778	810	Loss
DEL00015167	chr2	38,566,073	38,566,131	58	Loss
DEL00015168	chr2	38,586,287	38,586,356	69	Loss
DEL00015180	chr2	38,745,277	38,745,988	711	Loss
DEL00015181	chr2	38,768,572	38,768,626	54	Loss
DEL00015184	chr2	38,794,202	38,794,253	51	Loss
DEL00015189	chr2	38,930,031	38,930,154	123	Loss
DEL00015190	chr2	38,971,332	38,971,558	226	Loss
DEL00015192	chr2	39,129,127	39,129,913	786	Loss
DEL00015196	chr2	39,217,560	39,217,795	235	Loss
DEL00015199	chr2	39,259,927	39,260,145	218	Loss
DEL00015205	chr2	39,362,349	39,362,502	153	Loss
DEL00015220	chr2	39,591,729	39,592,320	591	Loss
DEL00015221	chr2	39,605,500	39,605,579	79	Loss
DEL00015223	chr2	39,606,917	39,607,482	565	Loss
DEL00015229	chr2	39,744,151	39,744,462	311	Loss
DEL00015230	chr2	39,838,971	39,839,064	93	Loss
DEL00015235	chr2	40,038,775	40,039,149	374	Loss
DEL00015236	chr2	40,100,818	40,100,878	60	Loss
DEL00015330	chr2	40,546,736	40,547,560	824	Loss
DEL00015332	chr2	40,579,539	40,579,606	67	Loss
DEL00015336	chr2	40,602,126	40,602,184	58	Loss
DEL00015339	chr2	40,620,352	40,620,555	203	Loss
DEL00015343	chr2	40,679,069	40,679,450	381	Loss
DEL00015344	chr2	40,679,476	40,680,111	635	Loss
DEL00015346	chr2	40,827,591	40,827,899	308	Loss

DEL00015353	chr2	40,901,456	40,902,049	593	Loss
DEL00015354	chr2	40,921,242	40,921,311	69	Loss
DEL00015361	chr2	41,096,346	41,096,619	273	Loss
DEL00015362	chr2	41,100,632	41,101,067	435	Loss
DEL00015366	chr2	41,217,727	41,218,236	509	Loss
DEL00015372	chr2	41,304,154	41,304,366	212	Loss
DEL00015377	chr2	41,385,104	41,385,233	129	Loss
DEL00015381	chr2	41,413,988	41,414,263	275	Loss
DEL00015382	chr2	41,418,117	41,418,595	478	Loss
DEL00015383	chr2	41,436,802	41,436,867	65	Loss
DEL00015385	chr2	41,457,712	41,458,313	601	Loss
DEL00015386	chr2	41,465,172	41,465,232	60	Loss
DEL00015392	chr2	41,615,635	41,616,215	580	Loss
DEL00015393	chr2	41,639,734	41,639,815	81	Loss
DEL00015400	chr2	41,676,538	41,677,263	725	Loss
DUP00015403	chr2	41,736,960	41,737,028	68	Gain
DEL00015411	chr2	41,870,069	41,879,624	9,555	Loss
DEL00015417	chr2	41,931,745	41,934,475	2,730	Loss
DEL00015418	chr2	41,980,316	41,980,367	51	Loss
DEL00015419	chr2	41,984,788	41,984,903	115	Loss
DEL00015422	chr2	42,049,090	42,050,442	1,352	Loss
DEL00015425	chr2	42,099,157	42,099,458	301	Loss
DEL00015431	chr2	42,161,306	42,161,611	305	Loss
DEL00015434	chr2	42,287,958	42,288,018	60	Loss
DUP00015441	chr2	42,548,107	42,548,177	70	Gain
DEL00015443	chr2	42,563,940	42,564,018	78	Loss
DEL00015446	chr2	42,735,664	42,736,060	396	Loss
DEL00015449	chr2	42,796,480	42,796,869	389	Loss
DEL00015461	chr2	42,933,578	42,933,882	304	Loss
DEL00015462	chr2	42,940,193	42,941,019	826	Loss
DEL00015467	chr2	42,961,888	42,961,978	90	Loss
DEL00015471	chr2	43,008,388	43,008,498	110	Loss
DEL00015472	chr2	43,014,195	43,014,272	77	Loss
DEL00015479	chr2	43,070,756	43,071,177	421	Loss
DEL00015480	chr2	43,124,325	43,125,124	799	Loss
DEL00015482	chr2	43,201,168	43,201,230	62	Loss
DEL00015484	chr2	43,258,288	43,258,386	98	Loss
DEL00015492	chr2	43,473,242	43,474,094	852	Loss
DEL00015493	chr2	43,519,414	43,519,561	147	Loss
DEL00015495	chr2	43,550,400	43,550,680	280	Loss
DEL00015501	chr2	43,649,573	43,650,639	1,066	Loss
DEL00015504	chr2	43,675,644	43,676,198	554	Loss
DEL00015523	chr2	43,922,562	43,922,869	307	Loss
DEL00015524	chr2	43,946,284	43,946,371	87	Loss
DEL00015525	chr2	43,970,921	43,971,608	687	Loss
DEL00015526	chr2	43,975,043	43,975,154	111	Loss
DEL00015532	chr2	44,043,395	44,043,478	83	Loss
DEL00015533	chr2	44,043,696	44,043,877	181	Loss
DEL00015534	chr2	44,049,724	44,050,338	614	Loss

DEL00015537	chr2	44,071,590	44,071,725	135	Loss
DEL00015540	chr2	44,144,139	44,144,492	353	Loss
DEL00015543	chr2	44,219,845	44,220,636	791	Loss
DEL00015546	chr2	44,279,623	44,280,421	798	Loss
DEL00015549	chr2	44,341,909	44,342,296	387	Loss
DEL00015559	chr2	44,478,456	44,479,002	546	Loss
DEL00015563	chr2	44,561,179	44,561,296	117	Loss
DEL00015573	chr2	44,631,872	44,631,997	125	Loss
DEL00015576	chr2	44,699,058	44,699,182	124	Loss
DEL00015583	chr2	44,854,487	44,854,684	197	Loss
DEL00015586	chr2	44,916,103	44,916,179	76	Loss
DEL00015587	chr2	44,918,204	44,918,686	482	Loss
DEL00015596	chr2	44,994,125	44,994,296	171	Loss
DEL00015597	chr2	45,027,174	45,027,231	57	Loss
DEL00015600	chr2	45,038,943	45,039,442	499	Loss
DEL00015602	chr2	45,049,829	45,050,121	292	Loss
DEL00015603	chr2	45,051,300	45,051,401	101	Loss
DEL00015604	chr2	45,099,519	45,099,578	59	Loss
DEL00015609	chr2	45,143,520	45,143,578	58	Loss
DEL00015623	chr2	45,475,578	45,475,969	391	Loss
DEL00015625	chr2	45,517,921	45,521,734	3,813	Loss
DEL00015637	chr2	45,543,461	45,543,803	342	Loss
DEL00015638	chr2	45,547,882	45,548,496	614	Loss
DEL00015643	chr2	45,593,528	45,593,629	101	Loss
DEL00015645	chr2	45,607,597	45,608,006	409	Loss
DEL00015647	chr2	45,628,162	45,628,430	268	Loss
DEL00015649	chr2	45,720,386	45,720,471	85	Loss
DEL00015651	chr2	45,740,992	45,741,123	131	Loss
DEL00015653	chr2	45,744,586	45,744,775	189	Loss
DUP00015662	chr2	45,793,187	45,793,336	149	Gain
DEL00015663	chr2	45,820,177	45,820,632	455	Loss
DEL00015668	chr2	45,896,437	45,896,507	70	Loss
DEL00015671	chr2	45,953,944	45,954,117	173	Loss
DEL00015672	chr2	45,955,836	45,956,577	741	Loss
DEL00015687	chr2	46,094,441	46,094,519	78	Loss
DEL00015688	chr2	46,115,237	46,119,866	4,629	Loss
DEL00015696	chr2	46,209,926	46,210,518	592	Loss
DEL00015698	chr2	46,212,098	46,212,396	298	Loss
DEL00015702	chr2	46,297,944	46,298,001	57	Loss
DEL00015707	chr2	46,363,570	46,363,630	60	Loss
DEL00015708	chr2	46,437,337	46,437,398	61	Loss
DEL00015710	chr2	46,446,173	46,447,225	1,052	Loss
DEL00015712	chr2	46,485,343	46,485,454	111	Loss
DEL00015713	chr2	46,486,883	46,487,060	177	Loss
DEL00015716	chr2	46,578,199	46,578,364	165	Loss
DEL00015722	chr2	46,626,521	46,627,464	943	Loss
DEL00015725	chr2	46,645,502	46,646,069	567	Loss
DEL00015726	chr2	46,757,417	46,758,463	1,046	Loss
DEL00015730	chr2	46,818,078	46,819,249	1,171	Loss

DEL00015735	chr2	46,903,146	46,903,239	93	Loss
DEL00015749	chr2	47,231,256	47,231,374	118	Loss
DEL00015750	chr2	47,250,031	47,250,086	55	Loss
DEL00015761	chr2	47,440,865	47,441,259	394	Loss
DEL00015773	chr2	47,687,437	47,687,853	416	Loss
DEL00015776	chr2	47,723,373	47,723,664	291	Loss
DEL00015782	chr2	47,781,584	47,781,886	302	Loss
DEL00015786	chr2	47,795,493	47,795,864	371	Loss
DEL00015788	chr2	47,811,158	47,811,219	61	Loss
DEL00015792	chr2	47,863,562	47,863,903	341	Loss
DEL00015824	chr2	48,260,068	48,260,264	196	Loss
DEL00015825	chr2	48,266,037	48,266,149	112	Loss
DEL00015826	chr2	48,286,594	48,287,697	1,103	Loss
DEL00015846	chr2	48,586,232	48,586,842	610	Loss
DEL00015849	chr2	48,598,009	48,598,357	348	Loss
DEL00015858	chr2	48,721,877	48,722,170	293	Loss
DEL00015859	chr2	48,722,499	48,722,970	471	Loss
DEL00015862	chr2	48,750,911	48,751,763	852	Loss
DEL00015863	chr2	48,815,970	48,816,261	291	Loss
DEL00015869	chr2	48,879,139	48,879,214	75	Loss
DEL00015873	chr2	48,918,586	48,919,744	1,158	Loss
DEL00015974	chr2	49,062,885	49,063,362	477	Loss
DEL00015976	chr2	49,138,035	49,138,402	367	Loss
DEL00015977	chr2	49,152,095	49,152,290	195	Loss
DEL00015979	chr2	49,197,355	49,197,407	52	Loss
DEL00015980	chr2	49,202,245	49,202,936	691	Loss
DEL00015983	chr2	49,212,382	49,212,468	86	Loss
DEL00015987	chr2	49,298,825	49,299,004	179	Loss
DEL00015991	chr2	49,367,144	49,367,319	175	Loss
DEL00015992	chr2	49,379,060	49,379,168	108	Loss
DEL00015994	chr2	49,439,565	49,439,701	136	Loss
DEL00015998	chr2	49,510,643	49,510,810	167	Loss
DUP00016010	chr2	49,654,541	49,659,954	5,413	Mixed
DEL00016012	chr2	49,687,357	49,687,572	215	Loss
DEL00016014	chr2	49,742,516	49,742,934	418	Loss
DEL00016018	chr2	49,827,679	49,827,775	96	Loss
DEL00016026	chr2	49,970,944	49,971,144	200	Loss
DEL00016029	chr2	50,068,634	50,068,787	153	Loss
DEL00016030	chr2	50,130,002	50,131,743	1,741	Loss
DEL00016032	chr2	50,271,871	50,272,039	168	Loss
DEL00016038	chr2	50,601,132	50,601,524	392	Loss
DEL00016051	chr2	50,973,855	50,974,377	522	Loss
DEL00016052	chr2	50,994,750	50,996,292	1,542	Loss
DEL00016058	chr2	51,165,334	51,165,476	142	Loss
DEL00016059	chr2	51,217,377	51,219,324	1,947	Loss
DUP00016062	chr2	51,268,456	51,268,676	220	Gain
DEL00016065	chr2	51,428,367	51,428,839	472	Loss
DEL00016066	chr2	51,429,737	51,429,855	118	Loss
DEL00016072	chr2	51,587,177	51,587,345	168	Loss

DEL00016073	chr2	51,587,380	51,587,661	281	Loss
DEL00016086	chr2	51,862,188	51,862,428	240	Loss
DEL00016087	chr2	51,907,684	51,907,847	163	Loss
DEL00016089	chr2	52,003,342	52,003,704	362	Loss
DEL00016091	chr2	52,057,776	52,057,828	52	Loss
DEL00016095	chr2	52,717,650	52,737,875	20,225	Loss
DEL00016096	chr2	52,749,830	52,762,862	13,032	Loss
DEL00016102	chr2	52,872,214	52,873,002	788	Loss
DEL00016106	chr2	52,998,618	53,001,688	3,070	Loss
DEL00016121	chr2	53,097,056	53,098,687	1,631	Loss
DEL00016123	chr2	53,102,128	53,102,471	343	Loss
DEL00016141	chr2	53,386,210	53,386,271	61	Loss
DEL00016148	chr2	53,616,133	53,616,184	51	Loss
DEL00016165	chr2	53,963,660	53,963,821	161	Loss
DEL00016169	chr2	54,024,168	54,024,317	149	Loss
DEL00016170	chr2	54,104,773	54,105,403	630	Loss
DEL00016175	chr2	54,161,733	54,163,941	2,208	Loss
DEL00016176	chr2	54,171,346	54,171,775	429	Loss
DEL00016186	chr2	54,232,313	54,233,334	1,021	Loss
DEL00016188	chr2	54,277,766	54,278,928	1,162	Loss
DEL00016191	chr2	54,337,977	54,338,053	76	Loss
DEL00016192	chr2	54,351,004	54,351,057	53	Loss
DEL00016197	chr2	54,388,398	54,391,192	2,794	Loss
DEL00016199	chr2	54,411,272	54,411,622	350	Loss
DUP00016200	chr2	54,415,966	54,416,159	193	Gain
DEL00016211	chr2	54,487,884	54,488,421	537	Loss
DEL00016215	chr2	54,547,956	54,548,516	560	Loss
DEL00016223	chr2	54,698,019	54,699,823	1,804	Loss
DEL00016232	chr2	54,835,199	54,836,690	1,491	Loss
DEL00016233	chr2	54,862,668	54,862,896	228	Loss
DEL00016235	chr2	54,872,350	54,872,863	513	Loss
DEL00016256	chr2	55,102,917	55,102,983	66	Loss
DEL00016262	chr2	55,113,536	55,114,082	546	Loss
DEL00016268	chr2	55,150,867	55,151,720	853	Loss
DEL00016274	chr2	55,160,152	55,160,485	333	Loss
DEL00016278	chr2	55,210,669	55,210,861	192	Loss
DEL00016279	chr2	55,217,869	55,219,408	1,539	Loss
DEL00016288	chr2	55,303,284	55,303,469	185	Loss
DEL00016291	chr2	55,364,567	55,364,783	216	Loss
DEL00016294	chr2	55,404,976	55,406,397	1,421	Loss
DEL00016298	chr2	55,443,296	55,443,837	541	Loss
DEL00016301	chr2	55,474,771	55,474,873	102	Loss
DEL00016303	chr2	55,506,256	55,506,327	71	Loss
DEL00016307	chr2	55,522,862	55,522,917	55	Loss
DEL00016308	chr2	55,544,770	55,544,898	128	Loss
DEL00016310	chr2	55,572,975	55,573,155	180	Loss
DEL00016326	chr2	55,667,525	55,668,946	1,421	Loss
DEL00016328	chr2	55,682,565	55,682,641	76	Loss
DEL00016337	chr2	55,730,271	55,730,325	54	Loss

DEL00016341	chr2	55,812,008	55,812,159	151	Loss
DEL00016344	chr2	55,827,451	55,827,869	418	Loss
DEL00016347	chr2	55,879,443	55,881,013	1,570	Loss
DEL00016350	chr2	55,897,683	55,897,870	187	Loss
DEL00016351	chr2	55,954,296	55,954,368	72	Loss
DEL00016353	chr2	55,961,475	55,961,544	69	Loss
DEL00016356	chr2	56,058,920	56,059,066	146	Loss
DUP00016378	chr2	56,192,159	56,202,225	10,066	Gain
DEL00016380	chr2	56,213,010	56,214,506	1,496	Loss
DEL00016385	chr2	56,268,571	56,268,987	416	Loss
DEL00016387	chr2	56,288,771	56,288,878	107	Loss
DEL00016388	chr2	56,311,570	56,311,852	282	Loss
DEL00016400	chr2	56,418,273	56,418,487	214	Loss
DEL00016406	chr2	56,500,988	56,501,040	52	Loss
DEL00016407	chr2	56,542,042	56,542,346	304	Loss
DEL00016410	chr2	56,573,000	56,573,602	602	Loss
DEL00016424	chr2	56,836,114	56,836,172	58	Loss
DEL00016431	chr2	56,923,047	56,923,651	604	Loss
DEL00016435	chr2	57,099,740	57,099,883	143	Loss
DEL00016436	chr2	57,118,474	57,118,532	58	Loss
DEL00016438	chr2	57,133,947	57,135,227	1,280	Loss
DEL00016440	chr2	57,176,199	57,176,635	436	Loss
DEL00016443	chr2	57,222,677	57,223,136	459	Loss
DEL00016455	chr2	57,401,231	57,401,763	532	Loss
DEL00016458	chr2	57,498,712	57,498,877	165	Loss
DEL00016459	chr2	57,503,195	57,504,345	1,150	Loss
DEL00016462	chr2	57,586,516	57,586,623	107	Loss
DEL00016468	chr2	57,681,857	57,681,925	68	Loss
DEL00016469	chr2	57,687,712	57,687,782	70	Loss
DEL00016479	chr2	57,798,986	57,799,413	427	Loss
DEL00016482	chr2	57,806,064	57,806,218	154	Loss
DEL00016491	chr2	57,843,961	57,844,564	603	Loss
DEL00016502	chr2	57,929,298	57,931,741	2,443	Loss
DEL00016513	chr2	58,120,864	58,121,098	234	Loss
DEL00016518	chr2	58,191,628	58,191,682	54	Loss
DEL00016519	chr2	58,310,796	58,310,860	64	Loss
DEL00016521	chr2	58,325,513	58,326,257	744	Loss
DEL00016522	chr2	58,360,131	58,363,270	3,139	Loss
DEL00016523	chr2	58,390,231	58,391,901	1,670	Loss
DEL00016527	chr2	58,545,532	58,545,650	118	Loss
DEL00016532	chr2	58,648,689	58,648,868	179	Loss
DEL00016533	chr2	58,683,078	58,683,616	538	Loss
DEL00016541	chr2	58,782,727	58,784,035	1,308	Loss
DEL00016554	chr2	59,038,453	59,038,518	65	Loss
DEL00016556	chr2	59,044,677	59,046,096	1,419	Loss
DEL00016557	chr2	59,049,170	59,050,405	1,235	Loss
DEL00016558	chr2	59,052,138	59,053,348	1,210	Loss
DEL00016560	chr2	59,075,007	59,075,067	60	Loss
DEL00016561	chr2	59,102,296	59,103,254	958	Loss

DEL00016567	chr2	59,194,805	59,194,947	142	Loss
DEL00016569	chr2	59,233,548	59,233,620	72	Loss
DEL00016570	chr2	59,258,044	59,258,563	519	Loss
DEL00016573	chr2	59,276,529	59,276,768	239	Loss
DEL00016574	chr2	59,302,081	59,302,151	70	Loss
DEL00016580	chr2	59,377,572	59,377,690	118	Loss
DEL00016581	chr2	59,394,398	59,394,546	148	Loss
DEL00016590	chr2	59,579,771	59,579,847	76	Loss
DEL00016597	chr2	59,697,247	59,697,418	171	Loss
DEL00016606	chr2	59,907,024	59,907,412	388	Loss
DEL00016612	chr2	60,107,866	60,107,920	54	Loss
DEL00016613	chr2	60,113,411	60,113,527	116	Loss
DEL00016614	chr2	60,124,127	60,124,216	89	Loss
DEL00016619	chr2	60,284,957	60,286,662	1,705	Loss
DEL00016623	chr2	60,372,027	60,372,289	262	Loss
DEL00016629	chr2	60,479,855	60,479,958	103	Loss
DEL00016630	chr2	60,488,398	60,488,616	218	Loss
DEL00016642	chr2	60,708,358	60,708,409	51	Loss
DEL00016645	chr2	60,831,816	60,837,827	6,011	Loss
DEL00016646	chr2	60,849,023	60,849,198	175	Loss
DEL00016647	chr2	60,867,103	60,867,171	68	Loss
DEL00016652	chr2	60,953,219	60,953,649	430	Loss
DEL00016657	chr2	61,073,710	61,074,277	567	Loss
DEL00016659	chr2	61,089,646	61,089,698	52	Loss
DEL00016663	chr2	61,311,538	61,312,040	502	Loss
DEL00016675	chr2	61,450,013	61,450,711	698	Loss
DEL00016678	chr2	61,565,827	61,565,879	52	Loss
DEL00016682	chr2	61,704,248	61,705,798	1,550	Loss
DEL00016690	chr2	61,917,192	61,918,056	864	Loss
DEL00016697	chr2	62,016,579	62,017,119	540	Loss
DEL00016698	chr2	62,017,478	62,017,559	81	Loss
DEL00016701	chr2	62,114,101	62,114,417	316	Loss
DUP00016704	chr2	62,194,652	62,220,389	25,737	Gain
DEL00016708	chr2	62,263,550	62,263,805	255	Loss
DEL00016716	chr2	62,503,403	62,503,845	442	Loss
DEL00016720	chr2	62,524,146	62,524,231	85	Loss
DEL00016722	chr2	62,571,750	62,572,549	799	Loss
DEL00016724	chr2	62,593,193	62,593,924	731	Loss
DEL00016727	chr2	62,620,076	62,620,558	482	Loss
DEL00016736	chr2	62,952,553	62,952,725	172	Loss
DEL00016738	chr2	62,957,175	62,957,473	298	Loss
DEL00016741	chr2	63,021,918	63,022,115	197	Loss
DEL00016742	chr2	63,053,968	63,054,034	66	Loss
DEL00016745	chr2	63,114,322	63,114,547	225	Loss
DEL00016747	chr2	63,260,606	63,260,705	99	Loss
DEL00016750	chr2	63,273,446	63,273,497	51	Loss
DEL00016753	chr2	63,432,141	63,432,907	766	Loss
DEL00016754	chr2	63,452,734	63,452,792	58	Loss
DEL00016755	chr2	63,475,862	63,479,154	3,292	Loss

DEL00016757	chr2	63,549,439	63,550,398	959	Loss
DEL00016759	chr2	63,675,970	63,676,035	65	Loss
DEL00016762	chr2	63,696,452	63,696,644	192	Loss
DEL00016768	chr2	63,909,699	63,909,864	165	Loss
DEL00016769	chr2	63,925,947	63,927,034	1,087	Loss
DEL00016778	chr2	64,166,310	64,166,764	454	Loss
DEL00016781	chr2	64,198,304	64,198,528	224	Loss
DEL00016782	chr2	64,210,130	64,210,474	344	Loss
DEL00016788	chr2	64,434,401	64,434,466	65	Loss
DEL00016789	chr2	64,444,381	64,444,437	56	Loss
DEL00016796	chr2	64,617,902	64,617,964	62	Loss
DEL00016803	chr2	64,841,736	64,842,169	433	Loss
DEL00016808	chr2	64,920,994	64,921,110	116	Loss
DEL00016809	chr2	64,974,545	64,974,672	127	Loss
DEL00016816	chr2	64,993,950	64,994,300	350	Loss
DEL00016817	chr2	64,994,593	64,996,820	2,227	Loss
DEL00016839	chr2	65,257,688	65,257,771	83	Loss
DEL00016840	chr2	65,284,360	65,284,753	393	Loss
DEL00016845	chr2	65,341,955	65,342,018	63	Loss
DEL00016848	chr2	65,351,129	65,351,255	126	Loss
DEL00016851	chr2	65,376,089	65,376,487	398	Loss
DEL00016861	chr2	65,548,526	65,548,654	128	Loss
DEL00016863	chr2	65,592,281	65,593,775	1,494	Loss
DEL00016866	chr2	65,611,719	65,612,970	1,251	Loss
DEL00016867	chr2	65,644,513	65,644,800	287	Loss
DUP00016868	chr2	65,663,285	65,663,401	116	Gain
DEL00016869	chr2	65,699,335	65,699,415	80	Loss
DEL00016895	chr2	65,957,593	65,957,670	77	Loss
DEL00016900	chr2	66,074,811	66,074,934	123	Loss
DEL00016901	chr2	66,107,391	66,107,475	84	Loss
DEL00016902	chr2	66,115,418	66,115,710	292	Loss
DEL00016907	chr2	66,268,166	66,268,323	157	Loss
DEL00016908	chr2	66,287,139	66,287,266	127	Loss
DEL00016923	chr2	66,398,364	66,398,444	80	Loss
DEL00016924	chr2	66,408,142	66,408,211	69	Loss
DEL00016929	chr2	66,562,474	66,562,530	56	Loss
DEL00016931	chr2	66,610,349	66,610,408	59	Loss
DEL00016932	chr2	66,625,882	66,626,807	925	Loss
DEL00016939	chr2	66,758,720	66,758,810	90	Loss
DEL00016946	chr2	66,858,316	66,860,761	2,445	Loss
DEL00016950	chr2	66,912,069	66,912,234	165	Loss
DEL00016953	chr2	66,938,707	66,940,289	1,582	Loss
DEL00016957	chr2	67,024,892	67,024,948	56	Loss
DEL00016958	chr2	67,037,802	67,037,870	68	Loss
DEL00016961	chr2	67,176,030	67,176,089	59	Loss
DEL00016971	chr2	67,231,906	67,232,392	486	Loss
DEL00016973	chr2	67,328,361	67,328,843	482	Loss
DEL00016974	chr2	67,340,020	67,340,105	85	Loss
DEL00016978	chr2	67,360,558	67,360,742	184	Loss

DEL00016979	chr2	67,407,864	67,408,281	417	Loss
DEL00016980	chr2	67,433,745	67,433,958	213	Loss
DEL00016984	chr2	67,485,953	67,486,032	79	Loss
DEL00016989	chr2	67,519,322	67,519,472	150	Loss
DEL00016990	chr2	67,521,492	67,521,547	55	Loss
DEL00016998	chr2	67,625,885	67,625,950	65	Loss
DEL00017016	chr2	67,763,251	67,763,306	55	Loss
DEL00017026	chr2	67,909,798	67,910,035	237	Loss
DEL00017028	chr2	67,977,038	67,977,546	508	Loss
DEL00017033	chr2	68,049,111	68,049,162	51	Loss
DEL00017036	chr2	68,170,679	68,170,757	78	Loss
DUP00017037	chr2	68,178,493	68,178,619	126	Gain
DEL00017041	chr2	68,307,854	68,308,744	890	Loss
DEL00017045	chr2	68,351,448	68,351,637	189	Loss
DEL00017046	chr2	68,401,083	68,401,249	166	Loss
DEL00017047	chr2	68,472,091	68,472,212	121	Loss
DEL00017051	chr2	68,549,447	68,549,533	86	Loss
DEL00017053	chr2	68,590,573	68,590,696	123	Loss
DEL00017057	chr2	68,605,807	68,606,287	480	Loss
DEL00017059	chr2	68,646,618	68,648,189	1,571	Loss
DEL00017060	chr2	68,669,839	68,669,973	134	Loss
DUP00017062	chr2	68,767,150	68,767,318	168	Gain
DEL00017071	chr2	68,828,480	68,828,918	438	Loss
DEL00017073	chr2	68,954,486	68,955,095	609	Loss
DEL00017074	chr2	68,987,590	68,987,680	90	Loss
DEL00017076	chr2	68,993,766	68,993,981	215	Loss
DEL00017090	chr2	69,025,228	69,025,862	634	Loss
DEL00017121	chr2	69,040,412	69,046,934	6,522	Loss
DUP00017126	chr2	69,082,270	69,082,354	84	Gain
DEL00017128	chr2	69,145,790	69,146,634	844	Loss
DEL00017130	chr2	69,172,880	69,173,395	515	Loss
DEL00017137	chr2	69,315,515	69,315,922	407	Loss
DEL00017138	chr2	69,325,957	69,327,355	1,398	Loss
DEL00017140	chr2	69,354,679	69,354,962	283	Loss
DEL00017145	chr2	69,551,977	69,552,675	698	Loss
DEL00017151	chr2	69,639,072	69,639,659	587	Loss
DEL00017160	chr2	69,781,886	69,782,197	311	Loss
DEL00017161	chr2	69,839,318	69,840,998	1,680	Loss
DEL00017162	chr2	69,851,110	69,851,481	371	Loss
DEL00017165	chr2	69,893,391	69,897,538	4,147	Loss
DEL00017170	chr2	69,944,743	69,944,797	54	Loss
DEL00017172	chr2	69,951,506	69,951,647	141	Loss
DUP00017185	chr2	70,178,969	70,202,393	23,424	Gain
DEL00017187	chr2	70,235,285	70,235,775	490	Loss
DEL00017192	chr2	70,302,852	70,303,486	634	Loss
DUP00017199	chr2	70,398,315	70,398,522	207	Gain
DEL00017200	chr2	70,430,534	70,431,284	750	Loss
DEL00017204	chr2	70,471,761	70,472,809	1,048	Loss
DEL00017215	chr2	70,535,967	70,536,034	67	Loss

DEL00017216	chr2	70,630,194	70,630,711	517	Loss
DEL00017220	chr2	70,702,900	70,703,822	922	Loss
DEL00017221	chr2	70,712,702	70,712,757	55	Loss
DEL00017225	chr2	70,767,696	70,768,320	624	Loss
DUP00017227	chr2	70,773,785	70,785,802	12,017	Gain
DEL00017237	chr2	70,919,325	70,919,402	77	Loss
DEL00017238	chr2	70,939,243	70,939,313	70	Loss
DEL00017239	chr2	70,991,112	70,992,715	1,603	Loss
DEL00017243	chr2	71,067,275	71,068,485	1,210	Loss
DUP00017245	chr2	71,139,638	71,139,868	230	Gain
DEL00017247	chr2	71,211,799	71,211,928	129	Loss
DEL00017250	chr2	71,243,490	71,243,628	138	Loss
DEL00017255	chr2	71,343,015	71,343,245	230	Loss
DEL00017262	chr2	71,401,794	71,401,867	73	Loss
DEL00017264	chr2	71,445,585	71,445,642	57	Loss
DEL00017269	chr2	71,486,737	71,493,193	6,456	Loss
DEL00017274	chr2	71,579,358	71,579,509	151	Loss
DEL00017279	chr2	71,711,594	71,712,064	470	Loss
DEL00017286	chr2	71,835,953	71,836,009	56	Loss
DEL00017288	chr2	71,851,562	71,851,692	130	Loss
DEL00017289	chr2	71,869,196	71,869,415	219	Loss
DUP00017290	chr2	71,872,261	71,900,105	27,844	Mixed
DEL00017299	chr2	71,959,483	71,959,558	75	Loss
DEL00017301	chr2	71,971,097	71,971,225	128	Loss
DEL00017304	chr2	72,000,098	72,002,180	2,082	Loss
DEL00017317	chr2	72,202,674	72,203,414	740	Loss
DEL00017322	chr2	72,327,038	72,328,041	1,003	Loss
DEL00017323	chr2	72,331,733	72,331,941	208	Loss
DEL00017324	chr2	72,352,550	72,353,423	873	Loss
DEL00017326	chr2	72,411,390	72,412,508	1,118	Loss
DEL00017327	chr2	72,426,306	72,426,762	456	Loss
DEL00017339	chr2	72,502,392	72,503,618	1,226	Loss
DEL00017351	chr2	72,602,989	72,605,397	2,408	Loss
DEL00017354	chr2	72,636,772	72,636,843	71	Loss
DEL00017358	chr2	72,716,413	72,716,523	110	Loss
DEL00017368	chr2	72,855,780	72,856,251	471	Loss
DEL00017371	chr2	72,896,491	72,896,955	464	Loss
DEL00017377	chr2	73,025,083	73,026,868	1,785	Loss
DEL00017383	chr2	73,112,871	73,113,134	263	Loss
DUP00017386	chr2	73,171,704	73,171,891	187	Gain
DEL00017387	chr2	73,200,348	73,200,622	274	Loss
DEL00017388	chr2	73,239,808	73,242,253	2,445	Loss
DEL00017408	chr2	73,658,288	73,658,960	672	Loss
DEL00017411	chr2	73,760,002	73,760,321	319	Loss
DEL00017414	chr2	73,826,737	73,830,548	3,811	Loss
DEL00017422	chr2	74,012,978	74,019,392	6,414	Loss
DEL00017425	chr2	74,041,531	74,041,596	65	Loss
DEL00017430	chr2	74,076,488	74,077,033	545	Loss
DUP00017432	chr2	74,119,384	74,133,628	14,244	Gain

DEL00017434	chr2	74,228,927	74,234,146	5,219	Loss
DEL00017442	chr2	74,387,860	74,388,072	212	Loss
DEL00017444	chr2	74,395,764	74,396,203	439	Loss
DEL00017448	chr2	74,458,527	74,458,661	134	Loss
DEL00017449	chr2	74,466,365	74,469,214	2,849	Loss
DEL00017457	chr2	74,531,529	74,531,648	119	Loss
DEL00017459	chr2	74,536,901	74,536,966	65	Loss
DEL00017477	chr2	74,723,881	74,723,944	63	Loss
DEL00017484	chr2	74,933,163	74,933,452	289	Loss
DEL00017487	chr2	74,964,216	74,964,279	63	Loss
DEL00017489	chr2	74,999,712	75,000,993	1,281	Loss
DEL00017497	chr2	75,190,546	75,191,397	851	Loss
DEL00017498	chr2	75,198,138	75,198,202	64	Loss
DEL00017500	chr2	75,213,463	75,214,258	795	Loss
DEL00017501	chr2	75,226,773	75,228,177	1,404	Loss
DUP00017509	chr2	75,375,285	75,375,523	238	Gain
DEL00017512	chr2	75,485,946	75,486,115	169	Loss
DEL00017513	chr2	75,488,755	75,488,829	74	Loss
DEL00017514	chr2	75,494,703	75,497,501	2,798	Loss
DEL00017515	chr2	75,542,874	75,542,930	56	Loss
DUP00017517	chr2	75,582,810	75,582,934	124	Gain
DEL00017519	chr2	75,693,505	75,693,626	121	Loss
DEL00017527	chr2	75,882,845	75,882,998	153	Loss
DEL00017529	chr2	75,907,979	75,909,081	1,102	Loss
DEL00017530	chr2	75,913,703	75,913,858	155	Loss
DEL00017532	chr2	75,918,577	75,919,451	874	Loss
DEL00017534	chr2	75,950,340	75,950,523	183	Loss
DEL00017536	chr2	75,966,500	75,966,657	157	Loss
DEL00017537	chr2	75,967,386	75,969,780	2,394	Loss
DEL00017568	chr2	76,434,461	76,434,597	136	Loss
DEL00017575	chr2	76,597,875	76,598,068	193	Loss
DEL00017577	chr2	76,627,259	76,627,550	291	Loss
DEL00017578	chr2	76,627,953	76,628,143	190	Loss
DUP00017587	chr2	76,754,828	76,754,932	104	Gain
DEL00017590	chr2	76,804,366	76,805,593	1,227	Loss
DEL00017594	chr2	76,871,046	76,871,624	578	Loss
DEL00017596	chr2	76,885,958	76,886,119	161	Loss
DEL00017598	chr2	76,887,671	76,887,958	287	Loss
DEL00017599	chr2	76,893,700	76,893,939	239	Loss
DEL00017600	chr2	76,901,616	76,901,828	212	Loss
DEL00017603	chr2	76,948,688	76,948,817	129	Loss
DEL00017608	chr2	77,041,105	77,044,154	3,049	Loss
DEL00017609	chr2	77,069,361	77,073,616	4,255	Loss
DEL00017610	chr2	77,079,250	77,079,302	52	Loss
DEL00017612	chr2	77,095,414	77,095,897	483	Loss
DEL00017613	chr2	77,115,486	77,115,603	117	Loss
DEL00017615	chr2	77,138,848	77,140,688	1,840	Loss
DEL00017617	chr2	77,146,325	77,147,553	1,228	Loss
DEL00017619	chr2	77,153,823	77,154,033	210	Loss

DEL00017620	chr2	77,175,324	77,175,375	51	Loss
DEL00017621	chr2	77,176,050	77,176,794	744	Loss
DEL00017623	chr2	77,199,036	77,199,163	127	Loss
DEL00017628	chr2	77,252,520	77,252,834	314	Loss
DEL00017630	chr2	77,265,545	77,266,154	609	Loss
DEL00017631	chr2	77,283,041	77,283,134	93	Loss
DEL00017634	chr2	77,303,285	77,303,341	56	Loss
DEL00017636	chr2	77,311,785	77,311,838	53	Loss
DEL00017637	chr2	77,328,393	77,330,267	1,874	Loss
DEL00017639	chr2	77,356,016	77,356,097	81	Loss
DEL00017640	chr2	77,374,524	77,375,004	480	Loss
DEL00017642	chr2	77,400,862	77,402,278	1,416	Loss
DEL00017643	chr2	77,402,465	77,402,623	158	Loss
DEL00017646	chr2	77,479,950	77,480,084	134	Loss
DEL00017652	chr2	77,592,583	77,592,656	73	Loss
DEL00017653	chr2	77,598,653	77,598,849	196	Loss
DEL00017655	chr2	77,673,313	77,673,520	207	Loss
DEL00017659	chr2	77,815,383	77,815,435	52	Loss
DEL00017662	chr2	77,932,225	77,932,944	719	Loss
DEL00017669	chr2	78,177,964	78,178,234	270	Loss
DEL00017672	chr2	78,257,359	78,257,424	65	Loss
DEL00017674	chr2	78,269,418	78,269,479	61	Loss
DEL00017676	chr2	78,329,875	78,331,169	1,294	Loss
DEL00017679	chr2	78,341,318	78,341,912	594	Loss
DEL00017680	chr2	78,375,776	78,376,846	1,070	Loss
DEL00017683	chr2	78,482,501	78,485,317	2,816	Loss
DEL00017694	chr2	78,720,479	78,721,470	991	Loss
DUP00017695	chr2	78,723,239	78,733,524	10,285	Gain
DEL00017698	chr2	78,762,302	78,762,480	178	Loss
DEL00017702	chr2	78,807,749	78,807,800	51	Loss
DEL00017706	chr2	78,886,187	78,886,705	518	Loss
DEL00017709	chr2	78,901,263	78,901,322	59	Loss
DEL00017715	chr2	78,963,181	78,963,538	357	Loss
DEL00017723	chr2	79,049,397	79,049,496	99	Loss
DEL00017724	chr2	79,068,875	79,069,737	862	Loss
DEL00017728	chr2	79,094,826	79,098,689	3,863	Loss
DUP00017808	chr2	79,134,185	79,134,487	302	Gain
DEL00017814	chr2	79,258,629	79,261,866	3,237	Loss
DEL00017820	chr2	79,347,915	79,348,038	123	Loss
DEL00017826	chr2	79,441,080	79,441,431	351	Loss
DEL00017827	chr2	79,461,741	79,462,294	553	Loss
DEL00017835	chr2	79,637,270	79,637,441	171	Loss
DEL00017839	chr2	79,721,681	79,721,977	296	Loss
DEL00017842	chr2	79,778,453	79,778,934	481	Loss
DEL00017852	chr2	79,885,135	79,885,211	76	Loss
DEL00017860	chr2	79,999,952	80,000,122	170	Loss
DEL00017867	chr2	80,097,626	80,097,778	152	Loss
DEL00017871	chr2	80,132,080	80,132,732	652	Loss
DEL00017875	chr2	80,252,485	80,253,239	754	Loss

DEL00017883	chr2	80,438,979	80,439,762	783	Loss
DEL00017891	chr2	80,513,545	80,514,176	631	Loss
DEL00017895	chr2	80,549,050	80,549,102	52	Loss
DEL00017898	chr2	80,564,272	80,564,417	145	Loss
DEL00017899	chr2	80,567,222	80,567,319	97	Loss
DEL00017902	chr2	80,638,180	80,638,329	149	Loss
DEL00017908	chr2	80,748,229	80,748,839	610	Loss
DEL00017909	chr2	80,777,631	80,778,100	469	Loss
DEL00017913	chr2	80,806,470	80,806,533	63	Loss
DEL00017916	chr2	80,815,940	80,816,413	473	Loss
DEL00017935	chr2	81,063,786	81,063,845	59	Loss
DEL00017938	chr2	81,206,168	81,206,243	75	Loss
DEL00017948	chr2	81,380,277	81,380,463	186	Loss
DEL00017951	chr2	81,395,022	81,395,355	333	Loss
DEL00017961	chr2	81,641,776	81,642,949	1,173	Loss
DEL00017963	chr2	81,688,054	81,688,271	217	Loss
DEL00017964	chr2	81,710,064	81,710,928	864	Loss
DEL00017968	chr2	81,832,195	81,832,737	542	Loss
DEL00017969	chr2	81,913,073	81,913,995	922	Loss
DEL00017972	chr2	81,919,665	81,919,817	152	Loss
DEL00017976	chr2	82,023,914	82,024,306	392	Loss
DUP00017978	chr2	82,027,816	82,027,987	171	Gain
DEL00017979	chr2	82,030,800	82,031,732	932	Loss
DEL00017980	chr2	82,046,949	82,047,865	916	Loss
DEL00017981	chr2	82,063,107	82,069,312	6,205	Loss
DEL00017982	chr2	82,108,646	82,108,698	52	Loss
DEL00017983	chr2	82,160,433	82,160,507	74	Loss
DEL00017988	chr2	82,302,673	82,302,899	226	Loss
DEL00017990	chr2	82,339,088	82,339,461	373	Loss
DEL00017995	chr2	82,429,833	82,429,962	129	Loss
DEL00017998	chr2	82,634,094	82,634,500	406	Loss
DEL00018000	chr2	82,641,484	82,641,556	72	Loss
DEL00018007	chr2	82,769,897	82,770,377	480	Loss
DEL00018011	chr2	82,867,601	82,867,778	177	Loss
DEL00018012	chr2	82,875,424	82,876,600	1,176	Loss
DEL00018019	chr2	83,004,306	83,004,589	283	Loss
DEL00018024	chr2	83,118,565	83,118,731	166	Loss
DEL00018026	chr2	83,133,388	83,133,441	53	Loss
DEL00018030	chr2	83,274,485	83,274,732	247	Loss
DUP00018031	chr2	83,275,818	83,276,279	461	Gain
DEL00018035	chr2	83,414,143	83,414,848	705	Loss
DEL00018036	chr2	83,445,522	83,445,654	132	Loss
DEL00018038	chr2	83,477,524	83,477,766	242	Loss
DEL00018040	chr2	83,502,059	83,502,171	112	Loss
DEL00018041	chr2	83,530,830	83,531,097	267	Loss
DEL00018042	chr2	83,572,491	83,572,995	504	Loss
DEL00018048	chr2	83,613,372	83,613,432	60	Loss
DEL00018049	chr2	83,617,956	83,618,014	58	Loss
DEL00018050	chr2	83,714,793	83,714,844	51	Loss

DEL00018052	chr2	83,788,011	83,788,092	81	Loss
DEL00018059	chr2	84,037,552	84,037,639	87	Loss
DEL00018060	chr2	84,038,382	84,038,489	107	Loss
DEL00018067	chr2	84,113,058	84,132,235	19,177	Loss
DEL00018069	chr2	84,162,767	84,163,420	653	Loss
DEL00018074	chr2	84,201,903	84,202,670	767	Loss
DEL00018077	chr2	84,216,702	84,216,820	118	Loss
DEL00018080	chr2	84,255,848	84,255,902	54	Loss
DEL00018083	chr2	84,332,106	84,332,396	290	Loss
DEL00018084	chr2	84,366,993	84,367,303	310	Loss
DEL00018086	chr2	84,380,786	84,381,087	301	Loss
DEL00018089	chr2	84,441,019	84,441,248	229	Loss
DEL00018091	chr2	84,476,869	84,477,028	159	Loss
DEL00018099	chr2	84,711,284	84,711,344	60	Loss
DEL00018102	chr2	84,779,460	84,779,521	61	Loss
DEL00018104	chr2	84,844,883	84,845,310	427	Loss
DEL00018107	chr2	84,849,765	84,860,875	11,110	Mixed
DEL00018109	chr2	84,885,518	84,886,792	1,274	Loss
DEL00018111	chr2	84,899,097	84,899,366	269	Loss
DEL00018112	chr2	84,940,007	84,940,058	51	Loss
DEL00018113	chr2	84,993,722	84,994,202	480	Loss
DEL00018115	chr2	85,061,048	85,061,909	861	Loss
DEL00018117	chr2	85,069,573	85,070,631	1,058	Loss
DEL00018119	chr2	85,088,850	85,089,050	200	Loss
DEL00018121	chr2	85,093,406	85,093,465	59	Loss
DEL00018128	chr2	85,323,734	85,324,486	752	Loss
DEL00018130	chr2	85,441,918	85,442,074	156	Loss
DEL00018131	chr2	85,463,197	85,464,002	805	Loss
DEL00018132	chr2	85,464,726	85,465,894	1,168	Loss
DEL00018134	chr2	85,516,138	85,516,218	80	Loss
DEL00018138	chr2	85,618,292	85,618,349	57	Loss
DEL00018141	chr2	85,661,818	85,663,457	1,639	Loss
DEL00018146	chr2	85,742,683	85,742,821	138	Loss
DEL00018149	chr2	85,743,959	85,744,247	288	Loss
DUP00018158	chr2	85,853,155	85,856,647	3,492	Gain
DEL00018159	chr2	85,867,273	85,867,340	67	Loss
DEL00018170	chr2	85,923,627	85,926,671	3,044	Loss
DEL00018171	chr2	85,976,646	85,977,177	531	Loss
DEL00018173	chr2	86,011,622	86,012,398	776	Loss
DEL00018177	chr2	86,077,009	86,077,111	102	Loss
DEL00018184	chr2	86,150,992	86,151,048	56	Loss
DEL00018185	chr2	86,160,230	86,160,315	85	Loss
DEL00018186	chr2	86,191,738	86,191,887	149	Loss
DEL00018191	chr2	86,266,801	86,266,883	82	Loss
DEL00018193	chr2	86,353,130	86,355,868	2,738	Loss
DEL00018195	chr2	86,401,798	86,401,906	108	Loss
DEL00018200	chr2	86,449,635	86,453,315	3,680	Loss
DEL00018201	chr2	86,493,420	86,493,499	79	Loss
DEL00018207	chr2	86,716,982	86,717,324	342	Loss

DEL00018209	chr2	86,747,256	86,756,201	8,945	Loss
DEL00018211	chr2	86,818,669	86,819,110	441	Loss
DEL00018214	chr2	86,886,880	86,893,909	7,029	Loss
DEL00018224	chr2	87,317,211	87,318,878	1,667	Loss
DEL00018229	chr2	87,457,862	87,462,527	4,665	Loss
DEL00018231	chr2	87,487,518	87,487,833	315	Loss
DEL00018233	chr2	87,505,335	87,505,565	230	Loss
DEL00018234	chr2	87,519,441	87,519,565	124	Loss
DEL00018235	chr2	87,542,607	87,542,663	56	Loss
DEL00018239	chr2	87,654,184	87,654,562	378	Loss
DEL00018240	chr2	87,665,783	87,666,020	237	Loss
DEL00018241	chr2	87,687,475	87,688,443	968	Loss
DEL00018242	chr2	87,725,976	87,728,122	2,146	Loss
DEL00018243	chr2	87,799,913	87,800,028	115	Loss
DEL00018248	chr2	87,900,967	87,901,038	71	Loss
DEL00018254	chr2	87,971,861	87,972,147	286	Loss
DEL00018256	chr2	88,050,978	88,051,038	60	Loss
DEL00018263	chr2	88,169,796	88,169,863	67	Loss
DEL00018267	chr2	88,277,125	88,277,253	128	Loss
DEL00018268	chr2	88,304,087	88,304,606	519	Loss
DEL00018274	chr2	88,446,106	88,446,185	79	Loss
DEL00018283	chr2	88,543,670	88,544,101	431	Loss
DEL00018287	chr2	88,576,578	88,576,762	184	Loss
DEL00018288	chr2	88,576,984	88,577,091	107	Loss
DEL00018289	chr2	88,591,634	88,593,621	1,987	Loss
DEL00018292	chr2	88,649,307	88,649,763	456	Loss
DEL00018301	chr2	88,787,950	88,788,016	66	Loss
DEL00018302	chr2	88,795,890	88,795,944	54	Loss
DEL00018311	chr2	88,866,114	88,866,386	272	Loss
DEL00018312	chr2	88,884,834	88,885,324	490	Loss
DEL00018316	chr2	88,932,752	88,932,828	76	Loss
DEL00018317	chr2	89,001,537	89,002,594	1,057	Loss
DEL00018319	chr2	89,022,063	89,035,354	13,291	Loss
DEL00018321	chr2	89,111,590	89,111,673	83	Loss
DEL00018323	chr2	89,121,247	89,121,315	68	Loss
DEL00018327	chr2	89,213,170	89,213,430	260	Loss
DEL00018336	chr2	89,368,855	89,369,332	477	Loss
DEL00018338	chr2	89,370,456	89,370,784	328	Loss
DEL00018340	chr2	89,404,161	89,408,793	4,632	Loss
DEL00018347	chr2	89,662,143	89,662,319	176	Loss
DUP00018353	chr2	89,743,157	89,743,456	299	Gain
DEL00018358	chr2	89,765,313	89,765,561	248	Loss
DEL00018362	chr2	89,814,331	89,814,787	456	Loss
DEL00018367	chr2	89,891,493	89,892,768	1,275	Loss
DEL00018369	chr2	89,930,603	89,930,835	232	Loss
DEL00018370	chr2	89,940,211	89,940,293	82	Loss
DEL00018378	chr2	90,035,318	90,035,618	300	Loss
DEL00018386	chr2	90,119,401	90,119,466	65	Loss
DEL00018391	chr2	90,240,747	90,241,434	687	Loss

DEL00018402	chr2	90,368,504	90,368,808	304	Loss
DEL00018404	chr2	90,386,089	90,386,185	96	Loss
DEL00018409	chr2	90,461,814	90,462,850	1,036	Loss
DEL00018412	chr2	90,499,439	90,499,520	81	Loss
DEL00018416	chr2	90,558,797	90,558,892	95	Loss
DEL00018420	chr2	90,691,287	90,691,367	80	Loss
DEL00018422	chr2	90,695,759	90,696,161	402	Loss
DEL00018431	chr2	90,838,305	90,838,516	211	Loss
DEL00018434	chr2	90,881,291	90,881,373	82	Loss
DEL00018453	chr2	91,732,826	91,733,553	727	Loss
DEL00018454	chr2	91,740,555	91,741,337	782	Loss
DEL00018456	chr2	91,787,618	91,787,758	140	Loss
DEL00018459	chr2	91,857,184	91,857,329	145	Loss
DEL00018460	chr2	91,889,328	91,889,444	116	Loss
DEL00018463	chr2	91,936,355	91,937,212	857	Loss
DEL00018471	chr2	92,105,862	92,105,947	85	Loss
DEL00018477	chr2	92,273,137	92,273,239	102	Loss
DEL00018478	chr2	92,324,851	92,325,049	198	Loss
DEL00018482	chr2	92,515,685	92,516,467	782	Loss
DEL00018486	chr2	92,584,675	92,584,737	62	Loss
DEL00018490	chr2	92,591,622	92,592,066	444	Loss
DEL00018495	chr2	92,676,436	92,676,487	51	Loss
DEL00018496	chr2	92,678,827	92,678,902	75	Loss
DEL00018497	chr2	92,734,734	92,735,079	345	Loss
DEL00018499	chr2	92,739,520	92,740,008	488	Loss
DEL00018501	chr2	92,801,111	92,801,977	866	Loss
DEL00018502	chr2	92,806,398	92,806,526	128	Loss
DEL00018508	chr2	92,942,285	92,942,577	292	Loss
DEL00018514	chr2	92,986,352	92,986,766	414	Loss
DEL00018521	chr2	93,091,177	93,092,097	920	Loss
DEL00018533	chr2	93,204,092	93,204,170	78	Loss
DEL00018534	chr2	93,216,106	93,216,385	279	Loss
DEL00018535	chr2	93,230,834	93,238,247	7,413	Loss
DEL00018536	chr2	93,241,033	93,241,321	288	Loss
DEL00018537	chr2	93,316,652	93,318,028	1,376	Loss
DEL00018541	chr2	93,437,169	93,437,446	277	Loss
DEL00018543	chr2	93,509,214	93,509,998	784	Loss
DEL00018549	chr2	93,738,482	93,739,993	1,511	Loss
DEL00018550	chr2	93,775,234	93,775,349	115	Loss
DEL00018551	chr2	93,817,026	93,829,266	12,240	Loss
DEL00018552	chr2	93,838,192	93,838,783	591	Loss
DEL00018555	chr2	93,842,505	93,850,915	8,410	Loss
DEL00018557	chr2	93,857,375	93,857,467	92	Loss
DEL00018558	chr2	93,886,210	93,886,440	230	Loss
DEL00018561	chr2	93,889,550	93,889,831	281	Loss
DEL00018567	chr2	93,957,753	93,957,913	160	Loss
DEL00018568	chr2	93,978,371	93,978,432	61	Loss
DEL00018570	chr2	94,015,452	94,015,987	535	Loss
DEL00018573	chr2	94,100,882	94,101,011	129	Loss

DEL00018574	chr2	94,110,711	94,110,817	106	Loss
DEL00018575	chr2	94,124,118	94,125,082	964	Loss
DEL00018579	chr2	94,172,940	94,173,010	70	Loss
DEL00018580	chr2	94,183,676	94,184,166	490	Loss
DEL00018582	chr2	94,210,942	94,211,107	165	Loss
DEL00018584	chr2	94,214,930	94,215,114	184	Loss
DEL00018585	chr2	94,217,263	94,217,364	101	Loss
DEL00018592	chr2	94,259,148	94,260,532	1,384	Loss
DEL00018593	chr2	94,263,828	94,263,883	55	Loss
DEL00018596	chr2	94,299,406	94,299,460	54	Loss
DEL00018597	chr2	94,305,944	94,306,015	71	Loss
DUP00018608	chr2	94,423,596	94,423,729	133	Gain
DEL00018613	chr2	94,530,346	94,530,417	71	Loss
DEL00018614	chr2	94,541,277	94,541,343	66	Loss
DEL00018626	chr2	94,684,883	94,685,387	504	Loss
DUP00018629	chr2	94,705,561	94,718,586	13,025	Gain
DEL00018630	chr2	94,733,399	94,733,607	208	Loss
DEL00018632	chr2	94,780,997	94,781,440	443	Loss
DUP00018641	chr2	94,845,263	94,853,252	7,989	Gain
DEL00018646	chr2	94,877,144	94,877,307	163	Loss
DEL00018647	chr2	94,956,231	94,958,026	1,795	Loss
DUP00018648	chr2	94,959,021	94,959,233	212	Gain
DEL00018650	chr2	95,019,141	95,019,635	494	Loss
DEL00018651	chr2	95,020,458	95,021,378	920	Loss
DEL00018653	chr2	95,048,181	95,048,750	569	Loss
DEL00018668	chr2	95,415,244	95,415,435	191	Loss
DEL00018675	chr2	95,535,774	95,536,851	1,077	Loss
DEL00018677	chr2	95,567,092	95,567,413	321	Loss
DEL00018678	chr2	95,718,860	95,718,913	53	Loss
DEL00018683	chr2	95,926,055	95,926,128	73	Loss
DEL00018685	chr2	95,957,821	95,957,893	72	Loss
DEL00018693	chr2	96,075,863	96,083,412	7,549	Loss
DEL00018697	chr2	96,147,127	96,148,553	1,426	Loss
DEL00018700	chr2	96,171,773	96,171,853	80	Loss
DEL00018705	chr2	96,284,609	96,284,897	288	Loss
DEL00018708	chr2	96,350,754	96,350,997	243	Loss
DEL00018713	chr2	96,369,567	96,381,696	12,129	Mixed
DEL00018770	chr2	96,402,793	96,402,897	104	Loss
DEL00018777	chr2	96,434,191	96,434,859	668	Loss
DEL00018784	chr2	96,559,570	96,559,747	177	Loss
DEL00018785	chr2	96,560,639	96,560,779	140	Loss
DEL00018791	chr2	96,671,058	96,671,197	139	Loss
DEL00018794	chr2	96,731,175	96,731,254	79	Loss
DEL00018795	chr2	96,762,894	96,763,206	312	Loss
DEL00018798	chr2	96,791,480	96,792,339	859	Loss
DEL00018802	chr2	96,868,335	96,868,386	51	Loss
DEL00018809	chr2	96,965,243	96,966,105	862	Loss
DEL00018813	chr2	97,070,261	97,070,375	114	Loss
DEL00018815	chr2	97,111,533	97,111,604	71	Loss

DEL00018816	chr2	97,116,219	97,116,446	227	Loss
DEL00018819	chr2	97,138,871	97,138,946	75	Loss
DEL00018826	chr2	97,280,469	97,280,619	150	Loss
DEL00018836	chr2	97,508,479	97,508,747	268	Loss
DEL00018837	chr2	97,528,263	97,528,365	102	Loss
DEL00018838	chr2	97,541,500	97,541,579	79	Loss
DEL00018842	chr2	97,608,539	97,608,597	58	Loss
DEL00018843	chr2	97,656,966	97,657,207	241	Loss
DEL00018847	chr2	97,813,665	97,814,388	723	Loss
DEL00018849	chr2	97,853,158	97,860,349	7,191	Loss
DEL00018852	chr2	97,894,424	97,894,493	69	Loss
DEL00018863	chr2	98,060,604	98,061,331	727	Loss
DEL00018864	chr2	98,085,983	98,086,047	64	Loss
DEL00018865	chr2	98,091,001	98,091,068	67	Loss
DEL00018882	chr2	98,543,858	98,544,038	180	Loss
DEL00018887	chr2	98,622,299	98,622,355	56	Loss
DEL00018893	chr2	98,727,478	98,727,643	165	Loss
DEL00018898	chr2	98,905,657	98,906,149	492	Loss
DEL00018904	chr2	99,113,162	99,113,724	562	Loss
DEL00018909	chr2	99,215,271	99,215,468	197	Loss
DEL00018913	chr2	99,314,256	99,314,314	58	Loss
DEL00018921	chr2	99,644,193	99,644,680	487	Loss
DEL00018928	chr2	99,807,069	99,807,145	76	Loss
DEL00018929	chr2	99,834,748	99,834,910	162	Loss
DEL00018933	chr2	99,892,482	99,892,662	180	Loss
DEL00018944	chr2	100,229,442	100,229,849	407	Loss
DEL00018960	chr2	100,559,899	100,559,984	85	Loss
DEL00018963	chr2	100,608,651	100,609,132	481	Loss
DEL00018969	chr2	100,693,261	100,693,328	67	Loss
DEL00018974	chr2	100,826,319	100,826,421	102	Loss
DEL00018985	chr2	100,931,510	100,931,604	94	Loss
DEL00018992	chr2	101,101,779	101,101,840	61	Loss
DEL00018994	chr2	101,118,426	101,118,520	94	Loss
DEL00019009	chr2	101,221,188	101,221,274	86	Loss
DEL00019013	chr2	101,270,342	101,270,404	62	Loss
DEL00019024	chr2	101,342,853	101,343,349	496	Loss
DEL00019033	chr2	101,491,632	101,491,771	139	Loss
DEL00019036	chr2	101,492,117	101,492,611	494	Loss
DEL00019038	chr2	101,494,935	101,495,597	662	Loss
DEL00019041	chr2	101,528,887	101,528,963	76	Loss
DEL00019043	chr2	101,574,352	101,574,725	373	Loss
DEL00019044	chr2	101,576,435	101,576,539	104	Loss
DEL00019045	chr2	101,592,894	101,593,323	429	Loss
DEL00019075	chr2	102,048,611	102,049,095	484	Loss
DEL00019077	chr2	102,060,966	102,061,338	372	Loss
DEL00019080	chr2	102,091,977	102,092,085	108	Loss
DEL00019090	chr2	102,289,538	102,289,697	159	Loss
DEL00019092	chr2	102,299,872	102,300,219	347	Loss
DEL00019094	chr2	102,331,512	102,331,569	57	Loss

DEL00019103	chr2	102,442,658	102,446,419	3,761	Loss
DEL00019107	chr2	102,449,792	102,449,872	80	Loss
DEL00019109	chr2	102,481,435	102,481,580	145	Loss
DEL00019113	chr2	102,566,343	102,566,579	236	Loss
DEL00019118	chr2	102,640,419	102,640,710	291	Loss
DUP00019120	chr2	102,695,283	102,716,378	21,095	Gain
DEL00019124	chr2	102,821,342	102,821,841	499	Loss
DEL00019127	chr2	102,848,611	102,848,897	286	Loss
DEL00019130	chr2	102,860,716	102,861,078	362	Loss
DEL00019134	chr2	102,891,992	102,892,189	197	Loss
DEL00019141	chr2	103,026,795	103,026,890	95	Loss
DEL00019142	chr2	103,042,807	103,042,879	72	Loss
DEL00019145	chr2	103,070,043	103,070,384	341	Loss
DEL00019146	chr2	103,071,867	103,071,927	60	Loss
DEL00019147	chr2	103,075,848	103,076,102	254	Loss
DEL00019156	chr2	103,151,095	103,151,284	189	Loss
DEL00019167	chr2	103,342,079	103,342,399	320	Loss
DEL00019172	chr2	103,399,390	103,399,476	86	Loss
DEL00019182	chr2	103,876,547	103,876,611	64	Loss
DEL00019184	chr2	103,915,999	103,916,128	129	Loss
DEL00019188	chr2	104,123,834	104,124,407	573	Loss
DEL00019193	chr2	104,149,969	104,150,203	234	Loss
DEL00019194	chr2	104,188,764	104,189,340	576	Loss
DEL00019196	chr2	104,245,806	104,246,600	794	Loss
DEL00019197	chr2	104,333,911	104,334,015	104	Loss
DEL00019201	chr2	104,367,673	104,368,208	535	Loss
DEL00019203	chr2	104,402,402	104,402,896	494	Loss
DEL00019215	chr2	104,533,562	104,533,659	97	Loss
DEL00019216	chr2	104,543,224	104,543,282	58	Loss
DEL00019220	chr2	104,623,026	104,629,378	6,352	Loss
DEL00019221	chr2	104,708,325	104,708,493	168	Loss
DUP00019230	chr2	104,901,829	104,902,151	322	Gain
DEL00019234	chr2	104,974,100	104,974,190	90	Loss
DEL00019235	chr2	104,991,764	104,991,997	233	Loss
DEL00019243	chr2	105,065,186	105,065,238	52	Loss
DEL00019249	chr2	105,266,883	105,267,542	659	Loss
DEL00019253	chr2	105,445,042	105,445,595	553	Loss
DEL00019254	chr2	105,519,266	105,523,609	4,343	Loss
DEL00019256	chr2	105,608,746	105,610,498	1,752	Loss
DUP00019258	chr2	105,663,616	105,679,019	15,403	Gain
DEL00019261	chr2	105,726,497	105,726,911	414	Loss
DEL00019264	chr2	105,775,076	105,775,227	151	Loss
DEL00019266	chr2	105,814,798	105,815,283	485	Loss
DEL00019273	chr2	106,000,512	106,001,996	1,484	Loss
DEL00019274	chr2	106,005,155	106,005,579	424	Loss
DEL00019294	chr2	106,329,002	106,329,248	246	Loss
DEL00019298	chr2	106,441,482	106,441,570	88	Loss
DEL00019301	chr2	106,503,475	106,503,823	348	Loss
DEL00019303	chr2	106,556,803	106,556,918	115	Loss

DEL00019306	chr2	106,614,344	106,614,830	486	Loss
DEL00019310	chr2	106,748,133	106,748,456	323	Loss
DEL00019316	chr2	106,916,001	106,916,502	501	Loss
DEL00019321	chr2	107,173,247	107,173,435	188	Loss
DEL00019323	chr2	107,223,952	107,224,427	475	Loss
DEL00019340	chr2	107,475,836	107,475,893	57	Loss
DEL00019343	chr2	107,492,442	107,492,880	438	Loss
DEL00019346	chr2	107,536,164	107,536,670	506	Loss
DEL00019348	chr2	107,545,945	107,546,873	928	Loss
DEL00019352	chr2	107,589,306	107,589,366	60	Loss
DUP00019354	chr2	107,756,635	107,756,718	83	Gain
DEL00019355	chr2	107,763,475	107,763,529	54	Loss
DEL00019358	chr2	107,857,853	107,858,002	149	Loss
DEL00019359	chr2	107,863,580	107,863,809	229	Loss
DEL00019362	chr2	107,877,063	107,877,155	92	Loss
DEL00019372	chr2	108,097,156	108,098,544	1,388	Loss
DEL00019377	chr2	108,207,012	108,207,072	60	Loss
DEL00019389	chr2	108,421,346	108,421,511	165	Loss
DEL00019390	chr2	108,449,243	108,449,303	60	Loss
DEL00019404	chr2	108,675,180	108,675,634	454	Loss
DEL00019407	chr2	108,818,768	108,818,986	218	Loss
DEL00019408	chr2	108,822,269	108,823,658	1,389	Loss
DEL00019414	chr2	109,038,842	109,038,985	143	Loss
DEL00019415	chr2	109,062,886	109,063,129	243	Loss
DEL00019420	chr2	109,146,143	109,146,644	501	Loss
DEL00019422	chr2	109,219,602	109,219,845	243	Loss
DEL00019424	chr2	109,231,948	109,237,638	5,690	Loss
DEL00019436	chr2	109,407,423	109,407,556	133	Loss
DEL00019437	chr2	109,427,234	109,427,508	274	Loss
DEL00019442	chr2	109,479,448	109,479,553	105	Loss
DEL00019446	chr2	109,582,025	109,583,501	1,476	Loss
DEL00019447	chr2	109,626,073	109,626,138	65	Loss
DEL00019449	chr2	109,627,280	109,630,643	3,363	Loss
DEL00019453	chr2	109,761,591	109,761,734	143	Loss
DEL00019456	chr2	109,830,493	109,848,543	18,050	Loss
DEL00019463	chr2	109,911,887	109,913,525	1,638	Loss
DEL00019470	chr2	110,031,905	110,032,234	329	Loss
DEL00019489	chr2	110,278,988	110,279,073	85	Loss
DEL00019500	chr2	110,466,294	110,466,459	165	Loss
DEL00019501	chr2	110,526,961	110,527,114	153	Loss
DEL00019504	chr2	110,542,071	110,542,270	199	Loss
DEL00019505	chr2	110,551,715	110,551,855	140	Loss
DEL00019512	chr2	110,589,164	110,589,216	52	Loss
DEL00019513	chr2	110,620,034	110,621,645	1,611	Loss
DEL00019524	chr2	110,826,930	110,827,042	112	Loss
DEL00019531	chr2	110,881,026	110,882,227	1,201	Loss
DEL00019533	chr2	110,885,146	110,885,658	512	Loss
DUP00019539	chr2	111,057,207	111,057,352	145	Gain
DEL00019547	chr2	111,433,625	111,434,279	654	Loss

DEL00019551	chr2	111,453,957	111,454,260	303	Loss
DEL00019556	chr2	111,499,609	111,499,667	58	Loss
DEL00019558	chr2	111,537,172	111,537,708	536	Loss
DEL00019559	chr2	111,554,554	111,555,130	576	Loss
DEL00019561	chr2	111,626,815	111,628,281	1,466	Loss
DEL00019566	chr2	111,652,379	111,655,127	2,748	Loss
DEL00019567	chr2	111,712,936	111,713,076	140	Loss
DEL00019573	chr2	111,798,582	111,798,638	56	Loss
DEL00019575	chr2	111,812,889	111,849,562	36,673	Loss
DEL00019582	chr2	111,866,811	111,866,934	123	Loss
DEL00019587	chr2	111,997,928	111,998,078	150	Loss
DEL00019590	chr2	112,069,077	112,069,462	385	Loss
DEL00019600	chr2	112,129,073	112,129,260	187	Loss
DEL00019605	chr2	112,241,543	112,241,792	249	Loss
DEL00019609	chr2	112,498,716	112,498,796	80	Loss
DEL00019616	chr2	112,533,272	112,533,323	51	Loss
DEL00019627	chr2	112,769,266	112,770,138	872	Loss
DEL00019632	chr2	112,840,673	112,841,154	481	Loss
DEL00019633	chr2	112,887,071	112,888,148	1,077	Loss
DEL00019634	chr2	112,919,284	112,920,352	1,068	Loss
DEL00019635	chr2	112,922,098	112,922,178	80	Loss
DEL00019637	chr2	112,954,703	112,954,812	109	Loss
DEL00019642	chr2	113,073,618	113,074,321	703	Loss
DEL00019651	chr2	113,221,051	113,221,164	113	Loss
DEL00019652	chr2	113,227,120	113,227,184	64	Loss
DEL00019661	chr2	113,376,770	113,377,074	304	Loss
DEL00019662	chr2	113,423,254	113,423,382	128	Loss
DEL00019667	chr2	113,571,374	113,571,880	506	Loss
DEL00019668	chr2	113,613,222	113,613,628	406	Loss
DEL00019674	chr2	113,660,051	113,660,376	325	Loss
DEL00019675	chr2	113,687,223	113,687,434	211	Loss
DUP00019677	chr2	113,714,146	113,728,705	14,559	Mixed
DEL00019679	chr2	113,730,492	113,730,595	103	Loss
DEL00019682	chr2	113,776,891	113,777,485	594	Loss
DEL00019691	chr2	113,908,282	113,908,377	95	Loss
DEL00019695	chr2	114,001,879	114,002,164	285	Loss
DEL00019697	chr2	114,051,626	114,052,143	517	Loss
DEL00019701	chr2	114,088,984	114,089,521	537	Loss
DUP00019707	chr2	114,210,484	114,223,834	13,350	Gain
DEL00019715	chr2	114,538,572	114,538,711	139	Loss
DEL00019716	chr2	114,546,960	114,547,594	634	Loss
DEL00019720	chr2	114,595,616	114,595,744	128	Loss
DEL00019729	chr2	114,767,697	114,768,950	1,253	Loss
DUP00019744	chr2	115,009,726	115,012,775	3,049	Gain
DUP00019799	chr2	115,338,942	115,364,896	25,954	Gain
DEL00019805	chr2	115,408,235	115,408,421	186	Loss
DEL00019808	chr2	115,436,774	115,437,353	579	Loss
DEL00019810	chr2	115,447,427	115,447,698	271	Loss
DEL00019816	chr2	115,475,113	115,475,336	223	Loss

DEL00019819	chr2	115,593,877	115,593,964	87	Loss
DEL00019820	chr2	115,601,609	115,602,430	821	Loss
DEL00019821	chr2	115,626,641	115,626,698	57	Loss
DEL00019823	chr2	115,636,941	115,637,128	187	Loss
DEL00019828	chr2	115,665,510	115,666,046	536	Loss
DEL00019830	chr2	115,676,464	115,676,931	467	Loss
DEL00019835	chr2	115,703,608	115,703,838	230	Loss
DEL00019840	chr2	115,788,774	115,788,828	54	Loss
DEL00019841	chr2	115,818,509	115,818,563	54	Loss
DEL00019842	chr2	115,824,001	115,824,059	58	Loss
DEL00019844	chr2	115,855,496	115,855,832	336	Loss
DEL00019847	chr2	115,942,829	115,952,989	10,160	Loss
DEL00019850	chr2	115,978,910	115,979,664	754	Loss
DEL00019853	chr2	116,027,125	116,027,306	181	Loss
DEL00019854	chr2	116,039,082	116,039,319	237	Loss
DEL00019857	chr2	116,083,599	116,083,720	121	Loss
DUP00019861	chr2	116,200,967	116,214,187	13,220	Gain
DEL00019868	chr2	116,353,587	116,353,964	377	Loss
DEL00019873	chr2	116,475,142	116,475,610	468	Loss
DEL00019876	chr2	116,505,215	116,505,328	113	Loss
DEL00019877	chr2	116,516,292	116,517,502	1,210	Loss
DEL00019881	chr2	116,551,765	116,551,821	56	Loss
DEL00019883	chr2	116,563,510	116,563,653	143	Loss
DEL00019885	chr2	116,588,111	116,588,248	137	Loss
DEL00019889	chr2	116,679,235	116,679,371	136	Loss
DEL00019890	chr2	116,735,982	116,736,086	104	Loss
DEL00019893	chr2	116,825,859	116,825,932	73	Loss
DEL00019894	chr2	116,830,698	116,831,233	535	Loss
DEL00019897	chr2	116,866,182	116,866,252	70	Loss
DEL00019900	chr2	116,953,026	116,953,096	70	Loss
DEL00019907	chr2	117,241,781	117,242,369	588	Loss
DEL00019913	chr2	117,278,941	117,279,149	208	Loss
DEL00019914	chr2	117,279,387	117,279,490	103	Loss
DEL00019916	chr2	117,315,802	117,316,785	983	Loss
DEL00019917	chr2	117,325,610	117,327,493	1,883	Loss
DEL00019924	chr2	117,494,201	117,495,271	1,070	Loss
DEL00019926	chr2	117,561,231	117,561,363	132	Loss
DEL00019929	chr2	117,580,636	117,581,455	819	Loss
DEL00019930	chr2	117,591,541	117,591,691	150	Loss
DUP00019931	chr2	117,705,999	117,706,076	77	Gain
DEL00019932	chr2	117,709,484	117,709,538	54	Loss
DEL00019938	chr2	117,783,558	117,784,001	443	Loss
DEL00019942	chr2	117,795,186	117,795,530	344	Loss
DEL00019950	chr2	117,994,078	117,994,249	171	Loss
DEL00019953	chr2	118,012,707	118,013,896	1,189	Loss
DEL00019955	chr2	118,027,476	118,027,558	82	Loss
DEL00019962	chr2	118,115,819	118,115,879	60	Loss
DEL00019964	chr2	118,148,023	118,148,106	83	Loss
DEL00019966	chr2	118,250,643	118,250,758	115	Loss

DEL00019969	chr2	118,291,900	118,291,995	95	Loss
DEL00019970	chr2	118,317,385	118,317,501	116	Loss
DEL00019971	chr2	118,321,682	118,321,766	84	Loss
DEL00019974	chr2	118,458,387	118,459,298	911	Loss
DEL00019976	chr2	118,479,848	118,480,112	264	Loss
DEL00019979	chr2	118,542,038	118,542,529	491	Loss
DEL00019980	chr2	118,550,810	118,551,410	600	Loss
DEL00019984	chr2	118,590,504	118,590,852	348	Loss
DEL00019985	chr2	118,641,904	118,641,969	65	Loss
DEL00019988	chr2	118,662,909	118,663,592	683	Loss
DEL00019992	chr2	118,728,639	118,728,779	140	Loss
DEL00019994	chr2	118,733,555	118,733,643	88	Loss
DEL00019995	chr2	118,831,556	118,831,614	58	Loss
DEL00020000	chr2	118,974,073	118,978,542	4,469	Loss
DEL00020003	chr2	119,022,084	119,022,174	90	Loss
DEL00020007	chr2	119,139,052	119,139,838	786	Loss
DEL00020011	chr2	119,305,246	119,314,758	9,512	Loss
DEL00020019	chr2	119,475,171	119,475,365	194	Loss
DEL00020026	chr2	119,666,667	119,666,905	238	Loss
DEL00020028	chr2	119,678,920	119,679,052	132	Loss
DEL00020030	chr2	119,726,158	119,728,616	2,458	Loss
DEL00020042	chr2	119,821,639	119,822,087	448	Loss
DEL00020045	chr2	119,866,372	119,866,518	146	Loss
DEL00020060	chr2	120,256,771	120,257,102	331	Loss
DEL00020063	chr2	120,272,784	120,273,321	537	Loss
DEL00020075	chr2	120,476,810	120,477,049	239	Loss
DEL00020090	chr2	120,621,403	120,621,454	51	Loss
DEL00020106	chr2	120,777,135	120,777,210	75	Loss
DEL00020127	chr2	120,958,930	120,961,257	2,327	Loss
DEL00020129	chr2	120,973,160	120,973,375	215	Loss
DEL00020140	chr2	121,180,559	121,180,625	66	Loss
DEL00020146	chr2	121,245,139	121,246,130	991	Loss
DEL00020147	chr2	121,278,073	121,278,320	247	Loss
DEL00020150	chr2	121,342,673	121,342,953	280	Loss
DEL00020153	chr2	121,361,237	121,361,360	123	Loss
DEL00020154	chr2	121,384,585	121,385,070	485	Loss
DEL00020155	chr2	121,388,703	121,388,867	164	Loss
DEL00020164	chr2	121,560,986	121,561,516	530	Loss
DEL00020167	chr2	121,585,201	121,585,693	492	Loss
DEL00020172	chr2	121,640,981	121,641,074	93	Loss
DEL00020180	chr2	121,818,544	121,818,603	59	Loss
DEL00020181	chr2	121,871,163	121,871,240	77	Loss
DEL00020186	chr2	121,964,813	121,964,885	72	Loss
DEL00020188	chr2	122,007,499	122,007,761	262	Loss
DEL00020190	chr2	122,054,055	122,054,398	343	Loss
DEL00020200	chr2	122,187,542	122,188,465	923	Loss
DEL00020203	chr2	122,285,756	122,286,554	798	Loss
DEL00020207	chr2	122,346,323	122,346,548	225	Loss
DEL00020210	chr2	122,379,679	122,379,746	67	Loss

DEL00020211	chr2	122,403,065	122,403,217	152	Loss
DEL00020212	chr2	122,427,223	122,427,274	51	Loss
DEL00020219	chr2	122,549,008	122,549,436	428	Loss
DUP00020225	chr2	122,612,722	122,612,796	74	Gain
DEL00020231	chr2	122,642,929	122,643,108	179	Loss
DEL00020232	chr2	122,653,615	122,654,442	827	Loss
DEL00020236	chr2	122,693,188	122,693,423	235	Loss
DEL00020238	chr2	122,736,175	122,736,392	217	Loss
DEL00020244	chr2	122,907,705	122,907,776	71	Loss
DEL00020245	chr2	122,912,268	122,912,432	164	Loss
DEL00020249	chr2	122,990,824	122,990,903	79	Loss
DEL00020250	chr2	123,085,547	123,085,678	131	Loss
DEL00020259	chr2	123,204,965	123,205,060	95	Loss
DEL00020260	chr2	123,216,875	123,217,416	541	Loss
DEL00020264	chr2	123,249,722	123,250,177	455	Loss
DEL00020267	chr2	123,316,485	123,316,537	52	Loss
DEL00020270	chr2	123,366,830	123,367,136	306	Loss
DEL00020271	chr2	123,401,156	123,401,236	80	Loss
DEL00020272	chr2	123,403,799	123,405,055	1,256	Loss
DEL00020274	chr2	123,489,064	123,490,074	1,010	Loss
DEL00020277	chr2	123,612,554	123,612,900	346	Loss
DEL00020279	chr2	123,654,337	123,654,402	65	Loss
DEL00020283	chr2	123,697,105	123,698,154	1,049	Loss
DEL00020284	chr2	123,703,576	123,704,498	922	Loss
DEL00020292	chr2	123,803,527	123,803,987	460	Loss
DEL00020295	chr2	123,804,346	123,805,024	678	Loss
DEL00020299	chr2	123,874,720	123,874,779	59	Loss
DEL00020308	chr2	124,030,201	124,030,553	352	Loss
DEL00020312	chr2	124,086,110	124,086,583	473	Loss
DEL00020314	chr2	124,102,970	124,103,263	293	Loss
DEL00020318	chr2	124,120,855	124,121,289	434	Loss
DEL00020325	chr2	124,146,396	124,146,706	310	Loss
DEL00020331	chr2	124,199,563	124,205,754	6,191	Loss
DEL00020334	chr2	124,245,810	124,246,323	513	Loss
DEL00020341	chr2	124,332,187	124,332,720	533	Loss
DEL00020344	chr2	124,373,473	124,373,703	230	Loss
DEL00020352	chr2	124,525,911	124,526,604	693	Loss
DEL00020365	chr2	124,690,767	124,690,818	51	Loss
DEL00020369	chr2	124,723,395	124,723,577	182	Loss
DEL00020371	chr2	124,733,459	124,733,575	116	Loss
DEL00020379	chr2	124,802,163	124,802,827	664	Loss
DEL00020381	chr2	124,821,683	124,822,159	476	Loss
DEL00020386	chr2	124,886,338	124,887,437	1,099	Loss
DEL00020396	chr2	124,982,814	124,983,310	496	Loss
DEL00020400	chr2	125,082,416	125,082,507	91	Loss
DEL00020406	chr2	125,192,112	125,192,183	71	Loss
DEL00020416	chr2	125,356,469	125,356,533	64	Loss
DEL00020417	chr2	125,366,759	125,366,992	233	Loss
DEL00020418	chr2	125,390,714	125,391,293	579	Loss

DEL00020419	chr2	125,406,274	125,406,329	55	Loss
DEL00020433	chr2	125,630,840	125,630,941	101	Loss
DEL00020434	chr2	125,669,322	125,669,402	80	Loss
DEL00020435	chr2	125,682,721	125,683,291	570	Loss
DEL00020445	chr2	125,808,015	125,808,081	66	Loss
DEL00020452	chr2	125,874,079	125,874,137	58	Loss
DEL00020461	chr2	125,976,386	125,978,025	1,639	Loss
DEL00020464	chr2	125,984,366	125,984,430	64	Loss
DEL00020466	chr2	126,040,396	126,040,862	466	Loss
DEL00020467	chr2	126,045,605	126,048,269	2,664	Loss
DEL00020472	chr2	126,090,213	126,090,292	79	Loss
DEL00020473	chr2	126,130,121	126,130,909	788	Loss
DEL00020479	chr2	126,348,393	126,348,538	145	Loss
DEL00020486	chr2	126,399,777	126,399,911	134	Loss
DEL00020487	chr2	126,416,938	126,417,410	472	Loss
DEL00020498	chr2	126,512,013	126,512,287	274	Loss
DEL00020502	chr2	126,653,523	126,653,576	53	Loss
DEL00020507	chr2	126,702,911	126,703,118	207	Loss
DEL00020509	chr2	126,820,666	126,821,297	631	Loss
DEL00020511	chr2	126,821,874	126,821,938	64	Loss
DEL00020512	chr2	126,822,502	126,822,773	271	Loss
DEL00020513	chr2	126,827,791	126,827,862	71	Loss
DEL00020514	chr2	126,829,427	126,829,500	73	Loss
DEL00020526	chr2	126,929,745	126,929,991	246	Loss
DEL00020527	chr2	126,946,948	126,947,038	90	Loss
DEL00020531	chr2	126,993,411	126,993,503	92	Loss
DEL00020533	chr2	127,043,389	127,044,136	747	Loss
DEL00020535	chr2	127,051,459	127,051,994	535	Loss
DEL00020537	chr2	127,053,603	127,053,885	282	Loss
DEL00020545	chr2	127,120,468	127,120,579	111	Loss
DEL00020546	chr2	127,126,125	127,126,283	158	Loss
DEL00020552	chr2	127,169,776	127,169,827	51	Loss
DEL00020559	chr2	127,203,467	127,204,187	720	Loss
DEL00020561	chr2	127,240,632	127,241,491	859	Loss
DEL00020562	chr2	127,252,410	127,252,481	71	Loss
DEL00020574	chr2	127,349,508	127,350,795	1,287	Loss
DEL00020580	chr2	127,375,926	127,379,068	3,142	Loss
DEL00020581	chr2	127,386,339	127,386,427	88	Loss
DEL00020586	chr2	127,473,425	127,473,839	414	Loss
DEL00020587	chr2	127,475,903	127,476,433	530	Loss
DEL00020593	chr2	127,570,859	127,570,965	106	Loss
DEL00020594	chr2	127,586,116	127,586,402	286	Loss
DEL00020595	chr2	127,603,723	127,603,970	247	Loss
DEL00020597	chr2	127,626,761	127,626,836	75	Loss
DEL00020601	chr2	127,685,752	127,685,993	241	Loss
DEL00020603	chr2	127,735,970	127,736,066	96	Loss
DEL00020605	chr2	127,778,677	127,778,785	108	Loss
DEL00020606	chr2	127,819,695	127,819,761	66	Loss
DEL00020607	chr2	127,851,680	127,851,889	209	Loss

DEL00020612	chr2	127,901,049	127,901,132	83	Loss
DEL00020613	chr2	127,932,380	127,932,499	119	Loss
DEL00020615	chr2	127,944,146	127,944,831	685	Loss
DEL00020617	chr2	128,006,012	128,006,209	197	Loss
DEL00020623	chr2	128,098,474	128,098,938	464	Loss
DEL00020629	chr2	128,125,555	128,125,655	100	Loss
DEL00020630	chr2	128,126,231	128,126,743	512	Loss
DEL00020635	chr2	128,277,161	128,277,279	118	Loss
DEL00020651	chr2	128,554,367	128,554,820	453	Loss
DEL00020654	chr2	128,566,491	128,566,739	248	Loss
DEL00020658	chr2	128,669,822	128,669,957	135	Loss
DEL00020659	chr2	128,683,179	128,683,234	55	Loss
DEL00020670	chr2	128,845,217	128,845,346	129	Loss
DUP00020671	chr2	128,901,164	128,901,271	107	Gain
DEL00020679	chr2	129,105,290	129,105,413	123	Loss
DUP00020690	chr2	129,356,506	129,432,555	76,049	Gain
DEL00020697	chr2	129,616,799	129,616,997	198	Loss
DUP00020699	chr2	129,669,542	129,674,617	5,075	Gain
DEL00020700	chr2	129,769,771	129,769,833	62	Loss
DEL00020701	chr2	129,831,934	129,831,986	52	Loss
DEL00020704	chr2	129,866,232	129,866,441	209	Loss
DEL00020705	chr2	129,873,813	129,873,866	53	Loss
DEL00020708	chr2	129,917,902	129,918,048	146	Loss
DEL00020712	chr2	129,946,271	129,946,803	532	Loss
DEL00020715	chr2	129,947,248	129,948,301	1,053	Loss
DEL00020716	chr2	129,964,303	129,964,525	222	Loss
DEL00020724	chr2	130,039,217	130,039,530	313	Loss
DEL00020727	chr2	130,164,047	130,164,171	124	Loss
DEL00020733	chr2	130,326,388	130,332,261	5,873	Loss
DEL00020736	chr2	130,354,758	130,356,486	1,728	Loss
DEL00020738	chr2	130,379,331	130,379,421	90	Loss
DEL00020741	chr2	130,494,345	130,494,505	160	Loss
DEL00020749	chr2	130,702,482	130,702,631	149	Loss
DEL00020752	chr2	130,717,924	130,717,990	66	Loss
DEL00020754	chr2	130,767,780	130,768,154	374	Loss
DEL00020757	chr2	130,796,344	130,796,995	651	Loss
DEL00020758	chr2	130,827,830	130,829,329	1,499	Loss
DEL00020763	chr2	130,946,333	130,946,860	527	Loss
DEL00020765	chr2	130,960,661	130,960,895	234	Loss
DEL00020768	chr2	131,026,901	131,027,252	351	Loss
DEL00020771	chr2	131,034,979	131,035,431	452	Loss
DEL00020772	chr2	131,038,066	131,038,431	365	Loss
DEL00020779	chr2	131,151,675	131,152,348	673	Loss
DEL00020781	chr2	131,174,864	131,175,470	606	Loss
DEL00020785	chr2	131,242,389	131,242,500	111	Loss
DEL00020795	chr2	131,296,571	131,296,954	383	Loss
DEL00020798	chr2	131,349,032	131,349,386	354	Loss
DEL00020804	chr2	131,358,302	131,358,657	355	Loss
DEL00020807	chr2	131,392,725	131,393,046	321	Loss

DEL00020808	chr2	131,406,906	131,407,508	602	Loss
DEL00020810	chr2	131,415,534	131,415,755	221	Loss
DEL00020811	chr2	131,425,127	131,425,210	83	Loss
DEL00020813	chr2	131,440,621	131,440,679	58	Loss
DEL00020818	chr2	131,491,540	131,492,032	492	Loss
DEL00020822	chr2	131,513,130	131,513,208	78	Loss
DEL00020841	chr2	131,685,405	131,685,501	96	Loss
DEL00020842	chr2	131,688,200	131,688,382	182	Loss
DEL00020844	chr2	131,701,520	131,701,786	266	Loss
DEL00020846	chr2	131,774,473	131,774,779	306	Loss
DEL00020849	chr2	131,788,357	131,788,495	138	Loss
DEL00020851	chr2	131,821,954	131,822,114	160	Loss
DEL00020854	chr2	131,877,111	131,877,299	188	Loss
DEL00020855	chr2	131,901,815	131,902,266	451	Loss
DEL00020859	chr2	131,987,173	131,987,276	103	Loss
DEL00020870	chr2	132,041,805	132,041,944	139	Loss
DEL00020882	chr2	132,123,431	132,123,487	56	Loss
DEL00020885	chr2	132,161,730	132,161,842	112	Loss
DEL00020888	chr2	132,234,241	132,234,801	560	Loss
DEL00020890	chr2	132,240,209	132,240,495	286	Loss
DEL00020892	chr2	132,295,008	132,295,066	58	Loss
DEL00020894	chr2	132,317,578	132,317,661	83	Loss
DUP00020899	chr2	132,423,896	132,424,052	156	Gain
DEL00020900	chr2	132,434,916	132,435,479	563	Loss
DEL00020905	chr2	132,513,841	132,514,155	314	Loss
DEL00020909	chr2	132,540,390	132,541,351	961	Loss
DEL00020910	chr2	132,549,639	132,553,159	3,520	Loss
DEL00020914	chr2	132,602,547	132,602,600	53	Loss
DEL00020917	chr2	132,687,825	132,689,069	1,244	Loss
DEL00020918	chr2	132,698,076	132,698,134	58	Loss
DEL00020919	chr2	132,698,831	132,698,900	69	Loss
DEL00020929	chr2	132,760,616	132,760,701	85	Loss
DEL00020930	chr2	132,777,310	132,777,478	168	Loss
DEL00020931	chr2	132,794,508	132,795,454	946	Loss
DEL00020932	chr2	132,796,526	132,803,173	6,647	Loss
DEL00020935	chr2	132,804,872	132,805,606	734	Loss
DEL00020936	chr2	132,861,818	132,862,572	754	Loss
DEL00020939	chr2	132,904,643	132,904,816	173	Loss
DUP00020940	chr2	132,930,862	132,931,302	440	Gain
DEL00020947	chr2	133,004,939	133,005,081	142	Loss
DEL00020948	chr2	133,028,362	133,029,165	803	Loss
DEL00020950	chr2	133,051,396	133,059,530	8,134	Loss
DEL00020953	chr2	133,143,809	133,144,122	313	Loss
DEL00020954	chr2	133,148,804	133,149,589	785	Loss
DEL00020955	chr2	133,181,688	133,184,031	2,343	Loss
DEL00020957	chr2	133,379,495	133,379,619	124	Loss
DEL00020961	chr2	133,420,157	133,420,964	807	Loss
DEL00020973	chr2	133,562,031	133,562,264	233	Loss
DEL00020974	chr2	133,589,905	133,589,958	53	Loss

DEL00020980	chr2	133,730,171	133,731,728	1,557	Loss
DEL00020981	chr2	133,737,438	133,742,461	5,023	Loss
DEL00020984	chr2	133,757,369	133,757,520	151	Loss
DEL00020989	chr2	133,818,991	133,819,157	166	Loss
DEL00020997	chr2	133,857,991	133,858,622	631	Loss
DEL00021000	chr2	133,873,904	133,874,691	787	Loss
DEL00021006	chr2	133,983,310	133,983,537	227	Loss
DEL00021009	chr2	133,993,641	133,993,742	101	Loss
DEL00021014	chr2	134,036,738	134,037,143	405	Loss
DEL00021015	chr2	134,047,789	134,047,888	99	Loss
DEL00021016	chr2	134,053,196	134,054,506	1,310	Loss
DEL00021017	chr2	134,064,206	134,065,267	1,061	Loss
DEL00021018	chr2	134,075,467	134,075,805	338	Loss
DEL00021020	chr2	134,083,220	134,083,471	251	Loss
DEL00021021	chr2	134,086,223	134,087,252	1,029	Loss
DEL00021024	chr2	134,118,277	134,122,360	4,083	Loss
DEL00021025	chr2	134,130,616	134,132,490	1,874	Loss
DEL00021028	chr2	134,138,009	134,141,321	3,312	Loss
DEL00021029	chr2	134,144,753	134,144,805	52	Loss
DEL00021030	chr2	134,156,682	134,156,749	67	Loss
DEL00021033	chr2	134,177,815	134,177,956	141	Loss
DEL00021051	chr2	134,668,430	134,668,897	467	Loss
DEL00021054	chr2	134,744,462	134,745,406	944	Loss
DEL00021079	chr2	135,234,632	135,234,881	249	Loss
DEL00021083	chr2	135,255,518	135,255,662	144	Loss
DEL00021098	chr2	135,620,839	135,620,970	131	Loss
DEL00021101	chr2	135,692,010	135,692,565	555	Loss
DEL00021104	chr2	135,728,844	135,728,950	106	Loss
DEL00021106	chr2	135,771,364	135,771,611	247	Loss
DEL00021110	chr2	135,868,548	135,868,786	238	Loss
DEL00021111	chr2	135,877,754	135,877,916	162	Loss
DEL00021116	chr2	136,056,611	136,063,968	7,357	Loss
DEL00021124	chr2	136,196,520	136,197,075	555	Loss
DEL00021125	chr2	136,202,108	136,202,246	138	Loss
DEL00021132	chr2	136,287,413	136,287,620	207	Loss
DEL00021136	chr2	136,388,466	136,389,184	718	Loss
DEL00021139	chr2	136,391,898	136,392,203	305	Loss
DEL00021145	chr2	136,409,634	136,410,179	545	Loss
DEL00021148	chr2	136,509,153	136,510,222	1,069	Loss
DEL00021149	chr2	136,614,573	136,614,918	345	Loss
DEL00021154	chr2	136,657,877	136,658,185	308	Loss
DEL00021155	chr2	136,664,801	136,664,874	73	Loss
DEL00021167	chr2	136,774,655	136,775,765	1,110	Loss
DEL00021173	chr2	136,841,118	136,841,175	57	Loss
DEL00021178	chr2	136,897,109	136,897,289	180	Loss
DEL00021183	chr2	137,025,658	137,025,780	122	Loss
DEL00021184	chr2	137,050,347	137,050,599	252	Loss
DEL00021190	chr2	137,237,915	137,238,421	506	Loss
DEL00021202	chr2	137,538,627	137,538,695	68	Loss

DEL00021204	chr2	137,540,035	137,540,963	928	Loss
DEL00021216	chr2	137,652,278	137,652,373	95	Loss
DEL00021222	chr2	137,749,596	137,752,512	2,916	Loss
DEL00021224	chr2	137,783,953	137,784,710	757	Loss
DUP00021226	chr2	137,813,436	137,813,503	67	Gain
DEL00021238	chr2	138,048,774	138,048,834	60	Loss
DEL00021245	chr2	138,120,522	138,120,708	186	Loss
DEL00021251	chr2	138,135,533	138,135,983	450	Loss
DEL00021254	chr2	138,138,329	138,139,188	859	Loss
DEL00021256	chr2	138,150,141	138,150,279	138	Loss
DEL00021260	chr2	138,232,862	138,233,003	141	Loss
DUP00021267	chr2	138,296,796	138,313,031	16,235	Gain
DEL00021271	chr2	138,364,945	138,365,161	216	Loss
DEL00021274	chr2	138,412,064	138,412,286	222	Loss
DEL00021275	chr2	138,414,520	138,416,536	2,016	Loss
DEL00021277	chr2	138,440,200	138,440,757	557	Loss
DEL00021279	chr2	138,457,826	138,458,578	752	Loss
DEL00021281	chr2	138,491,093	138,491,234	141	Loss
DEL00021282	chr2	138,508,352	138,508,723	371	Loss
DEL00021295	chr2	138,612,112	138,613,028	916	Loss
DEL00021301	chr2	138,758,797	138,758,922	125	Loss
DEL00021307	chr2	138,799,862	138,802,454	2,592	Loss
DEL00021308	chr2	138,825,001	138,825,693	692	Loss
DEL00021311	chr2	138,926,554	138,926,617	63	Loss
DEL00021314	chr2	138,956,095	138,956,262	167	Loss
DEL00021316	chr2	138,966,679	138,966,844	165	Loss
DEL00021335	chr2	139,407,414	139,407,599	185	Loss
DEL00021336	chr2	139,443,912	139,444,328	416	Loss
DEL00021339	chr2	139,528,936	139,529,243	307	Loss
DEL00021346	chr2	139,590,287	139,592,122	1,835	Loss
DEL00021362	chr2	139,764,759	139,764,817	58	Loss
DEL00021374	chr2	139,884,285	139,886,244	1,959	Loss
DEL00021377	chr2	139,915,401	139,915,553	152	Loss
DEL00021381	chr2	139,943,411	139,944,078	667	Loss
DEL00021382	chr2	139,945,400	139,945,461	61	Loss
DEL00021383	chr2	140,001,325	140,001,428	103	Loss
DEL00021386	chr2	140,073,414	140,074,579	1,165	Loss
DEL00021388	chr2	140,138,584	140,139,200	616	Loss
DEL00021398	chr2	140,398,078	140,398,584	506	Loss
DEL00021401	chr2	140,418,267	140,418,451	184	Loss
DEL00021402	chr2	140,428,036	140,428,267	231	Loss
DEL00021407	chr2	140,508,278	140,508,780	502	Loss
DEL00021411	chr2	140,526,454	140,526,511	57	Loss
DEL00021412	chr2	140,531,001	140,531,080	79	Loss
DEL00021417	chr2	140,594,944	140,595,009	65	Loss
DEL00021422	chr2	140,680,504	140,687,816	7,312	Loss
DEL00021424	chr2	140,722,047	140,722,102	55	Loss
DEL00021427	chr2	140,814,046	140,814,114	68	Loss
DEL00021436	chr2	141,023,050	141,023,176	126	Loss

DEL00021437	chr2	141,034,234	141,040,066	5,832	Loss
DEL00021438	chr2	141,091,617	141,091,691	74	Loss
DEL00021445	chr2	141,208,333	141,208,441	108	Loss
DEL00021457	chr2	141,409,890	141,410,365	475	Loss
DEL00021458	chr2	141,429,659	141,429,710	51	Loss
DEL00021466	chr2	141,515,289	141,515,391	102	Loss
DEL00021468	chr2	141,559,124	141,560,383	1,259	Loss
DEL00021469	chr2	141,581,706	141,581,765	59	Loss
DEL00021471	chr2	141,683,326	141,684,550	1,224	Loss
DUP00021479	chr2	141,810,266	141,815,013	4,747	Gain
DEL00021480	chr2	141,834,931	141,836,329	1,398	Loss
DEL00021481	chr2	141,841,981	141,842,120	139	Loss
DEL00021493	chr2	141,902,847	141,903,067	220	Loss
DEL00021497	chr2	142,077,187	142,077,338	151	Loss
DEL00021568	chr2	142,918,107	142,918,159	52	Loss
DEL00021585	chr2	143,156,129	143,156,198	69	Loss
DEL00021587	chr2	143,164,492	143,165,102	610	Loss
DEL00021590	chr2	143,189,882	143,196,068	6,186	Loss
DEL00021591	chr2	143,204,303	143,204,485	182	Loss
DEL00021594	chr2	143,318,362	143,318,437	75	Loss
DEL00021596	chr2	143,355,966	143,357,228	1,262	Loss
DEL00021599	chr2	143,394,106	143,394,455	349	Loss
DEL00021604	chr2	143,427,953	143,428,013	60	Loss
DEL00021605	chr2	143,429,708	143,429,766	58	Loss
DEL00021609	chr2	143,477,988	143,478,052	64	Loss
DEL00021611	chr2	143,527,938	143,528,858	920	Loss
DEL00021612	chr2	143,546,037	143,546,472	435	Loss
DEL00021615	chr2	143,601,861	143,603,062	1,201	Loss
DEL00021621	chr2	143,794,651	143,796,172	1,521	Loss
DUP00021622	chr2	143,823,640	143,823,827	187	Gain
DEL00021623	chr2	143,827,081	143,827,347	266	Loss
DEL00021634	chr2	144,025,043	144,025,610	567	Loss
DEL00021636	chr2	144,060,313	144,060,376	63	Loss
DEL00021637	chr2	144,070,101	144,070,290	189	Loss
DEL00021647	chr2	144,385,616	144,385,979	363	Loss
DEL00021648	chr2	144,387,425	144,388,903	1,478	Loss
DEL00021652	chr2	144,453,580	144,453,709	129	Loss
DEL00021655	chr2	144,506,332	144,506,448	116	Loss
DEL00021657	chr2	144,574,931	144,575,098	167	Loss
DEL00021658	chr2	144,586,784	144,586,859	75	Loss
DEL00021662	chr2	144,709,120	144,709,188	68	Loss
DEL00021673	chr2	144,784,418	144,784,543	125	Loss
DEL00021674	chr2	144,797,552	144,797,611	59	Loss
DEL00021688	chr2	144,942,194	144,942,334	140	Loss
DEL00021692	chr2	144,996,365	144,996,421	56	Loss
DEL00021693	chr2	145,022,448	145,022,548	100	Loss
DEL00021696	chr2	145,046,185	145,046,755	570	Loss
DEL00021705	chr2	145,229,438	145,229,619	181	Loss
DEL00021707	chr2	145,251,249	145,251,304	55	Loss

DEL00021711	chr2	145,272,807	145,273,250	443	Loss
DEL00021718	chr2	145,395,604	145,395,966	362	Loss
DEL00021720	chr2	145,396,475	145,396,731	256	Loss
DEL00021723	chr2	145,439,628	145,440,123	495	Loss
DUP00021726	chr2	145,497,949	145,498,040	91	Gain
DEL00021732	chr2	145,594,027	145,594,221	194	Loss
DEL00021733	chr2	145,695,975	145,696,034	59	Loss
DEL00021740	chr2	145,797,699	145,797,751	52	Loss
DEL00021752	chr2	145,943,034	145,943,178	144	Loss
DEL00021759	chr2	145,999,272	145,999,940	668	Loss
DEL00021775	chr2	146,133,053	146,133,542	489	Loss
DEL00021782	chr2	146,234,248	146,234,306	58	Loss
DEL00021797	chr2	146,466,195	146,466,276	81	Loss
DEL00021801	chr2	146,664,281	146,664,482	201	Loss
DEL00021811	chr2	146,850,270	146,850,328	58	Loss
DEL00021812	chr2	146,851,144	146,851,219	75	Loss
DEL00021814	chr2	146,870,774	146,871,078	304	Loss
DEL00021815	chr2	146,912,598	146,912,686	88	Loss
DEL00021817	chr2	146,929,798	146,930,345	547	Loss
DEL00021821	chr2	146,991,872	146,992,134	262	Loss
DEL00021822	chr2	146,997,463	146,997,594	131	Loss
DUP00021824	chr2	147,101,944	147,102,200	256	Gain
DEL00021846	chr2	147,409,047	147,409,287	240	Loss
DEL00021848	chr2	147,442,094	147,442,573	479	Loss
DEL00021856	chr2	147,605,063	147,605,155	92	Loss
DEL00021858	chr2	147,620,046	147,620,198	152	Loss
DEL00021860	chr2	147,646,283	147,646,389	106	Loss
DEL00021865	chr2	147,671,002	147,671,079	77	Loss
DEL00021866	chr2	147,674,491	147,675,648	1,157	Loss
DEL00021871	chr2	147,768,336	147,768,630	294	Loss
DEL00021872	chr2	147,800,307	147,800,790	483	Loss
DEL00021873	chr2	147,851,041	147,851,568	527	Loss
DEL00021880	chr2	148,068,960	148,069,070	110	Loss
DEL00021882	chr2	148,130,827	148,130,920	93	Loss
DEL00021887	chr2	148,182,733	148,183,284	551	Loss
DEL00021892	chr2	148,260,742	148,260,838	96	Loss
DEL00021894	chr2	148,270,230	148,270,334	104	Loss
DUP00021901	chr2	148,337,644	148,337,763	119	Gain
DUP00022187	chr2	148,433,437	148,460,474	27,037	Gain
DUP00022294	chr2	148,478,480	148,538,342	59,862	Gain
DUP00022426	chr2	148,607,865	148,608,540	675	Gain
DEL00022548	chr2	148,732,182	148,732,369	187	Loss
DEL00022550	chr2	148,751,159	148,751,319	160	Loss
DEL00022552	chr2	148,753,823	148,760,195	6,372	Loss
DEL00022562	chr2	148,772,634	148,773,156	522	Loss
DEL00022577	chr2	148,794,848	148,795,108	260	Loss
DEL00022609	chr2	148,840,089	148,840,670	581	Loss
DEL00022621	chr2	148,851,423	148,851,494	71	Loss
DEL00022626	chr2	148,859,080	148,859,473	393	Loss

DUP00022695	chr2	149,058,245	149,059,007	762	Gain
DUP00022700	chr2	149,059,089	149,059,675	586	Gain
DUP00022730	chr2	149,072,440	149,073,388	948	Mixed
DEL00022742	chr2	149,074,738	149,075,881	1,143	Mixed
DEL00022765	chr2	149,080,109	149,080,673	564	Loss
DUP00022771	chr2	149,083,242	149,083,449	207	Gain
DUP00022781	chr2	149,095,067	149,095,573	506	Mixed
DUP00022786	chr2	149,096,130	149,096,835	705	Gain
DUP00022798	chr2	149,101,073	149,101,550	477	Mixed
DEL00022813	chr2	149,109,285	149,109,418	133	Loss
DEL00022817	chr2	149,117,902	149,118,042	140	Loss
DEL00022818	chr2	149,118,629	149,118,703	74	Loss
DEL00022826	chr2	149,127,938	149,128,025	87	Loss
DEL00022827	chr2	149,128,177	149,128,576	399	Loss
DEL00022834	chr2	149,169,654	149,169,740	86	Loss
DUP00022836	chr2	149,172,106	149,172,993	887	Mixed
DUP00022842	chr2	149,179,482	149,179,957	475	Mixed
DUP00022862	chr2	149,181,162	149,182,247	1,085	Gain
DUP00023029	chr2	149,229,075	149,229,735	660	Gain
DUP00023087	chr2	149,234,023	149,234,233	210	Gain
DEL00023138	chr2	149,250,750	149,251,465	715	Loss
DEL00023180	chr2	149,278,061	149,278,500	439	Loss
DEL00023197	chr2	149,286,011	149,286,085	74	Loss
DEL00023216	chr2	149,290,499	149,291,327	828	Loss
DEL00023220	chr2	149,291,351	149,291,708	357	Loss
DEL00023241	chr2	149,315,848	149,316,462	614	Loss
DEL00023245	chr2	149,320,215	149,320,610	395	Loss
DEL00023265	chr2	149,326,943	149,327,435	492	Loss
DEL00023307	chr2	149,340,970	149,341,839	869	Loss
DUP00023332	chr2	149,346,672	149,347,234	562	Gain
DEL00023351	chr2	149,353,696	149,354,234	538	Loss
DUP00023386	chr2	149,372,433	149,372,879	446	Gain
DUP00023453	chr2	149,394,851	149,395,236	385	Mixed
DUP00023459	chr2	149,399,050	149,399,389	339	Gain
DEL00023462	chr2	149,399,921	149,400,081	160	Loss
DEL00023463	chr2	149,403,987	149,404,080	93	Loss
DEL00023528	chr2	149,419,434	149,420,111	677	Mixed
DEL00023539	chr2	149,425,533	149,425,694	161	Loss
DUP00023585	chr2	149,444,778	149,446,094	1,316	Mixed
DEL00023608	chr2	149,451,249	149,452,378	1,129	Loss
DUP00023718	chr2	149,487,964	149,488,252	288	Gain
DUP00023742	chr2	149,490,812	149,491,554	742	Gain
DEL00023804	chr2	149,504,864	149,505,518	654	Loss
DEL00023814	chr2	149,505,957	149,507,357	1,400	Mixed
DUP00023853	chr2	149,512,129	149,512,978	849	Gain
DEL00023908	chr2	149,524,414	149,525,626	1,212	Loss
DEL00023918	chr2	149,525,948	149,526,462	514	Loss
DEL00023952	chr2	149,530,333	149,530,520	187	Loss
DUP00023982	chr2	149,536,478	149,537,927	1,449	Mixed

DEL00024006	chr3	70,537	70,677	140	Loss
DEL00024008	chr3	73,090	79,265	6,175	Loss
DEL00024010	chr3	82,675	82,857	182	Loss
DEL00024012	chr3	119,667	119,722	55	Loss
DEL00024014	chr3	149,554	149,654	100	Loss
DEL00024016	chr3	171,862	174,579	2,717	Loss
DEL00024018	chr3	252,163	252,245	82	Loss
DEL00024020	chr3	277,345	277,815	470	Loss
DEL00024031	chr3	442,377	442,495	118	Loss
DEL00024039	chr3	547,793	548,112	319	Loss
DEL00024041	chr3	572,805	575,898	3,093	Loss
DEL00024045	chr3	608,805	611,305	2,500	Loss
DEL00024046	chr3	612,514	612,662	148	Loss
DEL00024047	chr3	627,746	629,022	1,276	Loss
DEL00024050	chr3	641,916	642,315	399	Loss
DUP00024069	chr3	1,014,959	1,015,019	60	Gain
DEL00024077	chr3	1,376,989	1,377,352	363	Loss
DEL00024081	chr3	1,533,397	1,533,824	427	Loss
DEL00024094	chr3	1,981,516	1,982,221	705	Loss
DEL00024102	chr3	2,374,411	2,374,474	63	Loss
DEL00024108	chr3	2,966,688	2,967,190	502	Loss
DEL00024113	chr3	3,095,708	3,095,867	159	Loss
DEL00024114	chr3	3,115,330	3,115,413	83	Loss
DEL00024120	chr3	3,206,847	3,207,535	688	Loss
DUP00024123	chr3	3,224,656	3,242,549	17,893	Gain
DEL00024140	chr3	3,389,225	3,389,409	184	Loss
DEL00024141	chr3	3,407,274	3,407,409	135	Loss
DEL00024150	chr3	3,523,295	3,523,575	280	Loss
DEL00024170	chr3	3,754,919	3,755,075	156	Loss
DEL00024185	chr3	4,042,806	4,042,979	173	Loss
DEL00024186	chr3	4,043,710	4,043,764	54	Loss
DEL00024192	chr3	4,105,398	4,105,971	573	Loss
DEL00024202	chr3	4,194,657	4,194,803	146	Loss
DEL00024215	chr3	4,335,240	4,336,262	1,022	Loss
DEL00024219	chr3	4,378,852	4,379,042	190	Loss
DEL00024222	chr3	4,413,719	4,413,770	51	Loss
DEL00024227	chr3	4,530,436	4,530,582	146	Loss
DEL00024243	chr3	4,740,019	4,740,454	435	Loss
DEL00024245	chr3	4,748,994	4,749,299	305	Loss
DEL00024249	chr3	4,768,842	4,769,377	535	Loss
DEL00024258	chr3	4,878,522	4,879,136	614	Loss
DEL00024265	chr3	4,945,501	4,945,808	307	Loss
DEL00024272	chr3	5,044,115	5,044,285	170	Loss
DEL00024275	chr3	5,150,074	5,150,187	113	Loss
DEL00024280	chr3	5,220,963	5,221,447	484	Loss
DEL00024281	chr3	5,228,243	5,229,301	1,058	Loss
DEL00024292	chr3	5,574,728	5,574,782	54	Loss
DEL00024304	chr3	5,768,353	5,768,443	90	Loss
DEL00024305	chr3	5,788,116	5,788,971	855	Loss

DEL00024307	chr3	5,826,731	5,827,300	569	Loss
DEL00024322	chr3	6,141,031	6,141,109	78	Loss
DEL00024333	chr3	6,246,512	6,247,271	759	Loss
DEL00024334	chr3	6,273,346	6,273,408	62	Loss
DEL00024335	chr3	6,303,197	6,304,153	956	Loss
DEL00024336	chr3	6,317,660	6,317,951	291	Loss
DEL00024338	chr3	6,372,508	6,372,893	385	Loss
DEL00024342	chr3	6,455,505	6,457,360	1,855	Loss
DEL00024348	chr3	6,558,626	6,559,636	1,010	Loss
DEL00024351	chr3	6,612,078	6,612,182	104	Loss
DEL00024352	chr3	6,628,153	6,628,369	216	Loss
DEL00024360	chr3	6,775,974	6,776,036	62	Loss
DEL00024367	chr3	6,887,456	6,887,651	195	Loss
DEL00024372	chr3	6,940,896	6,941,292	396	Loss
DEL00024378	chr3	7,029,736	7,029,829	93	Loss
DEL00024385	chr3	7,096,163	7,096,227	64	Loss
DEL00024392	chr3	7,152,817	7,152,905	88	Loss
DEL00024399	chr3	7,315,291	7,321,736	6,445	Loss
DEL00024414	chr3	7,688,426	7,688,629	203	Loss
DEL00024416	chr3	7,699,751	7,700,121	370	Loss
DEL00024417	chr3	7,777,097	7,777,152	55	Loss
DEL00024418	chr3	7,783,070	7,783,272	202	Loss
DEL00024420	chr3	7,810,444	7,810,650	206	Loss
DEL00024422	chr3	7,875,984	7,876,773	789	Loss
DEL00024424	chr3	7,921,008	7,921,635	627	Loss
DEL00024425	chr3	7,932,156	7,932,249	93	Loss
DEL00024427	chr3	7,951,767	7,952,057	290	Loss
DEL00024428	chr3	7,971,858	7,971,918	60	Loss
DEL00024437	chr3	8,129,502	8,130,662	1,160	Loss
DEL00024438	chr3	8,149,667	8,149,784	117	Loss
DEL00024448	chr3	8,324,947	8,325,002	55	Loss
DEL00024450	chr3	8,379,768	8,379,923	155	Loss
DEL00024452	chr3	8,452,121	8,452,300	179	Loss
DEL00024457	chr3	8,481,477	8,481,943	466	Loss
DEL00024464	chr3	8,567,284	8,567,382	98	Loss
DEL00024468	chr3	8,628,348	8,629,030	682	Loss
DEL00024472	chr3	8,662,266	8,662,512	246	Loss
DEL00024477	chr3	8,727,398	8,727,580	182	Loss
DEL00024478	chr3	8,730,163	8,730,363	200	Loss
DEL00024479	chr3	8,753,218	8,753,370	152	Loss
DEL00024483	chr3	8,845,550	8,845,973	423	Loss
DEL00024494	chr3	9,041,540	9,041,658	118	Loss
DEL00024496	chr3	9,054,085	9,054,156	71	Loss
DUP00024497	chr3	9,117,214	9,117,281	67	Gain
DEL00024499	chr3	9,155,307	9,155,523	216	Loss
DEL00024505	chr3	9,307,417	9,307,922	505	Loss
DEL00024507	chr3	9,389,259	9,389,450	191	Loss
DEL00024508	chr3	9,389,728	9,389,877	149	Loss
DEL00024514	chr3	9,506,414	9,506,717	303	Loss

DEL00024518	chr3	9,558,909	9,558,988	79	Loss
DEL00024519	chr3	9,576,680	9,576,751	71	Loss
DEL00024531	chr3	9,877,302	9,877,728	426	Loss
DEL00024534	chr3	9,952,728	9,952,845	117	Loss
DEL00024539	chr3	10,085,632	10,085,684	52	Loss
DEL00024540	chr3	10,101,882	10,102,390	508	Loss
DEL00024543	chr3	10,128,497	10,128,668	171	Loss
DEL00024546	chr3	10,175,019	10,175,144	125	Loss
DEL00024549	chr3	10,206,550	10,206,601	51	Loss
DEL00024560	chr3	10,484,072	10,484,188	116	Loss
DEL00024568	chr3	10,530,923	10,531,217	294	Loss
DEL00024571	chr3	10,593,908	10,594,390	482	Loss
DEL00024576	chr3	10,703,219	10,703,312	93	Loss
DEL00024587	chr3	11,224,278	11,224,613	335	Loss
DUP00024588	chr3	11,229,250	11,232,027	2,777	Gain
DEL00024590	chr3	11,374,958	11,375,027	69	Loss
DEL00024591	chr3	11,460,658	11,460,716	58	Loss
DEL00024593	chr3	11,491,950	11,492,017	67	Loss
DEL00024594	chr3	11,588,164	11,588,378	214	Loss
DEL00024596	chr3	11,657,176	11,657,671	495	Loss
DEL00024608	chr3	11,952,153	11,952,729	576	Loss
DEL00024609	chr3	11,954,539	11,954,623	84	Loss
DEL00024610	chr3	11,960,840	11,961,930	1,090	Loss
DEL00024642	chr3	12,218,002	12,218,059	57	Loss
DEL00024649	chr3	12,257,141	12,257,549	408	Loss
DEL00024655	chr3	12,529,228	12,529,761	533	Loss
DUP00024661	chr3	12,595,086	12,595,148	62	Gain
DEL00024670	chr3	12,755,661	12,755,871	210	Loss
DEL00024674	chr3	12,821,756	12,822,868	1,112	Loss
DEL00024682	chr3	12,998,043	12,998,303	260	Loss
DEL00024684	chr3	13,070,654	13,071,028	374	Loss
DEL00024689	chr3	13,114,164	13,114,236	72	Loss
DEL00024694	chr3	13,325,579	13,325,636	57	Loss
DEL00024697	chr3	13,495,990	13,496,171	181	Loss
DEL00024698	chr3	13,544,686	13,545,756	1,070	Loss
DEL00024699	chr3	13,552,090	13,552,950	860	Loss
DEL00024700	chr3	13,575,386	13,575,524	138	Loss
DEL00024701	chr3	13,595,510	13,595,585	75	Loss
DEL00024703	chr3	13,598,143	13,598,280	137	Loss
DEL00024712	chr3	13,943,064	13,943,293	229	Loss
DEL00024715	chr3	13,963,985	13,964,288	303	Loss
DEL00024717	chr3	13,992,718	13,993,234	516	Loss
DUP00024718	chr3	14,044,845	14,044,913	68	Gain
DEL00024722	chr3	14,118,032	14,118,154	122	Loss
DEL00024736	chr3	14,304,033	14,304,117	84	Loss
DEL00024738	chr3	14,329,059	14,331,275	2,216	Loss
DEL00024749	chr3	14,762,666	14,762,744	78	Loss
DEL00024753	chr3	14,786,264	14,787,465	1,201	Loss
DEL00024760	chr3	15,025,038	15,025,229	191	Loss

DEL00024763	chr3	15,083,271	15,083,522	251	Loss
DEL00024765	chr3	15,172,300	15,172,733	433	Loss
DEL00024767	chr3	15,244,627	15,244,830	203	Loss
DEL00024772	chr3	15,325,221	15,326,211	990	Loss
DEL00024787	chr3	15,689,238	15,689,695	457	Loss
DEL00024790	chr3	15,753,417	15,753,535	118	Loss
DEL00024800	chr3	15,901,241	15,901,383	142	Loss
DEL00024806	chr3	16,074,728	16,075,650	922	Loss
DEL00024809	chr3	16,087,220	16,088,104	884	Loss
DEL00024811	chr3	16,105,434	16,105,614	180	Loss
DEL00024816	chr3	16,207,484	16,207,674	190	Loss
DEL00024818	chr3	16,227,229	16,227,416	187	Loss
DEL00024819	chr3	16,243,644	16,243,821	177	Loss
DEL00024823	chr3	16,311,114	16,311,175	61	Loss
DEL00024830	chr3	16,498,920	16,498,997	77	Loss
DEL00024838	chr3	16,698,999	16,699,260	261	Loss
DEL00024842	chr3	16,781,726	16,781,880	154	Loss
DEL00024848	chr3	16,910,321	16,910,857	536	Loss
DEL00024858	chr3	17,152,962	17,153,018	56	Loss
DEL00024875	chr3	17,351,093	17,351,199	106	Loss
DEL00024885	chr3	17,612,481	17,612,541	60	Loss
DEL00024886	chr3	17,660,507	17,660,660	153	Loss
DEL00024889	chr3	17,785,809	17,786,263	454	Loss
DEL00024895	chr3	18,064,009	18,064,341	332	Loss
DEL00024897	chr3	18,131,170	18,131,222	52	Loss
DEL00024900	chr3	18,198,154	18,198,745	591	Loss
DEL00024906	chr3	18,412,868	18,412,985	117	Loss
DEL00024911	chr3	18,539,834	18,539,930	96	Loss
DEL00024914	chr3	18,600,161	18,600,406	245	Loss
DEL00024919	chr3	18,608,637	18,608,895	258	Loss
DEL00024921	chr3	18,636,732	18,636,842	110	Loss
DEL00024923	chr3	18,705,901	18,706,326	425	Loss
DEL00024924	chr3	18,731,913	18,732,353	440	Loss
DEL00024930	chr3	18,852,532	18,852,598	66	Loss
DEL00024931	chr3	18,857,052	18,857,145	93	Loss
DEL00024935	chr3	18,980,547	18,980,606	59	Loss
DEL00024939	chr3	19,003,704	19,003,789	85	Loss
DEL00024941	chr3	19,012,629	19,012,748	119	Loss
DEL00024946	chr3	19,072,039	19,072,372	333	Loss
DEL00024949	chr3	19,094,258	19,095,094	836	Loss
DEL00024959	chr3	19,203,756	19,204,013	257	Loss
DEL00024960	chr3	19,237,763	19,237,849	86	Loss
DEL00024961	chr3	19,255,442	19,255,741	299	Loss
DEL00024962	chr3	19,260,250	19,260,476	226	Loss
DEL00024964	chr3	19,287,397	19,290,688	3,291	Loss
DUP00024967	chr3	19,297,915	19,298,049	134	Gain
DEL00024968	chr3	19,315,042	19,315,097	55	Loss
DEL00024969	chr3	19,338,115	19,338,184	69	Loss
DEL00024972	chr3	19,371,919	19,371,983	64	Loss

DEL00024980	chr3	19,548,964	19,549,015	51	Loss
DEL00024985	chr3	19,643,451	19,644,197	746	Loss
DEL00024986	chr3	19,654,890	19,654,951	61	Loss
DEL00024988	chr3	19,713,183	19,713,259	76	Loss
DEL00024992	chr3	19,803,244	19,803,306	62	Loss
DEL00024995	chr3	19,956,532	19,956,623	91	Loss
DEL00025001	chr3	20,188,299	20,188,442	143	Loss
DEL00025002	chr3	20,237,950	20,238,039	89	Loss
DEL00025010	chr3	20,445,841	20,445,896	55	Loss
DEL00025011	chr3	20,476,392	20,477,575	1,183	Loss
DEL00025012	chr3	20,493,214	20,493,715	501	Loss
DEL00025013	chr3	20,511,794	20,511,915	121	Loss
DEL00025014	chr3	20,525,874	20,525,959	85	Loss
DEL00025015	chr3	20,541,411	20,541,660	249	Loss
DEL00025021	chr3	20,694,135	20,694,472	337	Loss
DEL00025027	chr3	20,828,067	20,828,123	56	Loss
DEL00025036	chr3	20,972,171	20,972,242	71	Loss
DEL00025037	chr3	20,978,502	20,979,254	752	Loss
DEL00025048	chr3	21,456,597	21,457,273	676	Loss
DEL00025049	chr3	21,526,288	21,526,427	139	Loss
DEL00025059	chr3	21,956,221	21,956,987	766	Loss
DEL00025064	chr3	22,039,253	22,039,835	582	Loss
DEL00025069	chr3	22,118,437	22,119,056	619	Loss
DEL00025070	chr3	22,120,417	22,120,543	126	Loss
DEL00025074	chr3	22,202,866	22,206,262	3,396	Loss
DEL00025080	chr3	22,388,928	22,389,029	101	Loss
DEL00025083	chr3	22,473,236	22,473,312	76	Loss
DEL00025093	chr3	22,614,153	22,614,256	103	Loss
DEL00025098	chr3	22,717,592	22,717,744	152	Loss
DEL00025099	chr3	22,723,668	22,723,751	83	Loss
DEL00025103	chr3	22,819,617	22,820,052	435	Loss
DEL00025105	chr3	22,960,948	22,961,048	100	Loss
DEL00025116	chr3	23,040,282	23,040,500	218	Loss
DEL00025120	chr3	23,122,303	23,122,995	692	Loss
DEL00025126	chr3	23,258,454	23,258,527	73	Loss
DEL00025129	chr3	23,373,626	23,373,888	262	Loss
DEL00025135	chr3	23,481,847	23,482,427	580	Loss
DEL00025136	chr3	23,489,847	23,490,043	196	Loss
DEL00025141	chr3	23,575,615	23,576,132	517	Loss
DEL00025142	chr3	23,581,681	23,582,256	575	Loss
DEL00025143	chr3	23,582,344	23,582,476	132	Loss
DEL00025147	chr3	23,619,216	23,619,267	51	Loss
DEL00025153	chr3	23,678,923	23,678,974	51	Loss
DEL00025154	chr3	23,682,931	23,683,373	442	Loss
DEL00025156	chr3	23,739,561	23,739,875	314	Loss
DEL00025164	chr3	24,058,926	24,059,521	595	Loss
DEL00025167	chr3	24,148,210	24,148,335	125	Loss
DEL00025168	chr3	24,186,986	24,187,946	960	Loss
DEL00025171	chr3	24,242,253	24,242,698	445	Loss

DEL00025175	chr3	24,264,779	24,265,246	467	Loss
DEL00025182	chr3	24,557,948	24,558,007	59	Loss
DEL00025185	chr3	24,632,378	24,632,555	177	Loss
DEL00025187	chr3	24,648,099	24,648,327	228	Loss
DEL00025190	chr3	24,769,607	24,769,661	54	Loss
DEL00025194	chr3	24,941,531	24,941,595	64	Loss
DEL00025199	chr3	24,991,485	24,991,884	399	Loss
DEL00025201	chr3	25,009,917	25,009,991	74	Loss
DEL00025206	chr3	25,061,186	25,061,534	348	Loss
DEL00025213	chr3	25,168,800	25,169,261	461	Loss
DEL00025214	chr3	25,180,827	25,180,890	63	Loss
DEL00025220	chr3	25,341,428	25,341,639	211	Loss
DEL00025221	chr3	25,364,304	25,364,612	308	Loss
DEL00025222	chr3	25,395,511	25,395,570	59	Loss
DEL00025223	chr3	25,421,948	25,422,073	125	Loss
DEL00025227	chr3	25,462,539	25,462,646	107	Loss
DEL00025230	chr3	25,499,141	25,499,211	70	Loss
DEL00025255	chr3	25,927,417	25,927,468	51	Loss
DUP00025256	chr3	25,978,025	25,996,245	18,220	Gain
DEL00025262	chr3	26,096,638	26,096,806	168	Loss
DEL00025265	chr3	26,110,926	26,111,411	485	Loss
DEL00025269	chr3	26,169,730	26,170,512	782	Loss
DUP00025277	chr3	26,309,292	26,309,352	60	Gain
DEL00025282	chr3	26,369,346	26,369,850	504	Loss
DEL00025285	chr3	26,476,588	26,477,003	415	Loss
DEL00025286	chr3	26,487,429	26,487,498	69	Loss
DEL00025287	chr3	26,540,406	26,540,577	171	Loss
DEL00025293	chr3	26,733,071	26,733,337	266	Loss
DEL00025294	chr3	26,765,410	26,766,257	847	Loss
DEL00025300	chr3	26,956,460	26,958,349	1,889	Loss
DEL00025306	chr3	27,007,671	27,008,230	559	Loss
DEL00025308	chr3	27,081,539	27,081,900	361	Loss
DEL00025309	chr3	27,088,998	27,089,218	220	Loss
DEL00025312	chr3	27,210,220	27,210,445	225	Loss
DEL00025319	chr3	27,291,752	27,292,100	348	Loss
DEL00025326	chr3	27,436,809	27,436,919	110	Loss
DEL00025329	chr3	27,464,906	27,465,081	175	Loss
DEL00025335	chr3	27,539,855	27,539,917	62	Loss
DEL00025337	chr3	27,605,743	27,605,935	192	Loss
DEL00025349	chr3	27,903,834	27,904,103	269	Loss
DEL00025354	chr3	27,983,385	27,983,462	77	Loss
DEL00025362	chr3	28,076,504	28,076,702	198	Loss
DEL00025370	chr3	28,243,335	28,244,395	1,060	Loss
DEL00025373	chr3	28,253,931	28,257,162	3,231	Loss
DEL00025379	chr3	28,353,882	28,354,156	274	Loss
DEL00025380	chr3	28,410,172	28,410,403	231	Loss
DEL00025381	chr3	28,410,503	28,411,993	1,490	Loss
DEL00025384	chr3	28,447,738	28,447,919	181	Loss
DEL00025387	chr3	28,520,104	28,520,856	752	Loss

DEL00025389	chr3	28,612,970	28,614,135	1,165	Loss
DEL00025393	chr3	28,667,176	28,667,491	315	Loss
DEL00025401	chr3	28,960,719	28,961,383	664	Loss
DEL00025404	chr3	29,131,461	29,134,265	2,804	Loss
DEL00025408	chr3	29,270,146	29,270,445	299	Loss
DEL00025410	chr3	29,290,922	29,290,989	67	Loss
DEL00025424	chr3	29,409,492	29,409,604	112	Loss
DEL00025427	chr3	29,433,932	29,434,611	679	Loss
DEL00025431	chr3	29,454,555	29,454,719	164	Loss
DEL00025433	chr3	29,527,089	29,527,433	344	Loss
DEL00025436	chr3	29,733,219	29,733,471	252	Loss
DEL00025438	chr3	29,738,477	29,738,643	166	Loss
DEL00025447	chr3	29,890,147	29,890,970	823	Loss
DEL00025448	chr3	29,946,211	29,946,264	53	Loss
DEL00025467	chr3	30,536,363	30,536,483	120	Loss
DUP00025475	chr3	30,981,143	30,981,214	71	Gain
DEL00025478	chr3	31,029,316	31,029,470	154	Loss
DUP00025480	chr3	31,039,656	31,039,837	181	Gain
DEL00025482	chr3	31,045,658	31,046,013	355	Loss
DEL00025486	chr3	31,073,670	31,073,738	68	Loss
DEL00025488	chr3	31,153,890	31,157,713	3,823	Loss
DEL00025497	chr3	31,346,415	31,346,893	478	Loss
DEL00025498	chr3	31,357,764	31,357,875	111	Loss
DEL00025502	chr3	31,403,720	31,404,516	796	Loss
DEL00025507	chr3	31,428,646	31,428,721	75	Loss
DEL00025512	chr3	31,471,335	31,471,625	290	Loss
DEL00025515	chr3	31,554,580	31,554,637	57	Loss
DEL00025516	chr3	31,561,475	31,561,539	64	Loss
DEL00025519	chr3	31,579,740	31,579,794	54	Loss
DEL00025526	chr3	31,670,588	31,670,787	199	Loss
DEL00025527	chr3	31,687,335	31,687,386	51	Loss
DEL00025528	chr3	31,692,058	31,692,147	89	Loss
DEL00025529	chr3	31,714,147	31,714,660	513	Loss
DEL00025535	chr3	31,867,378	31,867,697	319	Loss
DEL00025540	chr3	31,936,828	31,937,178	350	Loss
DEL00025545	chr3	32,027,444	32,027,703	259	Loss
DEL00025549	chr3	32,071,602	32,071,786	184	Loss
DEL00025550	chr3	32,089,337	32,089,451	114	Loss
DEL00025551	chr3	32,168,255	32,168,387	132	Loss
DEL00025555	chr3	32,244,990	32,246,681	1,691	Loss
DEL00025564	chr3	32,415,493	32,418,117	2,624	Loss
DEL00025570	chr3	32,591,448	32,591,592	144	Loss
DEL00025571	chr3	32,596,032	32,596,292	260	Loss
DEL00025573	chr3	32,609,154	32,609,225	71	Loss
DEL00025575	chr3	32,633,306	32,633,357	51	Loss
DEL00025577	chr3	32,655,251	32,655,316	65	Loss
DEL00025578	chr3	32,704,955	32,705,123	168	Loss
DEL00025580	chr3	32,717,452	32,717,672	220	Loss
DEL00025581	chr3	32,721,496	32,722,434	938	Loss

DUP00025587	chr3	32,807,753	32,820,669	12,916	Gain
DEL00025592	chr3	32,871,854	32,872,526	672	Loss
DEL00025596	chr3	32,987,134	32,987,188	54	Loss
DEL00025600	chr3	33,023,474	33,023,532	58	Loss
DEL00025602	chr3	33,049,418	33,049,810	392	Loss
DEL00025603	chr3	33,056,488	33,056,553	65	Loss
DEL00025605	chr3	33,110,119	33,111,247	1,128	Loss
DEL00025617	chr3	33,303,449	33,303,528	79	Loss
DEL00025619	chr3	33,339,172	33,339,452	280	Loss
DEL00025622	chr3	33,372,632	33,372,697	65	Loss
DEL00025650	chr3	33,752,248	33,753,047	799	Loss
DEL00025651	chr3	33,758,442	33,758,551	109	Loss
DEL00025653	chr3	33,793,095	33,793,164	69	Loss
DEL00025659	chr3	33,901,100	33,901,243	143	Loss
DEL00025668	chr3	34,073,882	34,074,012	130	Loss
DEL00025674	chr3	34,137,110	34,137,482	372	Loss
DEL00025675	chr3	34,159,105	34,159,181	76	Loss
DUP00025677	chr3	34,178,978	34,179,105	127	Gain
DUP00025683	chr3	34,293,591	34,293,752	161	Gain
DEL00025685	chr3	34,351,604	34,351,878	274	Loss
DEL00025690	chr3	34,423,361	34,424,377	1,016	Loss
DEL00025694	chr3	34,534,965	34,535,049	84	Loss
DEL00025695	chr3	34,535,673	34,539,066	3,393	Loss
DEL00025700	chr3	34,591,831	34,591,997	166	Loss
DEL00025701	chr3	34,633,613	34,633,725	112	Loss
DEL00025702	chr3	34,659,147	34,659,229	82	Loss
DEL00025703	chr3	34,665,164	34,665,788	624	Loss
DEL00025704	chr3	34,687,005	34,687,069	64	Loss
DEL00025707	chr3	34,764,865	34,764,962	97	Loss
DEL00025709	chr3	34,801,163	34,801,255	92	Loss
DEL00025721	chr3	35,120,040	35,120,276	236	Loss
DEL00025723	chr3	35,148,362	35,148,621	259	Loss
DUP00025725	chr3	35,247,727	35,247,872	145	Gain
DEL00025727	chr3	35,290,950	35,291,278	328	Loss
DEL00025735	chr3	35,413,067	35,413,360	293	Loss
DEL00025739	chr3	35,509,782	35,509,960	178	Loss
DEL00025741	chr3	35,530,305	35,530,575	270	Loss
DEL00025747	chr3	35,639,836	35,640,061	225	Loss
DEL00025750	chr3	35,658,896	35,658,979	83	Loss
DEL00025751	chr3	35,659,678	35,659,778	100	Loss
DEL00025757	chr3	35,781,205	35,781,380	175	Loss
DEL00025762	chr3	35,852,792	35,852,909	117	Loss
DEL00025764	chr3	35,861,481	35,862,001	520	Loss
DEL00025766	chr3	35,869,897	35,872,637	2,740	Loss
DEL00025771	chr3	35,926,212	35,926,623	411	Loss
DEL00025775	chr3	36,044,574	36,044,876	302	Loss
DEL00025777	chr3	36,069,851	36,070,950	1,099	Loss
DEL00025780	chr3	36,114,552	36,114,613	61	Loss
DEL00025782	chr3	36,132,579	36,132,822	243	Loss

DEL00025802	chr3	36,664,559	36,664,640	81	Loss
DEL00025814	chr3	36,784,168	36,784,221	53	Loss
DEL00025815	chr3	36,817,471	36,817,554	83	Loss
DEL00025821	chr3	36,869,517	36,869,671	154	Loss
DEL00025823	chr3	36,915,330	36,918,167	2,837	Loss
DEL00025827	chr3	37,040,400	37,040,925	525	Loss
DEL00025828	chr3	37,052,838	37,053,007	169	Loss
DEL00025835	chr3	37,382,758	37,383,381	623	Loss
DEL00025836	chr3	37,399,700	37,399,837	137	Loss
DEL00025838	chr3	37,412,888	37,413,084	196	Loss
DEL00025846	chr3	37,550,891	37,550,992	101	Loss
DEL00025847	chr3	37,606,277	37,606,582	305	Loss
DEL00025849	chr3	37,608,504	37,608,639	135	Loss
DEL00025853	chr3	37,655,257	37,655,317	60	Loss
DEL00025855	chr3	37,672,650	37,672,725	75	Loss
DEL00025866	chr3	37,754,076	37,754,846	770	Loss
DEL00025871	chr3	37,914,306	37,914,457	151	Loss
DEL00025872	chr3	37,964,070	37,966,893	2,823	Loss
DEL00025875	chr3	38,002,465	38,003,061	596	Loss
DEL00025877	chr3	38,018,798	38,019,065	267	Loss
DEL00025878	chr3	38,039,496	38,039,892	396	Loss
DEL00025880	chr3	38,151,456	38,151,549	93	Loss
DEL00025883	chr3	38,183,521	38,183,633	112	Loss
DEL00025886	chr3	38,184,318	38,184,640	322	Loss
DEL00025889	chr3	38,364,239	38,364,955	716	Loss
DUP00025891	chr3	38,381,749	38,381,828	79	Gain
DEL00025897	chr3	38,473,721	38,475,057	1,336	Loss
DEL00025898	chr3	38,477,591	38,477,701	110	Loss
DEL00025899	chr3	38,499,389	38,500,442	1,053	Loss
DEL00025900	chr3	38,540,645	38,540,714	69	Loss
DEL00025906	chr3	38,600,322	38,600,387	65	Loss
DEL00025908	chr3	38,630,708	38,630,763	55	Loss
DEL00025913	chr3	38,840,079	38,840,859	780	Loss
DEL00025915	chr3	38,879,578	38,879,650	72	Loss
DEL00025916	chr3	38,906,598	38,906,660	62	Loss
DEL00025918	chr3	38,947,495	38,947,546	51	Loss
DEL00025919	chr3	39,031,992	39,032,113	121	Loss
DEL00025926	chr3	39,153,532	39,153,622	90	Loss
DEL00025928	chr3	39,157,576	39,157,647	71	Loss
DEL00025931	chr3	39,211,911	39,211,999	88	Loss
DEL00025932	chr3	39,233,483	39,233,668	185	Loss
DEL00025934	chr3	39,281,941	39,282,394	453	Loss
DEL00025940	chr3	39,425,597	39,426,169	572	Loss
DEL00025943	chr3	39,479,836	39,479,951	115	Loss
DEL00025949	chr3	39,553,474	39,553,654	180	Loss
DEL00025950	chr3	39,568,196	39,569,842	1,646	Loss
DEL00025954	chr3	39,611,180	39,616,856	5,676	Loss
DEL00025955	chr3	39,626,100	39,626,164	64	Loss
DEL00025957	chr3	39,634,942	39,635,117	175	Loss

DEL00025969	chr3	39,900,874	39,901,374	500	Loss
DEL00025972	chr3	39,931,896	39,932,006	110	Loss
DEL00025975	chr3	40,004,783	40,004,873	90	Loss
DEL00025977	chr3	40,005,544	40,005,755	211	Loss
DEL00025978	chr3	40,009,493	40,009,679	186	Loss
DEL00025979	chr3	40,024,125	40,024,278	153	Loss
DEL00025985	chr3	40,122,856	40,123,086	230	Loss
DEL00025986	chr3	40,149,052	40,149,523	471	Loss
DEL00025995	chr3	40,275,147	40,275,285	138	Loss
DEL00026002	chr3	40,411,848	40,411,945	97	Loss
DEL00026005	chr3	40,507,687	40,507,749	62	Loss
DEL00026013	chr3	40,581,555	40,582,298	743	Loss
DEL00026023	chr3	40,841,131	40,841,908	777	Loss
DEL00026024	chr3	40,842,001	40,842,299	298	Loss
DEL00026029	chr3	40,896,562	40,896,720	158	Loss
DEL00026034	chr3	40,965,200	40,965,697	497	Loss
DEL00026039	chr3	41,073,762	41,074,476	714	Loss
DEL00026043	chr3	41,091,850	41,092,027	177	Loss
DEL00026053	chr3	41,184,570	41,184,688	118	Loss
DEL00026057	chr3	41,301,810	41,304,824	3,014	Loss
DEL00026059	chr3	41,316,361	41,316,439	78	Loss
DEL00026060	chr3	41,318,003	41,318,851	848	Loss
DEL00026062	chr3	41,344,066	41,344,268	202	Loss
DEL00026068	chr3	41,454,556	41,455,088	532	Loss
DEL00026069	chr3	41,472,523	41,472,618	95	Loss
DEL00026074	chr3	41,586,299	41,586,353	54	Loss
DEL00026075	chr3	41,598,431	41,598,642	211	Loss
DEL00026083	chr3	41,911,442	41,912,262	820	Loss
DUP00026084	chr3	41,937,807	41,937,940	133	Gain
DEL00026087	chr3	41,963,824	41,964,066	242	Loss
DEL00026091	chr3	42,085,172	42,085,233	61	Loss
DEL00026095	chr3	42,126,128	42,126,410	282	Loss
DEL00026097	chr3	42,129,538	42,129,898	360	Loss
DEL00026105	chr3	42,178,656	42,178,921	265	Loss
DEL00026109	chr3	42,237,151	42,237,485	334	Loss
DEL00026113	chr3	42,273,453	42,273,550	97	Loss
DEL00026114	chr3	42,280,930	42,281,140	210	Loss
DEL00026115	chr3	42,282,669	42,282,970	301	Loss
DEL00026118	chr3	42,326,716	42,327,641	925	Loss
DUP00026121	chr3	42,417,385	42,417,903	518	Gain
DEL00026126	chr3	42,457,311	42,457,397	86	Loss
DEL00026143	chr3	42,663,036	42,663,182	146	Loss
DEL00026154	chr3	42,771,444	42,771,519	75	Loss
DEL00026155	chr3	42,782,912	42,783,357	445	Loss
DEL00026160	chr3	42,881,247	42,881,333	86	Loss
DEL00026162	chr3	42,905,403	42,905,560	157	Loss
DEL00026166	chr3	42,951,136	42,951,223	87	Loss
DEL00026170	chr3	43,010,160	43,015,174	5,014	Loss
DEL00026178	chr3	43,128,053	43,128,255	202	Loss

DEL00026182	chr3	43,189,275	43,189,450	175	Loss
DEL00026193	chr3	43,260,722	43,260,773	51	Loss
DEL00026199	chr3	43,358,824	43,359,514	690	Loss
DEL00026207	chr3	43,431,102	43,431,370	268	Loss
DEL00026209	chr3	43,561,889	43,562,357	468	Loss
DEL00026217	chr3	43,756,396	43,756,778	382	Loss
DEL00026218	chr3	43,757,302	43,757,378	76	Loss
DEL00026222	chr3	43,785,067	43,785,389	322	Loss
DEL00026245	chr3	44,160,580	44,160,688	108	Loss
DEL00026247	chr3	44,197,418	44,197,519	101	Loss
DEL00026252	chr3	44,348,904	44,349,451	547	Loss
DEL00026254	chr3	44,378,273	44,378,326	53	Loss
DEL00026256	chr3	44,424,971	44,426,950	1,979	Loss
DEL00026260	chr3	44,454,842	44,454,983	141	Loss
DEL00026262	chr3	44,456,485	44,456,779	294	Loss
DEL00026263	chr3	44,467,776	44,467,832	56	Loss
DEL00026266	chr3	44,503,031	44,503,086	55	Loss
DUP00026268	chr3	44,544,185	44,544,407	222	Gain
DUP00026271	chr3	44,632,440	44,632,510	70	Gain
DEL00026273	chr3	44,665,500	44,667,753	2,253	Loss
DEL00026275	chr3	44,681,857	44,682,606	749	Loss
DEL00026279	chr3	44,711,166	44,711,265	99	Loss
DEL00026280	chr3	44,713,078	44,713,173	95	Loss
DUP00026285	chr3	44,772,089	44,776,784	4,695	Gain
DEL00026286	chr3	44,777,394	44,780,098	2,704	Loss
DEL00026304	chr3	44,967,211	44,967,851	640	Loss
DEL00026310	chr3	45,026,298	45,026,664	366	Loss
DEL00026312	chr3	45,074,713	45,074,766	53	Loss
DEL00026315	chr3	45,159,223	45,160,065	842	Loss
DEL00026316	chr3	45,202,909	45,204,114	1,205	Loss
DEL00026327	chr3	45,325,633	45,325,880	247	Loss
DEL00026332	chr3	45,367,910	45,368,270	360	Loss
DEL00026336	chr3	45,527,651	45,527,764	113	Loss
DEL00026337	chr3	45,537,666	45,538,281	615	Loss
DEL00026341	chr3	45,614,795	45,615,606	811	Loss
DEL00026342	chr3	45,628,087	45,628,146	59	Loss
DEL00026351	chr3	45,801,002	45,801,585	583	Loss
DEL00026360	chr3	45,868,321	45,871,911	3,590	Loss
DEL00026372	chr3	46,024,493	46,025,740	1,247	Loss
DEL00026373	chr3	46,046,666	46,047,884	1,218	Loss
DEL00026378	chr3	46,137,389	46,137,442	53	Loss
DEL00026379	chr3	46,205,580	46,205,739	159	Loss
DEL00026382	chr3	46,226,431	46,226,953	522	Loss
DEL00026386	chr3	46,311,243	46,311,486	243	Loss
DEL00026387	chr3	46,337,362	46,338,314	952	Loss
DEL00026392	chr3	46,366,664	46,366,948	284	Loss
DEL00026393	chr3	46,408,378	46,409,484	1,106	Loss
DEL00026395	chr3	46,501,480	46,501,565	85	Loss
DEL00026401	chr3	46,739,390	46,739,944	554	Loss

DEL00026402	chr3	46,740,208	46,740,286	78	Loss
DEL00026406	chr3	46,786,661	46,786,917	256	Loss
DEL00026414	chr3	46,910,427	46,910,530	103	Loss
DEL00026417	chr3	46,941,364	46,941,470	106	Loss
DEL00026418	chr3	46,960,924	46,961,339	415	Loss
DEL00026421	chr3	47,040,612	47,042,504	1,892	Loss
DEL00026435	chr3	47,269,515	47,269,578	63	Loss
DEL00026440	chr3	47,316,807	47,316,969	162	Loss
DEL00026448	chr3	47,398,357	47,399,954	1,597	Loss
DEL00026453	chr3	47,442,033	47,442,558	525	Loss
DEL00026456	chr3	47,468,403	47,483,749	15,346	Loss
DEL00026461	chr3	47,611,632	47,612,626	994	Loss
DEL00026463	chr3	47,753,725	47,753,784	59	Loss
DEL00026467	chr3	47,952,212	47,952,583	371	Loss
DEL00026471	chr3	48,013,811	48,014,791	980	Loss
DEL00026472	chr3	48,032,838	48,033,461	623	Loss
DEL00026485	chr3	48,155,285	48,155,361	76	Loss
DEL00026486	chr3	48,175,811	48,176,646	835	Loss
DEL00026488	chr3	48,247,378	48,247,526	148	Loss
DEL00026491	chr3	48,300,203	48,300,262	59	Loss
DEL00026498	chr3	48,411,661	48,411,729	68	Loss
DEL00026500	chr3	48,457,693	48,457,866	173	Loss
DEL00026518	chr3	48,822,650	48,823,105	455	Loss
DEL00026521	chr3	48,853,196	48,853,591	395	Loss
DEL00026525	chr3	48,901,841	48,902,064	223	Loss
DEL00026529	chr3	49,006,953	49,007,050	97	Loss
DEL00026530	chr3	49,010,644	49,010,792	148	Loss
DEL00026539	chr3	49,174,425	49,174,536	111	Loss
DEL00026540	chr3	49,186,076	49,186,151	75	Loss
DEL00026543	chr3	49,261,267	49,261,967	700	Loss
DEL00026544	chr3	49,306,960	49,307,042	82	Loss
DEL00026545	chr3	49,359,033	49,359,178	145	Loss
DEL00026546	chr3	49,364,320	49,364,435	115	Loss
DEL00026547	chr3	49,377,259	49,377,545	286	Loss
DEL00026548	chr3	49,380,171	49,380,486	315	Loss
DEL00026549	chr3	49,389,806	49,390,376	570	Loss
DEL00026553	chr3	49,458,873	49,458,947	74	Loss
DEL00026561	chr3	49,565,624	49,568,795	3,171	Loss
DEL00026562	chr3	49,586,706	49,586,799	93	Loss
DEL00026567	chr3	49,636,232	49,636,492	260	Loss
DEL00026574	chr3	49,762,325	49,762,614	289	Loss
DEL00026577	chr3	49,764,128	49,764,186	58	Loss
DEL00026578	chr3	49,810,502	49,810,943	441	Loss
DEL00026580	chr3	49,812,597	49,812,784	187	Loss
DEL00026581	chr3	49,833,153	49,833,254	101	Loss
DEL00026582	chr3	49,862,205	49,865,779	3,574	Loss
DEL00026588	chr3	50,143,296	50,143,620	324	Loss
DEL00026591	chr3	50,158,018	50,158,837	819	Loss
DEL00026594	chr3	50,365,289	50,365,506	217	Loss

DEL00026600	chr3	50,432,140	50,432,295	155	Loss
DEL00026605	chr3	50,466,386	50,466,507	121	Loss
DUP00026608	chr3	50,595,181	50,603,143	7,962	Gain
DEL00026612	chr3	50,771,445	50,771,667	222	Loss
DEL00026620	chr3	50,872,570	50,872,745	175	Loss
DEL00026628	chr3	51,312,282	51,312,343	61	Loss
DEL00026629	chr3	51,393,575	51,394,149	574	Loss
DEL00026632	chr3	51,444,394	51,444,732	338	Loss
DEL00026639	chr3	51,666,628	51,666,681	53	Loss
DEL00026642	chr3	51,746,284	51,747,466	1,182	Loss
DEL00026664	chr3	52,098,021	52,098,072	51	Loss
DEL00026667	chr3	52,157,226	52,157,361	135	Loss
DEL00026668	chr3	52,169,404	52,169,796	392	Loss
DEL00026669	chr3	52,190,805	52,190,857	52	Loss
DEL00026670	chr3	52,247,792	52,248,184	392	Loss
DEL00026674	chr3	52,348,201	52,348,704	503	Loss
DEL00026675	chr3	52,370,515	52,370,983	468	Loss
DEL00026680	chr3	52,590,449	52,591,095	646	Loss
DEL00026681	chr3	52,615,578	52,615,683	105	Loss
DEL00026684	chr3	52,686,765	52,686,826	61	Loss
DEL00026695	chr3	52,971,760	52,971,817	57	Loss
DEL00026697	chr3	52,995,619	52,996,061	442	Loss
DEL00026703	chr3	53,129,241	53,129,404	163	Loss
DEL00026709	chr3	53,177,053	53,177,400	347	Loss
DEL00026716	chr3	53,306,259	53,306,313	54	Loss
DEL00026717	chr3	53,347,009	53,347,065	56	Loss
DEL00026720	chr3	53,388,850	53,388,911	61	Loss
DEL00026721	chr3	53,390,869	53,390,935	66	Loss
DEL00026722	chr3	53,393,720	53,394,490	770	Loss
DEL00026723	chr3	53,400,892	53,400,954	62	Loss
DEL00026724	chr3	53,426,079	53,426,659	580	Loss
DEL00026726	chr3	53,429,915	53,430,103	188	Loss
DEL00026727	chr3	53,448,659	53,448,933	274	Loss
DUP00026728	chr3	53,488,292	53,488,406	114	Gain
DEL00026731	chr3	53,496,249	53,496,668	419	Loss
DEL00026737	chr3	53,584,395	53,585,190	795	Loss
DEL00026738	chr3	53,585,597	53,585,651	54	Loss
DEL00026742	chr3	53,658,205	53,658,326	121	Loss
DEL00026754	chr3	53,826,576	53,827,434	858	Loss
DEL00026756	chr3	53,829,371	53,829,918	547	Loss
DEL00026764	chr3	53,937,107	53,937,178	71	Loss
DEL00026767	chr3	53,999,692	53,999,978	286	Loss
DEL00026770	chr3	54,019,221	54,019,400	179	Loss
DEL00026772	chr3	54,075,473	54,075,549	76	Loss
DEL00026774	chr3	54,082,544	54,082,628	84	Loss
DEL00026775	chr3	54,116,122	54,116,217	95	Loss
DEL00026777	chr3	54,168,282	54,168,366	84	Loss
DEL00026778	chr3	54,170,061	54,170,114	53	Loss
DEL00026780	chr3	54,187,316	54,188,800	1,484	Loss

DEL00026785	chr3	54,228,745	54,229,040	295	Loss
DEL00026788	chr3	54,276,595	54,277,199	604	Loss
DEL00026805	chr3	54,560,683	54,560,756	73	Loss
DEL00026810	chr3	54,640,511	54,640,852	341	Loss
DEL00026835	chr3	55,093,001	55,093,072	71	Loss
DEL00026842	chr3	55,239,938	55,240,123	185	Loss
DEL00026843	chr3	55,240,231	55,242,983	2,752	Loss
DEL00026846	chr3	55,250,453	55,250,964	511	Loss
DEL00026850	chr3	55,394,479	55,394,599	120	Loss
DEL00026856	chr3	55,491,249	55,491,349	100	Loss
DEL00026857	chr3	55,503,461	55,503,666	205	Loss
DEL00026858	chr3	55,542,103	55,542,163	60	Loss
DEL00026861	chr3	55,561,803	55,562,037	234	Loss
DEL00026864	chr3	55,579,969	55,580,020	51	Loss
DEL00026867	chr3	55,854,842	55,854,907	65	Loss
DEL00026868	chr3	55,881,858	55,882,654	796	Loss
DEL00026870	chr3	55,897,671	55,897,953	282	Loss
DEL00026876	chr3	56,051,325	56,051,422	97	Loss
DEL00026880	chr3	56,081,316	56,081,419	103	Loss
DEL00026885	chr3	56,148,956	56,150,993	2,037	Loss
DEL00026908	chr3	56,364,771	56,365,008	237	Loss
DEL00026913	chr3	56,403,636	56,403,692	56	Loss
DEL00026914	chr3	56,413,047	56,413,172	125	Loss
DEL00026916	chr3	56,441,944	56,442,123	179	Loss
DEL00026925	chr3	56,717,500	56,717,572	72	Loss
DEL00026926	chr3	56,719,753	56,719,904	151	Loss
DEL00026928	chr3	56,793,774	56,793,830	56	Loss
DEL00026929	chr3	56,799,689	56,799,871	182	Loss
DEL00026932	chr3	56,822,211	56,822,279	68	Loss
DUP00026933	chr3	56,828,206	56,828,280	74	Gain
DEL00026935	chr3	56,854,582	56,854,742	160	Loss
DEL00026938	chr3	56,897,146	56,897,220	74	Loss
DEL00026942	chr3	56,914,879	56,914,939	60	Loss
DEL00026943	chr3	56,926,743	56,926,997	254	Loss
DUP00026945	chr3	56,940,640	56,940,746	106	Gain
DEL00026956	chr3	57,205,002	57,205,240	238	Loss
DEL00026961	chr3	57,298,825	57,298,895	70	Loss
DEL00026964	chr3	57,386,996	57,387,529	533	Loss
DEL00026967	chr3	57,499,108	57,499,738	630	Loss
DEL00026970	chr3	57,530,637	57,530,695	58	Loss
DEL00026971	chr3	57,542,922	57,543,403	481	Loss
DEL00026972	chr3	57,582,854	57,582,987	133	Loss
DEL00026977	chr3	57,684,128	57,693,242	9,114	Loss
DEL00026981	chr3	57,761,975	57,762,045	70	Loss
DEL00026982	chr3	57,787,702	57,789,173	1,471	Loss
DEL00026984	chr3	57,860,539	57,860,599	60	Loss
DEL00026985	chr3	57,864,515	57,864,632	117	Loss
DEL00026987	chr3	57,883,345	57,883,922	577	Loss
DEL00026996	chr3	58,042,727	58,042,834	107	Loss

DEL00026999	chr3	58,066,746	58,066,912	166	Loss
DEL00027005	chr3	58,090,580	58,091,104	524	Loss
DEL00027007	chr3	58,127,638	58,128,187	549	Loss
DEL00027008	chr3	58,133,785	58,134,423	638	Loss
DEL00027011	chr3	58,236,988	58,237,130	142	Loss
DEL00027014	chr3	58,312,121	58,312,783	662	Loss
DEL00027020	chr3	58,421,833	58,421,968	135	Loss
DEL00027023	chr3	58,485,904	58,486,048	144	Loss
DEL00027024	chr3	58,493,104	58,493,943	839	Loss
DEL00027025	chr3	58,587,918	58,589,023	1,105	Loss
DEL00027026	chr3	58,604,043	58,604,164	121	Loss
DEL00027033	chr3	58,670,506	58,670,605	99	Loss
DEL00027047	chr3	59,083,122	59,083,217	95	Loss
DUP00027048	chr3	59,108,322	59,108,581	259	Gain
DUP00027052	chr3	59,216,969	59,226,118	9,149	Gain
DEL00027061	chr3	59,331,154	59,331,205	51	Loss
DEL00027069	chr3	59,455,254	59,455,427	173	Loss
DEL00027072	chr3	59,518,790	59,519,170	380	Loss
DEL00027073	chr3	59,519,691	59,520,001	310	Loss
DEL00027086	chr3	59,773,893	59,774,587	694	Loss
DEL00027089	chr3	59,804,344	59,804,736	392	Loss
DEL00027101	chr3	59,945,923	59,946,472	549	Loss
DEL00027111	chr3	60,015,342	60,015,536	194	Loss
DEL00027121	chr3	60,076,852	60,077,160	308	Loss
DEL00027129	chr3	60,184,768	60,185,417	649	Loss
DEL00027131	chr3	60,187,105	60,187,665	560	Loss
DEL00027133	chr3	60,310,617	60,310,674	57	Loss
DEL00027138	chr3	60,375,586	60,375,675	89	Loss
DEL00027140	chr3	60,391,516	60,392,052	536	Loss
DEL00027141	chr3	60,399,684	60,401,836	2,152	Loss
DEL00027142	chr3	60,436,705	60,437,119	414	Loss
DEL00027153	chr3	60,677,502	60,677,827	325	Loss
DEL00027160	chr3	60,707,027	60,708,108	1,081	Loss
DEL00027162	chr3	60,721,685	60,722,113	428	Loss
DEL00027169	chr3	60,747,652	60,749,047	1,395	Loss
DEL00027171	chr3	60,765,938	60,766,367	429	Loss
DEL00027173	chr3	60,790,965	60,791,440	475	Loss
DEL00027174	chr3	60,811,166	60,811,245	79	Loss
DEL00027175	chr3	60,852,284	60,852,794	510	Loss
DEL00027182	chr3	60,933,409	60,933,947	538	Loss
DUP00027183	chr3	60,941,742	60,941,875	133	Gain
DEL00027188	chr3	60,954,603	60,954,783	180	Loss
DEL00027190	chr3	60,976,375	60,976,465	90	Loss
DEL00027197	chr3	61,131,919	61,131,980	61	Loss
DEL00027201	chr3	61,202,721	61,203,648	927	Loss
DEL00027203	chr3	61,249,905	61,250,320	415	Loss
DEL00027220	chr3	61,569,983	61,570,496	513	Loss
DEL00027221	chr3	61,580,103	61,581,656	1,553	Loss
DEL00027224	chr3	61,602,540	61,602,599	59	Loss

DEL00027232	chr3	61,702,173	61,702,368	195	Loss
DEL00027237	chr3	61,764,680	61,764,810	130	Loss
DEL00027239	chr3	61,796,400	61,797,420	1,020	Loss
DEL00027251	chr3	61,882,050	61,882,331	281	Loss
DEL00027255	chr3	61,975,997	61,976,584	587	Loss
DEL00027259	chr3	61,995,779	61,996,458	679	Loss
DEL00027261	chr3	62,057,997	62,058,085	88	Loss
DEL00027266	chr3	62,209,247	62,210,479	1,232	Loss
DEL00027268	chr3	62,232,952	62,233,843	891	Loss
DEL00027270	chr3	62,247,173	62,247,297	124	Loss
DEL00027272	chr3	62,319,095	62,319,294	199	Loss
DEL00027273	chr3	62,419,956	62,420,034	78	Loss
DEL00027275	chr3	62,484,269	62,484,416	147	Loss
DEL00027276	chr3	62,564,190	62,564,244	54	Loss
DEL00027278	chr3	62,565,324	62,566,109	785	Loss
DEL00027281	chr3	62,597,310	62,597,361	51	Loss
DEL00027282	chr3	62,611,782	62,612,006	224	Loss
DEL00027288	chr3	62,729,895	62,730,355	460	Loss
DEL00027294	chr3	62,826,310	62,827,222	912	Loss
DEL00027304	chr3	63,004,762	63,004,869	107	Loss
DEL00027312	chr3	63,173,148	63,173,931	783	Loss
DEL00027316	chr3	63,276,465	63,276,873	408	Loss
DEL00027317	chr3	63,334,550	63,334,732	182	Loss
DEL00027336	chr3	63,640,082	63,640,205	123	Loss
DEL00027339	chr3	63,704,313	63,704,711	398	Loss
DEL00027340	chr3	63,751,321	63,751,574	253	Loss
DEL00027345	chr3	63,826,183	63,826,285	102	Loss
DEL00027347	chr3	63,828,978	63,829,033	55	Loss
DEL00027348	chr3	63,829,258	63,829,802	544	Loss
DEL00027350	chr3	63,841,279	63,841,364	85	Loss
DEL00027369	chr3	64,279,125	64,279,867	742	Loss
DEL00027375	chr3	64,353,315	64,353,468	153	Loss
DEL00027377	chr3	64,391,133	64,397,564	6,431	Loss
DEL00027380	chr3	64,420,473	64,421,033	560	Loss
DEL00027392	chr3	64,615,066	64,615,190	124	Loss
DEL00027394	chr3	64,644,582	64,644,649	67	Loss
DEL00027396	chr3	64,658,386	64,662,748	4,362	Loss
DEL00027401	chr3	64,709,433	64,709,580	147	Loss
DEL00027404	chr3	64,784,452	64,784,524	72	Loss
DEL00027406	chr3	64,858,153	64,858,420	267	Loss
DEL00027416	chr3	65,045,033	65,046,459	1,426	Loss
DEL00027429	chr3	65,261,053	65,261,980	927	Loss
DEL00027433	chr3	65,319,413	65,320,041	628	Loss
DEL00027437	chr3	65,347,429	65,347,497	68	Loss
DEL00027445	chr3	65,470,934	65,471,790	856	Loss
DEL00027447	chr3	65,517,182	65,517,278	96	Loss
DEL00027450	chr3	65,538,044	65,538,900	856	Loss
DEL00027456	chr3	65,588,482	65,589,101	619	Loss
DEL00027463	chr3	65,729,574	65,729,915	341	Loss

DEL00027466	chr3	65,753,819	65,753,936	117	Loss
DEL00027468	chr3	65,772,282	65,772,333	51	Loss
DEL00027470	chr3	65,793,443	65,793,549	106	Loss
DEL00027476	chr3	65,860,201	65,860,491	290	Loss
DEL00027482	chr3	66,065,814	66,066,064	250	Loss
DEL00027484	chr3	66,094,515	66,094,607	92	Loss
DEL00027489	chr3	66,196,159	66,196,763	604	Loss
DEL00027490	chr3	66,261,848	66,261,967	119	Loss
DEL00027492	chr3	66,287,759	66,287,878	119	Loss
DEL00027495	chr3	66,307,168	66,309,152	1,984	Loss
DEL00027497	chr3	66,340,436	66,340,489	53	Loss
DEL00027502	chr3	66,370,807	66,370,860	53	Loss
DEL00027508	chr3	66,442,425	66,442,620	195	Loss
DEL00027514	chr3	66,527,288	66,527,463	175	Loss
DEL00027515	chr3	66,541,609	66,541,682	73	Loss
DEL00027516	chr3	66,566,278	66,566,902	624	Loss
DEL00027523	chr3	66,648,945	66,649,166	221	Loss
DEL00027524	chr3	66,660,248	66,660,440	192	Loss
DEL00027525	chr3	66,678,623	66,679,168	545	Loss
DEL00027526	chr3	66,694,848	66,694,955	107	Loss
DEL00027534	chr3	66,729,182	66,729,262	80	Loss
DEL00027543	chr3	66,814,854	66,815,049	195	Loss
DEL00027551	chr3	66,886,126	66,886,249	123	Loss
DEL00027553	chr3	66,925,658	66,926,827	1,169	Loss
DEL00027562	chr3	67,050,987	67,051,130	143	Loss
DEL00027571	chr3	67,166,455	67,167,342	887	Loss
DEL00027580	chr3	67,250,291	67,250,517	226	Loss
DUP00027585	chr3	67,369,711	67,382,096	12,385	Gain
DEL00027586	chr3	67,409,323	67,409,644	321	Loss
DEL00027592	chr3	67,527,625	67,527,786	161	Loss
DEL00027596	chr3	67,559,079	67,559,277	198	Loss
DEL00027601	chr3	67,626,734	67,628,925	2,191	Loss
DEL00027605	chr3	67,909,438	67,909,492	54	Loss
DEL00027609	chr3	67,949,436	67,949,506	70	Loss
DEL00027615	chr3	68,036,268	68,036,330	62	Loss
DEL00027620	chr3	68,113,432	68,113,484	52	Loss
DEL00027640	chr3	68,685,916	68,686,616	700	Loss
DEL00027642	chr3	68,749,626	68,749,681	55	Loss
DUP00027648	chr3	68,796,173	68,807,194	11,021	Gain
DEL00027650	chr3	68,881,860	68,881,932	72	Loss
DEL00027659	chr3	69,114,108	69,117,297	3,189	Loss
DEL00027663	chr3	69,323,414	69,323,477	63	Loss
DEL00027668	chr3	69,494,341	69,495,028	687	Loss
DEL00027674	chr3	69,588,624	69,590,303	1,679	Loss
DEL00027675	chr3	69,608,283	69,610,415	2,132	Loss
DEL00027685	chr3	69,677,458	69,678,162	704	Loss
DEL00027689	chr3	69,696,726	69,698,431	1,705	Loss
DEL00027700	chr3	69,766,671	69,768,433	1,762	Loss
DEL00027705	chr3	69,773,278	69,783,928	10,650	Loss

DEL00027709	chr3	69,798,999	69,801,184	2,185	Loss
DEL00027729	chr3	70,176,433	70,176,991	558	Loss
DEL00027737	chr3	70,245,387	70,246,972	1,585	Loss
DEL00027738	chr3	70,264,118	70,264,220	102	Loss
DEL00027740	chr3	70,271,062	70,271,740	678	Loss
DEL00027746	chr3	70,386,689	70,386,979	290	Loss
DEL00027753	chr3	70,508,075	70,509,132	1,057	Loss
DEL00027755	chr3	70,564,592	70,567,350	2,758	Loss
DEL00027756	chr3	70,568,293	70,568,741	448	Loss
DEL00027759	chr3	70,599,798	70,599,852	54	Loss
DEL00027765	chr3	70,708,819	70,709,076	257	Loss
DEL00027766	chr3	70,742,867	70,743,094	227	Loss
DEL00027768	chr3	70,760,378	70,760,470	92	Loss
DEL00027781	chr3	71,026,891	71,027,153	262	Loss
DEL00027783	chr3	71,058,633	71,058,782	149	Loss
DEL00027788	chr3	71,077,600	71,077,701	101	Loss
DEL00027794	chr3	71,161,558	71,161,965	407	Loss
DEL00027798	chr3	71,201,153	71,201,221	68	Loss
DEL00027800	chr3	71,226,031	71,227,086	1,055	Loss
DEL00027812	chr3	71,384,996	71,385,056	60	Loss
DEL00027814	chr3	71,436,376	71,436,468	92	Loss
DEL00027822	chr3	71,664,523	71,664,643	120	Loss
DEL00027824	chr3	71,748,265	71,748,393	128	Loss
DEL00027831	chr3	71,922,152	71,922,730	578	Loss
DEL00027832	chr3	71,929,982	71,930,460	478	Loss
DEL00027834	chr3	71,957,692	71,957,806	114	Loss
DEL00027837	chr3	72,030,270	72,030,632	362	Loss
DEL00027838	chr3	72,041,060	72,042,429	1,369	Loss
DEL00027841	chr3	72,115,534	72,115,586	52	Loss
DEL00027842	chr3	72,117,817	72,118,066	249	Loss
DEL00027845	chr3	72,142,870	72,143,372	502	Loss
DEL00027848	chr3	72,199,991	72,200,404	413	Loss
DEL00027866	chr3	72,327,145	72,328,131	986	Loss
DEL00027867	chr3	72,332,504	72,332,661	157	Loss
DEL00027868	chr3	72,343,123	72,343,290	167	Loss
DEL00027874	chr3	72,455,239	72,455,297	58	Loss
DEL00027879	chr3	72,519,548	72,520,332	784	Loss
DEL00027880	chr3	72,535,774	72,535,858	84	Loss
DEL00027907	chr3	72,645,187	72,645,488	301	Loss
DEL00027913	chr3	72,736,591	72,736,764	173	Loss
DEL00027920	chr3	72,772,466	72,772,576	110	Loss
DUP00027924	chr3	72,801,587	72,821,049	19,462	Gain
DEL00027929	chr3	72,863,165	72,864,832	1,667	Loss
DEL00027932	chr3	72,929,220	72,929,292	72	Loss
DEL00027933	chr3	72,951,159	72,951,507	348	Loss
DEL00027938	chr3	73,078,066	73,078,127	61	Loss
DUP00027939	chr3	73,078,782	73,080,937	2,155	Gain
DEL00027943	chr3	73,155,516	73,157,263	1,747	Loss
DEL00027948	chr3	73,170,910	73,171,106	196	Loss

DEL00027952	chr3	73,220,871	73,221,424	553	Loss
DEL00027953	chr3	73,225,219	73,228,015	2,796	Loss
DEL00027956	chr3	73,257,971	73,258,074	103	Loss
DEL00027963	chr3	73,455,955	73,456,512	557	Loss
DEL00027965	chr3	73,481,601	73,482,433	832	Loss
DEL00027967	chr3	73,536,146	73,536,215	69	Loss
DEL00027977	chr3	73,648,410	73,648,475	65	Loss
DEL00027981	chr3	73,749,992	73,750,107	115	Loss
DEL00027985	chr3	73,883,687	73,884,128	441	Loss
DEL00027987	chr3	73,944,664	73,945,282	618	Loss
DEL00027988	chr3	74,082,597	74,082,918	321	Loss
DEL00027989	chr3	74,088,040	74,088,331	291	Loss
DEL00028001	chr3	74,207,793	74,207,903	110	Loss
DEL00028003	chr3	74,257,349	74,258,035	686	Loss
DEL00028004	chr3	74,266,250	74,267,203	953	Loss
DUP00028007	chr3	74,302,011	74,325,533	23,522	Mixed
DEL00028019	chr3	74,507,071	74,507,672	601	Loss
DEL00028020	chr3	74,508,217	74,511,118	2,901	Loss
DEL00028028	chr3	74,731,394	74,731,500	106	Loss
DEL00028029	chr3	74,733,176	74,733,270	94	Loss
DEL00028037	chr3	74,975,198	74,975,290	92	Loss
DEL00028040	chr3	75,121,995	75,122,128	133	Loss
DEL00028048	chr3	75,369,577	75,369,963	386	Loss
DEL00028050	chr3	75,373,091	75,373,143	52	Loss
DEL00028052	chr3	75,378,428	75,378,753	325	Loss
DEL00028054	chr3	75,387,864	75,388,877	1,013	Loss
DEL00028056	chr3	75,429,435	75,430,288	853	Loss
DEL00028057	chr3	75,438,011	75,438,080	69	Loss
DEL00028066	chr3	75,516,895	75,516,951	56	Loss
DEL00028067	chr3	75,524,922	75,525,107	185	Loss
DEL00028069	chr3	75,531,818	75,532,768	950	Loss
DEL00028075	chr3	75,558,109	75,558,380	271	Loss
DEL00028078	chr3	75,571,766	75,572,217	451	Loss
DEL00028087	chr3	75,643,695	75,644,856	1,161	Loss
DEL00028088	chr3	75,645,217	75,645,519	302	Loss
DEL00028090	chr3	75,697,223	75,697,566	343	Loss
DEL00028093	chr3	75,728,384	75,728,498	114	Loss
DEL00028107	chr3	75,863,796	75,864,490	694	Loss
DEL00028108	chr3	75,941,614	75,941,674	60	Loss
DEL00028110	chr3	76,018,847	76,018,907	60	Loss
DEL00028126	chr3	76,215,589	76,215,661	72	Loss
DEL00028129	chr3	76,246,763	76,247,044	281	Loss
DEL00028135	chr3	76,378,596	76,378,682	86	Loss
DUP00028136	chr3	76,440,859	76,446,516	5,657	Gain
DEL00028138	chr3	76,448,851	76,448,957	106	Loss
DEL00028143	chr3	76,526,677	76,526,837	160	Loss
DEL00028145	chr3	76,531,007	76,531,063	56	Loss
DEL00028153	chr3	76,595,046	76,595,419	373	Loss
DEL00028156	chr3	76,676,709	76,676,813	104	Loss

DEL00028171	chr3	76,844,809	76,845,267	458	Loss
DEL00028175	chr3	76,926,051	76,926,315	264	Loss
DEL00028184	chr3	77,122,200	77,122,268	68	Loss
DEL00028201	chr3	77,446,636	77,448,285	1,649	Loss
DEL00028204	chr3	77,517,104	77,517,842	738	Loss
DEL00028207	chr3	77,580,943	77,581,356	413	Loss
DEL00028219	chr3	77,769,883	77,770,337	454	Loss
DEL00028220	chr3	77,786,609	77,786,840	231	Loss
DEL00028226	chr3	77,883,891	77,884,000	109	Loss
DEL00028229	chr3	77,906,250	77,906,330	80	Loss
DEL00028233	chr3	77,938,256	77,938,726	470	Loss
DEL00028246	chr3	78,089,940	78,089,995	55	Loss
DUP00028250	chr3	78,147,635	78,147,957	322	Gain
DEL00028251	chr3	78,166,224	78,166,299	75	Loss
DEL00028255	chr3	78,216,395	78,216,970	575	Loss
DUP00028256	chr3	78,229,143	78,229,856	713	Gain
DEL00028257	chr3	78,231,877	78,232,033	156	Loss
DEL00028258	chr3	78,246,353	78,246,724	371	Loss
DEL00028261	chr3	78,260,173	78,260,225	52	Loss
DUP00028263	chr3	78,277,181	78,277,246	65	Gain
DEL00028264	chr3	78,302,689	78,303,141	452	Loss
DEL00028272	chr3	78,500,494	78,500,772	278	Loss
DEL00028273	chr3	78,526,118	78,526,252	134	Loss
DEL00028274	chr3	78,526,430	78,528,307	1,877	Loss
DEL00028283	chr3	78,624,800	78,625,527	727	Loss
DEL00028285	chr3	78,634,219	78,634,622	403	Loss
DEL00028289	chr3	78,662,109	78,663,081	972	Loss
DEL00028293	chr3	78,728,789	78,728,889	100	Loss
DEL00028295	chr3	78,737,074	78,737,278	204	Loss
DEL00028296	chr3	78,752,289	78,752,355	66	Loss
DEL00028301	chr3	78,848,847	78,849,009	162	Loss
DEL00028303	chr3	78,863,678	78,864,198	520	Loss
DEL00028304	chr3	78,867,290	78,867,410	120	Loss
DEL00028305	chr3	78,869,077	78,869,144	67	Loss
DEL00028316	chr3	79,080,364	79,080,435	71	Loss
DEL00028318	chr3	79,087,480	79,087,566	86	Loss
DEL00028323	chr3	79,155,365	79,155,673	308	Loss
DUP00028326	chr3	79,204,165	79,204,281	116	Gain
DEL00028327	chr3	79,229,412	79,229,493	81	Loss
DEL00028337	chr3	79,321,359	79,321,979	620	Loss
DEL00028341	chr3	79,402,011	79,402,255	244	Loss
DEL00028343	chr3	79,443,691	79,443,771	80	Loss
DEL00028352	chr3	79,564,154	79,565,741	1,587	Loss
DEL00028357	chr3	79,691,459	79,692,476	1,017	Loss
DEL00028360	chr3	79,714,385	79,714,436	51	Loss
DEL00028361	chr3	79,729,531	79,729,647	116	Loss
DEL00028363	chr3	79,739,433	79,739,852	419	Loss
DEL00028365	chr3	79,760,796	79,760,927	131	Loss
DEL00028371	chr3	79,785,768	79,785,865	97	Loss

DEL00028379	chr3	79,815,621	79,817,305	1,684	Loss
DEL00028382	chr3	79,872,063	79,872,122	59	Loss
DEL00028385	chr3	79,891,998	79,892,438	440	Loss
DEL00028396	chr3	80,054,846	80,055,294	448	Loss
DEL00028398	chr3	80,112,714	80,112,981	267	Loss
DEL00028403	chr3	80,137,797	80,138,754	957	Loss
DEL00028408	chr3	80,179,662	80,180,056	394	Loss
DEL00028410	chr3	80,184,119	80,184,248	129	Loss
DEL00028413	chr3	80,208,932	80,209,459	527	Loss
DEL00028419	chr3	80,260,144	80,260,632	488	Loss
DEL00028421	chr3	80,263,835	80,263,934	99	Loss
DEL00028422	chr3	80,292,888	80,292,940	52	Loss
DEL00028423	chr3	80,311,488	80,311,665	177	Loss
DEL00028424	chr3	80,312,085	80,312,890	805	Loss
DEL00028431	chr3	80,453,733	80,453,796	63	Loss
DEL00028440	chr3	80,544,441	80,544,521	80	Loss
DEL00028441	chr3	80,546,374	80,546,452	78	Loss
DEL00028458	chr3	80,728,044	80,728,494	450	Loss
DEL00028459	chr3	80,749,122	80,749,176	54	Loss
DEL00028463	chr3	80,814,582	80,815,054	472	Loss
DUP00028465	chr3	80,851,396	80,853,337	1,941	Gain
DEL00028468	chr3	80,869,584	80,869,803	219	Loss
DEL00028469	chr3	80,887,601	80,887,676	75	Loss
DEL00028474	chr3	80,916,767	80,917,642	875	Loss
DEL00028483	chr3	80,999,781	80,999,844	63	Loss
DEL00028488	chr3	81,019,600	81,021,609	2,009	Loss
DEL00028492	chr3	81,137,379	81,137,771	392	Loss
DEL00028495	chr3	81,183,838	81,183,904	66	Loss
DEL00028499	chr3	81,234,908	81,234,959	51	Loss
DEL00028502	chr3	81,278,197	81,278,700	503	Loss
DEL00028504	chr3	81,322,836	81,322,895	59	Loss
DEL00028512	chr3	81,366,600	81,366,692	92	Loss
DEL00028520	chr3	81,494,268	81,494,326	58	Loss
DEL00028521	chr3	81,560,432	81,560,619	187	Loss
DEL00028525	chr3	81,678,585	81,686,002	7,417	Loss
DEL00028526	chr3	81,702,490	81,703,446	956	Loss
DEL00028536	chr3	81,892,670	81,892,726	56	Loss
DEL00028543	chr3	81,993,548	81,993,766	218	Loss
DEL00028545	chr3	82,026,462	82,026,537	75	Loss
DEL00028547	chr3	82,044,023	82,044,401	378	Loss
DEL00028548	chr3	82,053,771	82,053,964	193	Loss
DEL00028551	chr3	82,085,137	82,085,241	104	Loss
DEL00028552	chr3	82,094,382	82,095,790	1,408	Loss
DEL00028553	chr3	82,106,087	82,106,265	178	Loss
DEL00028555	chr3	82,135,796	82,135,962	166	Loss
DEL00028561	chr3	82,185,136	82,185,189	53	Loss
DEL00028563	chr3	82,281,128	82,281,454	326	Loss
DEL00028564	chr3	82,332,945	82,333,007	62	Loss
DEL00028569	chr3	82,508,800	82,510,245	1,445	Loss

DEL00028574	chr3	82,572,409	82,573,915	1,506	Loss
DEL00028575	chr3	82,604,474	82,605,029	555	Loss
DEL00028576	chr3	82,628,345	82,629,400	1,055	Loss
DEL00028579	chr3	82,717,140	82,720,095	2,955	Loss
DUP00028584	chr3	82,752,608	82,752,728	120	Gain
DEL00028594	chr3	82,816,038	82,816,335	297	Loss
DEL00028598	chr3	82,946,135	82,946,587	452	Loss
DEL00028600	chr3	82,950,737	82,951,150	413	Loss
DEL00028607	chr3	83,028,336	83,028,427	91	Loss
DEL00028608	chr3	83,036,712	83,037,519	807	Loss
DEL00028613	chr3	83,098,465	83,098,521	56	Loss
DEL00028615	chr3	83,108,181	83,108,393	212	Loss
DEL00028621	chr3	83,333,477	83,333,546	69	Loss
DEL00028627	chr3	83,476,014	83,476,904	890	Loss
DEL00028629	chr3	83,564,700	83,565,616	916	Loss
DEL00028633	chr3	83,639,648	83,640,258	610	Loss
DEL00028634	chr3	83,640,761	83,641,278	517	Loss
DEL00028638	chr3	83,659,260	83,659,314	54	Loss
DEL00028641	chr3	83,675,947	83,680,613	4,666	Loss
DEL00028653	chr3	83,914,297	83,914,846	549	Loss
DEL00028654	chr3	83,917,890	83,918,288	398	Loss
DEL00028660	chr3	84,021,165	84,021,263	98	Loss
DEL00028661	chr3	84,028,112	84,031,079	2,967	Loss
DEL00028668	chr3	84,196,876	84,197,796	920	Loss
DEL00028682	chr3	84,540,242	84,540,870	628	Loss
DEL00028684	chr3	84,608,365	84,608,866	501	Loss
DEL00028685	chr3	84,628,894	84,629,490	596	Loss
DUP00028696	chr3	84,834,333	84,834,414	81	Gain
DEL00028698	chr3	84,893,763	84,899,588	5,825	Loss
DEL00028702	chr3	84,943,014	84,944,217	1,203	Loss
DEL00028715	chr3	85,352,120	85,356,185	4,065	Loss
DUP00028718	chr3	85,429,241	85,450,177	20,936	Gain
DEL00028725	chr3	85,518,608	85,518,663	55	Loss
DEL00028726	chr3	85,616,453	85,616,618	165	Loss
DEL00028732	chr3	85,727,672	85,727,791	119	Loss
DEL00028733	chr3	85,734,804	85,734,879	75	Loss
DEL00028734	chr3	85,743,846	85,744,038	192	Loss
DEL00028735	chr3	85,747,759	85,747,833	74	Loss
DEL00028749	chr3	85,832,268	85,834,964	2,696	Loss
DEL00028751	chr3	85,836,141	85,836,486	345	Loss
DEL00028755	chr3	85,869,800	85,870,965	1,165	Loss
DEL00028757	chr3	85,873,992	85,876,890	2,898	Loss
DEL00028762	chr3	85,917,573	85,917,709	136	Loss
DEL00028763	chr3	85,942,230	85,942,404	174	Loss
DEL00028778	chr3	86,301,300	86,301,545	245	Loss
DEL00028781	chr3	86,336,516	86,336,911	395	Loss
DEL00028783	chr3	86,430,943	86,431,112	169	Loss
DEL00028784	chr3	86,465,178	86,465,750	572	Loss
DEL00028785	chr3	86,501,540	86,502,723	1,183	Loss

DUP00028801	chr3	86,702,545	86,713,680	11,135	Gain
DEL00028805	chr3	86,796,958	86,797,398	440	Loss
DEL00028807	chr3	86,814,271	86,814,676	405	Loss
DEL00028812	chr3	86,824,337	86,824,430	93	Loss
DEL00028816	chr3	86,890,575	86,890,715	140	Loss
DEL00028818	chr3	86,930,436	86,931,117	681	Loss
DEL00028832	chr3	87,231,125	87,231,237	112	Loss
DEL00028839	chr3	87,307,481	87,307,533	52	Loss
DEL00028848	chr3	87,470,860	87,470,975	115	Loss
DEL00028854	chr3	87,593,640	87,593,801	161	Loss
DEL00028858	chr3	87,703,719	87,703,931	212	Loss
DEL00028861	chr3	87,719,741	87,720,438	697	Loss
DEL00028862	chr3	87,726,217	87,726,545	328	Loss
DEL00028867	chr3	87,800,712	87,800,770	58	Loss
DEL00028873	chr3	88,009,432	88,010,639	1,207	Loss
DEL00028879	chr3	88,142,267	88,143,238	971	Loss
DEL00028887	chr3	88,206,243	88,206,529	286	Loss
DEL00028889	chr3	88,245,316	88,246,452	1,136	Loss
DEL00028892	chr3	88,253,042	88,253,754	712	Loss
DEL00028894	chr3	88,283,729	88,284,564	835	Loss
DEL00028896	chr3	88,291,271	88,291,398	127	Loss
DEL00028918	chr3	88,313,428	88,327,137	13,709	Loss
DEL00028921	chr3	88,333,141	88,333,623	482	Loss
DEL00028923	chr3	88,377,204	88,377,260	56	Loss
DEL00028925	chr3	88,426,635	88,427,638	1,003	Loss
DEL00028927	chr3	88,439,711	88,440,033	322	Loss
DEL00028928	chr3	88,474,139	88,474,261	122	Loss
DEL00028929	chr3	88,475,473	88,475,563	90	Loss
DEL00028933	chr3	88,548,593	88,548,674	81	Loss
DUP00028945	chr3	88,777,426	88,777,932	506	Gain
DEL00028949	chr3	88,799,905	88,800,961	1,056	Loss
DUP00028958	chr3	89,014,236	89,014,526	290	Gain
DUP00028961	chr3	89,143,632	89,155,395	11,763	Gain
DEL00028965	chr3	89,287,635	89,288,268	633	Loss
DEL00028969	chr3	89,314,709	89,315,037	328	Loss
DEL00028970	chr3	89,321,258	89,322,156	898	Loss
DEL00028974	chr3	89,388,970	89,389,181	211	Loss
DEL00028984	chr3	89,457,538	89,457,641	103	Loss
DEL00028985	chr3	89,504,183	89,504,320	137	Loss
DEL00028990	chr3	89,561,059	89,562,192	1,133	Loss
DEL00028997	chr3	89,617,165	89,617,879	714	Loss
DEL00029001	chr3	89,689,993	89,690,558	565	Loss
DEL00029012	chr3	89,803,352	89,805,914	2,562	Loss
DEL00029013	chr3	89,819,219	89,819,732	513	Loss
DEL00029017	chr3	89,865,296	89,867,790	2,494	Loss
DEL00029025	chr3	89,921,189	89,921,994	805	Loss
DEL00029028	chr3	89,962,853	89,964,101	1,248	Loss
DEL00029029	chr3	89,968,491	89,969,851	1,360	Loss
DEL00029035	chr3	90,013,665	90,013,873	208	Loss

DEL00029036	chr3	90,023,530	90,023,624	94	Loss
DEL00029039	chr3	90,082,636	90,084,681	2,045	Loss
DEL00029046	chr3	90,130,784	90,132,121	1,337	Loss
DEL00029047	chr3	90,136,840	90,137,047	207	Loss
DUP00029050	chr3	90,191,892	90,206,885	14,993	Gain
DEL00029055	chr3	90,260,561	90,260,714	153	Loss
DEL00029062	chr3	90,327,634	90,328,181	547	Loss
DEL00029064	chr3	90,328,455	90,328,577	122	Loss
DEL00029065	chr3	90,334,086	90,334,320	234	Loss
DEL00029067	chr3	90,363,531	90,363,896	365	Loss
DEL00029068	chr3	90,366,243	90,366,315	72	Loss
DEL00029069	chr3	90,371,067	90,371,232	165	Loss
DEL00029070	chr3	90,373,050	90,373,348	298	Loss
DEL00029085	chr3	90,559,003	90,559,139	136	Loss
DEL00029087	chr3	90,590,781	90,590,930	149	Loss
DEL00029095	chr3	90,719,692	90,720,213	521	Loss
DEL00029097	chr3	90,744,194	90,744,447	253	Loss
DEL00029101	chr3	90,849,574	90,849,646	72	Loss
DEL00029103	chr3	90,863,618	90,863,843	225	Loss
DEL00029110	chr3	90,945,157	90,945,231	74	Loss
DEL00029113	chr3	90,971,665	90,972,061	396	Loss
DEL00029119	chr3	91,048,266	91,048,323	57	Loss
DUP00029139	chr3	91,330,659	91,330,853	194	Gain
DEL00029140	chr3	91,340,524	91,340,621	97	Loss
DEL00029142	chr3	91,364,609	91,364,673	64	Loss
DEL00029145	chr3	91,407,610	91,407,712	102	Loss
DEL00029161	chr3	91,645,091	91,645,172	81	Loss
DEL00029165	chr3	91,656,551	91,656,611	60	Loss
DEL00029171	chr3	91,761,412	91,761,469	57	Loss
DEL00029191	chr3	91,966,354	91,966,452	98	Loss
DEL00029193	chr3	91,981,477	91,981,759	282	Loss
DEL00029200	chr3	92,060,418	92,060,872	454	Loss
DEL00029201	chr3	92,069,199	92,070,121	922	Loss
DEL00029204	chr3	92,085,376	92,086,050	674	Loss
DEL00029205	chr3	92,089,099	92,089,155	56	Loss
DEL00029206	chr3	92,093,297	92,093,374	77	Loss
DEL00029213	chr3	92,143,375	92,145,296	1,921	Loss
DEL00029214	chr3	92,165,571	92,167,226	1,655	Loss
DEL00029216	chr3	92,189,847	92,193,988	4,141	Loss
DEL00029217	chr3	92,196,283	92,220,006	23,723	Loss
DEL00029221	chr3	92,220,881	92,222,459	1,578	Loss
DEL00029222	chr3	92,253,416	92,254,656	1,240	Loss
DEL00029225	chr3	92,307,043	92,307,234	191	Loss
DEL00029226	chr3	92,322,934	92,322,990	56	Loss
DEL00029232	chr3	92,451,333	92,451,831	498	Loss
DEL00029235	chr3	92,458,449	92,458,863	414	Loss
DEL00029237	chr3	92,492,253	92,493,052	799	Loss
DEL00029241	chr3	92,534,996	92,535,152	156	Loss
DEL00029244	chr3	92,607,147	92,607,389	242	Loss

DEL00029246	chr3	92,643,419	92,644,530	1,111	Loss
DEL00029249	chr3	92,698,866	92,698,922	56	Loss
DEL00029254	chr3	92,763,402	92,763,692	290	Loss
DEL00029255	chr3	92,801,587	92,801,653	66	Loss
DEL00029261	chr3	92,850,013	92,850,067	54	Loss
DEL00029267	chr3	93,025,591	93,025,841	250	Loss
DEL00029273	chr3	93,072,792	93,073,679	887	Loss
DEL00029274	chr3	93,075,795	93,076,031	236	Loss
DEL00029276	chr3	93,118,598	93,118,659	61	Loss
DEL00029282	chr3	93,234,218	93,234,844	626	Loss
DEL00029285	chr3	93,431,008	93,431,061	53	Loss
DEL00029286	chr3	93,481,366	93,481,424	58	Loss
DEL00029291	chr3	93,536,410	93,537,409	999	Loss
DEL00029293	chr3	93,577,884	93,578,261	377	Loss
DEL00029299	chr3	93,617,786	93,617,937	151	Loss
DEL00029301	chr3	93,635,646	93,635,708	62	Loss
DEL00029304	chr3	93,650,372	93,650,813	441	Loss
DEL00029313	chr3	93,723,762	93,726,084	2,322	Loss
DEL00029323	chr3	93,833,621	93,835,089	1,468	Loss
DEL00029325	chr3	93,854,868	93,854,938	70	Loss
DEL00029334	chr3	93,942,676	93,945,082	2,406	Loss
DEL00029340	chr3	93,968,894	93,970,852	1,958	Loss
DEL00029347	chr3	94,052,122	94,052,187	65	Loss
DEL00029348	chr3	94,080,097	94,080,260	163	Loss
DEL00029350	chr3	94,133,879	94,133,930	51	Loss
DEL00029356	chr3	94,147,811	94,147,928	117	Loss
DEL00029360	chr3	94,180,919	94,181,154	235	Loss
DEL00029362	chr3	94,194,696	94,195,631	935	Loss
DEL00029365	chr3	94,242,504	94,243,483	979	Loss
DEL00029370	chr3	94,366,015	94,366,118	103	Loss
DEL00029379	chr3	94,551,801	94,552,415	614	Loss
DEL00029382	chr3	94,607,886	94,607,938	52	Loss
DEL00029390	chr3	94,621,147	94,621,219	72	Loss
DEL00029394	chr3	94,642,543	94,642,594	51	Loss
DEL00029416	chr3	94,974,209	94,974,282	73	Loss
DEL00029417	chr3	94,993,900	94,993,989	89	Loss
DEL00029418	chr3	94,996,713	94,997,533	820	Loss
DEL00029426	chr3	95,096,980	95,097,090	110	Loss
DEL00029429	chr3	95,130,314	95,130,491	177	Loss
DEL00029435	chr3	95,213,324	95,214,049	725	Loss
DEL00029438	chr3	95,286,878	95,286,933	55	Loss
DEL00029439	chr3	95,304,235	95,304,691	456	Loss
DEL00029445	chr3	95,459,098	95,459,203	105	Loss
DEL00029450	chr3	95,642,581	95,642,639	58	Loss
DEL00029455	chr3	95,736,281	95,736,690	409	Loss
DEL00029457	chr3	95,752,959	95,753,491	532	Loss
DEL00029466	chr3	95,776,731	95,776,787	56	Loss
DEL00029467	chr3	95,801,723	95,801,779	56	Loss
DEL00029473	chr3	95,859,675	95,859,971	296	Loss

DEL00029474	chr3	95,897,423	95,898,208	785	Loss
DEL00029478	chr3	95,950,525	95,950,598	73	Loss
DEL00029480	chr3	95,958,001	95,959,139	1,138	Loss
DEL00029483	chr3	95,992,706	95,992,848	142	Loss
DEL00029488	chr3	96,111,891	96,112,104	213	Loss
DEL00029494	chr3	96,219,018	96,219,459	441	Loss
DEL00029499	chr3	96,331,692	96,331,814	122	Loss
DEL00029505	chr3	96,371,099	96,371,227	128	Loss
DEL00029511	chr3	96,417,370	96,417,426	56	Loss
DEL00029512	chr3	96,471,996	96,472,070	74	Loss
DEL00029513	chr3	96,476,854	96,476,906	52	Loss
DEL00029517	chr3	96,504,864	96,505,217	353	Loss
DEL00029520	chr3	96,537,723	96,537,898	175	Loss
DEL00029524	chr3	96,576,927	96,577,300	373	Loss
DEL00029530	chr3	96,655,332	96,655,846	514	Loss
DEL00029532	chr3	96,675,744	96,676,254	510	Loss
DEL00029536	chr3	96,698,061	96,702,287	4,226	Loss
DUP00029538	chr3	96,753,127	96,753,280	153	Gain
DEL00029542	chr3	96,819,240	96,819,703	463	Loss
DEL00029545	chr3	96,863,741	96,863,960	219	Loss
DEL00029558	chr3	96,989,141	96,991,844	2,703	Loss
DEL00029559	chr3	96,996,540	96,997,037	497	Loss
DUP00029560	chr3	97,019,670	97,019,733	63	Gain
DEL00029561	chr3	97,040,957	97,041,036	79	Loss
DEL00029564	chr3	97,117,906	97,118,031	125	Loss
DEL00029569	chr3	97,159,798	97,159,936	138	Loss
DEL00029577	chr3	97,289,449	97,289,561	112	Loss
DEL00029583	chr3	97,352,480	97,353,293	813	Loss
DEL00029587	chr3	97,474,942	97,475,017	75	Loss
DEL00029594	chr3	97,599,287	97,599,551	264	Loss
DEL00029597	chr3	97,628,944	97,630,714	1,770	Loss
DEL00029604	chr3	97,686,753	97,686,804	51	Loss
DEL00029612	chr3	97,885,252	97,885,365	113	Loss
DEL00029613	chr3	97,913,530	97,913,582	52	Loss
DEL00029614	chr3	97,914,222	97,915,142	920	Loss
DEL00029615	chr3	97,933,436	97,934,370	934	Loss
DUP00029621	chr3	97,995,982	98,008,835	12,853	Gain
DEL00029634	chr3	98,168,077	98,168,409	332	Loss
DEL00029635	chr3	98,181,353	98,182,152	799	Loss
DEL00029638	chr3	98,290,930	98,291,073	143	Loss
DEL00029646	chr3	98,378,589	98,378,812	223	Loss
DEL00029653	chr3	98,480,553	98,481,617	1,064	Loss
DEL00029660	chr3	98,662,261	98,662,557	296	Loss
DEL00029663	chr3	98,672,163	98,672,609	446	Loss
DEL00029664	chr3	98,697,385	98,697,507	122	Loss
DEL00029674	chr3	98,785,288	98,785,365	77	Loss
DEL00029676	chr3	98,790,231	98,790,315	84	Loss
DEL00029677	chr3	98,809,879	98,810,059	180	Loss
DEL00029686	chr3	98,904,988	98,906,692	1,704	Loss

DEL00029688	chr3	98,935,857	98,935,997	140	Loss
DEL00029691	chr3	99,023,594	99,023,653	59	Loss
DEL00029695	chr3	99,065,857	99,066,863	1,006	Loss
DEL00029697	chr3	99,131,880	99,132,227	347	Loss
DEL00029699	chr3	99,151,717	99,152,231	514	Loss
DEL00029702	chr3	99,173,903	99,173,981	78	Loss
DEL00029704	chr3	99,188,734	99,189,492	758	Loss
DEL00029710	chr3	99,247,628	99,247,722	94	Loss
DEL00029713	chr3	99,336,503	99,339,109	2,606	Loss
DEL00029714	chr3	99,351,343	99,351,459	116	Loss
DEL00029722	chr3	99,476,611	99,476,694	83	Loss
DEL00029737	chr3	99,685,758	99,686,385	627	Loss
DEL00029741	chr3	99,759,396	99,759,483	87	Loss
DEL00029743	chr3	99,782,979	99,790,758	7,779	Loss
DEL00029761	chr3	100,006,635	100,007,480	845	Loss
DEL00029771	chr3	100,125,358	100,126,036	678	Loss
DEL00029773	chr3	100,141,312	100,141,419	107	Loss
DEL00029775	chr3	100,173,816	100,174,402	586	Loss
DEL00029785	chr3	100,305,425	100,305,535	110	Loss
DEL00029788	chr3	100,341,771	100,342,339	568	Loss
DEL00029789	chr3	100,354,926	100,355,683	757	Loss
DEL00029790	chr3	100,363,627	100,364,236	609	Loss
DEL00029793	chr3	100,375,326	100,376,169	843	Loss
DEL00029796	chr3	100,394,476	100,394,913	437	Loss
DEL00029798	chr3	100,404,374	100,405,495	1,121	Loss
DEL00029802	chr3	100,440,392	100,440,972	580	Loss
DEL00029812	chr3	100,547,527	100,548,057	530	Loss
DEL00029838	chr3	100,883,813	100,884,226	413	Loss
DEL00029842	chr3	100,944,969	100,945,523	554	Loss
DEL00029849	chr3	101,011,876	101,011,975	99	Loss
DEL00029852	chr3	101,084,332	101,084,385	53	Loss
DEL00029861	chr3	101,103,488	101,103,911	423	Loss
DEL00029862	chr3	101,104,693	101,105,011	318	Loss
DEL00029865	chr3	101,129,496	101,129,550	54	Loss
DEL00029869	chr3	101,157,521	101,157,640	119	Loss
DEL00029871	chr3	101,193,359	101,193,894	535	Loss
DEL00029874	chr3	101,211,638	101,211,898	260	Loss
DEL00029884	chr3	101,364,577	101,365,624	1,047	Loss
DEL00029887	chr3	101,390,071	101,390,122	51	Loss
DEL00029904	chr3	101,715,491	101,715,594	103	Loss
DEL00029907	chr3	101,742,031	101,742,587	556	Loss
DEL00029910	chr3	101,819,810	101,820,364	554	Loss
DEL00029923	chr3	102,073,695	102,073,816	121	Loss
DEL00029924	chr3	102,085,158	102,085,418	260	Loss
DEL00029926	chr3	102,154,795	102,155,145	350	Loss
DEL00029929	chr3	102,211,842	102,212,279	437	Loss
DEL00029941	chr3	102,500,197	102,500,503	306	Loss
DEL00029944	chr3	102,540,311	102,540,812	501	Loss
DEL00029946	chr3	102,548,985	102,549,087	102	Loss

DEL00029961	chr3	102,748,884	102,749,082	198	Loss
DEL00029964	chr3	102,852,020	102,852,189	169	Loss
DEL00029966	chr3	102,906,593	102,906,704	111	Loss
DEL00029975	chr3	103,009,333	103,010,214	881	Loss
DEL00029976	chr3	103,018,260	103,018,450	190	Loss
DEL00029978	chr3	103,022,279	103,022,608	329	Loss
DEL00029979	chr3	103,030,140	103,031,198	1,058	Loss
DEL00029986	chr3	103,151,798	103,151,932	134	Loss
DEL00029991	chr3	103,268,577	103,269,324	747	Loss
DEL00029992	chr3	103,367,441	103,368,136	695	Loss
DEL00029994	chr3	103,387,339	103,387,868	529	Loss
DEL00029996	chr3	103,388,220	103,388,282	62	Loss
DEL00029997	chr3	103,388,953	103,389,430	477	Loss
DEL00030007	chr3	103,746,314	103,746,368	54	Loss
DEL00030008	chr3	103,867,519	103,867,737	218	Loss
DEL00030012	chr3	103,934,694	103,934,893	199	Loss
DEL00030014	chr3	104,012,827	104,012,954	127	Loss
DEL00030015	chr3	104,026,870	104,026,992	122	Loss
DEL00030016	chr3	104,047,561	104,047,613	52	Loss
DEL00030017	chr3	104,062,779	104,062,834	55	Loss
DEL00030021	chr3	104,127,916	104,128,350	434	Loss
DEL00030031	chr3	104,142,381	104,142,567	186	Loss
DEL00030051	chr3	104,301,324	104,301,411	87	Loss
DEL00030052	chr3	104,309,755	104,309,840	85	Loss
DEL00030059	chr3	104,414,167	104,414,228	61	Loss
DEL00030094	chr3	104,505,324	104,505,723	399	Loss
DEL00030116	chr3	104,544,737	104,545,083	346	Loss
DUP00030120	chr3	104,549,659	104,549,807	148	Gain
DEL00030121	chr3	104,550,158	104,550,774	616	Loss
DEL00030124	chr3	104,566,382	104,566,433	51	Loss
DEL00030132	chr3	104,699,361	104,699,436	75	Loss
DEL00030146	chr3	104,807,660	104,807,789	129	Loss
DEL00030147	chr3	104,823,678	104,824,009	331	Loss
DEL00030158	chr3	104,899,239	104,899,299	60	Loss
DEL00030160	chr3	104,927,895	104,927,969	74	Loss
DEL00030167	chr3	104,951,888	104,989,437	37,549	Mixed
DEL00030180	chr3	105,065,337	105,065,423	86	Loss
DEL00030188	chr3	105,214,428	105,214,493	65	Loss
DEL00030196	chr3	105,307,049	105,307,138	89	Loss
DEL00030211	chr3	105,549,196	105,549,266	70	Loss
DEL00030230	chr3	105,590,666	105,591,308	642	Loss
DEL00030239	chr3	105,711,317	105,711,846	529	Loss
DEL00030242	chr3	105,732,914	105,733,230	316	Loss
DEL00030249	chr3	105,801,464	105,801,650	186	Loss
DEL00030258	chr3	105,918,872	105,919,912	1,040	Loss
DEL00030259	chr3	105,934,215	105,935,115	900	Loss
DEL00030261	chr3	105,967,941	105,968,521	580	Loss
DEL00030274	chr3	106,164,320	106,164,455	135	Loss
DEL00030277	chr3	106,276,023	106,276,172	149	Loss

DEL00030278	chr3	106,321,255	106,321,391	136	Loss
DEL00030282	chr3	106,396,841	106,396,915	74	Loss
DEL00030290	chr3	106,450,629	106,450,725	96	Loss
DUP00030291	chr3	106,486,742	106,503,545	16,803	Mixed
DEL00030296	chr3	106,577,009	106,577,102	93	Loss
DEL00030303	chr3	106,643,151	106,643,487	336	Loss
DEL00030322	chr3	106,868,981	106,869,320	339	Loss
DEL00030325	chr3	106,898,841	106,898,929	88	Loss
DEL00030327	chr3	106,962,192	106,962,451	259	Loss
DEL00030343	chr3	107,052,255	107,052,324	69	Loss
DUP00030346	chr3	107,056,890	107,059,139	2,249	Gain
DEL00030352	chr3	107,096,800	107,096,896	96	Loss
DEL00030362	chr3	107,154,637	107,154,787	150	Loss
DEL00030377	chr3	107,391,184	107,391,927	743	Loss
DEL00030381	chr3	107,407,163	107,407,251	88	Loss
DEL00030382	chr3	107,415,291	107,415,561	270	Loss
DEL00030384	chr3	107,419,019	107,419,150	131	Loss
DEL00030393	chr3	107,547,782	107,548,656	874	Loss
DEL00030395	chr3	107,582,339	107,582,564	225	Loss
DEL00030396	chr3	107,593,075	107,593,148	73	Loss
DEL00030405	chr3	107,645,012	107,645,222	210	Loss
DEL00030406	chr3	107,659,836	107,659,924	88	Loss
DEL00030410	chr3	107,749,894	107,750,459	565	Loss
DEL00030411	chr3	107,751,760	107,752,512	752	Loss
DEL00030418	chr3	107,861,841	107,862,059	218	Loss
DEL00030419	chr3	107,881,998	107,882,072	74	Loss
DEL00030421	chr3	107,928,045	107,928,205	160	Loss
DEL00030424	chr3	107,938,040	107,938,173	133	Loss
DEL00030425	chr3	107,992,177	107,992,627	450	Loss
DEL00030430	chr3	108,044,099	108,044,166	67	Loss
DEL00030439	chr3	108,154,883	108,156,109	1,226	Loss
DEL00030440	chr3	108,157,930	108,158,088	158	Loss
DEL00030451	chr3	108,397,260	108,397,635	375	Loss
DEL00030462	chr3	108,550,920	108,551,744	824	Loss
DEL00030470	chr3	108,600,441	108,600,971	530	Loss
DEL00030473	chr3	108,617,985	108,618,496	511	Loss
DEL00030482	chr3	108,693,843	108,694,746	903	Loss
DEL00030487	chr3	108,737,022	108,737,418	396	Loss
DEL00030502	chr3	108,867,158	108,867,403	245	Loss
DEL00030510	chr3	108,891,779	108,891,860	81	Loss
DEL00030513	chr3	108,899,825	108,900,107	282	Loss
DEL00030530	chr3	109,088,896	109,089,082	186	Loss
DEL00030531	chr3	109,099,946	109,100,104	158	Loss
DEL00030534	chr3	109,138,140	109,138,210	70	Loss
DEL00030538	chr3	109,180,666	109,181,561	895	Loss
DEL00030549	chr3	109,313,952	109,314,116	164	Loss
DEL00030561	chr3	109,392,357	109,392,428	71	Loss
DEL00030570	chr3	109,483,192	109,483,797	605	Loss
DEL00030575	chr3	109,511,071	109,511,286	215	Loss

DEL00030602	chr3	109,668,980	109,669,555	575	Loss
DEL00030603	chr3	109,671,251	109,671,321	70	Loss
DEL00030706	chr3	109,850,385	109,851,199	814	Loss
DEL00030728	chr3	109,939,557	109,939,986	429	Loss
DEL00030735	chr3	109,960,754	109,961,297	543	Loss
DEL00030738	chr3	109,963,196	109,963,734	538	Loss
DEL00030786	chr3	110,165,957	110,166,492	535	Loss
DEL00030789	chr3	110,184,373	110,185,175	802	Loss
DEL00030795	chr3	110,258,172	110,258,609	437	Loss
DEL00030798	chr3	110,280,083	110,280,502	419	Loss
DEL00030802	chr3	110,319,321	110,319,997	676	Loss
DEL00030810	chr3	110,407,423	110,407,593	170	Loss
DEL00030818	chr3	110,526,540	110,527,982	1,442	Loss
DEL00030820	chr3	110,536,069	110,536,132	63	Loss
DEL00030857	chr4	27,230	27,288	58	Loss
DEL00030867	chr4	307,762	308,316	554	Loss
DEL00030877	chr4	883,775	884,140	365	Loss
DEL00030881	chr4	1,044,679	1,044,736	57	Loss
DEL00030888	chr4	1,352,440	1,352,511	71	Loss
DEL00030919	chr4	2,132,620	2,132,685	65	Loss
DEL00030920	chr4	2,133,060	2,133,120	60	Loss
DEL00030923	chr4	2,191,907	2,192,355	448	Loss
DEL00030924	chr4	2,199,705	2,200,244	539	Loss
DEL00030925	chr4	2,226,678	2,226,763	85	Loss
DUP00030932	chr4	2,423,127	2,423,417	290	Gain
DEL00030936	chr4	2,548,785	2,548,837	52	Loss
DEL00030938	chr4	2,579,911	2,580,209	298	Loss
DEL00030941	chr4	2,607,781	2,607,945	164	Loss
DEL00030947	chr4	2,710,664	2,710,750	86	Loss
DEL00030956	chr4	2,851,327	2,851,384	57	Loss
DEL00030958	chr4	2,877,331	2,877,382	51	Loss
DEL00030963	chr4	2,946,644	2,946,736	92	Loss
DEL00030964	chr4	2,953,788	2,953,881	93	Loss
DEL00030969	chr4	3,015,976	3,016,209	233	Loss
DEL00030970	chr4	3,068,250	3,068,580	330	Loss
DEL00030974	chr4	3,171,242	3,171,786	544	Loss
DEL00030988	chr4	3,552,795	3,552,872	77	Loss
DEL00030990	chr4	3,573,056	3,573,195	139	Loss
DEL00030998	chr4	3,818,603	3,820,559	1,956	Loss
DEL00031003	chr4	3,927,439	3,927,529	90	Loss
DEL00031005	chr4	3,939,373	3,939,430	57	Loss
DEL00031009	chr4	4,101,801	4,102,030	229	Loss
DEL00031018	chr4	4,595,065	4,595,151	86	Loss
DEL00031026	chr4	5,035,359	5,035,415	56	Loss
DEL00031029	chr4	5,068,110	5,068,170	60	Loss
DEL00031033	chr4	5,191,528	5,191,608	80	Loss
DEL00031035	chr4	5,321,021	5,321,252	231	Loss
DUP00031037	chr4	5,380,894	5,381,016	122	Gain
DEL00031038	chr4	5,397,502	5,398,055	553	Loss

DEL00031039	chr4	5,398,839	5,398,953	114	Loss
DEL00031045	chr4	5,570,442	5,572,041	1,599	Loss
DEL00031048	chr4	5,682,550	5,682,725	175	Loss
DEL00031050	chr4	5,763,183	5,763,678	495	Loss
DEL00031052	chr4	5,801,179	5,801,552	373	Loss
DEL00031059	chr4	5,835,617	5,835,728	111	Loss
DEL00031065	chr4	5,973,462	5,973,686	224	Loss
DUP00031070	chr4	6,106,005	6,106,183	178	Gain
DEL00031074	chr4	6,200,758	6,201,314	556	Loss
DEL00031075	chr4	6,211,996	6,212,120	124	Loss
DUP00031076	chr4	6,246,713	6,255,364	8,651	Gain
DEL00031079	chr4	6,264,485	6,264,548	63	Loss
DEL00031080	chr4	6,269,037	6,269,134	97	Loss
DEL00031083	chr4	6,412,415	6,413,132	717	Loss
DEL00031084	chr4	6,434,124	6,434,659	535	Loss
DEL00031092	chr4	6,457,362	6,457,661	299	Loss
DEL00031093	chr4	6,484,862	6,487,944	3,082	Loss
DEL00031095	chr4	6,506,534	6,506,628	94	Loss
DEL00031102	chr4	6,916,788	6,916,887	99	Loss
DEL00031103	chr4	6,923,279	6,923,412	133	Loss
DEL00031107	chr4	6,970,887	6,970,946	59	Loss
DEL00031123	chr4	7,117,936	7,118,059	123	Loss
DEL00031125	chr4	7,146,327	7,147,149	822	Loss
DEL00031134	chr4	7,229,392	7,229,460	68	Loss
DEL00031137	chr4	7,287,326	7,287,470	144	Loss
DEL00031140	chr4	7,324,768	7,324,823	55	Loss
DEL00031142	chr4	7,404,433	7,404,999	566	Loss
DEL00031143	chr4	7,434,631	7,434,880	249	Loss
DEL00031150	chr4	7,575,128	7,576,036	908	Loss
DEL00031157	chr4	7,607,401	7,607,787	386	Loss
DEL00031160	chr4	7,628,508	7,629,082	574	Loss
DEL00031161	chr4	7,679,391	7,679,577	186	Loss
DEL00031164	chr4	7,712,111	7,712,421	310	Loss
DEL00031165	chr4	7,722,729	7,724,502	1,773	Loss
DEL00031168	chr4	7,747,512	7,747,568	56	Loss
DEL00031176	chr4	7,810,713	7,810,935	222	Loss
DEL00031177	chr4	7,818,747	7,818,881	134	Loss
DUP00031178	chr4	7,820,402	7,828,503	8,101	Gain
DEL00031179	chr4	7,829,639	7,829,931	292	Loss
DEL00031180	chr4	7,836,177	7,836,380	203	Loss
DEL00031184	chr4	7,879,643	7,879,788	145	Loss
DEL00031186	chr4	7,889,517	7,890,344	827	Loss
DEL00031191	chr4	7,943,382	7,943,991	609	Loss
DEL00031192	chr4	7,945,846	7,946,122	276	Loss
DEL00031194	chr4	7,950,315	7,950,984	669	Loss
DEL00031196	chr4	7,974,130	7,974,243	113	Loss
DUP00031200	chr4	7,998,277	7,998,443	166	Gain
DEL00031203	chr4	8,042,916	8,044,133	1,217	Loss
DEL00031206	chr4	8,124,576	8,125,432	856	Loss

DEL00031207	chr4	8,163,280	8,163,336	56	Loss
DEL00031211	chr4	8,216,244	8,216,306	62	Loss
DEL00031213	chr4	8,282,430	8,283,328	898	Loss
DEL00031214	chr4	8,288,777	8,288,846	69	Loss
DEL00031216	chr4	8,302,906	8,302,963	57	Loss
DEL00031219	chr4	8,351,139	8,351,399	260	Loss
DEL00031221	chr4	8,384,091	8,384,236	145	Loss
DEL00031225	chr4	8,442,676	8,443,129	453	Loss
DEL00031226	chr4	8,444,490	8,445,082	592	Loss
DEL00031232	chr4	8,581,748	8,581,808	60	Loss
DEL00031244	chr4	8,713,166	8,713,236	70	Loss
DEL00031246	chr4	8,717,378	8,717,706	328	Loss
DEL00031247	chr4	8,723,661	8,723,748	87	Loss
DEL00031254	chr4	8,903,130	8,903,223	93	Loss
DEL00031258	chr4	8,998,577	8,998,683	106	Loss
DEL00031260	chr4	9,078,203	9,078,580	377	Loss
DUP00031262	chr4	9,136,266	9,144,620	8,354	Gain
DEL00031264	chr4	9,287,979	9,288,035	56	Loss
DEL00031265	chr4	9,335,272	9,335,324	52	Loss
DEL00031278	chr4	9,623,152	9,623,580	428	Loss
DEL00031280	chr4	9,658,185	9,658,422	237	Loss
DEL00031283	chr4	9,735,852	9,736,347	495	Loss
DEL00031284	chr4	9,761,437	9,765,890	4,453	Loss
DEL00031293	chr4	9,967,550	9,968,167	617	Loss
DEL00031308	chr4	10,365,760	10,366,160	400	Loss
DEL00031315	chr4	10,415,259	10,415,335	76	Loss
DEL00031322	chr4	10,624,179	10,624,317	138	Loss
DEL00031323	chr4	10,631,239	10,631,299	60	Loss
DEL00031332	chr4	10,714,992	10,715,076	84	Loss
DEL00031337	chr4	10,825,393	10,825,447	54	Loss
DEL00031340	chr4	10,835,479	10,835,703	224	Loss
DEL00031344	chr4	10,896,053	10,896,621	568	Loss
DUP00031347	chr4	10,955,458	10,957,236	1,778	Gain
DEL00031353	chr4	11,027,861	11,027,966	105	Loss
DEL00031360	chr4	11,170,829	11,171,403	574	Loss
DEL00031362	chr4	11,237,386	11,237,917	531	Loss
DEL00031365	chr4	11,291,019	11,291,078	59	Loss
DEL00031366	chr4	11,291,439	11,291,581	142	Loss
DEL00031368	chr4	11,302,705	11,303,420	715	Loss
DEL00031372	chr4	11,402,873	11,402,960	87	Loss
DEL00031376	chr4	11,471,145	11,471,259	114	Loss
DEL00031378	chr4	11,483,778	11,484,047	269	Loss
DEL00031379	chr4	11,500,958	11,501,177	219	Loss
DEL00031381	chr4	11,563,193	11,563,982	789	Loss
DEL00031384	chr4	11,622,176	11,622,289	113	Loss
DEL00031387	chr4	11,728,142	11,728,225	83	Loss
DEL00031388	chr4	11,765,767	11,765,826	59	Loss
DEL00031389	chr4	11,799,559	11,799,717	158	Loss
DEL00031392	chr4	11,867,160	11,867,225	65	Loss

DEL00031393	chr4	11,960,600	11,960,656	56	Loss
DEL00031396	chr4	12,070,461	12,070,550	89	Loss
DEL00031400	chr4	12,077,979	12,078,230	251	Loss
DUP00031401	chr4	12,097,038	12,097,197	159	Gain
DEL00031406	chr4	12,466,472	12,466,944	472	Loss
DEL00031407	chr4	12,467,452	12,467,565	113	Loss
DEL00031414	chr4	12,548,094	12,548,950	856	Loss
DEL00031415	chr4	12,550,089	12,550,150	61	Loss
DEL00031421	chr4	12,614,203	12,614,276	73	Loss
DEL00031423	chr4	12,726,389	12,728,049	1,660	Loss
DEL00031425	chr4	12,750,986	12,751,117	131	Loss
DEL00031426	chr4	12,838,344	12,838,455	111	Loss
DEL00031433	chr4	12,987,968	12,988,179	211	Loss
DEL00031442	chr4	13,150,102	13,150,162	60	Loss
DEL00031443	chr4	13,159,326	13,159,737	411	Loss
DEL00031446	chr4	13,253,944	13,254,084	140	Loss
DEL00031450	chr4	13,303,955	13,304,191	236	Loss
DEL00031455	chr4	13,453,114	13,453,177	63	Loss
DEL00031466	chr4	13,667,076	13,667,146	70	Loss
DEL00031467	chr4	13,673,383	13,673,515	132	Loss
DEL00031468	chr4	13,676,831	13,676,968	137	Loss
DEL00031479	chr4	13,818,726	13,819,219	493	Loss
DEL00031490	chr4	13,917,709	13,917,804	95	Loss
DEL00031495	chr4	13,983,927	13,984,020	93	Loss
DEL00031496	chr4	14,043,162	14,044,079	917	Loss
DEL00031502	chr4	14,166,610	14,166,682	72	Loss
DEL00031503	chr4	14,236,837	14,236,898	61	Loss
DEL00031504	chr4	14,249,556	14,249,607	51	Loss
DEL00031505	chr4	14,272,780	14,272,983	203	Loss
DEL00031508	chr4	14,339,934	14,340,009	75	Loss
DEL00031509	chr4	14,366,050	14,366,149	99	Loss
DEL00031510	chr4	14,367,380	14,367,523	143	Loss
DEL00031530	chr4	14,735,582	14,736,265	683	Loss
DEL00031531	chr4	14,749,210	14,749,359	149	Loss
DEL00031548	chr4	14,984,417	14,985,018	601	Loss
DUP00031552	chr4	15,076,443	15,087,039	10,596	Gain
DEL00031555	chr4	15,117,515	15,117,571	56	Loss
DEL00031558	chr4	15,155,198	15,155,333	135	Loss
DEL00031562	chr4	15,196,395	15,196,615	220	Loss
DEL00031570	chr4	15,538,768	15,538,883	115	Loss
DEL00031575	chr4	15,786,542	15,789,906	3,364	Loss
DEL00031586	chr4	15,873,992	15,874,380	388	Loss
DEL00031590	chr4	15,936,267	15,936,932	665	Loss
DEL00031606	chr4	16,227,160	16,227,224	64	Loss
DEL00031610	chr4	16,253,585	16,253,946	361	Loss
DEL00031615	chr4	16,327,460	16,327,547	87	Loss
DEL00031617	chr4	16,357,404	16,357,542	138	Loss
DEL00031620	chr4	16,478,555	16,480,209	1,654	Loss
DEL00031621	chr4	16,480,576	16,480,635	59	Loss

DEL00031623	chr4	16,529,516	16,529,750	234	Loss
DEL00031637	chr4	16,826,997	16,827,586	589	Loss
DEL00031638	chr4	16,934,473	16,934,667	194	Loss
DEL00031646	chr4	17,040,232	17,040,400	168	Loss
DEL00031650	chr4	17,059,766	17,059,839	73	Loss
DEL00031654	chr4	17,075,124	17,075,270	146	Loss
DEL00031655	chr4	17,080,233	17,080,469	236	Loss
DEL00031659	chr4	17,103,563	17,105,017	1,454	Loss
DEL00031672	chr4	17,607,681	17,607,969	288	Loss
DEL00031677	chr4	17,665,457	17,666,284	827	Loss
DEL00031689	chr4	17,976,011	17,976,065	54	Loss
DEL00031691	chr4	17,988,714	17,991,308	2,594	Loss
DEL00031696	chr4	18,064,254	18,066,623	2,369	Loss
DEL00031697	chr4	18,108,120	18,108,226	106	Loss
DEL00031699	chr4	18,134,704	18,135,504	800	Loss
DEL00031731	chr4	18,683,323	18,686,632	3,309	Loss
DEL00031737	chr4	19,172,403	19,172,674	271	Loss
DEL00031747	chr4	19,275,018	19,277,823	2,805	Loss
DEL00031754	chr4	19,451,291	19,453,414	2,123	Loss
DEL00031791	chr4	20,162,482	20,162,689	207	Loss
DEL00031792	chr4	20,164,347	20,164,511	164	Loss
DEL00031797	chr4	20,253,672	20,253,735	63	Loss
DEL00031803	chr4	20,333,360	20,333,477	117	Loss
DEL00031808	chr4	20,465,681	20,466,032	351	Loss
DEL00031815	chr4	20,576,581	20,582,412	5,831	Loss
DEL00031820	chr4	20,632,100	20,632,156	56	Loss
DEL00031821	chr4	20,643,795	20,644,788	993	Loss
DEL00031822	chr4	20,656,580	20,656,796	216	Loss
DEL00031839	chr4	20,894,041	20,894,094	53	Loss
DEL00031840	chr4	20,910,446	20,910,519	73	Loss
DEL00031842	chr4	20,973,557	20,973,822	265	Loss
DEL00031853	chr4	21,160,311	21,161,435	1,124	Loss
DEL00031861	chr4	21,209,888	21,209,942	54	Loss
DEL00031873	chr4	21,295,819	21,295,897	78	Loss
DEL00031880	chr4	21,331,177	21,331,294	117	Loss
DEL00031885	chr4	21,373,210	21,374,190	980	Loss
DEL00031887	chr4	21,400,689	21,401,188	499	Loss
DUP00031895	chr4	21,483,138	21,483,355	217	Gain
DEL00031905	chr4	21,543,823	21,544,547	724	Loss
DEL00031911	chr4	21,556,997	21,557,565	568	Loss
DEL00031914	chr4	21,605,711	21,605,766	55	Loss
DEL00031919	chr4	21,672,334	21,672,556	222	Loss
DEL00031924	chr4	21,853,634	21,853,942	308	Loss
DEL00031928	chr4	21,873,689	21,873,961	272	Loss
DEL00031933	chr4	21,895,786	21,897,005	1,219	Loss
DEL00031940	chr4	21,953,226	21,953,603	377	Loss
DUP00031946	chr4	22,031,141	22,031,312	171	Gain
DEL00031947	chr4	22,046,959	22,047,486	527	Loss
DEL00031957	chr4	22,192,520	22,193,027	507	Loss

DEL00031958	chr4	22,229,240	22,229,443	203	Loss
DEL00031978	chr4	22,302,580	22,303,467	887	Loss
DEL00031980	chr4	22,375,245	22,375,310	65	Loss
DEL00031981	chr4	22,377,521	22,378,931	1,410	Loss
DEL00031984	chr4	22,423,873	22,423,935	62	Loss
DEL00031986	chr4	22,449,181	22,449,943	762	Loss
DEL00031987	chr4	22,459,331	22,459,395	64	Loss
DEL00031991	chr4	22,489,728	22,490,505	777	Loss
DEL00032000	chr4	22,586,841	22,588,429	1,588	Loss
DEL00032025	chr4	22,774,562	22,774,699	137	Loss
DEL00032026	chr4	22,792,530	22,792,614	84	Loss
DEL00032029	chr4	22,824,612	22,824,869	257	Loss
DEL00032033	chr4	22,888,830	22,888,970	140	Loss
DEL00032038	chr4	22,909,257	22,910,247	990	Loss
DEL00032041	chr4	22,919,709	22,920,069	360	Loss
DEL00032044	chr4	22,968,788	22,968,898	110	Loss
DEL00032046	chr4	22,996,439	22,996,894	455	Loss
DEL00032064	chr4	23,123,620	23,124,827	1,207	Loss
DEL00032065	chr4	23,142,376	23,142,533	157	Loss
DEL00032067	chr4	23,209,914	23,210,130	216	Loss
DEL00032068	chr4	23,232,041	23,232,103	62	Loss
DEL00032071	chr4	23,273,589	23,273,644	55	Loss
DEL00032076	chr4	23,331,916	23,332,627	711	Loss
DEL00032078	chr4	23,372,510	23,372,632	122	Loss
DEL00032085	chr4	23,463,607	23,464,259	652	Loss
DEL00032089	chr4	23,510,916	23,511,577	661	Loss
DEL00032098	chr4	23,644,055	23,644,352	297	Loss
DEL00032104	chr4	23,875,516	23,875,579	63	Loss
DUP00032167	chr4	24,142,988	24,143,341	353	Gain
DUP00032225	chr4	24,150,574	24,150,888	314	Gain
DEL00032248	chr4	24,192,210	24,192,902	692	Loss
DEL00032253	chr4	24,278,762	24,278,831	69	Loss
DEL00032255	chr4	24,294,622	24,294,731	109	Loss
DEL00032256	chr4	24,298,415	24,299,053	638	Loss
DEL00032259	chr4	24,325,300	24,325,440	140	Loss
DUP00032264	chr4	24,348,746	24,348,852	106	Gain
DUP00032266	chr4	24,359,047	24,359,152	105	Gain
DEL00032286	chr4	24,478,339	24,478,435	96	Loss
DEL00032291	chr4	24,509,710	24,510,380	670	Loss
DUP00032299	chr4	24,547,249	24,562,562	15,313	Gain
DEL00032304	chr4	24,572,435	24,572,508	73	Loss
DEL00032310	chr4	24,789,220	24,789,531	311	Loss
DEL00032311	chr4	24,795,130	24,795,256	126	Loss
DEL00032320	chr4	24,921,324	24,921,425	101	Loss
DEL00032321	chr4	24,953,156	24,954,077	921	Loss
DEL00032323	chr4	24,982,732	24,982,952	220	Loss
DEL00032325	chr4	24,989,598	24,989,662	64	Loss
DEL00032330	chr4	25,059,334	25,059,685	351	Loss
DEL00032331	chr4	25,060,397	25,060,452	55	Loss

DEL00032344	chr4	25,181,207	25,181,292	85	Loss
DEL00032345	chr4	25,198,921	25,199,458	537	Loss
DEL00032347	chr4	25,219,848	25,220,283	435	Loss
DEL00032348	chr4	25,239,285	25,239,347	62	Loss
DEL00032349	chr4	25,250,506	25,250,683	177	Loss
DEL00032352	chr4	25,303,429	25,303,627	198	Loss
DEL00032354	chr4	25,333,954	25,334,146	192	Loss
DEL00032360	chr4	25,363,116	25,363,663	547	Loss
DEL00032377	chr4	25,599,490	25,599,623	133	Loss
DEL00032379	chr4	25,626,683	25,626,796	113	Loss
DEL00032384	chr4	25,737,374	25,738,974	1,600	Loss
DEL00032389	chr4	25,849,623	25,849,932	309	Loss
DEL00032392	chr4	25,896,073	25,896,135	62	Loss
DEL00032406	chr4	25,996,712	25,997,440	728	Loss
DEL00032408	chr4	26,054,543	26,055,097	554	Loss
DEL00032412	chr4	26,167,166	26,168,524	1,358	Loss
DEL00032414	chr4	26,173,389	26,174,021	632	Loss
DEL00032420	chr4	26,210,519	26,211,699	1,180	Loss
DEL00032429	chr4	26,344,117	26,344,430	313	Loss
DEL00032438	chr4	26,484,127	26,484,585	458	Loss
DEL00032441	chr4	26,600,881	26,601,037	156	Loss
DEL00032444	chr4	26,620,358	26,620,908	550	Loss
DEL00032453	chr4	26,711,794	26,711,846	52	Loss
DEL00032457	chr4	26,792,440	26,792,562	122	Loss
DEL00032460	chr4	26,909,368	26,909,761	393	Loss
DEL00032465	chr4	26,971,761	26,971,953	192	Loss
DEL00032472	chr4	27,040,279	27,040,629	350	Loss
DEL00032483	chr4	27,234,144	27,235,063	919	Loss
DEL00032489	chr4	27,329,191	27,329,373	182	Loss
DEL00032492	chr4	27,339,431	27,339,813	382	Loss
DEL00032500	chr4	27,365,301	27,368,180	2,879	Loss
DEL00032505	chr4	27,483,930	27,483,984	54	Loss
DEL00032511	chr4	27,583,664	27,583,880	216	Loss
DEL00032518	chr4	27,653,731	27,653,977	246	Loss
DEL00032527	chr4	27,720,781	27,722,549	1,768	Loss
DEL00032542	chr4	27,910,074	27,910,371	297	Loss
DEL00032549	chr4	28,065,020	28,065,370	350	Loss
DEL00032550	chr4	28,138,315	28,138,509	194	Loss
DEL00032551	chr4	28,146,796	28,147,149	353	Loss
DEL00032566	chr4	28,380,122	28,380,182	60	Loss
DEL00032571	chr4	28,490,799	28,490,873	74	Loss
DEL00032579	chr4	28,546,743	28,547,241	498	Loss
DEL00032584	chr4	28,612,438	28,613,041	603	Loss
DEL00032586	chr4	28,642,117	28,642,168	51	Loss
DEL00032587	chr4	28,658,337	28,658,439	102	Loss
DEL00032610	chr4	29,000,702	29,001,196	494	Loss
DEL00032617	chr4	29,131,779	29,132,287	508	Loss
DEL00032622	chr4	29,158,459	29,158,766	307	Loss
DEL00032623	chr4	29,176,238	29,176,326	88	Loss

DEL00032624	chr4	29,191,028	29,191,115	87	Loss
DEL00032625	chr4	29,208,214	29,208,304	90	Loss
DEL00032632	chr4	29,245,441	29,245,541	100	Loss
DEL00032635	chr4	29,263,729	29,264,028	299	Loss
DEL00032646	chr4	29,495,307	29,495,422	115	Loss
DEL00032648	chr4	29,519,703	29,519,804	101	Loss
DEL00032651	chr4	29,559,792	29,560,706	914	Loss
DEL00032655	chr4	29,590,244	29,590,846	602	Loss
DEL00032663	chr4	29,653,408	29,654,598	1,190	Loss
DEL00032669	chr4	29,747,040	29,747,112	72	Loss
DEL00032672	chr4	29,816,359	29,817,564	1,205	Loss
DEL00032673	chr4	29,821,177	29,822,683	1,506	Loss
DEL00032681	chr4	30,004,453	30,005,033	580	Loss
DEL00032684	chr4	30,028,471	30,028,849	378	Loss
DEL00032687	chr4	30,106,313	30,106,841	528	Loss
DEL00032688	chr4	30,108,790	30,109,310	520	Loss
DEL00032690	chr4	30,111,008	30,111,183	175	Loss
DEL00032694	chr4	30,155,842	30,156,005	163	Loss
DEL00032696	chr4	30,186,849	30,186,928	79	Loss
DEL00032699	chr4	30,256,795	30,257,489	694	Loss
DEL00032702	chr4	30,296,873	30,296,966	93	Loss
DEL00032715	chr4	30,542,759	30,542,818	59	Loss
DEL00032717	chr4	30,557,748	30,558,277	529	Loss
DEL00032730	chr4	30,881,110	30,881,737	627	Loss
DEL00032732	chr4	30,914,490	30,914,616	126	Loss
DEL00032739	chr4	30,987,174	30,987,279	105	Loss
DEL00032749	chr4	31,098,897	31,099,031	134	Loss
DEL00032755	chr4	31,209,033	31,209,190	157	Loss
DEL00032756	chr4	31,213,417	31,213,657	240	Loss
DEL00032763	chr4	31,288,391	31,288,460	69	Loss
DEL00032765	chr4	31,330,977	31,331,200	223	Loss
DEL00032775	chr4	31,400,576	31,402,951	2,375	Loss
DEL00032776	chr4	31,403,128	31,403,634	506	Loss
DEL00032779	chr4	31,427,470	31,427,554	84	Loss
DEL00032782	chr4	31,473,976	31,474,630	654	Loss
DEL00032784	chr4	31,610,545	31,610,596	51	Loss
DEL00032787	chr4	31,672,441	31,672,501	60	Loss
DEL00032792	chr4	31,697,054	31,697,106	52	Loss
DEL00032794	chr4	31,759,659	31,759,891	232	Loss
DEL00032798	chr4	31,831,942	31,831,996	54	Loss
DEL00032800	chr4	31,900,303	31,900,403	100	Loss
DEL00032805	chr4	32,032,610	32,039,541	6,931	Loss
DEL00032813	chr4	32,193,753	32,194,341	588	Loss
DEL00032814	chr4	32,237,497	32,237,724	227	Loss
DEL00032820	chr4	32,467,740	32,467,791	51	Loss
DEL00032823	chr4	32,591,079	32,591,251	172	Loss
DEL00032830	chr4	32,693,317	32,693,465	148	Loss
DEL00032835	chr4	32,811,559	32,811,622	63	Loss
DEL00032837	chr4	32,826,012	32,826,433	421	Loss

DEL00032842	chr4	32,967,313	32,968,366	1,053	Loss
DEL00032846	chr4	32,997,745	32,998,070	325	Loss
DEL00032850	chr4	33,059,942	33,060,138	196	Loss
DEL00032851	chr4	33,116,588	33,116,653	65	Loss
DEL00032852	chr4	33,133,284	33,134,093	809	Loss
DEL00032855	chr4	33,147,625	33,147,694	69	Loss
DEL00032858	chr4	33,232,288	33,232,728	440	Loss
DEL00032865	chr4	33,268,435	33,268,519	84	Loss
DEL00032867	chr4	33,357,595	33,358,395	800	Loss
DUP00032876	chr4	33,487,347	33,487,590	243	Gain
DUP00032881	chr4	33,515,729	33,530,457	14,728	Gain
DEL00032885	chr4	33,563,642	33,564,517	875	Loss
DEL00032886	chr4	33,610,795	33,610,913	118	Loss
DEL00032887	chr4	33,629,209	33,629,477	268	Loss
DEL00032896	chr4	33,878,439	33,879,750	1,311	Loss
DEL00032897	chr4	33,893,989	33,894,218	229	Loss
DEL00032903	chr4	33,989,213	33,989,265	52	Loss
DEL00032904	chr4	33,991,486	33,991,557	71	Loss
DUP00032907	chr4	34,044,578	34,044,675	97	Gain
DEL00032908	chr4	34,110,456	34,110,552	96	Loss
DEL00032910	chr4	34,162,044	34,162,241	197	Loss
DEL00032917	chr4	34,274,953	34,275,009	56	Loss
DEL00032919	chr4	34,344,406	34,344,951	545	Loss
DEL00032921	chr4	34,442,748	34,442,823	75	Loss
DEL00032924	chr4	34,538,596	34,538,780	184	Loss
DEL00032929	chr4	34,582,635	34,582,752	117	Loss
DEL00032931	chr4	34,589,899	34,590,298	399	Loss
DEL00032933	chr4	34,637,452	34,637,947	495	Loss
DEL00032944	chr4	34,752,970	34,753,028	58	Loss
DEL00032949	chr4	34,798,625	34,798,705	80	Loss
DEL00032953	chr4	34,895,207	34,895,921	714	Loss
DEL00032954	chr4	34,903,585	34,903,640	55	Loss
DEL00032970	chr4	35,279,701	35,279,799	98	Loss
DEL00032971	chr4	35,285,366	35,285,637	271	Loss
DEL00032972	chr4	35,311,921	35,312,663	742	Loss
DEL00032977	chr4	35,362,089	35,362,390	301	Loss
DEL00032978	chr4	35,375,129	35,375,330	201	Loss
DEL00032983	chr4	35,468,481	35,468,823	342	Loss
DEL00032984	chr4	35,478,870	35,478,959	89	Loss
DEL00032990	chr4	35,598,312	35,598,573	261	Loss
DEL00032992	chr4	35,657,739	35,658,179	440	Loss
DUP00032993	chr4	35,695,779	35,711,663	15,884	Gain
DEL00032999	chr4	35,798,029	35,801,467	3,438	Loss
DEL00033004	chr4	35,804,795	35,804,851	56	Loss
DEL00033006	chr4	35,819,728	35,819,810	82	Loss
DEL00033010	chr4	35,825,743	35,826,378	635	Loss
DEL00033011	chr4	35,828,132	35,828,437	305	Loss
DEL00033015	chr4	35,860,555	35,860,885	330	Loss
DEL00033017	chr4	35,896,683	35,896,778	95	Loss

DEL00033022	chr4	35,999,707	35,999,878	171	Loss
DEL00033029	chr4	36,023,091	36,023,663	572	Loss
DEL00033036	chr4	36,062,721	36,062,927	206	Loss
DUP00033037	chr4	36,069,652	36,069,759	107	Gain
DEL00033038	chr4	36,079,594	36,079,821	227	Loss
DEL00033039	chr4	36,080,542	36,080,811	269	Loss
DEL00033049	chr4	36,287,777	36,288,268	491	Loss
DEL00033050	chr4	36,309,446	36,309,697	251	Loss
DEL00033072	chr4	36,769,225	36,769,278	53	Loss
DEL00033088	chr4	37,110,423	37,110,582	159	Loss
DEL00033089	chr4	37,118,567	37,118,675	108	Loss
DEL00033093	chr4	37,157,373	37,157,440	67	Loss
DEL00033096	chr4	37,185,896	37,185,986	90	Loss
DEL00033098	chr4	37,261,125	37,262,458	1,333	Loss
DEL00033108	chr4	37,398,122	37,398,174	52	Loss
DEL00033111	chr4	37,407,199	37,407,266	67	Loss
DEL00033114	chr4	37,443,523	37,443,581	58	Loss
DEL00033116	chr4	37,447,348	37,447,460	112	Loss
DEL00033117	chr4	37,453,691	37,453,758	67	Loss
DEL00033119	chr4	37,565,368	37,565,689	321	Loss
DEL00033121	chr4	37,611,623	37,611,802	179	Loss
DEL00033127	chr4	37,795,549	37,795,751	202	Loss
DEL00033137	chr4	37,945,357	37,945,596	239	Loss
DEL00033142	chr4	37,977,563	37,977,628	65	Loss
DEL00033145	chr4	38,024,264	38,028,386	4,122	Loss
DEL00033149	chr4	38,214,813	38,214,948	135	Loss
DEL00033154	chr4	38,218,780	38,218,834	54	Loss
DEL00033165	chr4	38,356,087	38,356,223	136	Loss
DEL00033166	chr4	38,361,393	38,361,625	232	Loss
DEL00033168	chr4	38,386,203	38,386,722	519	Loss
DEL00033175	chr4	38,479,368	38,479,429	61	Loss
DEL00033176	chr4	38,550,638	38,550,722	84	Loss
DEL00033179	chr4	38,603,475	38,603,690	215	Loss
DUP00033196	chr4	38,878,775	38,881,237	2,462	Gain
DEL00033201	chr4	38,930,123	38,930,211	88	Loss
DEL00033206	chr4	39,003,847	39,004,309	462	Loss
DEL00033207	chr4	39,011,912	39,011,999	87	Loss
DEL00033209	chr4	39,082,148	39,082,486	338	Loss
DEL00033216	chr4	39,188,208	39,188,459	251	Loss
DEL00033217	chr4	39,213,027	39,213,082	55	Loss
DEL00033218	chr4	39,213,233	39,213,983	750	Loss
DUP00033219	chr4	39,230,999	39,231,224	225	Gain
DEL00033220	chr4	39,248,965	39,249,184	219	Loss
DEL00033223	chr4	39,317,265	39,317,355	90	Loss
DEL00033226	chr4	39,487,754	39,487,875	121	Loss
DEL00033232	chr4	39,610,302	39,610,623	321	Loss
DEL00033241	chr4	39,720,429	39,720,502	73	Loss
DEL00033250	chr4	40,064,560	40,065,734	1,174	Loss
DEL00033251	chr4	40,072,146	40,072,295	149	Loss

DEL00033259	chr4	40,187,406	40,187,909	503	Loss
DEL00033270	chr4	40,409,930	40,410,515	585	Loss
DEL00033274	chr4	40,450,422	40,451,273	851	Loss
DEL00033278	chr4	40,529,864	40,530,003	139	Loss
DEL00033284	chr4	40,721,586	40,722,817	1,231	Loss
DEL00033285	chr4	40,726,068	40,726,120	52	Loss
DEL00033288	chr4	40,769,063	40,769,135	72	Loss
DEL00033301	chr4	41,004,336	41,004,616	280	Loss
DEL00033302	chr4	41,009,360	41,011,603	2,243	Loss
DEL00033311	chr4	41,170,653	41,172,420	1,767	Loss
DEL00033323	chr4	41,322,618	41,322,784	166	Loss
DEL00033336	chr4	41,534,199	41,534,411	212	Loss
DEL00033340	chr4	41,584,817	41,584,987	170	Loss
DEL00033342	chr4	41,717,545	41,717,682	137	Loss
DEL00033345	chr4	41,807,520	41,807,634	114	Loss
DEL00033348	chr4	41,823,139	41,823,651	512	Loss
DEL00033355	chr4	41,861,605	41,862,584	979	Loss
DEL00033356	chr4	41,911,037	41,911,090	53	Loss
DEL00033357	chr4	41,915,393	41,918,433	3,040	Loss
DEL00033361	chr4	41,961,798	41,961,925	127	Loss
DEL00033372	chr4	42,081,771	42,081,828	57	Loss
DEL00033373	chr4	42,098,994	42,099,216	222	Loss
DEL00033377	chr4	42,118,492	42,118,569	77	Loss
DEL00033379	chr4	42,150,256	42,150,636	380	Loss
DUP00033380	chr4	42,218,670	42,218,907	237	Gain
DEL00033382	chr4	42,239,805	42,239,879	74	Loss
DEL00033383	chr4	42,252,705	42,252,812	107	Loss
DUP00033385	chr4	42,282,571	42,283,231	660	Gain
DEL00033391	chr4	42,328,175	42,328,606	431	Loss
DEL00033395	chr4	42,381,400	42,381,749	349	Loss
DEL00033400	chr4	42,448,787	42,449,393	606	Loss
DEL00033403	chr4	42,492,555	42,497,568	5,013	Loss
DEL00033405	chr4	42,513,968	42,514,067	99	Loss
DEL00033410	chr4	42,593,376	42,593,568	192	Loss
DUP00033422	chr4	42,772,458	42,787,921	15,463	Gain
DEL00033423	chr4	42,799,116	42,799,720	604	Loss
DEL00033429	chr4	42,932,207	42,932,531	324	Loss
DEL00033433	chr4	42,986,536	42,986,626	90	Loss
DEL00033447	chr4	43,302,156	43,303,970	1,814	Loss
DEL00033453	chr4	43,378,348	43,378,651	303	Loss
DEL00033454	chr4	43,388,465	43,388,754	289	Loss
DEL00033456	chr4	43,391,294	43,391,350	56	Loss
DEL00033458	chr4	43,431,000	43,432,044	1,044	Loss
DEL00033460	chr4	43,498,439	43,498,526	87	Loss
DEL00033468	chr4	43,688,492	43,689,342	850	Loss
DEL00033469	chr4	43,694,698	43,694,796	98	Loss
DEL00033470	chr4	43,697,031	43,698,002	971	Loss
DEL00033480	chr4	43,875,734	43,876,556	822	Loss
DEL00033481	chr4	43,879,481	43,879,570	89	Loss

DEL00033485	chr4	43,948,751	43,954,207	5,456	Loss
DEL00033490	chr4	44,033,623	44,033,687	64	Loss
DEL00033492	chr4	44,087,191	44,088,442	1,251	Loss
DEL00033499	chr4	44,208,338	44,208,428	90	Loss
DEL00033502	chr4	44,259,463	44,259,739	276	Loss
DEL00033509	chr4	44,311,577	44,311,630	53	Loss
DEL00033511	chr4	44,333,880	44,334,499	619	Loss
DEL00033515	chr4	44,386,415	44,386,576	161	Loss
DEL00033519	chr4	44,399,035	44,399,262	227	Loss
DEL00033524	chr4	44,440,378	44,440,471	93	Loss
DEL00033525	chr4	44,456,691	44,457,182	491	Loss
DEL00033526	chr4	44,468,825	44,468,882	57	Loss
DEL00033536	chr4	44,543,404	44,543,572	168	Loss
DEL00033542	chr4	44,674,741	44,675,429	688	Loss
DEL00033545	chr4	44,790,339	44,790,396	57	Loss
DEL00033551	chr4	44,843,526	44,843,818	292	Loss
DEL00033553	chr4	44,966,263	44,966,329	66	Loss
DEL00033556	chr4	45,053,517	45,053,902	385	Loss
DEL00033559	chr4	45,207,123	45,207,194	71	Loss
DEL00033560	chr4	45,215,934	45,216,115	181	Loss
DEL00033563	chr4	45,394,010	45,394,099	89	Loss
DEL00033564	chr4	45,524,099	45,524,213	114	Loss
DEL00033566	chr4	45,550,101	45,550,851	750	Loss
DEL00033571	chr4	45,588,718	45,588,782	64	Loss
DEL00033575	chr4	45,764,513	45,764,571	58	Loss
DEL00033581	chr4	45,917,253	45,917,784	531	Loss
DEL00033585	chr4	45,966,826	45,966,951	125	Loss
DEL00033586	chr4	45,985,393	45,985,780	387	Loss
DEL00033594	chr4	46,108,995	46,109,059	64	Loss
DEL00033595	chr4	46,118,134	46,118,392	258	Loss
DEL00033596	chr4	46,128,634	46,128,833	199	Loss
DEL00033597	chr4	46,132,611	46,132,904	293	Loss
DEL00033598	chr4	46,136,840	46,136,901	61	Loss
DEL00033601	chr4	46,204,908	46,204,981	73	Loss
DUP00033614	chr4	46,396,579	46,406,934	10,355	Gain
DEL00033619	chr4	46,476,248	46,476,735	487	Loss
DEL00033629	chr4	46,562,879	46,563,185	306	Loss
DEL00033632	chr4	46,579,763	46,580,342	579	Loss
DEL00033640	chr4	46,680,658	46,680,718	60	Loss
DEL00033643	chr4	46,739,524	46,740,654	1,130	Loss
DEL00033647	chr4	46,918,993	46,919,045	52	Loss
DEL00033672	chr4	47,200,058	47,200,151	93	Loss
DEL00033673	chr4	47,290,213	47,290,410	197	Loss
DEL00033674	chr4	47,298,964	47,299,043	79	Loss
DEL00033679	chr4	47,345,293	47,345,586	293	Loss
DEL00033680	chr4	47,346,499	47,346,565	66	Loss
DEL00033682	chr4	47,374,367	47,374,562	195	Loss
DEL00033683	chr4	47,441,907	47,442,440	533	Loss
DEL00033694	chr4	47,723,834	47,724,064	230	Loss

DEL00033696	chr4	47,768,350	47,768,763	413	Loss
DEL00033699	chr4	47,856,565	47,857,300	735	Loss
DEL00033702	chr4	47,883,729	47,883,789	60	Loss
DEL00033706	chr4	47,960,514	47,960,773	259	Loss
DEL00033712	chr4	48,315,903	48,316,046	143	Loss
DEL00033718	chr4	48,366,606	48,374,115	7,509	Loss
DEL00033724	chr4	48,425,214	48,425,289	75	Loss
DEL00033728	chr4	48,573,885	48,574,416	531	Loss
DEL00033732	chr4	48,617,274	48,617,857	583	Loss
DEL00033735	chr4	48,677,718	48,679,223	1,505	Loss
DEL00033736	chr4	48,704,559	48,704,781	222	Loss
DEL00033745	chr4	48,827,677	48,827,810	133	Loss
DEL00033753	chr4	48,946,817	48,947,016	199	Loss
DEL00033756	chr4	49,014,061	49,017,144	3,083	Loss
DEL00033762	chr4	49,089,290	49,089,430	140	Loss
DEL00033765	chr4	49,145,632	49,145,784	152	Loss
DEL00033771	chr4	49,237,781	49,243,275	5,494	Loss
DEL00033785	chr4	49,322,376	49,322,497	121	Loss
DEL00033793	chr4	49,536,602	49,537,288	686	Loss
DEL00033800	chr4	49,583,217	49,583,305	88	Loss
DEL00033807	chr4	49,733,795	49,733,960	165	Loss
DEL00033810	chr4	49,820,196	49,820,482	286	Loss
DEL00033815	chr4	49,902,117	49,902,191	74	Loss
DEL00033817	chr4	49,952,588	49,952,671	83	Loss
DEL00033830	chr4	50,102,017	50,102,088	71	Loss
DEL00033834	chr4	50,149,589	50,149,987	398	Loss
DEL00033835	chr4	50,155,310	50,155,748	438	Loss
DEL00033845	chr4	50,306,836	50,306,917	81	Loss
DEL00033847	chr4	50,345,696	50,345,841	145	Loss
DEL00033858	chr4	50,452,663	50,452,728	65	Loss
DEL00033859	chr4	50,471,019	50,471,168	149	Loss
DEL00033860	chr4	50,494,346	50,498,968	4,622	Loss
DEL00033868	chr4	50,579,505	50,579,779	274	Loss
DEL00033870	chr4	50,597,254	50,597,351	97	Loss
DEL00033872	chr4	50,648,543	50,649,065	522	Loss
DEL00033879	chr4	50,749,613	50,749,702	89	Loss
DEL00033884	chr4	50,787,587	50,787,998	411	Loss
DEL00033886	chr4	50,814,516	50,814,573	57	Loss
DEL00033887	chr4	50,823,934	50,824,001	67	Loss
DEL00033892	chr4	50,860,482	50,861,442	960	Loss
DEL00033898	chr4	51,003,229	51,003,315	86	Loss
DEL00033899	chr4	51,023,622	51,024,077	455	Loss
DEL00033904	chr4	51,133,203	51,135,344	2,141	Loss
DEL00033905	chr4	51,140,361	51,140,775	414	Loss
DEL00033910	chr4	51,183,648	51,183,976	328	Loss
DEL00033911	chr4	51,229,606	51,229,667	61	Loss
DEL00033912	chr4	51,300,127	51,300,259	132	Loss
DEL00033919	chr4	51,494,581	51,494,635	54	Loss
DEL00033920	chr4	51,497,816	51,497,931	115	Loss

DEL00033925	chr4	51,588,668	51,589,128	460	Loss
DEL00033927	chr4	51,599,438	51,599,603	165	Loss
DEL00033929	chr4	51,638,251	51,638,734	483	Loss
DEL00033933	chr4	51,722,862	51,722,933	71	Loss
DEL00033935	chr4	51,734,410	51,734,731	321	Loss
DEL00033938	chr4	51,789,118	51,789,201	83	Loss
DEL00033942	chr4	51,849,052	51,849,156	104	Loss
DEL00033944	chr4	51,853,119	51,853,751	632	Loss
DEL00033946	chr4	51,923,736	51,923,853	117	Loss
DUP00033954	chr4	51,961,615	51,961,719	104	Gain
DEL00033956	chr4	51,969,731	51,969,791	60	Loss
DEL00033962	chr4	52,055,896	52,055,961	65	Loss
DEL00033972	chr4	52,146,746	52,146,824	78	Loss
DEL00033973	chr4	52,164,571	52,164,918	347	Loss
DEL00033974	chr4	52,170,169	52,171,053	884	Loss
DEL00034002	chr4	52,717,309	52,717,444	135	Loss
DEL00034006	chr4	52,861,905	52,862,956	1,051	Loss
DEL00034012	chr4	52,918,169	52,918,839	670	Loss
DEL00034013	chr4	52,959,230	52,959,290	60	Loss
DEL00034021	chr4	53,235,345	53,235,397	52	Loss
DEL00034023	chr4	53,285,119	53,285,176	57	Loss
DEL00034027	chr4	53,385,421	53,385,951	530	Loss
DEL00034031	chr4	53,451,779	53,451,903	124	Loss
DEL00034039	chr4	53,582,440	53,582,990	550	Loss
DEL00034040	chr4	53,604,508	53,604,686	178	Loss
DEL00034049	chr4	53,813,893	53,814,004	111	Loss
DEL00034053	chr4	53,845,388	53,845,455	67	Loss
DEL00034056	chr4	53,904,730	53,905,231	501	Loss
DEL00034068	chr4	54,167,041	54,167,590	549	Loss
DEL00034069	chr4	54,169,995	54,170,046	51	Loss
DEL00034071	chr4	54,225,475	54,226,172	697	Loss
DEL00034078	chr4	54,312,564	54,312,786	222	Loss
DEL00034079	chr4	54,323,309	54,325,443	2,134	Loss
DEL00034084	chr4	54,382,742	54,382,851	109	Loss
DEL00034088	chr4	54,391,803	54,391,923	120	Loss
DEL00034089	chr4	54,416,860	54,417,260	400	Loss
DEL00034090	chr4	54,435,476	54,435,636	160	Loss
DEL00034094	chr4	54,507,981	54,508,672	691	Loss
DEL00034101	chr4	54,575,501	54,575,606	105	Loss
DEL00034107	chr4	54,757,970	54,758,022	52	Loss
DEL00034109	chr4	54,758,418	54,758,648	230	Loss
DEL00034110	chr4	54,831,025	54,831,126	101	Loss
DEL00034111	chr4	54,848,760	54,848,872	112	Loss
DEL00034113	chr4	54,864,360	54,864,421	61	Loss
DEL00034114	chr4	54,923,398	54,923,501	103	Loss
DUP00034118	chr4	55,055,228	55,068,701	13,473	Gain
DEL00034125	chr4	55,127,108	55,128,562	1,454	Loss
DEL00034131	chr4	55,177,708	55,177,785	77	Loss
DEL00034133	chr4	55,187,150	55,188,160	1,010	Loss

DEL00034138	chr4	55,235,272	55,241,105	5,833	Loss
DEL00034143	chr4	55,363,640	55,363,935	295	Loss
DEL00034144	chr4	55,434,952	55,435,412	460	Loss
DEL00034165	chr4	55,887,456	55,887,863	407	Loss
DEL00034178	chr4	56,075,376	56,076,105	729	Loss
DEL00034179	chr4	56,088,376	56,088,483	107	Loss
DEL00034180	chr4	56,111,171	56,111,990	819	Loss
DEL00034183	chr4	56,126,916	56,127,369	453	Loss
DEL00034185	chr4	56,157,549	56,157,638	89	Loss
DEL00034189	chr4	56,188,910	56,189,005	95	Loss
DEL00034194	chr4	56,234,599	56,235,139	540	Loss
DEL00034196	chr4	56,237,631	56,237,741	110	Loss
DEL00034197	chr4	56,260,120	56,260,730	610	Loss
DEL00034205	chr4	56,537,003	56,539,872	2,869	Loss
DEL00034207	chr4	56,564,402	56,564,455	53	Loss
DEL00034218	chr4	56,820,118	56,820,347	229	Loss
DEL00034225	chr4	56,929,915	56,930,160	245	Loss
DEL00034232	chr4	57,052,906	57,053,063	157	Loss
DEL00034238	chr4	57,193,963	57,194,039	76	Loss
DEL00034240	chr4	57,196,998	57,197,565	567	Loss
DEL00034242	chr4	57,256,182	57,256,316	134	Loss
DEL00034247	chr4	57,360,935	57,361,000	65	Loss
DEL00034248	chr4	57,387,015	57,387,772	757	Loss
DEL00034249	chr4	57,390,456	57,390,535	79	Loss
DEL00034252	chr4	57,450,635	57,450,701	66	Loss
DEL00034259	chr4	57,659,864	57,660,047	183	Loss
DEL00034267	chr4	57,757,331	57,757,896	565	Loss
DEL00034271	chr4	57,827,007	57,828,231	1,224	Loss
DEL00034273	chr4	57,863,070	57,863,623	553	Loss
DEL00034278	chr4	57,925,969	57,926,525	556	Loss
DEL00034282	chr4	57,971,158	57,971,445	287	Loss
DEL00034285	chr4	58,032,240	58,032,731	491	Loss
DEL00034287	chr4	58,080,051	58,080,189	138	Loss
DUP00034294	chr4	58,199,124	58,199,214	90	Gain
DEL00034300	chr4	58,352,618	58,352,673	55	Loss
DEL00034316	chr4	58,626,261	58,626,642	381	Loss
DEL00034339	chr4	59,017,736	59,018,116	380	Loss
DEL00034340	chr4	59,032,069	59,035,991	3,922	Loss
DEL00034341	chr4	59,080,055	59,080,175	120	Loss
DEL00034342	chr4	59,114,332	59,115,004	672	Loss
DEL00034343	chr4	59,115,051	59,115,518	467	Loss
DEL00034344	chr4	59,130,218	59,130,806	588	Loss
DEL00034348	chr4	59,186,651	59,187,836	1,185	Loss
DEL00034352	chr4	59,316,423	59,316,502	79	Loss
DEL00034356	chr4	59,369,648	59,369,721	73	Loss
DEL00034357	chr4	59,399,766	59,399,870	104	Loss
DEL00034362	chr4	59,438,952	59,439,033	81	Loss
DEL00034369	chr4	59,491,422	59,491,545	123	Loss
DEL00034370	chr4	59,503,592	59,503,671	79	Loss

DEL00034371	chr4	59,506,365	59,509,142	2,777	Loss
DEL00034372	chr4	59,515,479	59,515,590	111	Loss
DEL00034379	chr4	59,579,489	59,579,709	220	Loss
DEL00034383	chr4	59,663,770	59,663,822	52	Loss
DEL00034384	chr4	59,667,192	59,667,409	217	Loss
DEL00034387	chr4	59,766,737	59,766,846	109	Loss
DEL00034400	chr4	59,868,039	59,868,269	230	Loss
DEL00034407	chr4	59,937,513	59,938,855	1,342	Loss
DEL00034408	chr4	59,954,476	59,954,550	74	Loss
DEL00034409	chr4	59,954,887	59,955,383	496	Loss
DEL00034412	chr4	59,978,243	59,978,323	80	Loss
DEL00034413	chr4	59,980,109	59,980,365	256	Loss
DEL00034415	chr4	60,034,628	60,034,704	76	Loss
DEL00034419	chr4	60,089,893	60,089,960	67	Loss
DEL00034420	chr4	60,129,749	60,129,868	119	Loss
DEL00034424	chr4	60,245,527	60,245,587	60	Loss
DEL00034428	chr4	60,286,080	60,286,757	677	Loss
DEL00034436	chr4	60,431,278	60,431,542	264	Loss
DEL00034437	chr4	60,483,858	60,484,012	154	Loss
DEL00034439	chr4	60,486,702	60,486,890	188	Loss
DEL00034441	chr4	60,589,340	60,589,417	77	Loss
DEL00034460	chr4	60,862,408	60,862,972	564	Loss
DEL00034471	chr4	61,020,567	61,020,670	103	Loss
DEL00034474	chr4	61,080,531	61,081,948	1,417	Loss
DEL00034480	chr4	61,147,084	61,148,216	1,132	Loss
DEL00034484	chr4	61,230,367	61,230,426	59	Loss
DEL00034487	chr4	61,258,733	61,258,981	248	Loss
DEL00034489	chr4	61,275,085	61,275,171	86	Loss
DEL00034492	chr4	61,394,926	61,395,846	920	Loss
DEL00034495	chr4	61,413,463	61,413,609	146	Loss
DEL00034496	chr4	61,414,351	61,414,583	232	Loss
DEL00034502	chr4	61,476,602	61,477,529	927	Loss
DEL00034503	chr4	61,479,590	61,479,662	72	Loss
DEL00034514	chr4	61,728,930	61,728,982	52	Loss
DEL00034516	chr4	61,750,190	61,750,656	466	Loss
DEL00034517	chr4	61,775,775	61,775,830	55	Loss
DEL00034521	chr4	61,798,704	61,798,921	217	Loss
DEL00034522	chr4	61,806,305	61,806,906	601	Loss
DEL00034524	chr4	61,885,147	61,885,210	63	Loss
DEL00034534	chr4	62,074,197	62,074,278	81	Loss
DEL00034539	chr4	62,116,654	62,116,762	108	Loss
DEL00034541	chr4	62,166,402	62,166,569	167	Loss
DEL00034545	chr4	62,248,848	62,248,908	60	Loss
DEL00034552	chr4	62,338,984	62,339,037	53	Loss
DEL00034554	chr4	62,353,059	62,353,470	411	Loss
DEL00034562	chr4	62,405,197	62,405,492	295	Loss
DEL00034571	chr4	62,529,528	62,529,713	185	Loss
DEL00034573	chr4	62,555,218	62,555,366	148	Loss
DEL00034580	chr4	62,681,688	62,681,794	106	Loss

DEL00034581	chr4	62,690,032	62,690,086	54	Loss
DEL00034586	chr4	62,897,111	62,897,214	103	Loss
DEL00034589	chr4	62,920,823	62,921,048	225	Loss
DEL00034595	chr4	62,934,482	62,934,757	275	Loss
DEL00034596	chr4	62,946,981	62,954,714	7,733	Mixed
DEL00034605	chr4	63,044,338	63,044,464	126	Loss
DEL00034609	chr4	63,143,553	63,143,635	82	Loss
DEL00034610	chr4	63,159,067	63,159,157	90	Loss
DUP00034621	chr4	63,361,998	63,362,110	112	Gain
DEL00034625	chr4	63,452,221	63,452,433	212	Loss
DEL00034626	chr4	63,452,609	63,452,660	51	Loss
DEL00034627	chr4	63,486,985	63,487,514	529	Loss
DEL00034632	chr4	63,580,876	63,583,194	2,318	Loss
DEL00034635	chr4	63,603,862	63,607,448	3,586	Loss
DEL00034639	chr4	63,634,908	63,634,993	85	Loss
DEL00034644	chr4	63,698,060	63,702,288	4,228	Loss
DUP00034648	chr4	63,720,047	63,720,177	130	Gain
DEL00034650	chr4	63,771,770	63,771,835	65	Loss
DEL00034651	chr4	63,776,880	63,783,424	6,544	Loss
DEL00034655	chr4	63,820,141	63,820,223	82	Loss
DEL00034656	chr4	63,829,073	63,829,137	64	Loss
DEL00034658	chr4	63,886,938	63,889,243	2,305	Loss
DEL00034659	chr4	63,955,831	63,956,026	195	Loss
DEL00034663	chr4	63,970,527	63,970,871	344	Loss
DEL00034664	chr4	64,005,862	64,007,445	1,583	Loss
DEL00034665	chr4	64,080,733	64,080,914	181	Loss
DEL00034666	chr4	64,122,984	64,123,806	822	Loss
DEL00034667	chr4	64,147,675	64,147,743	68	Loss
DEL00034669	chr4	64,185,572	64,185,635	63	Loss
DEL00034673	chr4	64,274,858	64,275,296	438	Loss
DEL00034674	chr4	64,323,593	64,324,243	650	Loss
DEL00034683	chr4	64,543,526	64,544,060	534	Loss
DEL00034687	chr4	64,603,737	64,603,946	209	Loss
DEL00034688	chr4	64,611,742	64,612,182	440	Loss
DEL00034691	chr4	64,655,865	64,656,020	155	Loss
DEL00034707	chr4	64,832,337	64,833,312	975	Loss
DEL00034712	chr4	64,856,134	64,856,197	63	Loss
DEL00034717	chr4	64,897,032	64,897,339	307	Loss
DEL00034725	chr4	64,963,700	64,964,265	565	Loss
DEL00034728	chr4	64,999,976	65,000,141	165	Loss
DEL00034729	chr4	65,027,397	65,027,773	376	Loss
DEL00034731	chr4	65,054,607	65,054,711	104	Loss
DEL00034732	chr4	65,110,292	65,110,462	170	Loss
DEL00034745	chr4	65,468,178	65,468,290	112	Loss
DEL00034747	chr4	65,552,547	65,553,067	520	Loss
DEL00034750	chr4	65,636,823	65,637,899	1,076	Loss
DEL00034754	chr4	65,666,560	65,666,663	103	Loss
DEL00034760	chr4	65,747,661	65,747,866	205	Loss
DEL00034767	chr4	66,004,162	66,004,231	69	Loss

DEL00034778	chr4	66,210,538	66,210,604	66	Loss
DEL00034789	chr4	66,417,925	66,418,107	182	Loss
DEL00034800	chr4	66,487,786	66,487,843	57	Loss
DEL00034806	chr4	66,552,586	66,552,677	91	Loss
DEL00034809	chr4	66,621,816	66,621,909	93	Loss
DEL00034811	chr4	66,647,496	66,648,295	799	Loss
DEL00034812	chr4	66,675,667	66,675,956	289	Loss
DEL00034815	chr4	66,739,935	66,740,006	71	Loss
DEL00034817	chr4	66,752,542	66,752,670	128	Loss
DEL00034823	chr4	66,877,102	66,877,168	66	Loss
DEL00034824	chr4	66,991,858	66,993,102	1,244	Loss
DEL00034828	chr4	67,079,332	67,080,677	1,345	Loss
DEL00034835	chr4	67,186,483	67,187,693	1,210	Loss
DEL00034837	chr4	67,231,096	67,232,273	1,177	Loss
DUP00034847	chr4	67,392,386	67,392,516	130	Gain
DEL00034853	chr4	67,476,184	67,476,283	99	Loss
DEL00034863	chr4	67,584,214	67,587,651	3,437	Loss
DEL00034864	chr4	67,612,396	67,612,987	591	Loss
DEL00034865	chr4	67,618,444	67,618,577	133	Loss
DEL00034867	chr4	67,651,382	67,651,486	104	Loss
DEL00034868	chr4	67,652,239	67,652,297	58	Loss
DEL00034873	chr4	67,703,937	67,704,002	65	Loss
DEL00034878	chr4	67,765,109	67,765,727	618	Loss
DEL00034879	chr4	67,792,815	67,794,562	1,747	Loss
DEL00034885	chr4	67,889,281	67,889,417	136	Loss
DEL00034888	chr4	67,918,820	67,919,206	386	Loss
DEL00034889	chr4	67,924,804	67,924,912	108	Loss
DEL00034892	chr4	67,949,766	67,950,629	863	Loss
DEL00034903	chr4	68,039,132	68,039,438	306	Loss
DEL00034904	chr4	68,042,787	68,042,897	110	Loss
DEL00034913	chr4	68,210,277	68,210,436	159	Loss
DEL00034916	chr4	68,276,602	68,276,668	66	Loss
DEL00034917	chr4	68,312,457	68,312,596	139	Loss
DEL00034927	chr4	68,540,497	68,540,611	114	Loss
DEL00034928	chr4	68,541,662	68,541,719	57	Loss
DEL00034933	chr4	68,618,372	68,618,615	243	Loss
DEL00034939	chr4	68,686,586	68,686,697	111	Loss
DEL00034942	chr4	68,817,423	68,817,901	478	Loss
DEL00034949	chr4	68,921,435	68,921,660	225	Loss
DEL00034956	chr4	69,212,941	69,213,212	271	Loss
DEL00034962	chr4	69,312,612	69,313,371	759	Loss
DEL00034967	chr4	69,360,483	69,360,699	216	Loss
DEL00034969	chr4	69,374,745	69,375,127	382	Loss
DEL00034975	chr4	69,397,549	69,397,712	163	Loss
DEL00034976	chr4	69,421,411	69,421,672	261	Loss
DEL00034978	chr4	69,427,136	69,441,694	14,558	Loss
DEL00034979	chr4	69,454,269	69,454,329	60	Loss
DEL00034982	chr4	69,511,109	69,511,163	54	Loss
DEL00034984	chr4	69,558,980	69,559,264	284	Loss

DEL00034990	chr4	69,608,355	69,608,607	252	Loss
DEL00034994	chr4	69,618,397	69,618,832	435	Loss
DEL00034996	chr4	69,689,954	69,690,419	465	Loss
DEL00034998	chr4	69,701,367	69,701,537	170	Loss
DEL00035007	chr4	70,033,331	70,033,431	100	Loss
DEL00035015	chr4	70,157,381	70,157,918	537	Loss
DEL00035016	chr4	70,205,691	70,205,749	58	Loss
DEL00035021	chr4	70,369,184	70,369,809	625	Loss
DEL00035023	chr4	70,402,812	70,402,985	173	Loss
DEL00035025	chr4	70,445,839	70,446,367	528	Loss
DEL00035032	chr4	70,630,356	70,630,426	70	Loss
DEL00035033	chr4	70,630,741	70,631,301	560	Loss
DEL00035035	chr4	70,681,542	70,681,610	68	Loss
DEL00035039	chr4	70,748,847	70,748,949	102	Loss
DEL00035044	chr4	70,802,775	70,803,096	321	Loss
DUP00035045	chr4	70,808,166	70,841,447	33,281	Gain
DEL00035048	chr4	70,883,925	70,884,754	829	Loss
DEL00035050	chr4	70,915,228	70,915,735	507	Loss
DEL00035052	chr4	70,921,705	70,921,760	55	Loss
DEL00035054	chr4	70,924,955	70,925,379	424	Loss
DEL00035060	chr4	70,985,053	70,985,113	60	Loss
DEL00035067	chr4	71,037,047	71,037,394	347	Loss
DEL00035076	chr4	71,097,966	71,099,449	1,483	Loss
DEL00035077	chr4	71,115,003	71,116,139	1,136	Loss
DEL00035090	chr4	71,189,471	71,189,961	490	Loss
DEL00035096	chr4	71,293,489	71,293,550	61	Loss
DEL00035104	chr4	71,572,723	71,575,322	2,599	Loss
DEL00035106	chr4	71,712,668	71,712,860	192	Loss
DEL00035108	chr4	71,763,610	71,764,530	920	Loss
DEL00035109	chr4	71,787,167	71,787,719	552	Loss
DEL00035117	chr4	71,899,613	71,900,634	1,021	Loss
DEL00035119	chr4	71,916,107	71,916,622	515	Loss
DEL00035120	chr4	71,934,255	71,934,779	524	Loss
DEL00035123	chr4	71,977,187	71,978,279	1,092	Loss
DEL00035124	chr4	71,979,461	71,979,960	499	Loss
DEL00035131	chr4	72,010,761	72,013,487	2,726	Loss
DEL00035132	chr4	72,014,146	72,014,267	121	Loss
DEL00035140	chr4	72,162,206	72,162,453	247	Loss
DEL00035141	chr4	72,180,872	72,184,313	3,441	Loss
DEL00035143	chr4	72,211,734	72,213,493	1,759	Loss
DEL00035144	chr4	72,237,368	72,237,460	92	Loss
DEL00035148	chr4	72,284,694	72,284,790	96	Loss
DEL00035152	chr4	72,328,673	72,329,509	836	Loss
DEL00035162	chr4	72,375,684	72,375,948	264	Loss
DEL00035163	chr4	72,422,277	72,423,295	1,018	Loss
DEL00035168	chr4	72,456,746	72,457,673	927	Loss
DEL00035177	chr4	72,648,173	72,648,292	119	Loss
DEL00035179	chr4	72,684,316	72,684,670	354	Loss
DEL00035189	chr4	73,057,789	73,057,960	171	Loss

DEL00035202	chr4	73,252,817	73,253,203	386	Loss
DEL00035208	chr4	73,364,200	73,365,113	913	Loss
DEL00035210	chr4	73,385,157	73,385,225	68	Loss
DEL00035214	chr4	73,467,047	73,467,107	60	Loss
DEL00035216	chr4	73,485,743	73,485,802	59	Loss
DEL00035235	chr4	74,271,221	74,277,054	5,833	Loss
DEL00035237	chr4	74,304,343	74,304,455	112	Loss
DEL00035240	chr4	74,317,914	74,317,977	63	Loss
DEL00035248	chr4	74,465,032	74,465,361	329	Loss
DEL00035252	chr4	74,486,006	74,487,365	1,359	Loss
DEL00035259	chr4	74,637,355	74,637,518	163	Loss
DEL00035263	chr4	74,686,498	74,686,551	53	Loss
DEL00035264	chr4	74,733,769	74,733,829	60	Loss
DEL00035266	chr4	74,778,026	74,779,068	1,042	Loss
DEL00035267	chr4	74,780,481	74,780,713	232	Loss
DEL00035271	chr4	74,856,038	74,856,880	842	Loss
DEL00035273	chr4	74,883,781	74,883,833	52	Loss
DEL00035281	chr4	74,970,321	74,970,946	625	Loss
DEL00035282	chr4	74,986,725	74,987,294	569	Loss
DEL00035283	chr4	74,989,426	74,989,482	56	Loss
DEL00035284	chr4	74,990,253	74,991,576	1,323	Loss
DEL00035291	chr4	75,119,798	75,119,935	137	Loss
DEL00035296	chr4	75,229,697	75,230,434	737	Loss
DEL00035297	chr4	75,236,052	75,236,123	71	Loss
DEL00035301	chr4	75,264,886	75,264,989	103	Loss
DEL00035305	chr4	75,306,951	75,307,113	162	Loss
DEL00035311	chr4	75,429,881	75,430,509	628	Loss
DEL00035312	chr4	75,441,056	75,441,623	567	Loss
DEL00035316	chr4	75,485,034	75,485,270	236	Loss
DEL00035322	chr4	75,599,254	75,599,363	109	Loss
DEL00035324	chr4	75,662,528	75,662,600	72	Loss
DEL00035330	chr4	75,752,612	75,752,689	77	Loss
DEL00035334	chr4	75,821,071	75,821,182	111	Loss
DEL00035336	chr4	75,882,077	75,882,619	542	Loss
DEL00035339	chr4	76,019,681	76,020,779	1,098	Loss
DEL00035343	chr4	76,060,249	76,060,706	457	Loss
DEL00035346	chr4	76,134,275	76,134,807	532	Loss
DEL00035351	chr4	76,183,789	76,183,854	65	Loss
DEL00035363	chr4	76,470,602	76,470,797	195	Loss
DEL00035368	chr4	76,519,477	76,519,568	91	Loss
DEL00035376	chr4	76,648,801	76,649,573	772	Loss
DEL00035378	chr4	76,690,731	76,690,822	91	Loss
DEL00035379	chr4	76,702,342	76,702,542	200	Loss
DEL00035380	chr4	76,708,087	76,708,184	97	Loss
DEL00035385	chr4	76,769,987	76,770,083	96	Loss
DEL00035386	chr4	76,785,225	76,785,289	64	Loss
DEL00035389	chr4	76,874,611	76,874,682	71	Loss
DEL00035395	chr4	77,018,277	77,018,888	611	Loss
DEL00035404	chr4	77,124,281	77,124,469	188	Loss

DEL00035417	chr4	77,350,871	77,351,341	470	Loss
DEL00035424	chr4	77,470,124	77,471,168	1,044	Loss
DEL00035425	chr4	77,492,948	77,493,042	94	Loss
DEL00035426	chr4	77,520,405	77,520,458	53	Loss
DEL00035434	chr4	77,616,065	77,616,556	491	Loss
DEL00035435	chr4	77,625,942	77,626,401	459	Loss
DEL00035445	chr4	77,805,878	77,808,198	2,320	Loss
DEL00035446	chr4	77,813,890	77,813,963	73	Loss
DEL00035448	chr4	77,836,529	77,836,682	153	Loss
DEL00035449	chr4	77,868,880	77,869,207	327	Loss
DEL00035454	chr4	77,993,376	77,993,455	79	Loss
DEL00035455	chr4	78,036,167	78,036,585	418	Loss
DEL00035457	chr4	78,042,741	78,042,952	211	Loss
DEL00035459	chr4	78,096,261	78,096,375	114	Loss
DEL00035461	chr4	78,224,214	78,224,285	71	Loss
DEL00035462	chr4	78,259,320	78,259,428	108	Loss
DEL00035463	chr4	78,287,525	78,287,577	52	Loss
DEL00035467	chr4	78,472,993	78,473,174	181	Loss
DEL00035471	chr4	78,488,716	78,489,077	361	Loss
DEL00035480	chr4	78,561,199	78,561,473	274	Loss
DEL00035488	chr4	78,765,902	78,766,997	1,095	Loss
DEL00035492	chr4	78,806,240	78,806,749	509	Loss
DEL00035510	chr4	79,105,077	79,105,181	104	Loss
DUP00035517	chr4	79,238,407	79,238,537	130	Gain
DEL00035520	chr4	79,254,516	79,254,708	192	Loss
DEL00035524	chr4	79,275,073	79,275,211	138	Loss
DEL00035525	chr4	79,290,795	79,291,324	529	Loss
DEL00035532	chr4	79,347,314	79,347,742	428	Loss
DEL00035533	chr4	79,353,993	79,354,455	462	Loss
DEL00035538	chr4	79,387,846	79,387,953	107	Loss
DEL00035539	chr4	79,393,640	79,393,820	180	Loss
DEL00035548	chr4	79,555,800	79,555,855	55	Loss
DEL00035551	chr4	79,611,601	79,612,159	558	Loss
DEL00035554	chr4	79,658,538	79,658,592	54	Loss
DEL00035563	chr4	79,823,732	79,823,829	97	Loss
DEL00035565	chr4	79,830,224	79,830,324	100	Loss
DEL00035572	chr4	80,038,088	80,039,067	979	Loss
DEL00035574	chr4	80,116,419	80,116,505	86	Loss
DEL00035576	chr4	80,123,764	80,124,004	240	Loss
DEL00035577	chr4	80,136,513	80,136,682	169	Loss
DEL00035578	chr4	80,144,379	80,145,908	1,529	Loss
DEL00035581	chr4	80,204,389	80,204,583	194	Loss
DEL00035583	chr4	80,207,671	80,208,305	634	Loss
DEL00035587	chr4	80,277,684	80,277,736	52	Loss
DEL00035594	chr4	80,366,577	80,366,726	149	Loss
DEL00035611	chr4	80,638,662	80,638,745	83	Loss
DUP00035615	chr4	80,687,438	80,687,532	94	Gain
DEL00035620	chr4	80,795,866	80,796,292	426	Loss
DEL00035621	chr4	80,802,896	80,803,009	113	Loss

DEL00035623	chr4	80,813,699	80,813,843	144	Loss
DEL00035625	chr4	80,834,812	80,834,891	79	Loss
DEL00035628	chr4	80,901,833	80,902,119	286	Loss
DEL00035642	chr4	81,099,990	81,100,897	907	Loss
DEL00035645	chr4	81,165,234	81,165,962	728	Loss
DEL00035655	chr4	81,245,234	81,245,598	364	Loss
DEL00035665	chr4	81,321,746	81,321,809	63	Loss
DEL00035668	chr4	81,415,729	81,417,101	1,372	Loss
DEL00035669	chr4	81,429,005	81,430,070	1,065	Loss
DEL00035675	chr4	81,493,680	81,493,745	65	Loss
DEL00035679	chr4	81,601,210	81,601,261	51	Loss
DEL00035680	chr4	81,611,878	81,612,400	522	Loss
DEL00035697	chr4	81,925,273	81,925,379	106	Loss
DEL00035705	chr4	81,999,452	81,999,554	102	Loss
DEL00035706	chr4	82,009,722	82,010,662	940	Loss
DUP00035709	chr4	82,109,779	82,110,781	1,002	Gain
DEL00035710	chr4	82,113,385	82,113,453	68	Loss
DEL00035715	chr4	82,209,377	82,209,631	254	Loss
DEL00035716	chr4	82,222,600	82,222,879	279	Loss
DEL00035719	chr4	82,254,506	82,254,586	80	Loss
DEL00035722	chr4	82,288,902	82,289,108	206	Loss
DEL00035725	chr4	82,344,555	82,344,760	205	Loss
DEL00035729	chr4	82,431,174	82,431,408	234	Loss
DEL00035731	chr4	82,464,637	82,464,699	62	Loss
DEL00035734	chr4	82,488,381	82,488,531	150	Loss
DEL00035738	chr4	82,548,250	82,548,621	371	Loss
DEL00035741	chr4	82,575,524	82,575,729	205	Loss
DEL00035742	chr4	82,667,478	82,667,565	87	Loss
DEL00035746	chr4	82,724,087	82,724,637	550	Loss
DEL00035748	chr4	82,741,722	82,742,035	313	Loss
DEL00035749	chr4	82,778,215	82,784,048	5,833	Loss
DEL00035753	chr4	82,846,889	82,846,950	61	Loss
DEL00035756	chr4	82,884,689	82,884,913	224	Loss
DEL00035763	chr4	82,968,190	82,968,737	547	Loss
DEL00035769	chr4	83,076,720	83,076,868	148	Loss
DEL00035779	chr4	83,502,716	83,502,767	51	Loss
DEL00035780	chr4	83,508,451	83,508,514	63	Loss
DEL00035782	chr4	83,527,675	83,528,072	397	Loss
DEL00035784	chr4	83,536,383	83,536,490	107	Loss
DUP00035787	chr4	83,592,377	83,592,529	152	Gain
DEL00035788	chr4	83,595,533	83,595,586	53	Loss
DEL00035789	chr4	83,603,955	83,604,066	111	Loss
DEL00035791	chr4	83,645,936	83,647,072	1,136	Loss
DUP00035801	chr4	83,735,198	83,735,877	679	Gain
DEL00035804	chr4	83,766,747	83,766,812	65	Loss
DEL00035806	chr4	83,783,091	83,783,159	68	Loss
DEL00035808	chr4	83,808,073	83,808,346	273	Loss
DEL00035814	chr4	83,913,204	83,913,320	116	Loss
DEL00035817	chr4	83,935,892	83,936,518	626	Loss

DEL00035820	chr4	83,951,664	83,951,761	97	Loss
DEL00035831	chr4	84,177,769	84,178,246	477	Loss
DEL00035834	chr4	84,187,340	84,187,656	316	Loss
DEL00035845	chr4	84,233,520	84,233,976	456	Loss
DEL00035853	chr4	84,237,363	84,237,792	429	Loss
DEL00035854	chr4	84,267,104	84,267,482	378	Loss
DEL00035861	chr4	84,310,961	84,311,521	560	Loss
DEL00035863	chr4	84,317,520	84,318,124	604	Loss
DEL00035869	chr4	84,357,821	84,357,886	65	Loss
DEL00035882	chr4	84,555,585	84,555,669	84	Loss
DEL00035883	chr4	84,560,072	84,560,135	63	Loss
DEL00035890	chr4	84,784,885	84,785,085	200	Loss
DEL00035895	chr4	84,824,228	84,825,082	854	Loss
DEL00035896	chr4	84,913,210	84,913,346	136	Loss
DEL00035904	chr4	85,071,591	85,072,179	588	Loss
DEL00035912	chr4	85,099,403	85,099,627	224	Loss
DEL00035917	chr4	85,198,854	85,198,942	88	Loss
DUP00035918	chr4	85,203,669	85,205,350	1,681	Gain
DEL00035926	chr4	85,285,115	85,285,192	77	Loss
DEL00035938	chr4	85,487,672	85,487,962	290	Loss
DUP00035953	chr4	85,530,517	85,634,812	104,295	Mixed
DUP00036500	chr4	85,642,797	85,677,630	34,833	Mixed
DEL00036585	chr4	85,683,821	85,688,785	4,964	Loss
DEL00036593	chr4	85,729,201	85,729,475	274	Loss
DEL00036594	chr4	85,741,347	85,742,220	873	Loss
DEL00036595	chr4	85,751,766	85,752,234	468	Loss
DEL00036597	chr4	85,765,749	85,766,062	313	Loss
DEL00036599	chr4	85,792,247	85,792,771	524	Loss
DEL00036611	chr4	85,923,636	85,923,698	62	Loss
DEL00036618	chr4	85,982,225	85,982,440	215	Loss
DEL00036619	chr4	85,989,335	85,989,423	88	Loss
DEL00036620	chr4	85,989,902	85,989,976	74	Loss
DEL00036627	chr4	86,023,306	86,023,423	117	Loss
DEL00036630	chr4	86,059,122	86,059,735	613	Loss
DEL00036645	chr4	86,235,945	86,284,008	48,063	Loss
DEL00036655	chr4	86,287,577	86,287,661	84	Loss
DEL00036659	chr4	86,339,743	86,340,114	371	Loss
DEL00036662	chr4	86,359,934	86,361,037	1,103	Loss
DEL00036664	chr4	86,400,889	86,401,294	405	Loss
DEL00036667	chr4	86,429,857	86,429,912	55	Loss
DEL00036680	chr4	86,587,119	86,587,525	406	Loss
DEL00036684	chr4	86,627,072	86,627,129	57	Loss
DEL00036716	chr4	87,085,033	87,086,397	1,364	Loss
DEL00036732	chr4	87,366,590	87,367,107	517	Loss
DUP00036733	chr4	87,390,402	87,391,384	982	Gain
DEL00036742	chr4	87,422,482	87,423,240	758	Loss
DEL00036746	chr4	87,475,059	87,475,110	51	Loss
DEL00036752	chr4	87,568,687	87,568,792	105	Loss
DEL00036754	chr4	87,585,415	87,586,384	969	Loss

DEL00036764	chr4	87,668,140	87,668,220	80	Loss
DEL00036776	chr4	87,850,852	87,851,425	573	Loss
DEL00036778	chr4	87,880,995	87,881,087	92	Loss
DEL00036779	chr4	87,894,526	87,895,421	895	Loss
DEL00036792	chr4	88,050,111	88,050,200	89	Loss
DEL00036800	chr4	88,150,971	88,151,997	1,026	Loss
DEL00036801	chr4	88,152,175	88,152,475	300	Loss
DEL00036812	chr4	88,284,196	88,284,584	388	Loss
DEL00036828	chr4	88,404,549	88,404,603	54	Loss
DEL00036832	chr4	88,441,553	88,441,655	102	Loss
DEL00036835	chr4	88,564,806	88,564,871	65	Loss
DEL00036841	chr4	88,639,517	88,639,581	64	Loss
DEL00036844	chr4	88,737,626	88,737,692	66	Loss
DEL00036852	chr4	88,872,990	88,873,386	396	Loss
DEL00036853	chr4	88,873,971	88,874,027	56	Loss
DEL00036854	chr4	88,876,780	88,877,066	286	Loss
DUP00036857	chr4	88,936,743	88,936,856	113	Gain
DEL00036866	chr4	88,981,667	88,981,767	100	Loss
DEL00036867	chr4	88,988,584	88,989,229	645	Loss
DEL00036891	chr4	89,146,374	89,146,431	57	Loss
DEL00036905	chr4	89,289,293	89,290,012	719	Loss
DEL00036916	chr4	89,389,515	89,389,725	210	Loss
DEL00036918	chr4	89,427,953	89,428,346	393	Loss
DEL00036931	chr4	89,495,949	89,496,041	92	Loss
DEL00036933	chr4	89,513,976	89,514,479	503	Loss
DEL00036951	chr4	89,663,459	89,663,541	82	Loss
DEL00036972	chr4	89,755,537	89,755,828	291	Loss
DEL00036975	chr4	89,780,663	89,780,801	138	Loss
DEL00036987	chr4	89,850,183	89,850,324	141	Loss
DEL00036990	chr4	89,889,529	89,890,056	527	Loss
DEL00036992	chr4	89,914,589	89,915,089	500	Loss
DEL00037001	chr4	90,090,022	90,090,089	67	Loss
DEL00037002	chr4	90,106,920	90,106,994	74	Loss
DEL00037006	chr4	90,178,070	90,178,122	52	Loss
DEL00037017	chr4	90,305,098	90,305,636	538	Loss
DEL00037022	chr4	90,422,544	90,422,689	145	Loss
DEL00037023	chr4	90,431,685	90,438,044	6,359	Loss
DUP00037025	chr4	90,484,779	90,485,591	812	Gain
DEL00037034	chr4	90,488,444	90,488,508	64	Loss
DUP00037052	chr4	90,498,482	90,498,877	395	Gain
DUP00037054	chr4	90,507,255	90,507,416	161	Mixed
DEL00037102	chr4	90,538,156	90,539,595	1,439	Mixed
DEL00037119	chr4	90,540,629	90,540,819	190	Loss
DUP00037156	chr4	90,555,140	90,555,303	163	Gain
DUP00037227	chr4	90,777,207	90,777,322	115	Gain
DEL00037229	chr4	90,857,918	90,857,995	77	Loss
DUP00037255	chr4	90,962,511	90,965,506	2,995	Gain
DEL00037262	chr4	90,980,118	90,980,224	106	Loss
DEL00037276	chr4	90,991,932	90,992,008	76	Loss

DEL00037298	chr5	132,948	133,013	65	Loss
DEL00037305	chr5	207,657	207,731	74	Loss
DEL00037324	chr5	446,564	447,990	1,426	Loss
DEL00037333	chr5	479,081	488,634	9,553	Loss
DEL00037334	chr5	526,702	526,758	56	Loss
DEL00037344	chr5	917,181	917,282	101	Loss
DEL00037346	chr5	990,159	990,733	574	Loss
DEL00037372	chr5	1,224,919	1,226,083	1,164	Loss
DEL00037378	chr5	1,302,114	1,302,169	55	Loss
DEL00037379	chr5	1,312,690	1,312,872	182	Loss
DEL00037381	chr5	1,329,730	1,330,024	294	Loss
DUP00037388	chr5	1,423,542	1,423,617	75	Gain
DEL00037389	chr5	1,435,729	1,436,316	587	Loss
DEL00037391	chr5	1,458,144	1,458,229	85	Loss
DEL00037398	chr5	1,530,505	1,530,677	172	Loss
DEL00037399	chr5	1,618,061	1,618,228	167	Loss
DEL00037401	chr5	1,676,387	1,676,642	255	Loss
DEL00037412	chr5	1,863,881	1,864,032	151	Loss
DEL00037419	chr5	1,974,773	1,974,956	183	Loss
DEL00037421	chr5	2,003,080	2,003,157	77	Loss
DEL00037427	chr5	2,111,284	2,111,472	188	Loss
DEL00037430	chr5	2,232,875	2,233,054	179	Loss
DEL00037431	chr5	2,284,635	2,284,784	149	Loss
DEL00037432	chr5	2,286,545	2,287,180	635	Loss
DEL00037446	chr5	2,454,473	2,454,548	75	Loss
DEL00037453	chr5	2,715,797	2,716,295	498	Loss
DEL00037456	chr5	2,817,084	2,824,444	7,360	Loss
DEL00037461	chr5	2,891,145	2,896,358	5,213	Loss
DUP00037462	chr5	2,984,764	2,984,942	178	Gain
DEL00037467	chr5	3,066,184	3,066,473	289	Loss
DEL00037469	chr5	3,072,286	3,073,046	760	Loss
DEL00037474	chr5	3,274,417	3,293,318	18,901	Loss
DEL00037477	chr5	3,299,253	3,301,579	2,326	Loss
DEL00037487	chr5	3,519,247	3,519,473	226	Loss
DEL00037489	chr5	3,562,467	3,565,903	3,436	Loss
DUP00037496	chr5	3,665,998	3,666,172	174	Gain
DEL00037498	chr5	3,722,470	3,722,536	66	Loss
DEL00037499	chr5	3,788,016	3,788,075	59	Loss
DEL00037510	chr5	4,131,142	4,131,598	456	Loss
DEL00037513	chr5	4,201,501	4,202,423	922	Loss
DEL00037514	chr5	4,203,339	4,205,287	1,948	Loss
DEL00037528	chr5	4,476,089	4,476,436	347	Loss
DEL00037533	chr5	4,503,201	4,503,482	281	Loss
DEL00037536	chr5	4,548,040	4,552,423	4,383	Loss
DEL00037538	chr5	4,572,963	4,573,145	182	Loss
DEL00037542	chr5	4,664,760	4,665,360	600	Loss
DEL00037546	chr5	4,714,610	4,714,944	334	Loss
DEL00037563	chr5	5,004,356	5,004,851	495	Loss
DEL00037569	chr5	5,056,851	5,057,380	529	Loss

DEL00037579	chr5	5,168,507	5,168,838	331	Loss
DEL00037585	chr5	5,217,988	5,218,319	331	Loss
DEL00037586	chr5	5,249,932	5,250,027	95	Loss
DEL00037588	chr5	5,268,233	5,269,040	807	Loss
DEL00037592	chr5	5,460,249	5,460,354	105	Loss
DEL00037599	chr5	5,721,760	5,721,845	85	Loss
DEL00037602	chr5	5,797,980	5,798,078	98	Loss
DEL00037603	chr5	5,805,282	5,805,514	232	Loss
DEL00037655	chr5	6,036,154	6,038,137	1,983	Loss
DEL00037667	chr5	6,104,429	6,104,503	74	Loss
DEL00037675	chr5	6,152,024	6,152,087	63	Loss
DEL00037684	chr5	6,298,365	6,298,497	132	Loss
DEL00037686	chr5	6,332,656	6,332,725	69	Loss
DEL00037701	chr5	6,573,592	6,573,852	260	Loss
DEL00037703	chr5	6,635,290	6,635,356	66	Loss
DEL00037704	chr5	6,660,100	6,660,158	58	Loss
DEL00037707	chr5	6,705,321	6,705,386	65	Loss
DEL00037725	chr5	6,960,094	6,960,162	68	Loss
DEL00037726	chr5	6,965,372	6,965,429	57	Loss
DEL00037739	chr5	7,173,330	7,174,125	795	Loss
DEL00037751	chr5	7,356,107	7,356,259	152	Loss
DEL00037754	chr5	7,432,728	7,434,419	1,691	Loss
DEL00037756	chr5	7,457,385	7,457,476	91	Loss
DEL00037780	chr5	7,823,726	7,823,784	58	Loss
DEL00037781	chr5	7,834,268	7,834,387	119	Loss
DEL00037784	chr5	7,890,338	7,890,594	256	Loss
DEL00037803	chr5	8,094,459	8,094,685	226	Loss
DEL00037809	chr5	8,168,952	8,169,235	283	Loss
DUP00037810	chr5	8,191,568	8,191,665	97	Gain
DEL00037812	chr5	8,232,038	8,232,258	220	Loss
DEL00037813	chr5	8,269,613	8,270,687	1,074	Loss
DUP00037814	chr5	8,305,234	8,314,990	9,756	Mixed
DEL00037816	chr5	8,345,177	8,345,243	66	Loss
DEL00037821	chr5	8,360,564	8,360,726	162	Loss
DEL00037827	chr5	8,497,780	8,499,460	1,680	Loss
DEL00037829	chr5	8,500,372	8,500,542	170	Loss
DEL00037830	chr5	8,532,361	8,532,440	79	Loss
DEL00037835	chr5	8,554,197	8,554,527	330	Loss
DEL00037839	chr5	8,608,731	8,609,164	433	Loss
DUP00037841	chr5	8,639,128	8,639,199	71	Gain
DEL00037843	chr5	8,674,533	8,674,649	116	Loss
DEL00037845	chr5	8,681,437	8,681,536	99	Loss
DEL00037850	chr5	8,746,529	8,746,838	309	Loss
DEL00037855	chr5	8,805,534	8,805,844	310	Loss
DEL00037857	chr5	8,852,504	8,852,722	218	Loss
DEL00037858	chr5	8,877,348	8,877,900	552	Loss
DEL00037868	chr5	8,957,750	8,957,811	61	Loss
DEL00037870	chr5	9,008,850	9,008,910	60	Loss
DEL00037871	chr5	9,030,579	9,032,516	1,937	Loss

DEL00037884	chr5	9,178,511	9,178,647	136	Loss
DEL00037885	chr5	9,183,980	9,184,056	76	Loss
DEL00037892	chr5	9,332,724	9,333,266	542	Loss
DEL00037893	chr5	9,334,621	9,334,699	78	Loss
DEL00037904	chr5	9,731,409	9,731,497	88	Loss
DEL00037911	chr5	9,958,573	9,959,332	759	Loss
DEL00037916	chr5	9,988,725	9,988,860	135	Loss
DEL00037919	chr5	10,044,911	10,045,728	817	Loss
DEL00037920	chr5	10,048,519	10,048,987	468	Loss
DEL00037929	chr5	10,263,686	10,263,899	213	Loss
DEL00037936	chr5	10,394,085	10,394,137	52	Loss
DEL00037937	chr5	10,394,584	10,394,739	155	Loss
DEL00037941	chr5	10,512,011	10,512,076	65	Loss
DEL00037943	chr5	10,546,120	10,546,246	126	Loss
DEL00037945	chr5	10,573,728	10,573,788	60	Loss
DEL00037948	chr5	10,707,068	10,707,318	250	Loss
DEL00037952	chr5	10,755,374	10,755,473	99	Loss
DEL00037956	chr5	10,840,237	10,840,711	474	Loss
DEL00037975	chr5	11,333,803	11,335,841	2,038	Loss
DEL00037979	chr5	11,458,580	11,458,663	83	Loss
DEL00037984	chr5	11,504,849	11,504,911	62	Loss
DEL00037987	chr5	11,534,043	11,534,208	165	Loss
DEL00037995	chr5	11,693,780	11,693,887	107	Loss
DEL00037997	chr5	11,764,477	11,764,903	426	Loss
DEL00038003	chr5	11,799,388	11,799,471	83	Loss
DEL00038014	chr5	11,949,998	11,950,461	463	Loss
DEL00038017	chr5	12,007,565	12,008,034	469	Loss
DEL00038021	chr5	12,046,959	12,047,105	146	Loss
DEL00038022	chr5	12,054,402	12,054,831	429	Loss
DEL00038028	chr5	12,151,652	12,151,736	84	Loss
DEL00038034	chr5	12,241,513	12,242,090	577	Loss
DEL00038036	chr5	12,247,504	12,247,623	119	Loss
DEL00038044	chr5	12,493,597	12,493,653	56	Loss
DEL00038049	chr5	12,651,311	12,651,394	83	Loss
DEL00038051	chr5	12,715,399	12,715,652	253	Loss
DEL00038052	chr5	12,786,626	12,787,710	1,084	Loss
DEL00038056	chr5	12,853,353	12,853,497	144	Loss
DEL00038060	chr5	12,947,188	12,948,271	1,083	Loss
DEL00038066	chr5	13,052,439	13,052,514	75	Loss
DEL00038071	chr5	13,204,212	13,204,290	78	Loss
DEL00038075	chr5	13,221,403	13,221,974	571	Loss
DEL00038081	chr5	13,285,493	13,285,815	322	Loss
DEL00038084	chr5	13,342,148	13,342,286	138	Loss
DEL00038094	chr5	13,690,538	13,690,610	72	Loss
DEL00038096	chr5	13,693,916	13,695,689	1,773	Loss
DEL00038114	chr5	13,767,430	13,767,509	79	Loss
DEL00038119	chr5	13,890,527	13,890,954	427	Loss
DEL00038121	chr5	13,925,356	13,925,754	398	Loss
DEL00038130	chr5	14,053,394	14,054,027	633	Loss

DEL00038136	chr5	14,132,021	14,134,030	2,009	Loss
DEL00038137	chr5	14,136,558	14,136,749	191	Loss
DEL00038138	chr5	14,165,076	14,165,859	783	Loss
DEL00038140	chr5	14,185,258	14,185,618	360	Loss
DEL00038142	chr5	14,235,850	14,236,017	167	Loss
DEL00038143	chr5	14,239,962	14,240,048	86	Loss
DEL00038145	chr5	14,273,119	14,273,293	174	Loss
DEL00038149	chr5	14,330,545	14,330,961	416	Loss
DEL00038153	chr5	14,427,055	14,427,163	108	Loss
DEL00038178	chr5	14,785,629	14,785,807	178	Loss
DEL00038195	chr5	14,987,141	14,987,193	52	Loss
DEL00038196	chr5	15,002,992	15,003,044	52	Loss
DEL00038198	chr5	15,073,646	15,073,740	94	Loss
DEL00038203	chr5	15,133,536	15,133,677	141	Loss
DEL00038219	chr5	15,355,131	15,355,233	102	Loss
DEL00038222	chr5	15,387,616	15,387,970	354	Loss
DEL00038226	chr5	15,506,697	15,507,022	325	Loss
DEL00038227	chr5	15,531,949	15,532,003	54	Loss
DEL00038231	chr5	15,621,830	15,625,455	3,625	Loss
DUP00038235	chr5	15,664,181	15,670,041	5,860	Gain
DEL00038237	chr5	15,688,834	15,688,909	75	Loss
DEL00038238	chr5	15,713,992	15,714,155	163	Loss
DEL00038239	chr5	15,861,435	15,861,487	52	Loss
DEL00038242	chr5	15,927,927	15,928,003	76	Loss
DEL00038248	chr5	16,183,471	16,183,581	110	Loss
DEL00038249	chr5	16,189,412	16,189,492	80	Loss
DEL00038257	chr5	16,367,177	16,367,229	52	Loss
DEL00038263	chr5	16,452,697	16,452,788	91	Loss
DEL00038355	chr5	16,491,153	16,497,115	5,962	Loss
DEL00038397	chr5	16,739,400	16,739,863	463	Loss
DEL00038400	chr5	16,788,260	16,788,877	617	Loss
DEL00038407	chr5	16,958,338	16,958,760	422	Loss
DEL00038410	chr5	17,024,307	17,024,367	60	Loss
DEL00038411	chr5	17,095,325	17,095,470	145	Loss
DEL00038415	chr5	17,235,368	17,235,426	58	Loss
DEL00038420	chr5	17,310,442	17,311,131	689	Loss
DEL00038434	chr5	17,499,488	17,499,568	80	Loss
DEL00038441	chr5	17,589,596	17,589,670	74	Loss
DEL00038446	chr5	17,663,709	17,663,781	72	Loss
DEL00038455	chr5	17,736,704	17,736,791	87	Loss
DUP00038462	chr5	17,819,270	17,829,846	10,576	Gain
DEL00038466	chr5	17,902,350	17,903,970	1,620	Loss
DEL00038472	chr5	18,007,033	18,007,136	103	Loss
DEL00038474	chr5	18,019,280	18,019,753	473	Loss
DEL00038476	chr5	18,035,518	18,041,588	6,070	Loss
DEL00038477	chr5	18,166,861	18,167,848	987	Loss
DEL00038478	chr5	18,184,291	18,185,057	766	Loss
DEL00038479	chr5	18,216,779	18,216,903	124	Loss
DEL00038483	chr5	18,253,245	18,253,444	199	Loss

DEL00038487	chr5	18,262,217	18,262,437	220	Loss
DEL00038495	chr5	18,393,946	18,394,033	87	Loss
DUP00038497	chr5	18,458,446	18,467,681	9,235	Gain
DEL00038500	chr5	18,474,054	18,474,160	106	Loss
DEL00038503	chr5	18,534,024	18,534,082	58	Loss
DEL00038516	chr5	18,708,766	18,708,823	57	Loss
DEL00038525	chr5	18,816,688	18,816,778	90	Loss
DEL00038544	chr5	19,377,404	19,377,727	323	Loss
DEL00038546	chr5	19,421,912	19,422,079	167	Loss
DEL00038548	chr5	19,433,370	19,434,233	863	Loss
DEL00038551	chr5	19,463,834	19,463,999	165	Loss
DEL00038553	chr5	19,471,813	19,472,074	261	Loss
DEL00038554	chr5	19,523,730	19,524,405	675	Loss
DEL00038558	chr5	19,561,733	19,562,311	578	Loss
DEL00038559	chr5	19,576,644	19,577,163	519	Loss
DEL00038564	chr5	19,642,459	19,643,251	792	Loss
DEL00038572	chr5	19,706,659	19,706,766	107	Loss
DEL00038573	chr5	19,720,538	19,721,912	1,374	Loss
DEL00038574	chr5	19,732,869	19,734,231	1,362	Loss
DEL00038589	chr5	19,876,433	19,876,548	115	Loss
DEL00038597	chr5	19,947,474	19,947,946	472	Loss
DEL00038603	chr5	20,017,076	20,017,534	458	Loss
DEL00038610	chr5	20,097,872	20,098,158	286	Loss
DUP00038626	chr5	20,307,026	20,322,111	15,085	Gain
DEL00038630	chr5	20,429,170	20,429,274	104	Loss
DEL00038635	chr5	20,477,148	20,477,843	695	Loss
DEL00038636	chr5	20,482,422	20,482,475	53	Loss
DEL00038638	chr5	20,510,987	20,511,380	393	Loss
DEL00038652	chr5	20,719,374	20,719,462	88	Loss
DUP00038653	chr5	20,720,078	20,720,211	133	Gain
DEL00038654	chr5	20,727,051	20,727,108	57	Loss
DEL00038664	chr5	20,872,501	20,872,861	360	Loss
DEL00038673	chr5	21,122,777	21,123,518	741	Loss
DEL00038677	chr5	21,155,179	21,155,685	506	Loss
DEL00038680	chr5	21,196,690	21,196,743	53	Loss
DEL00038687	chr5	21,379,907	21,380,279	372	Loss
DEL00038692	chr5	21,530,203	21,536,036	5,833	Loss
DEL00038695	chr5	21,645,096	21,645,220	124	Loss
DEL00038696	chr5	21,646,466	21,646,880	414	Loss
DEL00038698	chr5	21,678,798	21,678,980	182	Loss
DEL00038702	chr5	22,207,510	22,207,867	357	Loss
DEL00038704	chr5	22,258,500	22,258,645	145	Loss
DEL00038706	chr5	22,391,692	22,391,747	55	Loss
DEL00038708	chr5	22,393,705	22,394,116	411	Loss
DEL00038710	chr5	22,433,883	22,433,969	86	Loss
DEL00038711	chr5	22,446,735	22,446,792	57	Loss
DEL00038722	chr5	22,680,104	22,680,422	318	Loss
DEL00038724	chr5	22,760,646	22,760,733	87	Loss
DEL00038736	chr5	22,908,371	22,908,429	58	Loss

DEL00038741	chr5	22,967,381	22,967,595	214	Loss
DEL00038748	chr5	23,026,967	23,027,042	75	Loss
DEL00038764	chr5	23,599,592	23,599,694	102	Loss
DEL00038770	chr5	23,729,430	23,730,242	812	Loss
DEL00038774	chr5	23,810,818	23,811,399	581	Loss
DEL00038777	chr5	23,842,366	23,842,521	155	Loss
DEL00038786	chr5	23,949,868	23,949,934	66	Loss
DEL00038789	chr5	23,990,353	23,990,619	266	Loss
DEL00038792	chr5	24,076,149	24,076,278	129	Loss
DEL00038794	chr5	24,127,167	24,137,744	10,577	Loss
DUP00038796	chr5	24,156,703	24,162,143	5,440	Gain
DUP00038803	chr5	24,276,617	24,277,209	592	Gain
DEL00038809	chr5	24,450,958	24,451,043	85	Loss
DUP00038812	chr5	24,589,428	24,589,590	162	Gain
DEL00038828	chr5	24,894,251	24,894,330	79	Loss
DEL00038836	chr5	25,036,783	25,037,074	291	Loss
DEL00038837	chr5	25,055,141	25,055,275	134	Loss
DEL00038839	chr5	25,072,671	25,073,310	639	Loss
DEL00038840	chr5	25,077,249	25,078,038	789	Loss
DEL00038844	chr5	25,133,323	25,133,385	62	Loss
DEL00038848	chr5	25,280,901	25,281,384	483	Loss
DEL00038851	chr5	25,433,950	25,434,229	279	Loss
DEL00038854	chr5	25,481,766	25,482,432	666	Loss
DEL00038857	chr5	25,506,459	25,506,766	307	Loss
DEL00038862	chr5	25,557,307	25,557,433	126	Loss
DEL00038865	chr5	25,634,476	25,634,814	338	Loss
DEL00038867	chr5	25,691,610	25,691,807	197	Loss
DEL00038873	chr5	25,766,787	25,767,075	288	Loss
DEL00038876	chr5	25,826,291	25,826,753	462	Loss
DEL00038892	chr5	26,256,426	26,257,306	880	Loss
DEL00038893	chr5	26,257,559	26,259,068	1,509	Loss
DEL00038896	chr5	26,293,666	26,294,180	514	Loss
DEL00038902	chr5	26,354,159	26,354,210	51	Loss
DEL00038910	chr5	26,434,454	26,434,637	183	Loss
DEL00038913	chr5	26,440,962	26,441,014	52	Loss
DEL00038919	chr5	26,455,416	26,455,550	134	Loss
DEL00038923	chr5	26,553,154	26,553,642	488	Loss
DEL00038925	chr5	26,594,409	26,596,063	1,654	Loss
DEL00038930	chr5	26,627,867	26,628,061	194	Loss
DEL00038933	chr5	26,681,222	26,681,471	249	Loss
DEL00038938	chr5	26,820,261	26,820,458	197	Loss
DEL00038939	chr5	26,821,425	26,821,540	115	Loss
DEL00038947	chr5	26,986,242	26,986,767	525	Loss
DEL00038953	chr5	27,005,158	27,010,712	5,554	Loss
DEL00038956	chr5	27,067,352	27,067,460	108	Loss
DEL00038974	chr5	27,250,400	27,250,573	173	Loss
DEL00038976	chr5	27,302,909	27,303,117	208	Loss
DEL00038977	chr5	27,358,238	27,358,308	70	Loss
DEL00038978	chr5	27,360,384	27,360,732	348	Loss

DEL00038979	chr5	27,362,667	27,362,721	54	Loss
DEL00038991	chr5	27,530,188	27,530,281	93	Loss
DEL00038993	chr5	27,557,997	27,558,475	478	Loss
DEL00038995	chr5	27,595,036	27,595,256	220	Loss
DEL00038998	chr5	27,637,677	27,637,792	115	Loss
DEL00039008	chr5	27,707,906	27,708,070	164	Loss
DEL00039017	chr5	27,892,454	27,892,561	107	Loss
DEL00039018	chr5	27,896,678	27,896,796	118	Loss
DEL00039021	chr5	27,995,727	27,996,104	377	Loss
DEL00039026	chr5	28,046,547	28,046,951	404	Loss
DEL00039033	chr5	28,137,276	28,137,695	419	Loss
DEL00039044	chr5	28,344,123	28,344,236	113	Loss
DEL00039050	chr5	28,440,034	28,440,118	84	Loss
DEL00039052	chr5	28,452,773	28,452,839	66	Loss
DEL00039054	chr5	28,489,493	28,489,745	252	Loss
DEL00039059	chr5	28,619,498	28,619,653	155	Loss
DEL00039074	chr5	28,796,230	28,797,186	956	Loss
DEL00039075	chr5	28,820,195	28,820,633	438	Loss
DEL00039082	chr5	28,948,814	28,948,965	151	Loss
DEL00039084	chr5	28,980,443	28,980,532	89	Loss
DEL00039089	chr5	29,065,433	29,065,588	155	Loss
DEL00039090	chr5	29,070,569	29,072,767	2,198	Loss
DEL00039091	chr5	29,088,853	29,089,651	798	Loss
DEL00039092	chr5	29,100,540	29,105,273	4,733	Loss
DEL00039103	chr5	29,274,635	29,274,838	203	Loss
DEL00039107	chr5	29,392,676	29,393,271	595	Loss
DEL00039108	chr5	29,414,676	29,415,772	1,096	Loss
DEL00039118	chr5	29,652,320	29,652,378	58	Loss
DEL00039122	chr5	29,747,120	29,747,885	765	Loss
DEL00039128	chr5	29,929,172	29,929,986	814	Loss
DEL00039146	chr5	30,106,128	30,106,295	167	Loss
DEL00039152	chr5	30,141,569	30,141,732	163	Loss
DUP00039154	chr5	30,169,583	30,170,372	789	Gain
DEL00039157	chr5	30,190,796	30,191,069	273	Loss
DEL00039158	chr5	30,212,975	30,213,088	113	Loss
DEL00039167	chr5	30,471,639	30,471,739	100	Loss
DEL00039172	chr5	30,613,828	30,613,936	108	Loss
DUP00039176	chr5	30,798,925	30,815,162	16,237	Gain
DEL00039183	chr5	30,952,925	30,953,013	88	Loss
DEL00039186	chr5	31,077,287	31,077,421	134	Loss
DEL00039191	chr5	31,198,723	31,198,882	159	Loss
DEL00039193	chr5	31,302,227	31,302,609	382	Loss
DEL00039194	chr5	31,312,730	31,312,794	64	Loss
DUP00039196	chr5	31,430,629	31,430,930	301	Gain
DEL00039198	chr5	31,470,221	31,470,310	89	Loss
DEL00039199	chr5	31,480,257	31,480,346	89	Loss
DEL00039201	chr5	31,499,673	31,499,764	91	Loss
DEL00039205	chr5	31,573,295	31,574,758	1,463	Loss
DEL00039212	chr5	31,736,606	31,737,871	1,265	Loss

DEL00039215	chr5	31,753,185	31,753,566	381	Loss
DEL00039217	chr5	31,823,737	31,823,813	76	Loss
DEL00039218	chr5	31,834,430	31,834,868	438	Loss
DEL00039222	chr5	31,861,036	31,863,533	2,497	Loss
DEL00039223	chr5	31,894,055	31,894,825	770	Loss
DEL00039234	chr5	31,950,054	31,950,135	81	Loss
DEL00039235	chr5	31,952,445	31,952,653	208	Loss
DEL00039239	chr5	32,000,635	32,001,215	580	Loss
DEL00039246	chr5	32,109,907	32,110,231	324	Loss
DEL00039251	chr5	32,121,547	32,122,414	867	Loss
DEL00039254	chr5	32,160,912	32,161,451	539	Loss
DEL00039258	chr5	32,217,230	32,221,455	4,225	Loss
DEL00039263	chr5	32,344,217	32,344,334	117	Loss
DEL00039267	chr5	32,561,341	32,561,562	221	Loss
DEL00039270	chr5	32,598,753	32,599,870	1,117	Loss
DEL00039275	chr5	32,718,615	32,718,789	174	Loss
DEL00039279	chr5	32,838,389	32,838,477	88	Loss
DEL00039280	chr5	32,840,369	32,840,543	174	Loss
DEL00039281	chr5	32,853,554	32,853,763	209	Loss
DEL00039285	chr5	32,986,396	32,986,959	563	Loss
DEL00039289	chr5	33,049,205	33,049,326	121	Loss
DEL00039292	chr5	33,091,831	33,092,047	216	Loss
DEL00039297	chr5	33,297,620	33,298,173	553	Loss
DEL00039298	chr5	33,300,156	33,300,591	435	Loss
DEL00039302	chr5	33,413,639	33,413,857	218	Loss
DEL00039304	chr5	33,436,262	33,436,348	86	Loss
DEL00039310	chr5	33,533,653	33,534,459	806	Loss
DEL00039311	chr5	33,564,320	33,564,570	250	Loss
DEL00039312	chr5	33,593,257	33,595,155	1,898	Loss
DEL00039317	chr5	33,651,229	33,651,788	559	Loss
DEL00039318	chr5	33,657,584	33,657,806	222	Loss
DEL00039319	chr5	33,664,169	33,664,236	67	Loss
DEL00039323	chr5	33,694,558	33,694,657	99	Loss
DEL00039324	chr5	33,695,242	33,695,434	192	Loss
DEL00039325	chr5	33,700,396	33,700,453	57	Loss
DEL00039326	chr5	33,702,802	33,702,853	51	Loss
DEL00039337	chr5	33,719,712	33,719,816	104	Loss
DEL00039339	chr5	33,748,353	33,748,834	481	Loss
DEL00039343	chr5	33,778,805	33,778,895	90	Loss
DUP00039350	chr5	33,924,027	33,962,483	38,456	Gain
DEL00039353	chr5	34,068,362	34,068,515	153	Loss
DEL00039355	chr5	34,117,373	34,117,642	269	Loss
DEL00039357	chr5	34,179,304	34,179,716	412	Loss
DEL00039358	chr5	34,188,886	34,189,047	161	Loss
DEL00039364	chr5	34,311,741	34,311,942	201	Loss
DUP00039365	chr5	34,328,948	34,335,771	6,823	Mixed
DEL00039374	chr5	34,424,336	34,424,551	215	Loss
DUP00039376	chr5	34,467,203	34,467,257	54	Gain
DEL00039389	chr5	34,702,110	34,702,601	491	Loss

DEL00039391	chr5	34,712,321	34,712,650	329	Loss
DEL00039393	chr5	34,754,509	34,754,561	52	Loss
DEL00039395	chr5	34,807,835	34,819,456	11,621	Loss
DEL00039398	chr5	34,819,597	34,819,860	263	Loss
DEL00039400	chr5	34,861,136	34,861,216	80	Loss
DEL00039401	chr5	34,877,809	34,877,903	94	Loss
DEL00039402	chr5	34,890,594	34,890,719	125	Loss
DEL00039407	chr5	34,980,762	34,986,596	5,834	Loss
DEL00039411	chr5	35,148,023	35,148,131	108	Loss
DEL00039414	chr5	35,251,394	35,251,960	566	Loss
DEL00039420	chr5	35,416,264	35,416,703	439	Loss
DEL00039422	chr5	35,446,568	35,446,640	72	Loss
DUP00039428	chr5	35,593,708	35,607,831	14,123	Gain
DEL00039432	chr5	35,646,509	35,646,717	208	Loss
DEL00039438	chr5	35,845,073	35,845,272	199	Loss
DUP00039439	chr5	35,859,665	35,888,962	29,297	Gain
DEL00039441	chr5	35,903,265	35,903,333	68	Loss
DEL00039458	chr5	36,359,880	36,360,695	815	Loss
DEL00039460	chr5	36,419,234	36,419,324	90	Loss
DEL00039461	chr5	36,434,118	36,434,189	71	Loss
DEL00039464	chr5	36,572,023	36,572,113	90	Loss
DEL00039467	chr5	36,678,000	36,678,068	68	Loss
DEL00039478	chr5	36,910,350	36,910,484	134	Loss
DEL00039483	chr5	37,024,499	37,024,551	52	Loss
DEL00039488	chr5	37,081,757	37,082,001	244	Loss
DEL00039495	chr5	37,213,705	37,213,785	80	Loss
DEL00039496	chr5	37,226,105	37,226,232	127	Loss
DEL00039497	chr5	37,258,504	37,258,588	84	Loss
DEL00039508	chr5	37,555,687	37,555,746	59	Loss
DEL00039515	chr5	37,824,159	37,824,268	109	Loss
DEL00039520	chr5	37,845,464	37,845,581	117	Loss
DEL00039523	chr5	37,912,479	37,912,980	501	Loss
DEL00039527	chr5	38,049,249	38,049,832	583	Loss
DEL00039528	chr5	38,097,888	38,098,095	207	Loss
DEL00039537	chr5	38,361,622	38,361,676	54	Loss
DEL00039545	chr5	38,573,753	38,573,867	114	Loss
DEL00039547	chr5	38,611,349	38,611,456	107	Loss
DEL00039549	chr5	38,661,406	38,661,459	53	Loss
DEL00039551	chr5	38,689,905	38,690,379	474	Loss
DEL00039565	chr5	38,939,700	38,941,459	1,759	Loss
DEL00039569	chr5	38,966,023	38,966,091	68	Loss
DEL00039572	chr5	39,061,401	39,061,566	165	Loss
DEL00039573	chr5	39,075,662	39,075,745	83	Loss
DEL00039574	chr5	39,112,466	39,113,066	600	Loss
DEL00039578	chr5	39,208,169	39,208,329	160	Loss
DEL00039582	chr5	39,269,446	39,269,771	325	Loss
DEL00039592	chr5	39,488,018	39,488,710	692	Loss
DEL00039599	chr5	39,759,349	39,759,728	379	Loss
DEL00039611	chr5	40,083,115	40,084,347	1,232	Loss

DEL00039615	chr5	40,161,722	40,161,806	84	Loss
DEL00039618	chr5	40,221,867	40,222,476	609	Loss
DEL00039619	chr5	40,228,851	40,228,999	148	Loss
DEL00039623	chr5	40,285,772	40,286,522	750	Loss
DEL00039626	chr5	40,354,782	40,355,041	259	Loss
DEL00039638	chr5	40,539,974	40,540,025	51	Loss
DEL00039651	chr5	40,919,176	40,919,301	125	Loss
DEL00039654	chr5	41,049,277	41,049,840	563	Loss
DEL00039659	chr5	41,163,456	41,164,134	678	Loss
DEL00039660	chr5	41,178,802	41,179,622	820	Loss
DEL00039661	chr5	41,182,047	41,182,123	76	Loss
DEL00039662	chr5	41,182,736	41,183,191	455	Loss
DEL00039665	chr5	41,206,724	41,206,877	153	Loss
DEL00039668	chr5	41,344,925	41,345,057	132	Loss
DEL00039669	chr5	41,362,038	41,362,096	58	Loss
DEL00039672	chr5	41,495,854	41,515,585	19,731	Loss
DEL00039675	chr5	41,523,781	41,523,919	138	Loss
DEL00039687	chr5	41,758,790	41,759,212	422	Loss
DEL00039688	chr5	41,762,966	41,763,875	909	Loss
DEL00039692	chr5	41,771,923	41,771,991	68	Loss
DEL00039693	chr5	41,796,686	41,796,743	57	Loss
DEL00039695	chr5	41,838,896	41,838,998	102	Loss
DEL00039697	chr5	41,859,948	41,860,136	188	Loss
DEL00039704	chr5	42,082,863	42,083,441	578	Loss
DEL00039708	chr5	42,268,530	42,270,161	1,631	Loss
DEL00039709	chr5	42,309,223	42,309,722	499	Loss
DEL00039713	chr5	42,429,309	42,429,406	97	Loss
DEL00039714	chr5	42,436,417	42,436,485	68	Loss
DEL00039721	chr5	42,637,198	42,638,087	889	Loss
DEL00039724	chr5	42,657,837	42,657,897	60	Loss
DEL00039726	chr5	42,718,553	42,718,662	109	Loss
DEL00039730	chr5	42,760,426	42,760,917	491	Loss
DEL00039734	chr5	42,839,220	42,839,350	130	Loss
DEL00039737	chr5	42,866,801	42,866,853	52	Loss
DEL00039738	chr5	42,883,735	42,883,958	223	Loss
DEL00039740	chr5	42,957,580	42,957,696	116	Loss
DEL00039743	chr5	43,065,746	43,066,469	723	Loss
DEL00039745	chr5	43,109,007	43,109,204	197	Loss
DUP00039748	chr5	43,117,473	43,146,559	29,086	Mixed
DEL00039756	chr5	43,180,494	43,180,799	305	Loss
DEL00039759	chr5	43,214,044	43,214,428	384	Loss
DEL00039762	chr5	43,218,634	43,218,897	263	Loss
DEL00039765	chr5	43,255,528	43,255,762	234	Loss
DEL00039767	chr5	43,295,119	43,295,297	178	Loss
DEL00039777	chr5	43,420,145	43,420,774	629	Loss
DEL00039786	chr5	43,542,772	43,543,340	568	Loss
DEL00039787	chr5	43,562,087	43,566,157	4,070	Loss
DEL00039790	chr5	43,600,579	43,601,985	1,406	Loss
DEL00039792	chr5	43,614,759	43,614,826	67	Loss

DEL00039804	chr5	43,865,493	43,866,539	1,046	Loss
DEL00039805	chr5	43,889,861	43,889,985	124	Loss
DEL00039817	chr5	44,106,482	44,106,815	333	Loss
DEL00039826	chr5	44,187,156	44,187,246	90	Loss
DEL00039827	chr5	44,203,680	44,204,355	675	Loss
DEL00039833	chr5	44,233,944	44,234,087	143	Loss
DEL00039834	chr5	44,245,759	44,246,426	667	Loss
DEL00039835	chr5	44,249,321	44,249,478	157	Loss
DEL00039845	chr5	44,310,807	44,310,904	97	Loss
DEL00039850	chr5	44,361,337	44,362,210	873	Loss
DEL00039852	chr5	44,394,762	44,394,870	108	Loss
DEL00039856	chr5	44,425,498	44,425,737	239	Loss
DEL00039861	chr5	44,542,915	44,542,972	57	Loss
DEL00039879	chr5	44,911,945	44,912,390	445	Loss
DEL00039890	chr5	45,054,261	45,054,422	161	Loss
DEL00039891	chr5	45,071,892	45,071,973	81	Loss
DEL00039892	chr5	45,080,448	45,081,586	1,138	Loss
DEL00039893	chr5	45,081,687	45,081,742	55	Loss
DEL00039902	chr5	45,278,903	45,279,106	203	Loss
DEL00039904	chr5	45,308,225	45,308,276	51	Loss
DEL00039908	chr5	45,343,507	45,343,821	314	Loss
DEL00039913	chr5	45,374,062	45,374,133	71	Loss
DEL00039914	chr5	45,432,231	45,432,285	54	Loss
DEL00039920	chr5	45,513,411	45,513,466	55	Loss
DEL00039921	chr5	45,524,193	45,524,260	67	Loss
DEL00039923	chr5	45,527,110	45,527,303	193	Loss
DEL00039924	chr5	45,530,131	45,530,225	94	Loss
DEL00039929	chr5	45,616,644	45,616,959	315	Loss
DEL00039942	chr5	45,808,081	45,808,720	639	Loss
DEL00039944	chr5	45,816,647	45,816,916	269	Loss
DEL00039947	chr5	45,824,514	45,824,639	125	Loss
DEL00039948	chr5	45,833,102	45,834,201	1,099	Loss
DEL00039957	chr5	45,922,207	45,922,352	145	Loss
DEL00039970	chr5	46,207,061	46,207,118	57	Loss
DEL00039974	chr5	46,291,773	46,298,916	7,143	Loss
DEL00039977	chr5	46,358,220	46,358,302	82	Loss
DEL00039978	chr5	46,361,224	46,361,948	724	Loss
DEL00039979	chr5	46,365,203	46,367,429	2,226	Loss
DEL00039982	chr5	46,461,855	46,461,965	110	Loss
DEL00039993	chr5	46,740,569	46,740,822	253	Loss
DEL00039999	chr5	46,827,947	46,828,433	486	Loss
DUP00040001	chr5	46,856,904	46,857,073	169	Gain
DEL00040003	chr5	46,884,339	46,884,398	59	Loss
DEL00040004	chr5	46,901,336	46,901,872	536	Loss
DEL00040008	chr5	46,958,811	46,958,984	173	Loss
DEL00040018	chr5	47,051,855	47,052,257	402	Loss
DEL00040021	chr5	47,054,013	47,054,072	59	Loss
DEL00040027	chr5	47,197,608	47,197,665	57	Loss
DEL00040028	chr5	47,229,474	47,229,662	188	Loss

DEL00040043	chr5	47,675,028	47,675,191	163	Loss
DEL00040047	chr5	47,820,224	47,820,319	95	Loss
DEL00040074	chr5	48,474,414	48,480,764	6,350	Loss
DEL00040083	chr5	48,829,039	48,829,161	122	Loss
DEL00040090	chr5	48,882,203	48,882,384	181	Loss
DEL00040091	chr5	48,899,328	48,899,619	291	Loss
DEL00040097	chr5	48,984,415	48,984,481	66	Loss
DEL00040102	chr5	49,009,936	49,010,028	92	Loss
DEL00040103	chr5	49,013,542	49,013,606	64	Loss
DEL00040119	chr5	49,399,427	49,399,558	131	Loss
DEL00040122	chr5	49,474,713	49,476,551	1,838	Loss
DEL00040129	chr5	49,550,180	49,550,233	53	Loss
DUP00040130	chr5	49,561,182	49,567,068	5,886	Gain
DEL00040140	chr5	49,609,293	49,609,355	62	Loss
DEL00040145	chr5	49,738,787	49,738,876	89	Loss
DEL00040155	chr5	49,910,891	49,910,986	95	Loss
DEL00040163	chr5	50,073,389	50,073,859	470	Loss
DEL00040164	chr5	50,089,338	50,089,391	53	Loss
DEL00040165	chr5	50,104,729	50,104,826	97	Loss
DEL00040178	chr5	50,257,008	50,257,090	82	Loss
DEL00040184	chr5	50,292,759	50,293,065	306	Loss
DEL00040187	chr5	50,333,257	50,333,555	298	Loss
DEL00040189	chr5	50,395,103	50,395,460	357	Loss
DEL00040199	chr5	50,538,045	50,538,104	59	Loss
DEL00040208	chr5	50,758,957	50,759,472	515	Loss
DEL00040215	chr5	50,847,923	50,847,975	52	Loss
DEL00040217	chr5	50,910,874	50,910,925	51	Loss
DEL00040218	chr5	50,916,005	50,916,221	216	Loss
DEL00040234	chr5	51,341,950	51,342,505	555	Loss
DEL00040236	chr5	51,397,207	51,397,269	62	Loss
DEL00040240	chr5	51,502,750	51,502,821	71	Loss
DEL00040241	chr5	51,520,473	51,520,600	127	Loss
DEL00040244	chr5	51,531,791	51,532,174	383	Loss
DEL00040250	chr5	51,656,334	51,656,577	243	Loss
DEL00040253	chr5	51,704,465	51,704,535	70	Loss
DEL00040255	chr5	51,795,621	51,795,856	235	Loss
DEL00040257	chr5	51,819,278	51,819,368	90	Loss
DEL00040259	chr5	51,830,608	51,831,724	1,116	Loss
DEL00040260	chr5	51,863,496	51,863,550	54	Loss
DEL00040261	chr5	51,870,615	51,870,671	56	Loss
DEL00040271	chr5	51,916,093	51,916,157	64	Loss
DEL00040276	chr5	51,999,148	51,999,645	497	Loss
DEL00040279	chr5	52,047,344	52,047,850	506	Loss
DEL00040287	chr5	52,149,857	52,150,202	345	Loss
DEL00040297	chr5	52,254,884	52,255,399	515	Loss
DEL00040300	chr5	52,338,536	52,338,739	203	Loss
DEL00040305	chr5	52,536,480	52,536,620	140	Loss
DEL00040310	chr5	52,638,046	52,638,900	854	Loss
DEL00040315	chr5	52,739,223	52,739,714	491	Loss

DEL00040317	chr5	52,763,848	52,764,134	286	Loss
DEL00040325	chr5	52,941,660	52,941,836	176	Loss
DEL00040332	chr5	53,121,893	53,122,237	344	Loss
DEL00040356	chr5	53,426,668	53,426,963	295	Loss
DEL00040358	chr5	53,472,212	53,472,433	221	Loss
DEL00040360	chr5	53,533,406	53,534,391	985	Loss
DEL00040366	chr5	53,635,722	53,635,792	70	Loss
DEL00040369	chr5	53,685,923	53,686,024	101	Loss
DEL00040375	chr5	53,702,349	53,702,753	404	Loss
DEL00040382	chr5	53,809,771	53,810,302	531	Loss
DEL00040383	chr5	53,817,505	53,817,688	183	Loss
DEL00040384	chr5	53,856,786	53,856,866	80	Loss
DEL00040385	chr5	53,861,175	53,861,242	67	Loss
DEL00040386	chr5	53,877,706	53,879,551	1,845	Loss
DEL00040392	chr5	53,960,616	53,960,674	58	Loss
DEL00040394	chr5	53,963,040	53,963,371	331	Loss
DEL00040395	chr5	53,994,499	53,995,149	650	Loss
DEL00040396	chr5	54,009,012	54,009,149	137	Loss
DEL00040404	chr5	54,170,391	54,170,464	73	Loss
DEL00040405	chr5	54,170,677	54,171,268	591	Loss
DEL00040406	chr5	54,222,636	54,223,015	379	Loss
DEL00040409	chr5	54,242,724	54,242,785	61	Loss
DEL00040411	chr5	54,271,040	54,271,192	152	Loss
DEL00040417	chr5	54,394,684	54,394,769	85	Loss
DEL00040434	chr5	54,813,654	54,814,156	502	Loss
DEL00040442	chr5	54,978,472	54,978,532	60	Loss
DEL00040452	chr5	55,024,964	55,025,020	56	Loss
DEL00040475	chr5	55,358,241	55,358,299	58	Loss
DEL00040476	chr5	55,406,673	55,406,757	84	Loss
DUP00040477	chr5	55,466,670	55,466,768	98	Gain
DEL00040493	chr5	55,738,835	55,739,803	968	Loss
DEL00040494	chr5	55,741,741	55,762,047	20,306	Loss
DEL00040501	chr5	55,889,465	55,889,536	71	Loss
DEL00040509	chr5	55,967,809	55,968,477	668	Loss
DEL00040512	chr5	56,014,216	56,014,416	200	Loss
DEL00040516	chr5	56,077,525	56,077,704	179	Loss
DEL00040521	chr5	56,152,620	56,152,704	84	Loss
DEL00040523	chr5	56,175,262	56,175,515	253	Loss
DEL00040527	chr5	56,184,075	56,184,836	761	Loss
DEL00040548	chr5	56,329,007	56,329,402	395	Loss
DEL00040549	chr5	56,341,827	56,342,984	1,157	Loss
DEL00040553	chr5	56,390,271	56,390,443	172	Loss
DEL00040554	chr5	56,403,813	56,403,962	149	Loss
DEL00040557	chr5	56,556,710	56,557,186	476	Loss
DEL00040563	chr5	56,598,754	56,598,814	60	Loss
DEL00040573	chr5	56,712,011	56,712,629	618	Loss
DEL00040586	chr5	56,793,496	56,793,805	309	Loss
DEL00040589	chr5	56,795,681	56,795,861	180	Loss
DEL00040595	chr5	56,823,688	56,823,740	52	Loss

DEL00040599	chr5	56,838,062	56,838,304	242	Loss
DEL00040609	chr5	56,910,236	56,910,358	122	Loss
DEL00040642	chr5	57,233,920	57,234,051	131	Loss
DEL00040643	chr5	57,241,811	57,242,714	903	Loss
DEL00040651	chr5	57,336,389	57,336,517	128	Loss
DEL00040699	chr5	57,535,259	57,535,363	104	Loss
DEL00040700	chr5	57,564,600	57,565,013	413	Loss
DEL00040716	chr5	57,599,052	57,599,224	172	Loss
DEL00040719	chr5	57,605,631	57,606,087	456	Loss
DEL00040721	chr5	57,624,073	57,624,152	79	Loss
DEL00040727	chr5	57,673,557	57,676,826	3,269	Mixed
DEL00040741	chr5	57,727,481	57,727,558	77	Loss
DEL00040785	chr5	58,113,220	58,113,284	64	Loss
DEL00040791	chr5	58,150,107	58,150,316	209	Loss
DEL00040795	chr5	58,181,510	58,181,728	218	Loss
DEL00040800	chr5	58,300,682	58,300,848	166	Loss
DEL00040820	chr5	58,537,967	58,538,027	60	Loss
DEL00040822	chr5	58,577,093	58,577,928	835	Loss
DEL00040834	chr5	58,667,216	58,667,319	103	Loss
DEL00040841	chr5	58,693,085	58,693,763	678	Loss
DEL00040843	chr5	58,699,288	58,699,413	125	Loss
DEL00040876	chr5	59,056,429	59,056,481	52	Loss
DEL00040882	chr5	59,112,098	59,112,210	112	Loss
DUP00040887	chr5	59,192,659	59,193,117	458	Gain
DEL00040888	chr5	59,202,166	59,202,240	74	Loss
DUP00040890	chr5	59,238,244	59,238,454	210	Gain
DEL00040900	chr5	59,447,597	59,449,459	1,862	Loss
DEL00040945	chr6	186,761	189,431	2,670	Loss
DUP00040949	chr6	254,836	254,948	112	Gain
DEL00040950	chr6	271,342	272,417	1,075	Loss
DEL00040956	chr6	322,505	322,836	331	Loss
DEL00040957	chr6	324,871	324,944	73	Loss
DEL00040961	chr6	413,634	413,731	97	Loss
DEL00040970	chr6	483,472	483,614	142	Loss
DEL00040971	chr6	494,103	494,185	82	Loss
DEL00040979	chr6	578,367	578,900	533	Loss
DEL00040981	chr6	593,505	593,571	66	Loss
DEL00040986	chr6	665,012	666,190	1,178	Loss
DEL00040989	chr6	671,998	672,505	507	Loss
DEL00040993	chr6	752,279	752,338	59	Loss
DEL00040995	chr6	790,323	790,836	513	Loss
DEL00040996	chr6	791,128	791,228	100	Loss
DEL00041001	chr6	821,888	822,765	877	Loss
DEL00041015	chr6	1,006,210	1,007,083	873	Loss
DEL00041027	chr6	1,099,816	1,099,965	149	Loss
DEL00041028	chr6	1,118,527	1,118,636	109	Loss
DEL00041037	chr6	1,190,377	1,190,838	461	Loss
DEL00041045	chr6	1,283,994	1,284,219	225	Loss
DEL00041055	chr6	1,488,046	1,488,145	99	Loss

DEL00041056	chr6	1,606,584	1,606,647	63	Loss
DEL00041061	chr6	1,630,039	1,630,269	230	Loss
DEL00041063	chr6	1,635,551	1,635,947	396	Loss
DEL00041065	chr6	1,744,156	1,744,259	103	Loss
DEL00041066	chr6	1,746,761	1,746,826	65	Loss
DEL00041070	chr6	1,791,811	1,792,041	230	Loss
DEL00041078	chr6	1,839,869	1,840,320	451	Loss
DEL00041079	chr6	1,851,962	1,852,071	109	Loss
DEL00041080	chr6	1,852,453	1,852,997	544	Loss
DEL00041082	chr6	1,873,728	1,874,557	829	Loss
DEL00041087	chr6	1,910,368	1,910,813	445	Loss
DEL00041090	chr6	1,946,422	1,946,627	205	Loss
DEL00041095	chr6	2,013,276	2,013,585	309	Loss
DEL00041096	chr6	2,024,341	2,024,412	71	Loss
DEL00041098	chr6	2,055,150	2,055,202	52	Loss
DEL00041099	chr6	2,094,277	2,094,333	56	Loss
DEL00041105	chr6	2,136,777	2,136,845	68	Loss
DEL00041106	chr6	2,215,556	2,215,652	96	Loss
DEL00041109	chr6	2,238,100	2,238,232	132	Loss
DEL00041113	chr6	2,290,449	2,290,566	117	Loss
DEL00041117	chr6	2,309,604	2,310,134	530	Loss
DEL00041118	chr6	2,338,098	2,338,287	189	Loss
DEL00041125	chr6	2,427,437	2,427,997	560	Loss
DEL00041129	chr6	2,510,045	2,510,107	62	Loss
DEL00041144	chr6	2,739,101	2,739,250	149	Loss
DEL00041146	chr6	2,761,419	2,761,489	70	Loss
DEL00041150	chr6	2,783,551	2,783,619	68	Loss
DEL00041152	chr6	2,791,423	2,791,535	112	Loss
DEL00041168	chr6	3,008,483	3,008,643	160	Loss
DEL00041169	chr6	3,021,119	3,021,193	74	Loss
DEL00041172	chr6	3,087,116	3,087,171	55	Loss
DEL00041176	chr6	3,113,434	3,113,511	77	Loss
DEL00041177	chr6	3,141,894	3,142,003	109	Loss
DEL00041190	chr6	3,274,265	3,275,427	1,162	Loss
DEL00041199	chr6	3,349,402	3,349,852	450	Loss
DEL00041234	chr6	3,411,279	3,413,059	1,780	Loss
DEL00041237	chr6	3,440,457	3,441,237	780	Loss
DEL00041245	chr6	3,538,653	3,538,730	77	Loss
DEL00041250	chr6	3,573,904	3,574,017	113	Loss
DEL00041251	chr6	3,580,856	3,580,917	61	Loss
DEL00041263	chr6	3,783,029	3,783,344	315	Loss
DEL00041268	chr6	3,837,185	3,837,255	70	Loss
DEL00041269	chr6	3,844,482	3,845,394	912	Loss
DEL00041271	chr6	3,853,088	3,853,523	435	Loss
DEL00041291	chr6	4,046,743	4,048,525	1,782	Loss
DEL00041297	chr6	4,055,876	4,056,270	394	Loss
DEL00041304	chr6	4,090,397	4,090,752	355	Loss
DEL00041306	chr6	4,110,349	4,111,110	761	Loss
DUP00041313	chr6	4,164,980	4,183,056	18,076	Mixed

DEL00041318	chr6	4,189,446	4,189,948	502	Loss
DEL00041320	chr6	4,237,942	4,238,293	351	Loss
DEL00041322	chr6	4,239,114	4,239,267	153	Loss
DEL00041328	chr6	4,293,554	4,293,689	135	Loss
DEL00041337	chr6	4,364,973	4,367,366	2,393	Loss
DEL00041350	chr6	4,415,455	4,415,842	387	Loss
DEL00041354	chr6	4,441,424	4,441,525	101	Loss
DEL00041356	chr6	4,466,036	4,466,094	58	Loss
DEL00041365	chr6	4,592,206	4,592,297	91	Loss
DEL00041374	chr6	4,696,166	4,696,898	732	Loss
DEL00041380	chr6	4,710,824	4,711,176	352	Loss
DEL00041383	chr6	4,773,859	4,774,567	708	Loss
DEL00041384	chr6	4,789,594	4,789,660	66	Loss
DEL00041385	chr6	4,790,027	4,790,363	336	Loss
DEL00041386	chr6	4,791,763	4,791,826	63	Loss
DEL00041387	chr6	4,800,252	4,800,316	64	Loss
DEL00041395	chr6	4,965,536	4,965,805	269	Loss
DEL00041400	chr6	5,028,312	5,028,408	96	Loss
DEL00041401	chr6	5,035,367	5,035,535	168	Loss
DEL00041402	chr6	5,050,392	5,050,935	543	Loss
DEL00041403	chr6	5,057,926	5,057,984	58	Loss
DEL00041408	chr6	5,145,298	5,145,404	106	Loss
DEL00041422	chr6	5,263,460	5,263,512	52	Loss
DEL00041429	chr6	5,331,553	5,331,634	81	Loss
DEL00041434	chr6	5,372,157	5,373,549	1,392	Loss
DEL00041435	chr6	5,378,073	5,378,158	85	Loss
DEL00041437	chr6	5,385,458	5,385,523	65	Loss
DEL00041438	chr6	5,451,944	5,452,006	62	Loss
DUP00041442	chr6	5,506,305	5,517,309	11,004	Gain
DEL00041448	chr6	5,578,673	5,579,892	1,219	Loss
DEL00041464	chr6	5,829,920	5,830,338	418	Loss
DEL00041466	chr6	5,835,701	5,835,771	70	Loss
DEL00041467	chr6	5,858,887	5,864,020	5,133	Loss
DEL00041473	chr6	5,960,809	5,960,949	140	Loss
DEL00041484	chr6	6,098,825	6,098,922	97	Loss
DEL00041485	chr6	6,107,691	6,107,782	91	Loss
DEL00041488	chr6	6,139,833	6,140,041	208	Loss
DEL00041489	chr6	6,153,796	6,153,939	143	Loss
DEL00041492	chr6	6,213,792	6,214,051	259	Loss
DEL00041495	chr6	6,273,797	6,274,398	601	Loss
DEL00041498	chr6	6,320,386	6,320,534	148	Loss
DEL00041499	chr6	6,349,971	6,350,063	92	Loss
DEL00041502	chr6	6,380,794	6,381,717	923	Loss
DEL00041503	chr6	6,444,076	6,444,244	168	Loss
DEL00041506	chr6	6,483,676	6,484,257	581	Loss
DEL00041507	chr6	6,494,214	6,495,133	919	Loss
DEL00041517	chr6	6,682,401	6,682,533	132	Loss
DEL00041521	chr6	6,743,890	6,744,849	959	Loss
DEL00041527	chr6	6,828,055	6,828,564	509	Loss

DEL00041533	chr6	6,971,572	6,971,627	55	Loss
DEL00041534	chr6	7,014,711	7,014,823	112	Loss
DEL00041538	chr6	7,081,653	7,081,814	161	Loss
DEL00041539	chr6	7,093,000	7,093,081	81	Loss
DEL00041542	chr6	7,107,632	7,107,977	345	Loss
DEL00041547	chr6	7,167,310	7,167,474	164	Loss
DEL00041553	chr6	7,260,699	7,261,318	619	Loss
DEL00041572	chr6	7,665,980	7,666,133	153	Loss
DEL00041575	chr6	7,792,968	7,793,631	663	Loss
DEL00041580	chr6	7,927,161	7,927,622	461	Loss
DEL00041585	chr6	7,936,755	7,937,329	574	Loss
DEL00041593	chr6	7,984,062	7,984,262	200	Loss
DEL00041594	chr6	8,013,340	8,013,397	57	Loss
DEL00041600	chr6	8,214,316	8,214,837	521	Loss
DEL00041602	chr6	8,228,099	8,228,337	238	Loss
DEL00041607	chr6	8,313,081	8,313,186	105	Loss
DEL00041608	chr6	8,319,154	8,320,953	1,799	Loss
DEL00041609	chr6	8,361,389	8,362,352	963	Loss
DEL00041611	chr6	8,378,475	8,378,621	146	Loss
DEL00041619	chr6	8,602,504	8,603,162	658	Loss
DEL00041630	chr6	8,850,135	8,850,349	214	Loss
DEL00041631	chr6	8,864,785	8,864,882	97	Loss
DEL00041633	chr6	8,883,411	8,883,551	140	Loss
DEL00041634	chr6	8,887,145	8,887,306	161	Loss
DEL00041637	chr6	8,895,365	8,895,835	470	Loss
DEL00041639	chr6	9,063,036	9,063,570	534	Loss
DEL00041640	chr6	9,080,311	9,080,371	60	Loss
DEL00041650	chr6	9,314,687	9,314,879	192	Loss
DEL00041653	chr6	9,384,702	9,384,861	159	Loss
DEL00041658	chr6	9,692,536	9,693,374	838	Loss
DEL00041668	chr6	9,852,239	9,852,539	300	Loss
DEL00041673	chr6	9,892,527	9,896,092	3,565	Loss
DEL00041698	chr6	10,284,394	10,284,487	93	Loss
DEL00041700	chr6	10,303,003	10,303,092	89	Loss
DEL00041702	chr6	10,420,498	10,421,765	1,267	Loss
DEL00041710	chr6	10,553,025	10,553,107	82	Loss
DEL00041713	chr6	10,592,750	10,592,995	245	Loss
DEL00041714	chr6	10,600,601	10,600,697	96	Loss
DEL00041720	chr6	10,799,865	10,800,494	629	Loss
DEL00041744	chr6	11,001,746	11,002,245	499	Loss
DEL00041761	chr6	11,485,463	11,485,976	513	Loss
DEL00041771	chr6	11,667,469	11,667,537	68	Loss
DEL00041799	chr6	12,225,570	12,225,641	71	Loss
DEL00041806	chr6	12,314,173	12,314,297	124	Loss
DEL00041808	chr6	12,399,955	12,400,023	68	Loss
DEL00041816	chr6	12,554,487	12,555,470	983	Loss
DEL00041821	chr6	12,823,456	12,824,597	1,141	Loss
DEL00041832	chr6	12,962,376	12,962,484	108	Loss
DEL00041833	chr6	13,065,959	13,066,171	212	Loss

DEL00041837	chr6	13,088,746	13,089,138	392	Loss
DEL00041838	chr6	13,092,519	13,092,662	143	Loss
DEL00041842	chr6	13,184,252	13,184,566	314	Loss
DEL00041843	chr6	13,222,854	13,224,090	1,236	Loss
DEL00041846	chr6	13,452,454	13,453,911	1,457	Loss
DEL00041848	chr6	13,634,868	13,635,478	610	Loss
DEL00041849	chr6	13,639,558	13,640,035	477	Loss
DEL00041852	chr6	13,722,758	13,722,940	182	Loss
DEL00041870	chr6	14,009,258	14,009,453	195	Loss
DEL00041875	chr6	14,093,817	14,094,870	1,053	Loss
DEL00041885	chr6	14,199,641	14,200,130	489	Loss
DEL00041891	chr6	14,507,150	14,507,570	420	Loss
DEL00041897	chr6	14,678,459	14,679,554	1,095	Loss
DEL00041898	chr6	14,685,793	14,685,878	85	Loss
DEL00041899	chr6	14,705,497	14,706,570	1,073	Loss
DEL00041903	chr6	14,763,203	14,763,472	269	Loss
DEL00041906	chr6	14,935,822	14,935,873	51	Loss
DEL00041914	chr6	15,186,103	15,186,217	114	Loss
DEL00041917	chr6	15,196,544	15,197,159	615	Loss
DEL00041929	chr6	15,530,232	15,530,352	120	Loss
DEL00041932	chr6	15,681,537	15,681,935	398	Loss
DEL00041938	chr6	15,708,297	15,708,625	328	Loss
DEL00041941	chr6	15,775,797	15,775,859	62	Loss
DEL00041946	chr6	15,857,120	15,857,424	304	Loss
DEL00041949	chr6	15,859,762	15,860,238	476	Loss
DEL00041960	chr6	16,157,912	16,157,989	77	Loss
DEL00041963	chr6	16,326,261	16,326,350	89	Loss
DEL00041967	chr6	16,391,989	16,392,216	227	Loss
DEL00041973	chr6	16,700,928	16,701,261	333	Loss
DEL00041974	chr6	16,768,970	16,769,184	214	Loss
DEL00041975	chr6	17,013,978	17,014,589	611	Loss
DEL00041981	chr6	17,129,343	17,129,419	76	Loss
DEL00041982	chr6	17,361,678	17,361,750	72	Loss
DEL00041993	chr6	17,493,001	17,493,148	147	Loss
DEL00041994	chr6	17,494,605	17,501,741	7,136	Loss
DEL00042000	chr6	17,521,268	17,521,917	649	Loss
DEL00042001	chr6	17,570,611	17,570,686	75	Loss
DEL00042003	chr6	17,604,348	17,604,600	252	Loss
DEL00042005	chr6	17,716,049	17,716,707	658	Loss
DEL00042013	chr6	17,904,151	17,909,984	5,833	Loss
DEL00042018	chr6	17,973,080	17,973,519	439	Loss
DEL00042021	chr6	17,996,810	17,997,207	397	Loss
DEL00042024	chr6	18,068,536	18,068,675	139	Loss
DEL00042025	chr6	18,069,574	18,069,654	80	Loss
DEL00042026	chr6	18,148,385	18,155,333	6,948	Loss
DEL00042032	chr6	18,294,997	18,295,207	210	Loss
DEL00042036	chr6	18,331,496	18,331,571	75	Loss
DEL00042038	chr6	18,417,067	18,417,181	114	Loss
DEL00042040	chr6	18,459,448	18,459,584	136	Loss

DEL00042041	chr6	18,505,267	18,505,655	388	Loss
DEL00042043	chr6	18,630,619	18,634,175	3,556	Loss
DEL00042053	chr6	18,700,346	18,700,508	162	Loss
DEL00042067	chr6	18,932,935	18,933,061	126	Loss
DEL00042073	chr6	18,984,743	18,984,921	178	Loss
DEL00042077	chr6	19,239,506	19,239,727	221	Loss
DEL00042107	chr6	19,446,604	19,446,662	58	Loss
DEL00042117	chr6	19,565,774	19,565,993	219	Loss
DUP00042127	chr6	19,679,709	19,679,813	104	Gain
DUP00042132	chr6	19,766,851	19,778,170	11,319	Gain
DEL00042134	chr6	19,817,261	19,817,729	468	Loss
DEL00042136	chr6	19,889,290	19,889,719	429	Loss
DEL00042138	chr6	19,965,023	19,965,202	179	Loss
DEL00042139	chr6	20,008,423	20,008,919	496	Loss
DEL00042141	chr6	20,088,772	20,089,502	730	Loss
DEL00042144	chr6	20,216,179	20,216,284	105	Loss
DEL00042145	chr6	20,297,955	20,298,035	80	Loss
DEL00042146	chr6	20,365,541	20,365,641	100	Loss
DEL00042152	chr6	20,521,352	20,521,559	207	Loss
DEL00042154	chr6	20,627,476	20,627,540	64	Loss
DEL00042156	chr6	20,658,715	20,658,773	58	Loss
DEL00042157	chr6	20,676,829	20,676,991	162	Loss
DEL00042158	chr6	20,687,432	20,687,574	142	Loss
DEL00042163	chr6	20,720,075	20,720,370	295	Loss
DEL00042164	chr6	20,747,309	20,747,396	87	Loss
DEL00042171	chr6	21,071,345	21,071,568	223	Loss
DEL00042172	chr6	21,081,854	21,082,000	146	Loss
DEL00042174	chr6	21,088,782	21,088,848	66	Loss
DEL00042180	chr6	21,194,139	21,196,080	1,941	Loss
DEL00042188	chr6	21,332,377	21,332,501	124	Loss
DEL00042189	chr6	21,358,204	21,358,263	59	Loss
DEL00042190	chr6	21,369,341	21,369,841	500	Loss
DEL00042196	chr6	21,482,059	21,482,138	79	Loss
DEL00042200	chr6	21,641,052	21,641,109	57	Loss
DEL00042202	chr6	21,647,276	21,647,661	385	Loss
DEL00042214	chr6	22,135,779	22,136,003	224	Loss
DUP00042215	chr6	22,140,359	22,140,523	164	Gain
DEL00042236	chr6	22,726,065	22,726,381	316	Loss
DEL00042243	chr6	23,043,355	23,044,570	1,215	Loss
DEL00042250	chr6	23,147,574	23,147,925	351	Loss
DEL00042255	chr6	23,169,680	23,169,982	302	Loss
DEL00042256	chr6	23,195,547	23,195,605	58	Loss
DEL00042257	chr6	23,207,508	23,207,575	67	Loss
DEL00042261	chr6	23,254,092	23,254,830	738	Loss
DEL00042271	chr6	23,537,520	23,538,435	915	Loss
DEL00042277	chr6	23,543,518	23,543,579	61	Loss
DEL00042283	chr6	23,655,452	23,656,431	979	Loss
DEL00042290	chr6	23,902,012	23,902,745	733	Loss
DEL00042291	chr6	23,911,866	23,912,629	763	Loss

DEL00042297	chr6	24,056,701	24,056,754	53	Loss
DEL00042301	chr6	24,102,237	24,102,322	85	Loss
DEL00042304	chr6	24,112,771	24,112,881	110	Loss
DEL00042305	chr6	24,154,852	24,155,017	165	Loss
DEL00042312	chr6	24,224,302	24,224,674	372	Loss
DEL00042313	chr6	24,259,828	24,260,114	286	Loss
DEL00042316	chr6	24,381,655	24,381,730	75	Loss
DEL00042319	chr6	24,496,070	24,496,386	316	Loss
DEL00042325	chr6	24,653,850	24,657,215	3,365	Loss
DEL00042330	chr6	24,754,131	24,754,191	60	Loss
DEL00042333	chr6	24,808,184	24,808,357	173	Loss
DEL00042334	chr6	24,808,968	24,809,022	54	Loss
DEL00042344	chr6	24,975,935	24,976,006	71	Loss
DEL00042352	chr6	25,159,680	25,160,360	680	Loss
DEL00042353	chr6	25,182,910	25,184,487	1,577	Loss
DEL00042358	chr6	25,335,163	25,335,521	358	Loss
DEL00042360	chr6	25,364,301	25,364,569	268	Loss
DEL00042362	chr6	25,391,670	25,391,806	136	Loss
DEL00042366	chr6	25,528,462	25,528,574	112	Loss
DEL00042367	chr6	25,547,897	25,548,132	235	Loss
DEL00042370	chr6	25,615,362	25,615,678	316	Loss
DUP00042380	chr6	25,838,252	25,838,467	215	Gain
DEL00042400	chr6	26,302,506	26,303,065	559	Loss
DEL00042404	chr6	26,310,034	26,310,171	137	Loss
DEL00042408	chr6	26,439,628	26,439,756	128	Loss
DEL00042414	chr6	26,586,700	26,586,841	141	Loss
DEL00042416	chr6	26,588,937	26,589,263	326	Loss
DEL00042426	chr6	26,736,269	26,736,372	103	Loss
DEL00042427	chr6	26,736,961	26,737,510	549	Loss
DEL00042450	chr6	27,381,775	27,382,080	305	Loss
DEL00042464	chr6	27,585,760	27,585,935	175	Loss
DEL00042465	chr6	27,623,977	27,624,052	75	Loss
DEL00042467	chr6	27,675,458	27,675,680	222	Loss
DEL00042470	chr6	27,771,699	27,771,753	54	Loss
DEL00042471	chr6	27,791,509	27,791,937	428	Loss
DEL00042479	chr6	27,842,903	27,843,019	116	Loss
DEL00042486	chr6	28,078,875	28,078,926	51	Loss
DEL00042487	chr6	28,102,903	28,102,983	80	Loss
DEL00042490	chr6	28,114,261	28,114,463	202	Loss
DEL00042497	chr6	28,245,179	28,245,291	112	Loss
DEL00042498	chr6	28,250,510	28,250,983	473	Loss
DEL00042499	chr6	28,257,343	28,257,426	83	Loss
DEL00042500	chr6	28,273,352	28,273,436	84	Loss
DUP00042509	chr6	28,335,894	28,344,792	8,898	Gain
DEL00042515	chr6	28,470,311	28,470,589	278	Loss
DEL00042521	chr6	28,543,717	28,543,768	51	Loss
DEL00042528	chr6	28,816,697	28,816,749	52	Loss
DEL00042531	chr6	28,914,860	28,914,930	70	Loss
DEL00042533	chr6	28,937,374	28,937,551	177	Loss

DEL00042534	chr6	28,958,306	28,958,429	123	Loss
DEL00042536	chr6	28,962,160	28,962,212	52	Loss
DEL00042542	chr6	29,109,936	29,110,068	132	Loss
DEL00042545	chr6	29,255,146	29,255,491	345	Loss
DEL00042549	chr6	29,357,710	29,357,963	253	Loss
DEL00042550	chr6	29,383,376	29,383,439	63	Loss
DEL00042556	chr6	29,431,095	29,431,363	268	Loss
DUP00042559	chr6	29,539,445	29,539,554	109	Gain
DEL00042562	chr6	29,561,280	29,561,516	236	Loss
DEL00042564	chr6	29,641,314	29,641,395	81	Loss
DEL00042565	chr6	29,644,523	29,644,596	73	Loss
DEL00042575	chr6	29,797,517	29,797,601	84	Loss
DEL00042577	chr6	29,857,779	29,858,620	841	Loss
DEL00042579	chr6	29,943,161	29,943,644	483	Loss
DUP00042582	chr6	30,131,868	30,152,912	21,044	Gain
DEL00042585	chr6	30,283,136	30,283,236	100	Loss
DEL00042590	chr6	30,394,454	30,394,603	149	Loss
DEL00042591	chr6	30,414,807	30,415,713	906	Loss
DEL00042594	chr6	30,428,122	30,428,178	56	Loss
DEL00042601	chr6	30,484,234	30,484,433	199	Loss
DEL00042609	chr6	30,591,738	30,591,849	111	Loss
DEL00042610	chr6	30,644,149	30,644,302	153	Loss
DEL00042611	chr6	30,655,614	30,655,758	144	Loss
DEL00042620	chr6	30,815,802	30,816,243	441	Loss
DEL00042621	chr6	30,824,448	30,824,661	213	Loss
DEL00042627	chr6	30,916,413	30,916,488	75	Loss
DEL00042628	chr6	30,968,701	30,968,893	192	Loss
DEL00042643	chr6	31,133,181	31,133,617	436	Loss
DEL00042644	chr6	31,138,280	31,138,389	109	Loss
DEL00042646	chr6	31,235,920	31,246,904	10,984	Loss
DEL00042654	chr6	31,359,301	31,359,414	113	Loss
DEL00042659	chr6	31,555,346	31,555,402	56	Loss
DEL00042663	chr6	31,638,931	31,639,018	87	Loss
DEL00042672	chr6	31,826,715	31,826,871	156	Loss
DEL00042678	chr6	31,930,979	31,931,121	142	Loss
DEL00042690	chr6	32,103,368	32,103,940	572	Loss
DEL00042699	chr6	32,167,276	32,167,434	158	Loss
DEL00042709	chr6	32,393,470	32,393,655	185	Loss
DEL00042710	chr6	32,399,721	32,402,111	2,390	Loss
DEL00042712	chr6	32,443,891	32,443,980	89	Loss
DEL00042721	chr6	32,576,584	32,577,104	520	Loss
DEL00042726	chr6	32,627,074	32,627,231	157	Loss
DEL00042730	chr6	32,699,509	32,699,711	202	Loss
DEL00042731	chr6	32,719,737	32,719,866	129	Loss
DEL00042739	chr6	32,804,155	32,804,209	54	Loss
DEL00042743	chr6	32,877,802	32,878,750	948	Loss
DEL00042746	chr6	32,949,994	32,950,248	254	Loss
DEL00042748	chr6	32,983,572	32,983,681	109	Loss
DUP00042752	chr6	33,077,271	33,077,430	159	Gain

DEL00042764	chr6	33,152,049	33,152,187	138	Loss
DEL00042769	chr6	33,169,038	33,169,699	661	Loss
DEL00042777	chr6	33,229,388	33,229,507	119	Loss
DEL00042785	chr6	33,273,085	33,273,173	88	Loss
DEL00042803	chr6	33,749,743	33,749,942	199	Loss
DEL00042806	chr6	33,921,061	33,921,293	232	Loss
DEL00042807	chr6	33,928,966	33,929,466	500	Loss
DEL00042834	chr6	34,327,595	34,327,861	266	Loss
DEL00042837	chr6	34,339,677	34,339,943	266	Loss
DEL00042845	chr6	34,380,489	34,381,436	947	Loss
DEL00042876	chr6	34,675,812	34,676,967	1,155	Loss
DEL00042882	chr6	34,709,055	34,709,711	656	Loss
DEL00042883	chr6	34,738,870	34,739,299	429	Loss
DEL00042890	chr6	34,865,845	34,865,924	79	Loss
DEL00042896	chr6	34,901,261	34,901,360	99	Loss
DEL00042897	chr6	34,901,807	34,902,066	259	Loss
DEL00042904	chr6	35,047,633	35,048,404	771	Loss
DEL00042912	chr7	75,522	75,691	169	Loss
DEL00042915	chr7	76,905	77,187	282	Loss
DEL00042916	chr7	77,365	77,423	58	Loss
DEL00042922	chr7	184,009	184,160	151	Loss
DEL00042923	chr7	219,334	220,191	857	Loss
DEL00042939	chr7	529,662	530,564	902	Loss
DEL00042940	chr7	533,278	533,417	139	Loss
DEL00042945	chr7	551,406	551,540	134	Loss
DEL00042951	chr7	621,995	622,433	438	Loss
DUP00042954	chr7	646,764	662,000	15,236	Mixed
DEL00042959	chr7	768,844	769,460	616	Loss
DEL00042963	chr7	916,875	917,059	184	Loss
DEL00042964	chr7	943,446	943,866	420	Loss
DEL00042965	chr7	998,201	998,254	53	Loss
DEL00042966	chr7	1,001,462	1,001,522	60	Loss
DEL00042973	chr7	1,112,853	1,112,930	77	Loss
DEL00042975	chr7	1,180,849	1,181,862	1,013	Loss
DEL00042977	chr7	1,212,225	1,212,327	102	Loss
DEL00042978	chr7	1,212,386	1,212,540	154	Loss
DEL00042979	chr7	1,235,014	1,235,191	177	Loss
DEL00042984	chr7	1,442,159	1,442,412	253	Loss
DEL00042985	chr7	1,453,900	1,453,960	60	Loss
DEL00042989	chr7	1,509,283	1,509,527	244	Loss
DEL00042990	chr7	1,523,981	1,524,079	98	Loss
DUP00042991	chr7	1,534,191	1,534,280	89	Gain
DEL00042992	chr7	1,540,884	1,545,195	4,311	Loss
DEL00043007	chr7	1,698,432	1,698,519	87	Loss
DEL00043012	chr7	1,726,427	1,726,495	68	Loss
DEL00043017	chr7	1,751,996	1,752,102	106	Loss
DEL00043021	chr7	1,847,866	1,848,012	146	Loss
DEL00043033	chr7	2,009,144	2,009,216	72	Loss
DEL00043038	chr7	2,078,784	2,078,884	100	Loss

DEL00043039	chr7	2,122,646	2,122,930	284	Loss
DEL00043041	chr7	2,131,936	2,132,521	585	Loss
DEL00043043	chr7	2,147,636	2,147,689	53	Loss
DEL00043044	chr7	2,159,287	2,159,675	388	Loss
DEL00043045	chr7	2,180,367	2,180,449	82	Loss
DEL00043046	chr7	2,187,934	2,188,016	82	Loss
DEL00043049	chr7	2,214,463	2,214,522	59	Loss
DEL00043052	chr7	2,301,178	2,301,250	72	Loss
DEL00043054	chr7	2,326,365	2,326,539	174	Loss
DEL00043056	chr7	2,378,326	2,378,418	92	Loss
DEL00043058	chr7	2,445,874	2,445,992	118	Loss
DEL00043059	chr7	2,449,117	2,449,172	55	Loss
DEL00043063	chr7	2,518,994	2,519,185	191	Loss
DEL00043064	chr7	2,520,052	2,520,165	113	Loss
DEL00043068	chr7	2,551,839	2,551,927	88	Loss
DEL00043074	chr7	2,617,122	2,617,247	125	Loss
DEL00043075	chr7	2,666,544	2,667,141	597	Loss
DEL00043076	chr7	2,675,119	2,675,417	298	Loss
DEL00043078	chr7	2,725,064	2,725,250	186	Loss
DEL00043084	chr7	2,805,575	2,805,637	62	Loss
DEL00043087	chr7	2,884,931	2,885,163	232	Loss
DEL00043088	chr7	2,906,003	2,906,091	88	Loss
DEL00043093	chr7	3,077,350	3,078,268	918	Loss
DEL00043099	chr7	3,134,362	3,134,424	62	Loss
DEL00043101	chr7	3,245,601	3,246,070	469	Loss
DEL00043111	chr7	3,457,996	3,458,267	271	Loss
DEL00043123	chr7	3,743,359	3,743,922	563	Loss
DEL00043129	chr7	3,897,600	3,898,020	420	Loss
DEL00043133	chr7	3,969,791	3,969,852	61	Loss
DEL00043134	chr7	4,056,281	4,056,378	97	Loss
DEL00043139	chr7	4,127,261	4,127,898	637	Loss
DEL00043143	chr7	4,146,275	4,146,523	248	Loss
DEL00043145	chr7	4,167,554	4,167,617	63	Loss
DEL00043146	chr7	4,206,065	4,206,828	763	Loss
DEL00043151	chr7	4,309,091	4,309,163	72	Loss
DEL00043156	chr7	4,472,663	4,472,846	183	Loss
DEL00043157	chr7	4,498,038	4,498,151	113	Loss
DEL00043158	chr7	4,504,461	4,504,596	135	Loss
DEL00043159	chr7	4,506,315	4,506,454	139	Loss
DEL00043160	chr7	4,506,831	4,506,996	165	Loss
DEL00043161	chr7	4,510,912	4,511,025	113	Loss
DEL00043162	chr7	4,522,416	4,523,393	977	Loss
DUP00043164	chr7	4,570,692	4,593,919	23,227	Gain
DEL00043167	chr7	4,661,862	4,663,264	1,402	Loss
DEL00043174	chr7	4,790,348	4,790,420	72	Loss
DEL00043178	chr7	4,926,431	4,926,526	95	Loss
DEL00043188	chr7	5,153,521	5,153,644	123	Loss
DEL00043191	chr7	5,209,995	5,210,051	56	Loss
DEL00043195	chr7	5,262,133	5,262,827	694	Loss

DEL00043196	chr7	5,270,342	5,270,395	53	Loss
DEL00043197	chr7	5,287,786	5,287,980	194	Loss
DEL00043199	chr7	5,307,677	5,308,088	411	Loss
DEL00043210	chr7	5,569,145	5,569,226	81	Loss
DEL00043218	chr7	5,670,307	5,670,671	364	Loss
DEL00043229	chr7	5,806,075	5,806,267	192	Loss
DEL00043236	chr7	5,852,590	5,852,688	98	Loss
DEL00043243	chr7	5,861,442	5,861,564	122	Loss
DEL00043251	chr7	5,951,924	5,952,029	105	Loss
DEL00043252	chr7	5,978,676	5,978,738	62	Loss
DEL00043256	chr7	6,209,587	6,209,645	58	Loss
DEL00043263	chr7	6,399,994	6,400,270	276	Loss
DEL00043272	chr7	6,664,309	6,664,383	74	Loss
DEL00043275	chr7	6,779,572	6,780,532	960	Loss
DEL00043282	chr7	6,974,303	6,981,189	6,886	Loss
DEL00043306	chr7	7,688,679	7,689,394	715	Loss
DEL00043314	chr7	7,928,036	7,928,092	56	Loss
DEL00043328	chr7	8,305,623	8,305,687	64	Loss
DEL00043332	chr7	8,421,848	8,422,200	352	Loss
DEL00043341	chr7	8,561,095	8,561,878	783	Loss
DEL00043342	chr7	8,563,314	8,563,414	100	Loss
DEL00043346	chr7	8,600,136	8,600,493	357	Loss
DEL00043351	chr7	8,617,489	8,617,547	58	Loss
DUP00043352	chr7	8,633,998	8,634,095	97	Gain
DEL00043355	chr7	8,707,742	8,711,710	3,968	Loss
DEL00043359	chr7	8,738,410	8,738,962	552	Loss
DEL00043364	chr7	8,796,594	8,796,664	70	Loss
DEL00043365	chr7	8,811,512	8,811,621	109	Loss
DEL00043367	chr7	8,869,237	8,869,299	62	Loss
DEL00043383	chr7	9,144,078	9,144,209	131	Loss
DUP00043387	chr7	9,195,329	9,200,172	4,843	Gain
DEL00043392	chr7	9,290,036	9,290,147	111	Loss
DEL00043411	chr7	9,478,600	9,479,257	657	Loss
DEL00043413	chr7	9,491,437	9,491,570	133	Loss
DEL00043415	chr7	9,534,986	9,535,491	505	Loss
DEL00043416	chr7	9,566,394	9,566,710	316	Loss
DEL00043421	chr7	9,575,630	9,575,940	310	Loss
DEL00043422	chr7	9,585,867	9,585,999	132	Loss
DEL00043427	chr7	9,606,480	9,606,534	54	Loss
DEL00043428	chr7	9,623,597	9,623,655	58	Loss
DEL00043433	chr7	9,673,246	9,673,680	434	Loss
DEL00043438	chr7	9,721,121	9,721,172	51	Loss
DEL00043440	chr7	9,756,342	9,756,515	173	Loss
DEL00043450	chr7	9,846,914	9,847,669	755	Loss
DEL00043451	chr7	9,883,165	9,883,237	72	Loss
DEL00043452	chr7	9,908,596	9,908,683	87	Loss
DEL00043460	chr7	10,129,625	10,129,764	139	Loss
DEL00043464	chr7	10,201,245	10,201,484	239	Loss
DEL00043468	chr7	10,279,079	10,279,160	81	Loss

DEL00043469	chr7	10,283,124	10,283,218	94	Loss
DEL00043474	chr7	10,365,489	10,366,017	528	Loss
DEL00043497	chr7	10,706,193	10,706,350	157	Loss
DEL00043500	chr7	10,723,546	10,723,607	61	Loss
DEL00043501	chr7	10,725,416	10,725,495	79	Loss
DEL00043502	chr7	10,742,540	10,742,648	108	Loss
DEL00043504	chr7	10,782,065	10,782,556	491	Loss
DUP00043516	chr7	10,826,367	10,826,773	406	Gain
DEL00043528	chr7	10,901,551	10,901,683	132	Loss
DEL00043530	chr7	10,936,327	10,936,502	175	Loss
DEL00043534	chr7	11,037,039	11,037,093	54	Loss
DUP00043539	chr7	11,248,096	11,284,995	36,899	Gain
DEL00043541	chr7	11,290,818	11,290,914	96	Loss
DEL00043543	chr7	11,376,456	11,376,545	89	Loss
DEL00043544	chr7	11,380,124	11,380,187	63	Loss
DEL00043546	chr7	11,398,219	11,398,360	141	Loss
DEL00043550	chr7	11,495,800	11,497,517	1,717	Loss
DEL00043555	chr7	11,592,390	11,592,600	210	Loss
DEL00043562	chr7	11,678,613	11,678,733	120	Loss
DEL00043564	chr7	11,689,324	11,689,393	69	Loss
DEL00043570	chr7	11,756,460	11,756,532	72	Loss
DEL00043577	chr7	11,903,573	11,903,715	142	Loss
DEL00043579	chr7	11,955,999	11,956,909	910	Loss
DEL00043584	chr7	12,009,759	12,009,815	56	Loss
DEL00043585	chr7	12,031,575	12,031,645	70	Loss
DEL00043591	chr7	12,197,017	12,198,796	1,779	Loss
DEL00043594	chr7	12,315,865	12,315,954	89	Loss
DEL00043596	chr7	12,400,588	12,401,165	577	Loss
DEL00043599	chr7	12,533,455	12,533,658	203	Loss
DEL00043603	chr7	12,579,845	12,580,187	342	Loss
DEL00043605	chr7	12,597,125	12,597,188	63	Loss
DUP00043608	chr7	12,643,081	12,656,495	13,414	Gain
DEL00043622	chr7	12,813,107	12,816,032	2,925	Loss
DEL00043624	chr7	12,879,160	12,879,574	414	Loss
DEL00043626	chr7	12,944,928	12,945,067	139	Loss
DEL00043628	chr7	12,964,887	12,964,969	82	Loss
DEL00043631	chr7	13,017,844	13,018,186	342	Loss
DEL00043634	chr7	13,059,640	13,060,801	1,161	Loss
DEL00043637	chr7	13,088,823	13,088,949	126	Loss
DEL00043645	chr7	13,237,521	13,238,138	617	Loss
DEL00043646	chr7	13,253,353	13,253,509	156	Loss
DEL00043652	chr7	13,454,747	13,454,908	161	Loss
DEL00043663	chr7	13,613,341	13,613,549	208	Loss
DEL00043672	chr7	13,730,568	13,730,906	338	Loss
DEL00043673	chr7	13,808,753	13,808,853	100	Loss
DEL00043674	chr7	13,817,492	13,817,577	85	Loss
DEL00043681	chr7	13,896,354	13,897,012	658	Loss
DEL00043686	chr7	13,952,679	13,952,881	202	Loss
DEL00043689	chr7	13,994,085	13,994,566	481	Loss

DEL00043696	chr7	14,138,863	14,138,969	106	Loss
DEL00043697	chr7	14,155,100	14,155,900	800	Loss
DEL00043699	chr7	14,181,654	14,182,345	691	Loss
DEL00043702	chr7	14,217,893	14,218,181	288	Loss
DEL00043705	chr7	14,273,008	14,273,061	53	Loss
DEL00043706	chr7	14,307,578	14,308,200	622	Loss
DEL00043708	chr7	14,323,901	14,325,280	1,379	Loss
DEL00043710	chr7	14,367,272	14,367,648	376	Loss
DEL00043714	chr7	14,401,506	14,416,354	14,848	Loss
DEL00043717	chr7	14,459,326	14,459,710	384	Loss
DEL00043718	chr7	14,469,528	14,469,880	352	Loss
DEL00043723	chr7	14,531,257	14,531,488	231	Loss
DEL00043727	chr7	14,597,165	14,597,299	134	Loss
DEL00043737	chr7	14,669,885	14,670,155	270	Loss
DEL00043742	chr7	14,912,892	14,912,954	62	Loss
DEL00043751	chr7	15,055,386	15,055,526	140	Loss
DEL00043753	chr7	15,060,896	15,061,217	321	Loss
DEL00043756	chr7	15,114,630	15,115,357	727	Loss
DEL00043762	chr7	15,218,781	15,218,839	58	Loss
DEL00043763	chr7	15,260,891	15,261,717	826	Loss
DEL00043764	chr7	15,264,158	15,264,914	756	Loss
DEL00043767	chr7	15,338,244	15,338,295	51	Loss
DEL00043774	chr7	15,442,573	15,442,654	81	Loss
DUP00043778	chr7	15,495,068	15,495,218	150	Gain
DEL00043779	chr7	15,497,413	15,498,057	644	Loss
DEL00043782	chr7	15,537,323	15,537,604	281	Loss
DEL00043790	chr7	15,770,806	15,770,984	178	Loss
DEL00043799	chr7	16,112,930	16,113,182	252	Loss
DEL00043804	chr7	16,218,257	16,218,474	217	Loss
DEL00043805	chr7	16,267,425	16,267,650	225	Loss
DEL00043806	chr7	16,309,449	16,309,567	118	Loss
DEL00043810	chr7	16,452,527	16,453,019	492	Loss
DEL00043813	chr7	16,513,599	16,513,670	71	Loss
DEL00043816	chr7	16,649,361	16,649,964	603	Loss
DEL00043821	chr7	16,662,770	16,663,258	488	Loss
DEL00043833	chr7	16,770,881	16,772,295	1,414	Loss
DEL00043838	chr7	16,812,941	16,813,246	305	Loss
DEL00043850	chr7	17,044,178	17,044,355	177	Loss
DEL00043853	chr7	17,063,507	17,063,681	174	Loss
DEL00043854	chr7	17,069,545	17,069,605	60	Loss
DEL00043855	chr7	17,071,191	17,071,245	54	Loss
DEL00043862	chr7	17,305,700	17,307,628	1,928	Loss
DEL00043863	chr7	17,336,910	17,337,772	862	Loss
DEL00043871	chr7	17,462,561	17,463,252	691	Loss
DEL00043872	chr7	17,469,736	17,469,815	79	Loss
DEL00043878	chr7	17,530,222	17,530,374	152	Loss
DEL00043879	chr7	17,556,702	17,556,771	69	Loss
DEL00043885	chr7	17,731,797	17,732,382	585	Loss
DEL00043889	chr7	17,777,734	17,777,796	62	Loss

DEL00043895	chr7	17,846,644	17,848,426	1,782	Loss
DEL00043899	chr7	17,913,538	17,913,641	103	Loss
DEL00043902	chr7	17,941,168	17,941,609	441	Loss
DUP00043903	chr7	17,943,737	17,943,813	76	Gain
DEL00043904	chr7	17,957,820	17,958,444	624	Loss
DEL00043907	chr7	18,039,723	18,039,785	62	Loss
DEL00043911	chr7	18,080,910	18,081,249	339	Loss
DEL00043916	chr7	18,157,664	18,157,904	240	Loss
DEL00043919	chr7	18,194,811	18,195,353	542	Loss
DEL00043920	chr7	18,223,478	18,226,313	2,835	Loss
DEL00043922	chr7	18,258,317	18,258,885	568	Loss
DEL00043924	chr7	18,302,427	18,303,174	747	Loss
DEL00043928	chr7	18,363,023	18,363,106	83	Loss
DEL00043932	chr7	18,411,798	18,411,908	110	Loss
DEL00043933	chr7	18,417,562	18,417,694	132	Loss
DEL00043942	chr7	18,765,731	18,767,149	1,418	Loss
DEL00043948	chr7	18,803,378	18,803,832	454	Loss
DEL00043950	chr7	18,817,078	18,817,133	55	Loss
DEL00043952	chr7	18,827,196	18,827,305	109	Loss
DEL00043953	chr7	18,828,714	18,828,822	108	Loss
DEL00043960	chr7	18,975,399	18,975,869	470	Loss
DEL00043962	chr7	18,999,485	18,999,658	173	Loss
DEL00043966	chr7	19,039,150	19,039,346	196	Loss
DEL00043977	chr7	19,211,742	19,211,998	256	Loss
DEL00043978	chr7	19,234,962	19,235,086	124	Loss
DEL00043979	chr7	19,243,667	19,243,723	56	Loss
DEL00043984	chr7	19,347,223	19,348,458	1,235	Loss
DEL00043993	chr7	19,590,300	19,590,404	104	Loss
DEL00044000	chr7	19,685,702	19,685,861	159	Loss
DEL00044001	chr7	19,692,956	19,693,469	513	Loss
DEL00044003	chr7	19,697,284	19,697,336	52	Loss
DEL00044012	chr7	19,837,189	19,838,441	1,252	Loss
DUP00044017	chr7	19,965,679	19,980,256	14,577	Gain
DEL00044021	chr7	20,086,326	20,086,389	63	Loss
DEL00044029	chr7	20,164,106	20,164,184	78	Loss
DEL00044046	chr7	20,525,911	20,525,988	77	Loss
DUP00044055	chr7	20,607,620	20,620,558	12,938	Gain
DEL00044059	chr7	20,644,164	20,644,233	69	Loss
DEL00044065	chr7	20,867,029	20,867,630	601	Loss
DEL00044066	chr7	20,877,534	20,877,589	55	Loss
DEL00044067	chr7	20,914,642	20,914,814	172	Loss
DEL00044071	chr7	21,122,730	21,123,275	545	Loss
DEL00044087	chr7	21,397,499	21,397,615	116	Loss
DEL00044106	chr7	21,462,195	21,465,663	3,468	Loss
DEL00044125	chr7	21,758,147	21,758,233	86	Loss
DEL00044130	chr7	21,862,031	21,862,188	157	Loss
DEL00044131	chr7	21,935,397	21,935,452	55	Loss
DEL00044134	chr7	22,024,018	22,024,095	77	Loss
DEL00044136	chr7	22,030,836	22,030,952	116	Loss

DEL00044138	chr7	22,049,675	22,049,837	162	Loss
DEL00044142	chr7	22,227,829	22,227,915	86	Loss
DEL00044146	chr7	22,315,687	22,315,782	95	Loss
DEL00044149	chr7	22,336,251	22,336,693	442	Loss
DEL00044155	chr7	22,391,197	22,391,348	151	Loss
DEL00044166	chr7	22,530,563	22,530,739	176	Loss
DEL00044169	chr7	22,556,894	22,557,156	262	Loss
DEL00044174	chr7	22,733,579	22,733,665	86	Loss
DEL00044180	chr7	22,828,938	22,829,551	613	Loss
DEL00044184	chr7	23,097,531	23,097,676	145	Loss
DEL00044187	chr7	23,100,812	23,100,879	67	Loss
DEL00044190	chr7	23,113,341	23,113,462	121	Loss
DEL00044195	chr7	23,194,395	23,194,456	61	Loss
DEL00044205	chr7	23,323,062	23,326,614	3,552	Loss
DEL00044206	chr7	23,391,725	23,391,850	125	Loss
DEL00044210	chr7	23,468,389	23,468,467	78	Loss
DEL00044214	chr7	23,555,981	23,556,545	564	Loss
DEL00044220	chr7	23,670,046	23,672,033	1,987	Loss
DEL00044223	chr7	23,687,357	23,687,555	198	Loss
DEL00044224	chr7	23,713,597	23,713,680	83	Loss
DEL00044226	chr7	23,744,998	23,745,531	533	Loss
DEL00044231	chr7	23,971,362	23,971,423	61	Loss
DEL00044234	chr7	24,062,093	24,062,257	164	Loss
DEL00044240	chr7	24,108,034	24,108,574	540	Loss
DUP00044242	chr7	24,109,092	24,123,253	14,161	Gain
DEL00044248	chr7	24,136,441	24,137,163	722	Loss
DEL00044251	chr7	24,159,534	24,159,838	304	Loss
DEL00044255	chr7	24,188,477	24,188,534	57	Loss
DEL00044259	chr7	24,222,267	24,222,696	429	Loss
DEL00044265	chr7	24,256,282	24,256,530	248	Loss
DEL00044268	chr7	24,277,181	24,277,247	66	Loss
DEL00044269	chr7	24,283,400	24,283,679	279	Loss
DEL00044278	chr7	24,416,523	24,417,877	1,354	Loss
DEL00044283	chr7	24,438,196	24,438,270	74	Loss
DEL00044291	chr7	24,795,597	24,795,781	184	Loss
DEL00044295	chr7	24,821,318	24,821,797	479	Loss
DEL00044297	chr7	24,830,223	24,830,285	62	Loss
DEL00044298	chr7	24,830,313	24,830,440	127	Loss
DEL00044301	chr7	24,851,144	24,851,228	84	Loss
DEL00044302	chr7	24,871,287	24,871,664	377	Loss
DEL00044315	chr7	25,087,072	25,087,222	150	Loss
DEL00044316	chr7	25,207,706	25,207,799	93	Loss
DEL00044325	chr7	25,340,950	25,341,119	169	Loss
DEL00044329	chr7	25,434,320	25,434,818	498	Loss
DEL00044339	chr7	25,712,670	25,712,736	66	Loss
DEL00044341	chr7	25,735,900	25,735,968	68	Loss
DEL00044350	chr7	25,920,585	25,920,715	130	Loss
DEL00044356	chr7	26,146,675	26,150,069	3,394	Loss
DEL00044358	chr7	26,180,467	26,180,806	339	Loss

DEL00044359	chr7	26,184,405	26,185,948	1,543	Loss
DEL00044364	chr7	26,443,696	26,444,075	379	Loss
DEL00044371	chr7	26,605,066	26,605,144	78	Loss
DEL00044383	chr7	26,811,366	26,811,563	197	Loss
DEL00044391	chr7	26,921,236	26,921,296	60	Loss
DEL00044392	chr7	26,994,720	26,994,795	75	Loss
DEL00044394	chr7	27,139,038	27,139,096	58	Loss
DEL00044396	chr7	27,233,104	27,233,285	181	Loss
DEL00044400	chr7	27,273,970	27,274,029	59	Loss
DEL00044405	chr7	27,410,678	27,410,862	184	Loss
DEL00044421	chr7	27,481,894	27,483,204	1,310	Loss
DEL00044430	chr7	27,644,201	27,644,263	62	Loss
DEL00044436	chr7	27,852,182	27,852,272	90	Loss
DEL00044448	chr7	28,036,542	28,038,956	2,414	Loss
DEL00044454	chr7	28,275,533	28,275,674	141	Loss
DEL00044455	chr7	28,358,202	28,358,866	664	Loss
DEL00044458	chr7	28,433,774	28,433,906	132	Loss
DEL00044460	chr7	28,463,600	28,463,788	188	Loss
DEL00044462	chr7	28,476,739	28,476,936	197	Loss
DEL00044465	chr7	28,563,988	28,564,306	318	Loss
DEL00044466	chr7	28,612,448	28,614,375	1,927	Loss
DEL00044467	chr7	28,623,500	28,624,486	986	Loss
DEL00044468	chr7	28,671,112	28,671,168	56	Loss
DEL00044469	chr7	28,680,886	28,681,399	513	Loss
DEL00044470	chr7	28,685,951	28,686,440	489	Loss
DEL00044472	chr7	28,693,061	28,693,182	121	Loss
DEL00044479	chr7	28,782,837	28,783,364	527	Loss
DEL00044482	chr7	28,818,099	28,818,157	58	Loss
DEL00044485	chr7	28,831,873	28,834,284	2,411	Loss
DEL00044486	chr7	28,839,208	28,840,051	843	Loss
DEL00044490	chr7	28,853,916	28,854,339	423	Loss
DEL00044491	chr7	28,860,848	28,861,105	257	Loss
DEL00044497	chr7	28,914,377	28,914,626	249	Loss
DEL00044504	chr7	28,981,760	28,982,498	738	Loss
DEL00044508	chr7	29,000,095	29,003,059	2,964	Loss
DEL00044511	chr7	29,088,954	29,089,407	453	Loss
DEL00044512	chr7	29,089,595	29,089,661	66	Loss
DEL00044515	chr7	29,128,593	29,129,088	495	Loss
DEL00044516	chr7	29,181,297	29,181,386	89	Loss
DEL00044517	chr7	29,184,724	29,187,075	2,351	Loss
DEL00044532	chr7	29,399,191	29,399,871	680	Loss
DEL00044534	chr7	29,416,196	29,416,759	563	Loss
DEL00044541	chr7	29,725,142	29,725,243	101	Loss
DEL00044550	chr7	29,862,425	29,862,816	391	Loss
DEL00044553	chr7	29,946,680	29,947,465	785	Loss
DEL00044557	chr7	30,074,889	30,075,683	794	Loss
DEL00044560	chr7	30,128,530	30,128,704	174	Loss
DEL00044561	chr7	30,143,006	30,143,070	64	Loss
DEL00044568	chr7	30,230,592	30,231,178	586	Loss

DEL00044577	chr7	30,475,010	30,475,099	89	Loss
DEL00044583	chr7	30,625,954	30,626,105	151	Loss
DEL00044585	chr7	30,713,913	30,714,100	187	Loss
DEL00044593	chr7	30,921,332	30,921,392	60	Loss
DEL00044594	chr7	30,952,864	30,953,001	137	Loss
DEL00044597	chr7	31,001,935	31,002,019	84	Loss
DEL00044603	chr7	31,084,892	31,085,189	297	Loss
DEL00044608	chr7	31,118,613	31,118,693	80	Loss
DEL00044611	chr7	31,164,210	31,164,342	132	Loss
DEL00044618	chr7	31,309,981	31,310,164	183	Loss
DEL00044619	chr7	31,310,323	31,310,419	96	Loss
DEL00044620	chr7	31,403,586	31,404,641	1,055	Loss
DEL00044623	chr7	31,449,661	31,449,814	153	Loss
DEL00044626	chr7	31,532,258	31,532,588	330	Loss
DEL00044634	chr7	31,657,560	31,657,624	64	Loss
DEL00044636	chr7	31,693,737	31,693,994	257	Loss
DEL00044640	chr7	31,774,131	31,774,505	374	Loss
DUP00044645	chr7	32,015,515	32,050,581	35,066	Gain
DEL00044647	chr7	32,056,048	32,056,101	53	Loss
DEL00044651	chr7	32,166,717	32,166,883	166	Loss
DEL00044654	chr7	32,230,517	32,231,176	659	Loss
DEL00044674	chr7	32,679,022	32,680,235	1,213	Loss
DEL00044684	chr7	33,012,819	33,012,986	167	Loss
DEL00044692	chr7	33,122,337	33,122,416	79	Loss
DEL00044693	chr7	33,134,244	33,134,309	65	Loss
DEL00044696	chr7	33,159,015	33,159,107	92	Loss
DEL00044704	chr7	33,289,796	33,289,931	135	Loss
DEL00044710	chr7	33,519,021	33,519,249	228	Loss
DUP00044711	chr7	33,521,504	33,521,859	355	Gain
DEL00044716	chr7	33,582,649	33,582,824	175	Loss
DEL00044720	chr7	33,601,885	33,602,040	155	Loss
DEL00044722	chr7	33,625,007	33,625,257	250	Loss
DEL00044730	chr7	33,734,505	33,734,576	71	Loss
DEL00044739	chr7	33,968,240	33,968,650	410	Loss
DEL00044742	chr7	34,023,058	34,023,127	69	Loss
DEL00044744	chr7	34,085,525	34,085,721	196	Loss
DEL00044748	chr7	34,129,023	34,129,133	110	Loss
DEL00044757	chr7	34,260,341	34,260,437	96	Loss
DEL00044769	chr7	34,370,661	34,370,789	128	Loss
DEL00044774	chr7	34,428,695	34,428,750	55	Loss
DEL00044780	chr7	34,462,671	34,463,388	717	Loss
DEL00044783	chr7	34,468,566	34,468,839	273	Loss
DEL00044794	chr7	34,569,333	34,569,473	140	Loss
DEL00044799	chr7	34,594,931	34,595,058	127	Loss
DEL00044805	chr7	34,679,610	34,679,698	88	Loss
DEL00044808	chr7	34,708,266	34,709,830	1,564	Loss
DEL00044817	chr7	34,717,818	34,717,920	102	Loss
DEL00044818	chr7	34,718,526	34,718,625	99	Loss
DEL00044819	chr7	34,723,492	34,723,560	68	Loss

DUP00044820	chr7	34,724,564	34,724,720	156	Gain
DEL00044822	chr7	34,748,607	34,748,709	102	Loss
DUP00044838	chr7	34,818,537	34,819,396	859	Gain
DEL00044854	chr7	35,098,770	35,098,845	75	Loss
DUP00044885	chr7	35,320,574	35,320,739	165	Gain
DEL00044896	chr7	35,451,544	35,451,978	434	Loss
DEL00044902	chr7	35,553,389	35,553,531	142	Loss
DEL00044908	chr7	35,665,194	35,665,253	59	Loss
DEL00044912	chr7	35,744,126	35,744,222	96	Loss
DEL00044913	chr7	35,750,965	35,751,063	98	Loss
DEL00044918	chr7	35,859,255	35,859,748	493	Loss
DEL00044931	chr7	36,246,801	36,247,313	512	Loss
DEL00044935	chr7	36,248,670	36,249,578	908	Loss
DEL00044957	chr8	17,832	18,676	844	Loss
DEL00044973	chr8	585,505	588,147	2,642	Loss
DEL00044975	chr8	670,560	670,622	62	Loss
DEL00044980	chr8	757,823	759,225	1,402	Loss
DEL00044983	chr8	915,567	915,621	54	Loss
DEL00044989	chr8	1,005,748	1,006,315	567	Loss
DEL00044993	chr8	1,047,738	1,048,094	356	Loss
DEL00044995	chr8	1,070,331	1,070,490	159	Loss
DEL00045015	chr8	1,297,013	1,297,208	195	Loss
DEL00045016	chr8	1,346,495	1,346,595	100	Loss
DEL00045021	chr8	1,381,829	1,382,214	385	Loss
DEL00045031	chr8	1,481,982	1,482,242	260	Loss
DEL00045040	chr8	1,656,587	1,656,653	66	Loss
DEL00045041	chr8	1,657,905	1,657,958	53	Loss
DEL00045043	chr8	1,725,345	1,725,413	68	Loss
DEL00045044	chr8	1,816,600	1,816,820	220	Loss
DEL00045045	chr8	1,840,402	1,840,873	471	Loss
DEL00045046	chr8	1,893,115	1,893,368	253	Loss
DEL00045048	chr8	1,914,612	1,914,668	56	Loss
DEL00045052	chr8	1,966,609	1,969,428	2,819	Loss
DEL00045063	chr8	2,070,299	2,070,551	252	Loss
DEL00045066	chr8	2,103,835	2,103,971	136	Loss
DEL00045069	chr8	2,181,043	2,181,791	748	Loss
DEL00045072	chr8	2,243,336	2,243,589	253	Loss
DEL00045073	chr8	2,300,515	2,302,007	1,492	Loss
DEL00045075	chr8	2,330,323	2,330,988	665	Loss
DEL00045076	chr8	2,347,644	2,347,793	149	Loss
DEL00045077	chr8	2,370,170	2,370,283	113	Loss
DEL00045090	chr8	2,583,633	2,583,939	306	Loss
DEL00045096	chr8	2,634,771	2,635,080	309	Loss
DEL00045099	chr8	2,657,858	2,659,433	1,575	Loss
DEL00045102	chr8	2,707,045	2,707,097	52	Loss
DEL00045120	chr8	2,910,462	2,911,589	1,127	Loss
DEL00045132	chr8	3,113,170	3,114,288	1,118	Loss
DEL00045135	chr8	3,136,342	3,136,430	88	Loss
DEL00045143	chr8	3,191,436	3,191,652	216	Loss

DEL00045145	chr8	3,195,004	3,195,160	156	Loss
DEL00045157	chr8	3,301,911	3,303,485	1,574	Loss
DEL00045158	chr8	3,307,515	3,307,567	52	Loss
DEL00045167	chr8	3,401,697	3,401,981	284	Loss
DEL00045169	chr8	3,425,477	3,426,468	991	Loss
DEL00045183	chr8	3,674,990	3,675,214	224	Loss
DEL00045187	chr8	3,734,079	3,734,170	91	Loss
DEL00045188	chr8	3,841,717	3,841,788	71	Loss
DEL00045191	chr8	3,888,309	3,888,377	68	Loss
DUP00045192	chr8	3,898,712	3,898,790	78	Gain
DUP00045195	chr8	3,909,922	3,910,097	175	Gain
DEL00045201	chr8	4,049,261	4,050,213	952	Loss
DEL00045205	chr8	4,126,903	4,126,961	58	Loss
DEL00045219	chr8	4,316,694	4,316,769	75	Loss
DEL00045221	chr8	4,347,985	4,348,255	270	Loss
DEL00045222	chr8	4,387,034	4,387,293	259	Loss
DEL00045230	chr8	4,603,425	4,603,477	52	Loss
DEL00045232	chr8	4,670,857	4,670,918	61	Loss
DEL00045233	chr8	4,792,088	4,792,162	74	Loss
DEL00045235	chr8	4,835,016	4,835,179	163	Loss
DEL00045240	chr8	4,875,215	4,875,284	69	Loss
DEL00045242	chr8	4,951,841	4,952,054	213	Loss
DEL00045246	chr8	5,089,212	5,089,807	595	Loss
DEL00045249	chr8	5,159,244	5,159,364	120	Loss
DEL00045255	chr8	5,361,307	5,361,395	88	Loss
DEL00045265	chr8	5,525,989	5,526,143	154	Loss
DEL00045278	chr8	5,856,500	5,856,565	65	Loss
DEL00045288	chr8	6,108,271	6,108,608	337	Loss
DEL00045311	chr8	6,771,131	6,771,711	580	Loss
DEL00045320	chr8	6,939,051	6,939,743	692	Loss
DEL00045326	chr8	7,124,426	7,124,550	124	Loss
DEL00045327	chr8	7,124,832	7,125,969	1,137	Loss
DEL00045350	chr8	7,513,233	7,513,304	71	Loss
DEL00045351	chr8	7,518,847	7,519,441	594	Loss
DEL00045363	chr8	7,713,439	7,713,508	69	Loss
DEL00045364	chr8	7,777,723	7,777,792	69	Loss
DEL00045368	chr8	7,808,515	7,808,634	119	Loss
DEL00045372	chr8	7,966,077	7,966,312	235	Loss
DEL00045373	chr8	7,972,084	7,972,381	297	Loss
DEL00045376	chr8	8,023,985	8,024,270	285	Loss
DEL00045382	chr8	8,194,542	8,197,227	2,685	Loss
DEL00045386	chr8	8,229,243	8,229,386	143	Loss
DEL00045387	chr8	8,256,876	8,256,958	82	Loss
DEL00045391	chr8	8,324,918	8,324,980	62	Loss
DEL00045394	chr8	8,357,009	8,367,532	10,523	Loss
DEL00045398	chr8	8,453,120	8,453,234	114	Loss
DEL00045401	chr8	8,465,794	8,466,323	529	Loss
DEL00045405	chr8	8,595,337	8,596,169	832	Loss
DEL00045410	chr8	8,680,360	8,680,786	426	Loss

DEL00045421	chr8	8,730,780	8,730,889	109	Loss
DEL00045423	chr8	8,832,222	8,832,719	497	Loss
DEL00045426	chr8	8,871,099	8,871,489	390	Loss
DEL00045433	chr8	9,208,057	9,210,090	2,033	Loss
DEL00045439	chr8	9,239,240	9,239,541	301	Loss
DEL00045444	chr8	9,286,139	9,286,218	79	Loss
DUP00045448	chr8	9,353,462	9,353,696	234	Gain
DEL00045452	chr8	9,408,462	9,410,504	2,042	Loss
DEL00045453	chr8	9,418,618	9,419,455	837	Loss
DEL00045465	chr8	9,606,418	9,607,663	1,245	Loss
DEL00045498	chr8	9,996,189	9,998,479	2,290	Loss
DEL00045500	chr8	10,004,060	10,005,195	1,135	Loss
DEL00045502	chr8	10,030,691	10,031,128	437	Loss
DEL00045519	chr8	10,424,798	10,424,862	64	Loss
DEL00045529	chr8	10,776,724	10,776,910	186	Loss
DEL00045543	chr8	10,918,258	10,918,417	159	Loss
DEL00045556	chr8	11,373,423	11,375,536	2,113	Loss
DEL00045562	chr8	11,689,751	11,689,868	117	Loss
DEL00045593	chr8	12,594,334	12,595,013	679	Loss
DEL00045601	chr8	12,742,590	12,742,973	383	Loss
DEL00045602	chr8	12,751,022	12,751,135	113	Loss
DEL00045604	chr8	12,788,077	12,788,249	172	Loss
DEL00045605	chr8	12,802,597	12,803,099	502	Loss
DEL00045613	chr8	13,033,519	13,039,056	5,537	Loss
DEL00045629	chr8	13,260,758	13,260,823	65	Loss
DEL00045630	chr8	13,266,314	13,267,172	858	Loss
DEL00045633	chr8	13,295,287	13,295,529	242	Loss
DEL00045635	chr8	13,309,817	13,309,881	64	Loss
DEL00045636	chr8	13,310,402	13,310,560	158	Loss
DEL00045638	chr8	13,388,750	13,388,821	71	Loss
DEL00045653	chr8	13,572,583	13,572,641	58	Loss
DEL00045654	chr8	13,616,514	13,617,367	853	Loss
DEL00045659	chr8	13,676,302	13,676,359	57	Loss
DEL00045664	chr8	13,848,635	13,848,694	59	Loss
DEL00045665	chr8	13,849,311	13,850,295	984	Loss
DEL00045668	chr8	13,885,361	13,885,682	321	Loss
DEL00045672	chr8	14,023,561	14,023,635	74	Loss
DEL00045675	chr8	14,054,020	14,054,999	979	Loss
DEL00045681	chr8	14,142,277	14,142,418	141	Loss
DEL00045685	chr8	14,296,347	14,297,265	918	Loss
DEL00045687	chr8	14,324,306	14,324,419	113	Loss
DEL00045688	chr8	14,346,846	14,346,997	151	Loss
DEL00045713	chr8	14,702,280	14,702,483	203	Loss
DEL00045717	chr8	14,778,633	14,778,744	111	Loss
DEL00045719	chr8	14,886,223	14,886,759	536	Loss
DEL00045730	chr8	15,122,727	15,123,229	502	Loss
DEL00045736	chr8	15,164,081	15,164,763	682	Loss
DEL00045739	chr8	15,176,500	15,176,566	66	Loss
DEL00045746	chr8	15,358,194	15,358,273	79	Loss

DEL00045760	chr8	15,604,982	15,605,145	163	Loss
DEL00045764	chr8	15,792,345	15,792,640	295	Loss
DEL00045769	chr8	15,922,814	15,922,993	179	Loss
DEL00045772	chr8	16,124,641	16,124,712	71	Loss
DEL00045784	chr8	16,235,510	16,235,995	485	Loss
DEL00045786	chr8	16,252,157	16,252,212	55	Loss
DEL00045808	chr8	16,408,391	16,408,478	87	Loss
DEL00045814	chr8	16,532,113	16,533,233	1,120	Loss
DEL00045817	chr8	16,583,128	16,583,547	419	Loss
DEL00045819	chr8	16,621,532	16,621,621	89	Loss
DEL00045826	chr8	16,649,737	16,650,249	512	Loss
DEL00045833	chr8	16,729,141	16,729,202	61	Loss
DEL00045841	chr8	16,862,575	16,863,058	483	Loss
DEL00045848	chr8	16,959,681	16,959,757	76	Loss
DEL00045866	chr8	17,098,462	17,098,560	98	Loss
DEL00045869	chr8	17,153,198	17,153,973	775	Loss
DEL00045892	chr8	17,587,230	17,587,282	52	Loss
DEL00045899	chr8	17,661,008	17,661,068	60	Loss
DEL00045900	chr8	17,675,554	17,675,969	415	Loss
DEL00045905	chr8	17,733,892	17,734,059	167	Loss
DEL00045906	chr8	17,737,498	17,737,582	84	Loss
DEL00045913	chr8	17,898,933	17,899,084	151	Loss
DUP00045918	chr8	18,002,212	18,002,272	60	Gain
DUP00045922	chr8	18,131,301	18,131,403	102	Gain
DEL00045926	chr8	18,340,153	18,340,219	66	Loss
DEL00045929	chr8	18,503,695	18,503,940	245	Loss
DEL00045931	chr8	18,552,091	18,552,168	77	Loss
DEL00045933	chr8	18,557,692	18,557,805	113	Loss
DEL00045943	chr8	18,614,618	18,615,445	827	Loss
DEL00045945	chr8	18,676,308	18,676,590	282	Loss
DEL00045946	chr8	18,687,253	18,687,622	369	Loss
DEL00045951	chr8	18,788,962	18,789,611	649	Loss
DEL00045960	chr8	18,917,605	18,917,951	346	Loss
DEL00045968	chr8	19,008,341	19,012,454	4,113	Loss
DEL00045973	chr8	19,075,469	19,075,526	57	Loss
DEL00045976	chr8	19,167,712	19,167,808	96	Loss
DEL00045978	chr8	19,221,075	19,221,319	244	Loss
DEL00045981	chr8	19,309,739	19,309,942	203	Loss
DEL00045982	chr8	19,310,246	19,310,354	108	Loss
DEL00045983	chr8	19,321,346	19,321,598	252	Loss
DEL00045986	chr8	19,501,036	19,501,219	183	Loss
DEL00045997	chr8	19,645,807	19,646,081	274	Loss
DEL00046000	chr8	19,883,542	19,883,626	84	Loss
DEL00046006	chr8	20,009,279	20,009,535	256	Loss
DEL00046009	chr8	20,025,520	20,025,682	162	Loss
DEL00046014	chr8	20,282,411	20,282,573	162	Loss
DEL00046015	chr8	20,320,470	20,320,538	68	Loss
DUP00046021	chr8	20,540,734	20,541,896	1,162	Gain
DEL00046047	chr8	20,820,808	20,821,320	512	Loss

DEL00046051	chr8	20,880,980	20,881,303	323	Loss
DEL00046052	chr8	20,933,263	20,933,467	204	Loss
DEL00046053	chr8	20,936,226	20,936,532	306	Loss
DEL00046057	chr8	21,063,287	21,063,345	58	Loss
DEL00046066	chr8	21,369,194	21,369,252	58	Loss
DEL00046082	chr8	21,792,908	21,792,967	59	Loss
DEL00046085	chr8	21,871,892	21,872,011	119	Loss
DEL00046087	chr8	21,898,569	21,898,666	97	Loss
DEL00046092	chr8	21,985,257	21,985,594	337	Loss
DEL00046094	chr8	21,990,036	21,990,363	327	Loss
DEL00046096	chr8	22,014,263	22,014,389	126	Loss
DEL00046113	chr8	22,403,035	22,403,114	79	Loss
DEL00046114	chr8	22,408,109	22,408,275	166	Loss
DEL00046117	chr8	22,465,882	22,466,968	1,086	Loss
DEL00046122	chr8	22,669,788	22,669,846	58	Loss
DEL00046129	chr8	22,898,238	22,898,371	133	Loss
DEL00046130	chr8	22,914,685	22,914,739	54	Loss
DEL00046132	chr8	22,926,004	22,926,087	83	Loss
DEL00046133	chr8	22,944,784	22,948,144	3,360	Loss
DEL00046141	chr8	23,012,939	23,013,043	104	Loss
DEL00046155	chr8	23,169,348	23,169,665	317	Loss
DEL00046165	chr8	23,244,764	23,244,937	173	Loss
DEL00046167	chr8	23,394,508	23,394,651	143	Loss
DEL00046170	chr8	23,481,609	23,481,725	116	Loss
DUP00046171	chr8	23,570,689	23,591,145	20,456	Gain
DEL00046172	chr8	23,645,196	23,645,264	68	Loss
DEL00046186	chr8	23,858,920	23,859,466	546	Loss
DEL00046202	chr8	24,245,115	24,245,211	96	Loss
DEL00046204	chr8	24,258,429	24,258,676	247	Loss
DEL00046206	chr8	24,261,088	24,261,140	52	Loss
DEL00046213	chr8	24,402,957	24,403,027	70	Loss
DEL00046214	chr8	24,408,960	24,409,024	64	Loss
DEL00046216	chr8	24,423,989	24,424,102	113	Loss
DEL00046217	chr8	24,425,343	24,425,786	443	Loss
DEL00046220	chr8	24,435,236	24,435,291	55	Loss
DEL00046222	chr8	24,535,720	24,535,823	103	Loss
DEL00046225	chr8	24,560,629	24,563,831	3,202	Loss
DEL00046228	chr8	24,586,160	24,586,576	416	Loss
DEL00046237	chr8	24,756,444	24,756,666	222	Loss
DEL00046238	chr8	24,759,546	24,759,610	64	Loss
DEL00046241	chr8	24,811,597	24,811,691	94	Loss
DUP00046242	chr8	24,817,745	24,817,843	98	Gain
DEL00046243	chr8	24,856,754	24,856,806	52	Loss
DEL00046244	chr8	24,861,401	24,861,660	259	Loss
DEL00046254	chr8	25,049,861	25,049,959	98	Loss
DEL00046257	chr8	25,120,734	25,120,789	55	Loss
DEL00046261	chr8	25,160,351	25,160,642	291	Loss
DEL00046263	chr8	25,255,210	25,255,401	191	Loss
DEL00046264	chr8	25,264,564	25,264,618	54	Loss

DEL00046269	chr8	25,442,523	25,443,179	656	Loss
DEL00046275	chr8	25,614,336	25,614,416	80	Loss
DEL00046279	chr8	25,675,326	25,676,214	888	Loss
DEL00046287	chr8	25,762,056	25,762,533	477	Loss
DEL00046289	chr8	25,826,580	25,827,069	489	Loss
DEL00046291	chr8	25,964,049	25,964,270	221	Loss
DEL00046304	chr8	26,441,296	26,441,377	81	Loss
DUP00046332	chr8	26,503,984	26,504,109	125	Gain
DEL00046333	chr8	26,520,197	26,520,590	393	Loss
DEL00046334	chr8	26,549,947	26,550,052	105	Loss
DEL00046339	chr8	26,665,686	26,665,741	55	Loss
DEL00046341	chr8	26,742,593	26,742,756	163	Loss
DEL00046342	chr8	26,753,279	26,753,530	251	Loss
DEL00046351	chr8	27,121,588	27,121,695	107	Loss
DEL00046352	chr8	27,188,204	27,188,949	745	Loss
DEL00046354	chr8	27,293,611	27,293,744	133	Loss
DEL00046359	chr8	27,307,307	27,307,623	316	Loss
DEL00046372	chr8	27,355,671	27,355,788	117	Loss
DEL00046373	chr8	27,373,984	27,374,148	164	Loss
DEL00046374	chr8	27,483,352	27,483,461	109	Loss
DEL00046379	chr8	27,580,333	27,580,791	458	Loss
DEL00046392	chr8	27,613,275	27,613,575	300	Loss
DEL00046395	chr8	27,662,388	27,662,679	291	Loss
DEL00046399	chr8	27,681,595	27,681,770	175	Loss
DEL00046402	chr8	27,731,619	27,732,024	405	Loss
DEL00046427	chr8	27,827,901	27,827,955	54	Loss
DEL00046428	chr8	27,889,814	27,889,869	55	Loss
DEL00046436	chr8	27,988,084	27,988,312	228	Loss
DEL00046437	chr8	27,994,063	27,994,413	350	Loss
DUP00046438	chr8	27,999,862	27,999,972	110	Gain
DEL00046444	chr8	28,166,898	28,167,007	109	Loss
DEL00046453	chr8	28,386,616	28,386,860	244	Loss
DUP00046454	chr8	28,389,147	28,389,318	171	Gain
DEL00046461	chr8	28,565,266	28,565,332	66	Loss
DUP00046471	chr8	28,822,245	28,822,370	125	Gain
DEL00046475	chr8	28,859,225	28,859,335	110	Loss
DEL00046477	chr8	28,912,541	28,912,663	122	Loss
DEL00046479	chr8	28,916,775	28,917,261	486	Loss
DEL00046483	chr8	28,983,167	28,983,565	398	Loss
DEL00046501	chr8	29,242,384	29,242,708	324	Loss
DEL00046503	chr8	29,295,107	29,296,121	1,014	Loss
DEL00046504	chr8	29,342,304	29,342,836	532	Loss
DUP00046509	chr8	29,401,968	29,402,050	82	Gain
DEL00046513	chr8	29,471,492	29,474,142	2,650	Loss
DEL00046517	chr9	5,616	5,714	98	Loss
DEL00046527	chr9	234,611	234,674	63	Loss
DEL00046528	chr9	235,320	235,422	102	Loss
DEL00046530	chr9	262,296	262,370	74	Loss
DEL00046531	chr9	268,385	268,485	100	Loss

DEL00046537	chr9	527,447	527,797	350	Loss
DEL00046540	chr9	605,886	605,963	77	Loss
DEL00046553	chr9	878,983	879,305	322	Loss
DEL00046565	chr9	1,122,296	1,122,585	289	Loss
DEL00046574	chr9	1,307,342	1,307,402	60	Loss
DEL00046577	chr9	1,358,674	1,358,760	86	Loss
DEL00046579	chr9	1,360,869	1,361,171	302	Loss
DEL00046587	chr9	1,491,539	1,493,632	2,093	Loss
DEL00046588	chr9	1,497,962	1,498,258	296	Loss
DEL00046590	chr9	1,631,904	1,632,062	158	Loss
DEL00046599	chr9	1,772,035	1,772,159	124	Loss
DEL00046603	chr9	1,846,472	1,846,575	103	Loss
DEL00046614	chr9	1,964,739	1,964,845	106	Loss
DEL00046616	chr9	2,018,221	2,018,391	170	Loss
DEL00046626	chr9	2,148,101	2,148,232	131	Loss
DEL00046630	chr9	2,185,026	2,185,161	135	Loss
DEL00046634	chr9	2,194,762	2,196,194	1,432	Loss
DEL00046635	chr9	2,198,478	2,199,469	991	Loss
DEL00046636	chr9	2,201,469	2,201,573	104	Loss
DEL00046637	chr9	2,202,416	2,203,194	778	Loss
DEL00046649	chr9	2,333,822	2,334,037	215	Loss
DEL00046653	chr9	2,387,115	2,387,204	89	Loss
DEL00046654	chr9	2,424,876	2,424,956	80	Loss
DEL00046658	chr9	2,455,784	2,456,123	339	Loss
DEL00046664	chr9	2,504,466	2,504,592	126	Loss
DEL00046670	chr9	2,533,637	2,534,428	791	Loss
DEL00046676	chr9	2,561,035	2,561,110	75	Loss
DEL00046680	chr9	2,591,456	2,591,896	440	Loss
DEL00046694	chr9	2,678,639	2,678,691	52	Loss
DEL00046695	chr9	2,684,429	2,685,941	1,512	Loss
DEL00046699	chr9	2,739,775	2,740,002	227	Loss
DEL00046711	chr9	2,866,587	2,866,770	183	Loss
DEL00046714	chr9	2,895,669	2,895,897	228	Loss
DEL00046733	chr9	2,984,578	2,985,169	591	Loss
DEL00046739	chr9	3,117,029	3,117,137	108	Loss
DEL00046742	chr9	3,158,927	3,159,043	116	Loss
DEL00046744	chr9	3,186,089	3,186,227	138	Loss
DEL00046749	chr9	3,331,968	3,332,039	71	Loss
DEL00046751	chr9	3,368,802	3,372,567	3,765	Loss
DEL00046752	chr9	3,405,772	3,405,835	63	Loss
DEL00046757	chr9	3,483,400	3,483,704	304	Loss
DEL00046769	chr9	3,648,425	3,648,526	101	Loss
DEL00046771	chr9	3,666,171	3,666,753	582	Loss
DEL00046774	chr9	3,728,640	3,728,828	188	Loss
DUP00046789	chr9	3,893,387	3,908,511	15,124	Gain
DEL00046793	chr9	4,046,080	4,046,500	420	Loss
DEL00046798	chr9	4,205,395	4,205,572	177	Loss
DEL00046801	chr9	4,242,280	4,242,373	93	Loss
DEL00046804	chr9	4,350,464	4,351,189	725	Loss

DEL00046807	chr9	4,418,957	4,419,140	183	Loss
DEL00046815	chr9	4,529,386	4,529,450	64	Loss
DEL00046816	chr9	4,532,060	4,532,134	74	Loss
DEL00046818	chr9	4,566,655	4,566,710	55	Loss
DEL00046820	chr9	4,637,172	4,637,843	671	Loss
DEL00046823	chr9	4,701,357	4,701,415	58	Loss
DEL00046826	chr9	4,864,758	4,864,865	107	Loss
DEL00046835	chr9	4,982,363	4,982,806	443	Loss
DEL00046842	chr9	5,081,185	5,081,567	382	Loss
DEL00046844	chr9	5,095,990	5,096,043	53	Loss
DEL00046851	chr9	5,167,589	5,167,667	78	Loss
DEL00046854	chr9	5,262,905	5,262,964	59	Loss
DEL00046865	chr9	5,455,149	5,455,563	414	Loss
DEL00046868	chr9	5,473,288	5,473,768	480	Loss
DEL00046870	chr9	5,560,377	5,561,761	1,384	Loss
DEL00046873	chr9	5,577,539	5,578,506	967	Loss
DEL00046874	chr9	5,580,214	5,580,316	102	Loss
DEL00046880	chr9	5,729,819	5,729,913	94	Loss
DEL00046881	chr9	5,738,005	5,738,292	287	Loss
DEL00046889	chr9	5,843,131	5,843,225	94	Loss
DEL00046891	chr9	5,874,643	5,874,702	59	Loss
DEL00046894	chr9	5,944,552	5,944,849	297	Loss
DEL00046897	chr9	6,025,090	6,027,759	2,669	Loss
DEL00046908	chr9	6,305,467	6,306,550	1,083	Loss
DEL00046910	chr9	6,313,113	6,313,326	213	Loss
DUP00046912	chr9	6,362,103	6,376,341	14,238	Gain
DEL00046913	chr9	6,416,648	6,421,049	4,401	Loss
DEL00046927	chr9	6,566,849	6,569,936	3,087	Loss
DEL00046948	chr9	6,784,051	6,784,115	64	Loss
DEL00046964	chr9	7,052,712	7,053,126	414	Loss
DEL00046969	chr9	7,183,610	7,183,947	337	Loss
DEL00046980	chr9	7,446,895	7,447,091	196	Loss
DEL00046988	chr9	7,690,440	7,690,541	101	Loss
DEL00046989	chr9	7,725,800	7,726,348	548	Loss
DEL00046991	chr9	7,726,652	7,726,719	67	Loss
DEL00046995	chr9	7,783,106	7,783,162	56	Loss
DEL00046999	chr9	7,948,226	7,948,403	177	Loss
DEL00047000	chr9	7,957,697	7,957,879	182	Loss
DEL00047004	chr9	8,004,352	8,005,314	962	Loss
DEL00047008	chr9	8,113,225	8,113,430	205	Loss
DEL00047009	chr9	8,116,726	8,116,852	126	Loss
DEL00047011	chr9	8,175,610	8,179,350	3,740	Loss
DEL00047014	chr9	8,204,122	8,204,222	100	Loss
DEL00047019	chr9	8,297,405	8,297,458	53	Loss
DEL00047028	chr9	8,470,364	8,471,073	709	Loss
DEL00047029	chr9	8,489,686	8,489,826	140	Loss
DEL00047035	chr9	8,503,695	8,504,298	603	Loss
DEL00047040	chr9	8,560,548	8,565,795	5,247	Loss
DEL00047047	chr9	8,722,967	8,723,410	443	Loss

DEL00047063	chr9	8,965,817	8,966,266	449	Loss
DEL00047065	chr9	8,982,897	8,983,468	571	Loss
DEL00047067	chr9	9,011,628	9,012,129	501	Loss
DEL00047068	chr9	9,032,889	9,033,405	516	Loss
DEL00047072	chr9	9,066,077	9,066,726	649	Loss
DEL00047075	chr9	9,126,471	9,126,540	69	Loss
DEL00047085	chr9	9,312,221	9,312,617	396	Loss
DEL00047092	chr9	9,480,584	9,488,624	8,040	Loss
DEL00047093	chr9	9,489,386	9,489,573	187	Loss
DEL00047096	chr9	9,509,011	9,509,241	230	Loss
DEL00047098	chr9	9,612,357	9,612,419	62	Loss
DEL00047101	chr9	9,692,829	9,693,133	304	Loss
DEL00047108	chr9	9,777,631	9,777,835	204	Loss
DEL00047109	chr9	9,785,690	9,786,423	733	Loss
DEL00047110	chr9	9,790,932	9,791,011	79	Loss
DUP00047112	chr9	9,827,658	9,828,514	856	Gain
DEL00047116	chr9	9,899,218	9,899,874	656	Loss
DEL00047123	chr9	10,161,404	10,161,498	94	Loss
DEL00047126	chr9	10,196,234	10,197,378	1,144	Loss
DEL00047131	chr9	10,240,497	10,240,763	266	Loss
DEL00047150	chr9	10,635,437	10,639,906	4,469	Loss
DEL00047154	chr9	10,800,229	10,800,291	62	Loss
DEL00047157	chr9	10,848,426	10,852,570	4,144	Loss
DEL00047162	chr9	10,925,735	10,925,972	237	Loss
DEL00047189	chr9	11,103,892	11,104,103	211	Loss
DEL00047191	chr9	11,118,990	11,119,047	57	Loss
DEL00047197	chr9	11,236,080	11,243,612	7,532	Loss
DEL00047205	chr9	11,397,402	11,404,890	7,488	Loss
DEL00047210	chr9	11,414,799	11,419,969	5,170	Loss
DEL00047219	chr9	11,683,266	11,683,579	313	Loss
DEL00047225	chr9	11,969,997	11,970,085	88	Loss
DEL00047236	chr9	12,239,528	12,239,661	133	Loss
DEL00047237	chr9	12,254,746	12,254,837	91	Loss
DEL00047244	chr9	12,374,310	12,374,610	300	Loss
DEL00047249	chr9	12,543,021	12,546,089	3,068	Loss
DEL00047250	chr9	12,587,997	12,592,343	4,346	Loss
DEL00047271	chr9	12,728,341	12,729,464	1,123	Loss
DEL00047279	chr9	12,729,605	12,730,024	419	Loss
DEL00047286	chr9	12,883,614	12,883,765	151	Loss
DEL00047287	chr9	12,895,080	12,895,238	158	Loss
DEL00047288	chr9	12,897,511	12,897,712	201	Loss
DEL00047291	chr9	12,950,327	12,950,672	345	Loss
DEL00047296	chr9	13,048,017	13,048,238	221	Loss
DEL00047297	chr9	13,075,598	13,075,825	227	Loss
DEL00047299	chr9	13,182,716	13,183,242	526	Loss
DEL00047301	chr9	13,233,428	13,233,741	313	Loss
DEL00047310	chr9	13,517,273	13,517,350	77	Loss
DEL00047311	chr9	13,527,368	13,527,433	65	Loss
DEL00047312	chr9	13,555,955	13,556,010	55	Loss

DEL00047327	chr9	14,003,458	14,004,219	761	Loss
DEL00047328	chr9	14,046,026	14,046,278	252	Loss
DEL00047335	chr9	14,229,208	14,229,495	287	Loss
DUP00047344	chr9	14,531,920	14,533,226	1,306	Gain
DEL00047349	chr9	14,549,013	14,549,756	743	Loss
DEL00047357	chr9	14,550,335	14,551,044	709	Loss
DEL00047366	chr9	14,833,853	14,833,911	58	Loss
DEL00047371	chr9	14,946,792	14,946,897	105	Loss
DEL00047374	chr9	15,068,362	15,069,133	771	Loss
DEL00047379	chr9	15,340,765	15,340,881	116	Loss
DEL00047380	chr9	15,366,113	15,366,308	195	Loss
DEL00047397	chr9	15,807,267	15,807,703	436	Loss
DEL00047400	chr9	15,841,889	15,842,216	327	Loss
DEL00047402	chr9	15,885,186	15,885,254	68	Loss
DEL00047406	chr9	15,897,868	15,897,975	107	Loss
DEL00047407	chr9	15,909,737	15,910,193	456	Loss
DEL00047413	chr9	16,043,071	16,043,141	70	Loss
DEL00047416	chr9	16,161,674	16,161,851	177	Loss
DUP00047417	chr9	16,253,839	16,262,565	8,726	Gain
DEL00047429	chr9	16,610,495	16,611,545	1,050	Loss
DEL00047431	chr9	16,633,436	16,633,520	84	Loss
DEL00047434	chr9	16,691,249	16,691,321	72	Loss
DEL00047435	chr9	16,692,773	16,693,014	241	Loss
DEL00047437	chr9	16,746,816	16,746,918	102	Loss
DEL00047440	chr9	16,788,571	16,788,772	201	Loss
DEL00047444	chr9	16,868,452	16,868,609	157	Loss
DEL00047447	chr9	16,884,685	16,885,755	1,070	Loss
DEL00047456	chr9	17,016,113	17,016,183	70	Loss
DEL00047461	chr9	17,165,939	17,166,259	320	Loss
DEL00047464	chr9	17,482,785	17,482,836	51	Loss
DEL00047471	chr9	17,662,121	17,662,348	227	Loss
DEL00047474	chr9	17,701,303	17,701,726	423	Loss
DEL00047476	chr9	17,793,916	17,794,666	750	Loss
DEL00047479	chr9	17,836,996	17,837,318	322	Loss
DEL00047480	chr9	17,897,347	17,901,526	4,179	Loss
DEL00047481	chr9	17,935,405	17,935,456	51	Loss
DEL00047483	chr9	17,955,278	17,955,384	106	Loss
DEL00047489	chr9	18,035,364	18,035,557	193	Loss
DEL00047498	chr9	18,313,643	18,313,725	82	Loss
DEL00047500	chr9	18,351,891	18,352,034	143	Loss
DEL00047502	chr9	18,457,590	18,458,437	847	Loss
DEL00047509	chr9	18,639,136	18,639,431	295	Loss
DEL00047521	chr9	18,880,053	18,880,353	300	Loss
DEL00047525	chr9	19,062,505	19,062,642	137	Loss
DEL00047531	chr9	19,179,404	19,179,812	408	Loss
DEL00047536	chr9	19,390,114	19,390,174	60	Loss
DEL00047537	chr9	19,492,729	19,493,050	321	Loss
DEL00047541	chr9	19,569,379	19,569,441	62	Loss
DEL00047545	chr9	19,690,735	19,690,806	71	Loss

DEL00047546	chr9	19,747,673	19,748,951	1,278	Loss
DEL00047551	chr9	20,080,124	20,081,105	981	Loss
DEL00047556	chr9	20,181,611	20,182,162	551	Loss
DEL00047562	chr9	20,366,833	20,367,047	214	Loss
DEL00047571	chr9	20,613,570	20,615,491	1,921	Loss
DEL00047594	chr9	21,339,730	21,339,984	254	Loss
DEL00047596	chr9	21,398,603	21,399,119	516	Loss
DEL00047597	chr9	21,463,584	21,463,760	176	Loss
DEL00047605	chr9	21,667,409	21,667,488	79	Loss
DEL00047613	chr9	21,969,106	21,969,250	144	Loss
DEL00047615	chr9	22,013,704	22,013,819	115	Loss
DEL00047620	chr9	22,112,491	22,112,554	63	Loss
DEL00047624	chr9	22,209,605	22,209,682	77	Loss
DEL00047639	chr9	22,311,028	22,311,300	272	Loss
DEL00047658	chr9	22,704,224	22,704,433	209	Loss
DEL00047664	chr9	22,815,978	22,816,118	140	Loss
DEL00047665	chr9	22,824,342	22,824,486	144	Loss
DEL00047666	chr9	22,896,151	22,896,223	72	Loss
DEL00047670	chr9	23,054,340	23,054,582	242	Loss
DEL00047671	chr9	23,095,133	23,095,800	667	Loss
DEL00047692	chr9	23,520,413	23,520,814	401	Loss
DUP00047695	chr9	23,521,010	23,521,915	905	Mixed
DEL00047709	chr9	23,522,446	23,522,820	374	Loss
DEL00047861	chr10	171,220	184,045	12,825	Loss
DUP00047980	chr10	390,412	392,395	1,983	Gain
DEL00047984	chr10	449,535	450,599	1,064	Loss
DEL00047985	chr10	465,668	466,848	1,180	Loss
DEL00047999	chr10	583,153	583,859	706	Loss
DEL00048021	chr10	657,057	657,314	257	Loss
DEL00048022	chr10	662,297	662,398	101	Loss
DEL00048025	chr10	676,425	676,484	59	Loss
DEL00048030	chr10	798,251	798,313	62	Loss
DEL00048036	chr10	862,560	862,784	224	Loss
DEL00048038	chr10	873,307	873,460	153	Loss
DEL00048039	chr10	902,839	903,517	678	Loss
DEL00048057	chr10	1,256,732	1,256,783	51	Loss
DEL00048067	chr10	1,355,873	1,355,926	53	Loss
DEL00048068	chr10	1,367,759	1,367,894	135	Loss
DEL00048069	chr10	1,385,785	1,385,905	120	Loss
DEL00048072	chr10	1,404,480	1,404,536	56	Loss
DEL00048087	chr10	1,552,083	1,552,137	54	Loss
DEL00048101	chr10	1,798,948	1,799,254	306	Loss
DEL00048108	chr10	1,973,489	1,974,503	1,014	Loss
DEL00048121	chr10	2,146,973	2,147,691	718	Mixed
DEL00048142	chr10	2,335,090	2,335,182	92	Loss
DUP00048145	chr10	2,371,394	2,371,954	560	Gain
DEL00048161	chr10	2,539,583	2,539,655	72	Loss
DEL00048178	chr10	2,663,092	2,663,206	114	Loss
DEL00048179	chr10	2,672,248	2,672,466	218	Loss

DEL00048180	chr10	2,771,475	2,772,012	537	Loss
DEL00048189	chr10	2,848,460	2,848,518	58	Loss
DEL00048190	chr10	2,848,642	2,848,741	99	Loss
DEL00048191	chr10	2,883,802	2,884,057	255	Loss
DEL00048193	chr10	2,930,766	2,930,884	118	Loss
DEL00048194	chr10	2,960,196	2,960,310	114	Loss
DEL00048200	chr10	3,005,333	3,005,438	105	Loss
DEL00048201	chr10	3,006,768	3,007,284	516	Loss
DEL00048204	chr10	3,011,976	3,012,476	500	Loss
DEL00048213	chr10	3,055,792	3,055,880	88	Loss
DEL00048217	chr10	3,124,420	3,124,836	416	Loss
DEL00048225	chr10	3,201,601	3,201,715	114	Loss
DEL00048233	chr10	3,219,576	3,219,752	176	Loss
DEL00048240	chr10	3,322,291	3,322,469	178	Loss
DEL00048243	chr10	3,375,009	3,375,552	543	Loss
DEL00048247	chr10	3,413,934	3,414,770	836	Loss
DEL00048249	chr10	3,443,848	3,444,318	470	Loss
DEL00048255	chr10	3,573,554	3,573,814	260	Loss
DEL00048259	chr10	3,588,883	3,589,575	692	Loss
DEL00048261	chr10	3,616,556	3,616,664	108	Loss
DEL00048262	chr10	3,671,263	3,671,424	161	Loss
DEL00048263	chr10	3,675,778	3,676,038	260	Loss
DEL00048269	chr10	3,886,484	3,886,543	59	Loss
DEL00048270	chr10	3,905,266	3,905,319	53	Loss
DEL00048272	chr10	3,923,002	3,923,437	435	Loss
DEL00048279	chr10	4,014,763	4,014,833	70	Loss
DEL00048281	chr10	4,056,925	4,057,001	76	Loss
DEL00048288	chr10	4,167,964	4,170,013	2,049	Loss
DEL00048301	chr10	4,349,345	4,349,423	78	Loss
DEL00048305	chr10	4,365,039	4,365,224	185	Loss
DEL00048320	chr10	4,680,274	4,680,636	362	Loss
DEL00048321	chr10	4,733,597	4,734,090	493	Loss
DEL00048323	chr10	4,805,398	4,809,624	4,226	Loss
DEL00048328	chr10	4,880,229	4,880,345	116	Loss
DEL00048339	chr10	5,219,609	5,219,663	54	Loss
DEL00048346	chr10	5,275,277	5,276,375	1,098	Loss
DEL00048348	chr10	5,338,494	5,338,607	113	Loss
DEL00048352	chr10	5,465,406	5,465,556	150	Loss
DEL00048353	chr10	5,481,071	5,481,179	108	Loss
DEL00048358	chr10	5,673,514	5,674,276	762	Loss
DEL00048362	chr10	6,013,124	6,013,678	554	Loss
DEL00048371	chr10	6,103,012	6,103,103	91	Loss
DEL00048372	chr10	6,161,781	6,161,888	107	Loss
DEL00048375	chr10	6,201,727	6,201,795	68	Loss
DEL00048379	chr10	6,362,838	6,362,908	70	Loss
DEL00048380	chr10	6,366,664	6,367,302	638	Loss
DEL00048383	chr10	6,426,474	6,426,556	82	Loss
DEL00048386	chr10	6,436,630	6,436,787	157	Loss
DEL00048387	chr10	6,458,232	6,458,437	205	Loss

DEL00048389	chr10	6,476,715	6,476,986	271	Loss
DEL00048395	chr10	6,589,654	6,589,902	248	Loss
DEL00048399	chr10	6,687,762	6,687,899	137	Loss
DEL00048401	chr10	6,726,133	6,726,827	694	Loss
DEL00048406	chr10	6,823,397	6,823,928	531	Loss
DEL00048409	chr10	6,863,057	6,864,370	1,313	Loss
DEL00048410	chr10	6,876,109	6,877,962	1,853	Loss
DEL00048412	chr10	6,885,648	6,885,703	55	Loss
DEL00048419	chr10	6,977,238	6,977,417	179	Loss
DEL00048420	chr10	6,986,652	6,986,925	273	Loss
DEL00048422	chr10	7,085,279	7,085,343	64	Loss
DEL00048426	chr10	7,157,436	7,157,509	73	Loss
DEL00048428	chr10	7,178,384	7,178,830	446	Loss
DEL00048432	chr10	7,281,745	7,282,493	748	Loss
DEL00048435	chr10	7,304,753	7,304,812	59	Loss
DEL00048442	chr10	7,449,096	7,449,545	449	Loss
DEL00048444	chr10	7,507,439	7,509,249	1,810	Loss
DEL00048446	chr10	7,560,417	7,560,936	519	Loss
DEL00048451	chr10	7,621,454	7,621,569	115	Loss
DEL00048453	chr10	7,685,577	7,686,273	696	Loss
DEL00048454	chr10	7,722,019	7,722,108	89	Loss
DEL00048455	chr10	7,722,124	7,722,229	105	Loss
DEL00048456	chr10	7,755,531	7,755,637	106	Loss
DEL00048465	chr10	7,897,222	7,897,384	162	Loss
DEL00048471	chr10	8,122,832	8,123,349	517	Loss
DEL00048485	chr10	8,590,890	8,590,995	105	Loss
DEL00048486	chr10	8,592,824	8,592,930	106	Loss
DEL00048489	chr10	8,666,204	8,666,939	735	Loss
DEL00048490	chr10	8,669,210	8,669,392	182	Loss
DEL00048507	chr10	8,843,331	8,843,643	312	Loss
DEL00048511	chr10	8,933,210	8,933,270	60	Loss
DEL00048515	chr10	9,006,476	9,006,836	360	Loss
DEL00048517	chr10	9,048,161	9,048,694	533	Loss
DEL00048522	chr10	9,066,184	9,066,759	575	Loss
DEL00048525	chr10	9,214,994	9,215,179	185	Loss
DEL00048527	chr10	9,232,156	9,232,392	236	Loss
DEL00048528	chr10	9,246,629	9,246,691	62	Loss
DEL00048530	chr10	9,246,959	9,247,622	663	Loss
DEL00048531	chr10	9,254,493	9,254,643	150	Loss
DEL00048539	chr10	9,594,738	9,595,611	873	Loss
DUP00048540	chr10	9,630,563	9,630,647	84	Gain
DEL00048543	chr10	9,660,457	9,660,516	59	Loss
DEL00048551	chr10	9,946,374	9,946,747	373	Loss
DEL00048555	chr10	9,992,826	9,993,128	302	Loss
DEL00048557	chr10	10,017,923	10,018,314	391	Loss
DEL00048561	chr10	10,041,406	10,041,648	242	Loss
DEL00048578	chr10	10,285,696	10,285,787	91	Loss
DEL00048582	chr10	10,313,863	10,314,131	268	Loss
DUP00048583	chr10	10,383,912	10,387,923	4,011	Gain

DEL00048598	chr10	10,650,442	10,650,494	52	Loss
DEL00048602	chr10	10,688,498	10,689,010	512	Loss
DEL00048607	chr10	10,854,881	10,854,962	81	Loss
DEL00048610	chr10	10,874,103	10,874,159	56	Loss
DEL00048615	chr10	11,010,097	11,010,199	102	Loss
DEL00048621	chr10	11,134,958	11,136,105	1,147	Loss
DEL00048628	chr10	11,195,315	11,195,573	258	Loss
DEL00048632	chr10	11,264,299	11,264,931	632	Loss
DEL00048639	chr10	11,373,967	11,374,525	558	Loss
DEL00048640	chr10	11,422,926	11,423,034	108	Loss
DEL00048642	chr10	11,475,499	11,475,690	191	Loss
DEL00048646	chr10	11,621,759	11,621,857	98	Loss
DEL00048648	chr10	11,683,737	11,683,802	65	Loss
DEL00048650	chr10	11,759,143	11,759,499	356	Loss
DEL00048651	chr10	11,762,290	11,762,352	62	Loss
DEL00048677	chr10	12,346,194	12,346,367	173	Loss
DEL00048707	chr10	12,770,657	12,770,714	57	Loss
DEL00048714	chr10	13,134,381	13,143,666	9,285	Loss
DEL00048716	chr10	13,272,459	13,272,532	73	Loss
DEL00048717	chr10	13,280,898	13,281,448	550	Loss
DEL00048723	chr10	13,384,383	13,384,507	124	Loss
DEL00048724	chr10	13,537,601	13,541,154	3,553	Loss
DEL00048725	chr10	13,558,339	13,558,541	202	Loss
DEL00048727	chr10	13,615,808	13,618,768	2,960	Loss
DEL00048733	chr10	13,765,674	13,765,743	69	Loss
DEL00048737	chr10	13,920,825	13,920,896	71	Loss
DEL00048743	chr10	14,058,834	14,059,395	561	Loss
DEL00048745	chr10	14,068,496	14,068,678	182	Loss
DEL00048746	chr10	14,070,138	14,070,238	100	Loss
DEL00048752	chr10	14,200,972	14,201,181	209	Loss
DEL00048754	chr10	14,223,671	14,223,724	53	Loss
DEL00048758	chr10	14,364,124	14,364,191	67	Loss
DEL00048762	chr10	14,564,575	14,564,653	78	Loss
DEL00048766	chr10	14,598,276	14,598,678	402	Loss
DEL00048768	chr10	14,643,680	14,643,745	65	Loss
DEL00048770	chr10	14,753,237	14,753,342	105	Loss
DEL00048776	chr10	14,930,174	14,930,372	198	Loss
DEL00048786	chr10	15,384,373	15,384,430	57	Loss
DEL00048791	chr10	15,537,972	15,538,396	424	Loss
DEL00048794	chr10	15,665,511	15,665,628	117	Loss
DEL00048797	chr10	15,755,366	15,755,541	175	Loss
DUP00048798	chr10	15,780,898	15,781,019	121	Gain
DEL00048801	chr10	15,850,504	15,850,599	95	Loss
DEL00048806	chr10	16,067,159	16,067,346	187	Loss
DEL00048809	chr10	16,088,871	16,095,577	6,706	Loss
DEL00048815	chr10	16,216,684	16,216,804	120	Loss
DEL00048830	chr10	16,595,910	16,596,315	405	Loss
DEL00048843	chr10	16,712,869	16,712,979	110	Loss
DEL00048847	chr10	16,888,016	16,888,068	52	Loss

DEL00048857	chr10	17,104,907	17,105,726	819	Loss
DEL00048862	chr10	17,209,404	17,209,873	469	Loss
DEL00048871	chr10	17,425,539	17,426,804	1,265	Loss
DEL00048873	chr10	17,620,262	17,620,450	188	Loss
DEL00048881	chr10	17,929,273	17,929,555	282	Loss
DEL00048891	chr10	18,161,331	18,161,397	66	Loss
DEL00048893	chr10	18,196,589	18,196,647	58	Loss
DUP00048895	chr10	18,208,632	18,219,955	11,323	Gain
DEL00048898	chr10	18,299,063	18,299,928	865	Loss
DEL00048899	chr10	18,300,491	18,300,938	447	Loss
DEL00048900	chr10	18,301,909	18,302,051	142	Loss
DEL00048901	chr10	18,319,845	18,319,914	69	Loss
DEL00048902	chr10	18,322,028	18,322,275	247	Loss
DEL00048911	chr10	18,430,242	18,430,495	253	Loss
DEL00048912	chr10	18,481,301	18,481,353	52	Loss
DEL00048921	chr10	18,748,759	18,748,841	82	Loss
DEL00048923	chr10	18,767,200	18,767,255	55	Loss
DEL00048924	chr10	18,779,773	18,779,841	68	Loss
DEL00048926	chr10	18,862,521	18,862,788	267	Loss
DEL00048928	chr10	18,886,563	18,886,724	161	Loss
DEL00048930	chr10	19,009,139	19,009,367	228	Loss
DEL00048943	chr10	19,146,492	19,146,554	62	Loss
DEL00048944	chr10	19,178,537	19,179,301	764	Loss
DEL00048948	chr10	19,252,908	19,253,100	192	Loss
DEL00048956	chr10	19,378,024	19,378,092	68	Loss
DEL00048960	chr10	19,422,917	19,423,263	346	Loss
DEL00048963	chr10	19,444,534	19,445,135	601	Loss
DEL00048966	chr10	19,568,496	19,568,613	117	Loss
DEL00048968	chr10	19,575,898	19,575,962	64	Loss
DUP00048977	chr10	19,769,089	19,775,563	6,474	Mixed
DEL00048980	chr10	20,030,836	20,030,923	87	Loss
DEL00049032	chr11	90,537	91,300	763	Loss
DEL00049034	chr11	154,152	154,203	51	Loss
DEL00049042	chr11	380,538	381,108	570	Loss
DEL00049053	chr11	686,616	686,668	52	Loss
DEL00049062	chr11	1,018,705	1,019,675	970	Loss
DEL00049064	chr11	1,040,097	1,040,287	190	Loss
DEL00049067	chr11	1,066,243	1,066,360	117	Loss
DEL00049070	chr11	1,179,878	1,180,261	383	Loss
DEL00049071	chr11	1,181,048	1,182,073	1,025	Loss
DEL00049072	chr11	1,188,461	1,188,515	54	Loss
DEL00049092	chr11	1,315,970	1,316,215	245	Loss
DUP00049104	chr11	1,563,493	1,563,701	208	Gain
DUP00049107	chr11	1,589,333	1,589,592	259	Gain
DEL00049116	chr11	1,752,940	1,753,526	586	Loss
DEL00049121	chr11	1,757,362	1,757,832	470	Loss
DEL00049125	chr11	1,932,654	1,932,706	52	Loss
DEL00049130	chr11	1,969,051	1,969,233	182	Loss
DEL00049132	chr11	1,991,148	1,991,210	62	Loss

DEL00049141	chr11	2,291,279	2,291,437	158	Loss
DEL00049147	chr11	2,319,810	2,320,075	265	Loss
DUP00049148	chr11	2,352,626	2,352,690	64	Gain
DEL00049150	chr11	2,391,165	2,391,218	53	Loss
DEL00049155	chr11	2,466,764	2,467,324	560	Loss
DEL00049174	chr11	3,491,156	3,491,792	636	Loss
DEL00049175	chr11	3,704,112	3,704,208	96	Loss
DEL00049197	chr11	4,577,158	4,577,219	61	Loss
DEL00049199	chr11	4,661,644	4,662,119	475	Loss
DEL00049201	chr11	4,696,398	4,696,458	60	Loss
DEL00049203	chr11	4,758,514	4,758,794	280	Loss
DEL00049204	chr11	4,776,377	4,776,690	313	Loss
DEL00049215	chr11	5,135,104	5,136,886	1,782	Loss
DEL00049219	chr11	5,254,292	5,254,384	92	Loss
DEL00049229	chr11	5,420,888	5,420,948	60	Loss
DEL00049234	chr11	5,504,688	5,505,130	442	Loss
DEL00049262	chr11	5,985,642	5,985,769	127	Loss
DEL00049264	chr11	6,003,298	6,003,355	57	Loss
DEL00049265	chr11	6,120,101	6,120,207	106	Loss
DEL00049267	chr11	6,143,321	6,143,389	68	Loss
DEL00049269	chr11	6,174,543	6,174,638	95	Loss
DEL00049276	chr11	6,229,281	6,229,487	206	Loss
DEL00049278	chr11	6,269,982	6,270,556	574	Loss
DEL00049279	chr11	6,271,235	6,271,780	545	Loss
DEL00049282	chr11	6,295,688	6,295,950	262	Loss
DEL00049286	chr11	6,366,010	6,366,095	85	Loss
DEL00049290	chr11	6,442,208	6,442,804	596	Loss
DEL00049305	chr11	6,790,159	6,790,474	315	Loss
DEL00049315	chr11	6,855,336	6,855,467	131	Loss
DEL00049321	chr11	6,967,388	6,967,592	204	Loss
DEL00049322	chr11	6,971,632	6,971,685	53	Loss
DEL00049328	chr11	7,034,547	7,034,602	55	Loss
DEL00049333	chr11	7,064,836	7,065,376	540	Loss
DEL00049336	chr11	7,102,311	7,102,509	198	Loss
DEL00049341	chr11	7,186,237	7,186,413	176	Loss
DEL00049349	chr11	7,413,334	7,413,387	53	Loss
DEL00049350	chr11	7,418,914	7,419,134	220	Loss
DEL00049352	chr11	7,428,225	7,428,317	92	Loss
DEL00049353	chr11	7,442,989	7,443,163	174	Loss
DEL00049354	chr11	7,471,395	7,471,465	70	Loss
DEL00049355	chr11	7,483,006	7,483,156	150	Loss
DEL00049360	chr11	7,593,783	7,593,861	78	Loss
DEL00049368	chr11	7,686,742	7,686,927	185	Loss
DEL00049371	chr11	7,742,642	7,742,696	54	Loss
DEL00049372	chr11	7,744,930	7,745,005	75	Loss
DEL00049373	chr11	7,750,822	7,751,058	236	Loss
DEL00049377	chr11	7,784,328	7,784,413	85	Loss
DEL00049380	chr11	7,830,907	7,830,966	59	Loss
DEL00049384	chr11	8,056,683	8,056,892	209	Loss

DEL00049385	chr11	8,104,115	8,104,201	86	Loss
DEL00049393	chr11	8,208,212	8,208,680	468	Loss
DEL00049397	chr11	8,272,676	8,272,741	65	Loss
DEL00049403	chr11	8,395,370	8,395,608	238	Loss
DEL00049404	chr11	8,485,934	8,486,509	575	Loss
DEL00049405	chr11	8,491,654	8,491,965	311	Loss
DEL00049409	chr11	8,535,286	8,536,859	1,573	Loss
DEL00049420	chr11	8,847,661	8,847,780	119	Loss
DEL00049421	chr11	8,849,745	8,849,899	154	Loss
DEL00049425	chr11	8,977,717	8,977,788	71	Loss
DEL00049431	chr11	9,001,528	9,001,631	103	Loss
DUP00049439	chr11	9,186,521	9,186,588	67	Gain
DEL00049441	chr11	9,258,119	9,258,824	705	Loss
DEL00049442	chr11	9,258,840	9,258,894	54	Loss
DEL00049451	chr11	9,412,988	9,413,613	625	Loss
DEL00049453	chr11	9,476,851	9,476,937	86	Loss
DEL00049457	chr11	9,515,750	9,515,853	103	Loss
DEL00049458	chr11	9,596,512	9,596,698	186	Loss
DEL00049459	chr11	9,610,810	9,611,139	329	Loss
DEL00049461	chr11	9,618,967	9,619,296	329	Loss
DEL00049465	chr11	9,700,575	9,701,142	567	Loss
DEL00049477	chr11	10,039,977	10,040,819	842	Loss
DEL00049483	chr11	10,220,180	10,220,534	354	Loss
DEL00049484	chr11	10,228,155	10,228,236	81	Loss
DEL00049485	chr11	10,281,237	10,281,312	75	Loss
DEL00049491	chr11	10,359,107	10,359,189	82	Loss
DEL00049496	chr11	10,419,992	10,420,623	631	Loss
DEL00049498	chr11	10,555,735	10,556,492	757	Loss
DEL00049512	chr11	10,878,595	10,878,706	111	Loss
DEL00049513	chr11	10,902,740	10,902,806	66	Loss
DEL00049530	chr11	10,956,397	10,957,500	1,103	Loss
DEL00049533	chr11	10,996,623	10,996,797	174	Loss
DEL00049535	chr11	11,007,256	11,007,325	69	Loss
DEL00049545	chr11	11,139,951	11,140,660	709	Loss
DEL00049552	chr11	11,331,363	11,331,473	110	Loss
DEL00049554	chr11	11,366,599	11,367,108	509	Loss
DEL00049555	chr11	11,369,579	11,369,650	71	Loss
DEL00049556	chr11	11,373,304	11,373,805	501	Loss
DEL00049559	chr11	11,443,370	11,443,440	70	Loss
DEL00049560	chr11	11,445,235	11,445,341	106	Loss
DEL00049572	chr11	11,674,278	11,674,495	217	Loss
DEL00049586	chr11	12,006,596	12,007,444	848	Loss
DEL00049592	chr11	12,071,527	12,071,823	296	Loss
DEL00049597	chr11	12,181,399	12,181,487	88	Loss
DEL00049598	chr11	12,202,692	12,208,285	5,593	Loss
DEL00049599	chr11	12,225,371	12,225,496	125	Loss
DEL00049602	chr11	12,242,691	12,242,823	132	Loss
DEL00049609	chr11	12,291,547	12,291,618	71	Loss
DEL00049614	chr11	12,320,064	12,320,358	294	Loss

DEL00049617	chr11	12,352,984	12,353,045	61	Loss
DEL00049619	chr11	12,395,896	12,397,489	1,593	Loss
DEL00049621	chr11	12,494,656	12,494,784	128	Loss
DEL00049641	chr11	12,873,653	12,873,772	119	Loss
DEL00049651	chr11	13,113,956	13,114,951	995	Loss
DEL00049652	chr11	13,155,525	13,156,386	861	Loss
DUP00049658	chr11	13,320,649	13,320,744	95	Gain
DEL00049659	chr11	13,324,159	13,324,379	220	Loss
DEL00049662	chr11	13,351,741	13,366,217	14,476	Loss
DEL00049669	chr11	13,547,056	13,547,175	119	Loss
DEL00049677	chr11	13,770,359	13,770,418	59	Loss
DEL00049678	chr11	13,774,034	13,774,195	161	Loss
DEL00049683	chr11	13,820,409	13,820,633	224	Loss
DEL00049688	chr11	13,990,489	13,990,917	428	Loss
DEL00049692	chr11	14,145,584	14,145,784	200	Loss
DEL00049696	chr11	14,208,911	14,209,759	848	Loss
DEL00049699	chr11	14,244,168	14,244,958	790	Loss
DEL00049703	chr11	14,439,693	14,439,850	157	Loss
DEL00049705	chr11	14,548,774	14,549,196	422	Loss
DEL00049709	chr11	14,568,967	14,569,123	156	Loss
DEL00049721	chr11	14,832,253	14,832,387	134	Loss
DEL00049730	chr11	15,182,230	15,182,340	110	Loss
DEL00049733	chr11	15,263,296	15,263,377	81	Loss
DEL00049754	chr11	16,036,643	16,036,736	93	Loss
DEL00049756	chr11	16,129,789	16,130,268	479	Loss
DEL00049761	chr11	16,235,401	16,235,662	261	Loss
DEL00049773	chr11	16,464,272	16,464,397	125	Loss
DEL00049774	chr11	16,533,252	16,533,458	206	Loss
DEL00049777	chr11	16,584,281	16,584,336	55	Loss
DEL00049790	chr11	16,758,129	16,758,207	78	Loss
DUP00049796	chr11	16,947,245	16,963,739	16,494	Gain
DEL00049808	chr11	17,236,173	17,236,315	142	Loss
DEL00049809	chr11	17,237,318	17,237,693	375	Loss
DEL00049824	chr11	17,475,857	17,476,038	181	Loss
DEL00049843	chr11	17,804,454	17,804,506	52	Loss
DEL00049846	chr11	17,857,107	17,857,206	99	Loss
DUP00049860	chr11	18,160,400	18,160,695	295	Gain
DEL00049867	chr11	18,198,600	18,199,088	488	Mixed
DEL00049886	chr11	18,608,539	18,608,724	185	Loss
DEL00049892	chr11	18,863,491	18,863,636	145	Loss
DEL00049908	chr11	19,670,672	19,671,708	1,036	Loss
DEL00049936	chr12	320,033	320,097	64	Loss
DEL00049948	chr12	340,399	341,259	860	Loss
DEL00049967	chr12	458,762	463,827	5,065	Loss
DEL00049985	chr12	714,456	714,716	260	Loss
DEL00049986	chr12	719,252	719,654	402	Loss
DEL00049988	chr12	740,361	741,677	1,316	Loss
DEL00049989	chr12	745,502	745,557	55	Loss
DEL00049995	chr12	798,868	802,305	3,437	Loss

DEL00049999	chr12	869,978	870,275	297	Loss
DEL00050005	chr12	936,695	936,844	149	Loss
DEL00050010	chr12	995,844	995,989	145	Loss
DEL00050016	chr12	1,117,562	1,117,889	327	Loss
DEL00050018	chr12	1,171,849	1,172,032	183	Loss
DEL00050020	chr12	1,211,503	1,211,629	126	Loss
DEL00050034	chr12	1,276,483	1,276,889	406	Loss
DEL00050076	chr12	1,605,486	1,605,859	373	Loss
DEL00050080	chr12	1,608,332	1,608,402	70	Loss
DEL00050082	chr12	1,611,794	1,611,894	100	Loss
DEL00050083	chr12	1,635,220	1,635,274	54	Loss
DEL00050087	chr12	1,735,364	1,735,421	57	Loss
DEL00050115	chr12	1,878,177	1,878,273	96	Loss
DEL00050129	chr12	1,996,690	1,997,041	351	Loss
DEL00050130	chr12	2,031,767	2,032,407	640	Loss
DEL00050142	chr12	2,260,215	2,260,269	54	Loss
DEL00050145	chr12	2,279,902	2,280,100	198	Loss
DUP00050152	chr12	2,330,923	2,331,295	372	Gain
DEL00050171	chr12	2,400,274	2,400,358	84	Loss
DEL00050174	chr12	2,434,151	2,448,024	13,873	Loss
DEL00050179	chr12	2,452,554	2,452,684	130	Loss
DEL00050184	chr12	2,614,886	2,615,047	161	Loss
DEL00050189	chr12	2,641,182	2,641,781	599	Loss
DEL00050191	chr12	2,642,386	2,647,488	5,102	Loss
DEL00050216	chr12	2,758,921	2,759,022	101	Loss
DEL00050231	chr12	3,060,855	3,062,508	1,653	Loss
DEL00050240	chr12	3,241,723	3,242,229	506	Loss
DEL00050242	chr12	3,258,984	3,259,071	87	Loss
DEL00050245	chr12	3,280,487	3,281,513	1,026	Loss
DEL00050249	chr12	3,318,485	3,318,590	105	Loss
DEL00050255	chr12	3,352,239	3,361,730	9,491	Loss
DEL00050270	chr12	3,485,813	3,485,886	73	Loss
DEL00050282	chr12	3,743,558	3,743,619	61	Loss
DEL00050292	chr12	3,988,602	3,988,662	60	Loss
DEL00050300	chr12	4,110,282	4,110,508	226	Loss
DEL00050304	chr12	4,262,974	4,263,035	61	Loss
DEL00050318	chr12	4,432,734	4,432,812	78	Loss
DUP00050321	chr12	4,495,768	4,495,886	118	Gain
DEL00050322	chr12	4,541,147	4,541,331	184	Loss
DEL00050324	chr12	4,591,545	4,592,074	529	Loss
DEL00050328	chr12	4,631,978	4,632,096	118	Loss
DEL00050329	chr12	4,633,697	4,633,759	62	Loss
DEL00050333	chr12	4,672,770	4,673,491	721	Loss
DEL00050334	chr12	4,680,435	4,680,695	260	Loss
DEL00050338	chr12	4,703,144	4,704,643	1,499	Loss
DEL00050345	chr12	4,806,386	4,806,456	70	Loss
DEL00050346	chr12	4,822,183	4,824,098	1,915	Loss
DEL00050349	chr12	4,910,759	4,910,914	155	Loss
DEL00050350	chr12	4,925,769	4,925,824	55	Loss

DEL00050351	chr12	4,941,853	4,942,014	161	Loss
DEL00050352	chr12	4,999,917	5,000,008	91	Loss
DEL00050356	chr12	5,023,196	5,023,718	522	Loss
DEL00050365	chr12	5,169,716	5,169,781	65	Loss
DEL00050370	chr12	5,245,944	5,247,310	1,366	Loss
DEL00050371	chr12	5,257,847	5,258,110	263	Loss
DEL00050375	chr12	5,377,284	5,377,480	196	Loss
DEL00050378	chr12	5,386,347	5,386,641	294	Loss
DEL00050388	chr12	5,601,023	5,601,213	190	Loss
DEL00050390	chr12	5,607,115	5,607,289	174	Loss
DUP00050392	chr12	5,622,773	5,622,872	99	Gain
DEL00050398	chr12	5,715,540	5,715,716	176	Loss
DEL00050402	chr12	5,759,682	5,759,805	123	Loss
DEL00050404	chr12	5,761,670	5,762,178	508	Loss
DEL00050405	chr12	5,763,941	5,764,678	737	Loss
DEL00050406	chr12	5,794,556	5,796,022	1,466	Loss
DEL00050411	chr12	5,896,163	5,896,224	61	Loss
DEL00050413	chr12	6,000,910	6,001,179	269	Loss
DEL00050415	chr12	6,026,918	6,027,476	558	Loss
DEL00050416	chr12	6,036,258	6,036,455	197	Loss
DEL00050421	chr12	6,065,136	6,066,041	905	Loss
DEL00050426	chr12	6,146,868	6,147,929	1,061	Loss
DEL00050427	chr12	6,149,874	6,150,064	190	Loss
DEL00050433	chr12	6,251,531	6,251,811	280	Loss
DEL00050434	chr12	6,261,032	6,261,204	172	Loss
DEL00050440	chr12	6,368,981	6,369,036	55	Loss
DEL00050446	chr12	6,456,210	6,456,324	114	Loss
DEL00050461	chr12	6,692,361	6,692,526	165	Loss
DEL00050462	chr12	6,695,696	6,695,749	53	Loss
DEL00050463	chr12	6,706,226	6,706,282	56	Loss
DEL00050465	chr12	6,758,808	6,759,024	216	Loss
DEL00050472	chr12	6,817,167	6,817,480	313	Loss
DEL00050487	chr12	7,059,080	7,059,454	374	Loss
DEL00050491	chr12	7,234,889	7,234,954	65	Loss
DEL00050493	chr12	7,275,253	7,275,318	65	Loss
DEL00050503	chr12	7,392,383	7,392,669	286	Loss
DEL00050504	chr12	7,410,995	7,411,110	115	Loss
DEL00050506	chr12	7,478,380	7,478,505	125	Loss
DEL00050508	chr12	7,548,734	7,549,848	1,114	Loss
DEL00050509	chr12	7,592,139	7,592,241	102	Loss
DEL00050512	chr12	7,638,814	7,639,079	265	Loss
DEL00050513	chr12	7,654,111	7,654,292	181	Loss
DEL00050524	chr12	7,790,864	7,791,411	547	Loss
DEL00050527	chr12	7,831,912	7,833,429	1,517	Loss
DEL00050531	chr12	7,889,473	7,890,047	574	Loss
DEL00050537	chr12	7,964,632	7,964,800	168	Loss
DEL00050539	chr12	7,973,624	7,973,681	57	Loss
DEL00050550	chr12	8,064,557	8,064,948	391	Loss
DEL00050561	chr12	8,219,216	8,219,300	84	Loss

DEL00050565	chr12	8,269,220	8,269,346	126	Loss
DEL00050567	chr12	8,319,508	8,319,596	88	Loss
DEL00050569	chr12	8,378,962	8,379,266	304	Loss
DEL00050570	chr12	8,385,192	8,387,523	2,331	Loss
DEL00050581	chr12	8,534,702	8,535,361	659	Loss
DEL00050582	chr12	8,537,325	8,537,747	422	Loss
DEL00050588	chr12	8,596,074	8,596,157	83	Loss
DEL00050589	chr12	8,596,185	8,596,268	83	Loss
DUP00050595	chr12	8,721,149	8,721,214	65	Gain
DEL00050598	chr12	8,751,068	8,751,155	87	Loss
DEL00050599	chr12	8,759,551	8,759,753	202	Loss
DEL00050608	chr12	8,829,128	8,829,727	599	Loss
DEL00050609	chr12	8,836,109	8,836,182	73	Loss
DEL00050610	chr12	8,863,495	8,863,636	141	Loss
DEL00050616	chr12	8,897,352	8,897,749	397	Loss
DEL00050619	chr12	8,966,834	8,967,358	524	Loss
DEL00050621	chr12	8,991,713	8,992,090	377	Loss
DEL00050623	chr12	9,021,698	9,022,287	589	Loss
DEL00050628	chr12	9,068,417	9,068,482	65	Loss
DEL00050637	chr12	9,202,014	9,202,147	133	Loss
DEL00050644	chr12	9,260,108	9,260,183	75	Loss
DEL00050649	chr12	9,341,172	9,341,980	808	Loss
DEL00050655	chr12	9,395,204	9,395,265	61	Loss
DEL00050662	chr12	9,467,358	9,467,455	97	Loss
DEL00050687	chr12	10,283,312	10,283,373	61	Loss
DEL00050688	chr12	10,295,311	10,299,740	4,429	Loss
DEL00050689	chr12	10,300,844	10,302,270	1,426	Loss
DEL00050692	chr12	10,386,249	10,386,442	193	Loss
DEL00050695	chr12	10,522,252	10,522,311	59	Loss
DEL00050699	chr12	10,683,477	10,683,636	159	Loss
DEL00050709	chr12	10,959,025	10,959,304	279	Loss
DEL00050715	chr12	11,043,955	11,044,031	76	Loss
DEL00050717	chr12	11,109,208	11,109,714	506	Loss
DEL00050724	chr12	11,159,500	11,159,785	285	Loss
DUP00050725	chr12	11,179,851	11,199,013	19,162	Gain
DEL00050736	chr12	11,473,705	11,473,834	129	Loss
DEL00050737	chr12	11,492,216	11,492,351	135	Loss
DEL00050738	chr12	11,500,594	11,500,658	64	Loss
DEL00050742	chr12	11,617,359	11,617,452	93	Loss
DEL00050746	chr12	11,694,975	11,695,074	99	Loss
DEL00050755	chr12	11,769,365	11,769,509	144	Loss
DEL00050764	chr12	11,913,170	11,914,409	1,239	Loss
DEL00050781	chr12	12,230,562	12,230,970	408	Loss
DEL00050804	chr12	12,820,638	12,820,842	204	Loss
DEL00050813	chr12	13,009,144	13,009,955	811	Loss
DEL00050816	chr12	13,162,914	13,163,621	707	Loss
DEL00050827	chr12	13,286,728	13,286,779	51	Loss
DEL00050829	chr12	13,344,488	13,344,709	221	Loss
DEL00050832	chr12	13,357,586	13,357,656	70	Loss

DEL00050850	chr12	14,039,748	14,040,217	469	Loss
DEL00050856	chr12	14,086,762	14,086,962	200	Loss
DUP00050861	chr12	14,208,529	14,208,618	89	Gain
DEL00050869	chr12	14,530,853	14,530,913	60	Loss
DEL00050879	chr12	14,651,240	14,651,757	517	Loss
DEL00050886	chr12	14,975,427	14,975,479	52	Loss
DEL00050887	chr12	15,006,635	15,006,938	303	Loss
DEL00050892	chr12	15,341,470	15,341,847	377	Loss
DEL00050893	chr12	15,367,772	15,368,059	287	Loss
DEL00050895	chr12	15,375,459	15,376,418	959	Loss
DEL00050901	chr12	15,474,560	15,474,619	59	Loss
DEL00050909	chr12	15,717,466	15,717,596	130	Loss
DEL00050917	chr12	16,034,496	16,034,982	486	Loss
DEL00050918	chr12	16,053,161	16,053,222	61	Loss
DEL00050920	chr12	16,219,149	16,219,217	68	Loss
DEL00050921	chr12	16,234,643	16,234,836	193	Loss
DEL00050924	chr12	16,391,537	16,391,860	323	Loss
DEL00050925	chr12	16,427,051	16,427,110	59	Loss
DEL00050929	chr12	16,524,156	16,524,231	75	Loss
DEL00050937	chr12	16,797,610	16,797,832	222	Loss
DEL00050962	chr12	17,537,696	17,537,792	96	Loss
DEL00050964	chr12	17,834,101	17,834,648	547	Loss
DEL00050965	chr12	17,886,180	17,886,336	156	Loss
DEL00050971	chr12	18,029,613	18,034,402	4,789	Loss
DEL00050975	chr12	18,128,042	18,128,109	67	Loss
DEL00050976	chr12	18,193,414	18,193,472	58	Loss
DEL00050979	chr12	18,300,166	18,300,229	63	Loss
DEL00050993	chr12	18,509,718	18,509,878	160	Loss
DEL00050994	chr12	18,620,827	18,620,878	51	Loss
DEL00051002	chr12	18,713,554	18,713,615	61	Loss
DEL00051007	chr12	18,910,556	18,910,608	52	Loss
DEL00051009	chr12	19,019,225	19,019,286	61	Loss
DEL00051022	chr12	19,606,624	19,606,676	52	Loss
DEL00051027	chr12	19,622,708	19,622,801	93	Loss
DEL00051032	chr12	19,777,009	19,777,166	157	Loss
DEL00051037	chr12	19,797,212	19,797,382	170	Loss
DEL00051046	chr12	20,016,535	20,016,708	173	Loss
DEL00051054	chr12	20,415,612	20,416,616	1,004	Mixed
DEL00051062	chr12	20,416,620	20,417,487	867	Loss
DEL00051100	chr13	689,714	689,896	182	Loss
DEL00051109	chr13	1,111,394	1,111,616	222	Loss
DEL00051114	chr13	1,204,097	1,204,323	226	Loss
DEL00051115	chr13	1,205,305	1,206,091	786	Loss
DEL00051151	chr13	1,430,895	1,476,315	45,420	Loss
DEL00051199	chr13	1,516,490	1,529,200	12,710	Loss
DEL00051204	chr13	1,639,932	1,640,070	138	Loss
DEL00051275	chr13	1,691,576	1,742,334	50,758	Loss
DUP00051386	chr13	1,774,485	1,795,729	21,244	Gain
DUP00051474	chr13	1,886,204	1,932,302	46,098	Gain

DEL00051518	chr13	1,950,796	1,976,916	26,120	Loss
DEL00051556	chr13	2,576,504	2,576,922	418	Loss
DEL00051564	chr13	2,826,260	2,827,789	1,529	Loss
DEL00051575	chr13	2,879,382	2,879,570	188	Loss
DEL00051577	chr13	2,915,797	2,915,871	74	Loss
DEL00051581	chr13	3,101,243	3,101,406	163	Loss
DUP00051593	chr13	3,178,389	3,178,466	77	Gain
DEL00051599	chr13	3,198,175	3,198,657	482	Loss
DEL00051602	chr13	3,232,571	3,232,780	209	Loss
DEL00051606	chr13	3,303,220	3,304,060	840	Loss
DEL00051615	chr13	3,396,354	3,396,801	447	Loss
DEL00051619	chr13	3,472,615	3,472,681	66	Loss
DEL00051623	chr13	3,514,130	3,514,379	249	Loss
DEL00051625	chr13	3,542,124	3,542,910	786	Loss
DEL00051633	chr13	3,601,138	3,602,042	904	Loss
DEL00051644	chr13	3,698,140	3,698,221	81	Loss
DEL00051645	chr13	3,701,951	3,702,080	129	Loss
DEL00051649	chr13	3,755,270	3,755,572	302	Loss
DUP00051656	chr13	3,844,893	3,849,975	5,082	Gain
DEL00051658	chr13	3,876,669	3,877,306	637	Loss
DEL00051660	chr13	3,895,656	3,895,790	134	Loss
DEL00051661	chr13	3,933,345	3,933,396	51	Loss
DEL00051662	chr13	3,978,004	3,978,732	728	Loss
DEL00051671	chr13	4,186,252	4,186,342	90	Loss
DEL00051675	chr13	4,230,979	4,231,242	263	Loss
DEL00051685	chr13	4,328,414	4,328,584	170	Loss
DEL00051686	chr13	4,341,610	4,341,768	158	Loss
DEL00051688	chr13	4,382,958	4,383,826	868	Loss
DEL00051689	chr13	4,386,351	4,386,954	603	Loss
DEL00051699	chr13	4,560,531	4,560,796	265	Loss
DEL00051710	chr13	4,715,437	4,715,651	214	Loss
DEL00051712	chr13	4,753,345	4,753,408	63	Loss
DEL00051715	chr13	4,900,419	4,901,079	660	Loss
DEL00051722	chr13	4,974,223	4,974,291	68	Loss
DEL00051723	chr13	5,043,978	5,044,035	57	Loss
DEL00051727	chr13	5,137,853	5,138,267	414	Loss
DEL00051730	chr13	5,184,888	5,185,311	423	Loss
DEL00051735	chr13	5,269,048	5,269,132	84	Loss
DEL00051738	chr13	5,300,146	5,301,191	1,045	Loss
DEL00051739	chr13	5,301,889	5,302,541	652	Loss
DEL00051741	chr13	5,363,158	5,363,716	558	Loss
DEL00051745	chr13	5,390,421	5,390,973	552	Loss
DEL00051746	chr13	5,393,556	5,393,622	66	Loss
DEL00051749	chr13	5,463,474	5,463,632	158	Loss
DEL00051763	chr13	5,576,872	5,576,951	79	Loss
DEL00051766	chr13	5,623,320	5,623,372	52	Loss
DEL00051767	chr13	5,653,685	5,653,742	57	Loss
DEL00051768	chr13	5,674,203	5,675,217	1,014	Loss
DEL00051769	chr13	5,681,022	5,681,092	70	Loss

DEL00051779	chr13	5,837,658	5,837,710	52	Loss
DEL00051783	chr13	5,924,248	5,925,095	847	Loss
DEL00051788	chr13	5,977,018	5,977,209	191	Loss
DEL00051789	chr13	5,996,398	5,996,519	121	Loss
DEL00051793	chr13	6,066,848	6,066,923	75	Loss
DEL00051796	chr13	6,093,677	6,093,735	58	Loss
DEL00051802	chr13	6,141,987	6,142,248	261	Loss
DEL00051810	chr13	6,295,249	6,295,326	77	Loss
DEL00051817	chr13	6,325,105	6,325,231	126	Loss
DEL00051825	chr13	6,389,347	6,389,653	306	Loss
DEL00051841	chr13	6,614,459	6,614,520	61	Loss
DEL00051847	chr13	6,735,115	6,735,218	103	Loss
DEL00051850	chr13	6,812,850	6,812,929	79	Loss
DEL00051872	chr13	7,189,687	7,189,757	70	Loss
DEL00051873	chr13	7,192,549	7,192,860	311	Loss
DEL00051874	chr13	7,202,577	7,202,740	163	Loss
DEL00051877	chr13	7,239,653	7,239,730	77	Loss
DEL00051879	chr13	7,242,003	7,242,087	84	Loss
DEL00051881	chr13	7,272,717	7,274,737	2,020	Loss
DEL00051887	chr13	7,302,771	7,302,832	61	Loss
DEL00051895	chr13	7,323,541	7,324,314	773	Loss
DEL00051896	chr13	7,338,122	7,338,210	88	Loss
DEL00051898	chr13	7,384,967	7,385,077	110	Loss
DEL00051904	chr13	7,441,174	7,441,251	77	Loss
DEL00051908	chr13	7,487,202	7,497,476	10,274	Loss
DEL00051909	chr13	7,538,517	7,538,674	157	Loss
DUP00051915	chr13	7,632,702	7,633,128	426	Mixed
DEL00051922	chr13	7,731,971	7,733,335	1,364	Loss
DEL00051936	chr13	7,894,722	7,894,983	261	Loss
DEL00051941	chr13	8,017,498	8,017,641	143	Loss
DEL00051943	chr13	8,049,661	8,050,387	726	Loss
DEL00051947	chr13	8,145,928	8,146,391	463	Loss
DEL00051948	chr13	8,239,300	8,239,419	119	Loss
DEL00051952	chr13	8,300,075	8,300,285	210	Loss
DEL00051961	chr13	8,430,201	8,430,261	60	Loss
DEL00051980	chr13	8,749,748	8,750,210	462	Loss
DEL00051983	chr13	8,762,180	8,762,242	62	Loss
DEL00051993	chr13	9,010,196	9,010,257	61	Loss
DEL00052002	chr13	9,111,264	9,111,668	404	Loss
DEL00052003	chr13	9,114,095	9,114,194	99	Loss
DEL00052006	chr13	9,347,787	9,347,885	98	Loss
DEL00052013	chr13	9,633,559	9,634,335	776	Loss
DEL00052016	chr13	9,669,648	9,669,923	275	Loss
DEL00052017	chr13	9,675,294	9,675,501	207	Loss
DEL00052032	chr13	10,090,025	10,090,076	51	Loss
DEL00052036	chr13	10,251,499	10,251,616	117	Loss
DEL00052042	chr13	10,316,609	10,317,717	1,108	Loss
DEL00052057	chr13	10,688,497	10,688,552	55	Loss
DEL00052063	chr13	10,771,126	10,771,488	362	Loss

DEL00052066	chr13	10,947,376	10,947,689	313	Loss
DEL00052069	chr13	11,033,643	11,033,700	57	Loss
DEL00052070	chr13	11,056,217	11,056,407	190	Loss
DEL00052071	chr13	11,089,286	11,089,409	123	Loss
DEL00052073	chr13	11,230,662	11,230,731	69	Loss
DEL00052082	chr13	11,347,363	11,347,542	179	Loss
DEL00052083	chr13	11,366,651	11,366,793	142	Loss
DEL00052084	chr13	11,386,590	11,386,771	181	Loss
DEL00052087	chr13	11,466,690	11,467,439	749	Loss
DEL00052095	chr13	11,633,405	11,633,605	200	Loss
DEL00052096	chr13	11,658,190	11,658,368	178	Loss
DEL00052098	chr13	11,697,336	11,697,484	148	Loss
DEL00052109	chr13	11,821,960	11,822,041	81	Loss
DEL00052113	chr13	11,921,876	11,922,128	252	Loss
DEL00052116	chr13	11,951,115	11,951,168	53	Loss
DEL00052117	chr13	12,058,203	12,058,294	91	Loss
DEL00052118	chr13	12,074,441	12,074,909	468	Loss
DEL00052119	chr13	12,108,447	12,108,871	424	Loss
DEL00052139	chr13	12,785,443	12,786,062	619	Loss
DEL00052158	chr13	13,572,364	13,572,443	79	Loss
DEL00052167	chr13	13,816,923	13,817,464	541	Loss
DEL00052170	chr13	13,993,726	13,993,786	60	Loss
DEL00052175	chr13	14,084,749	14,086,195	1,446	Loss
DEL00052177	chr13	14,219,828	14,220,992	1,164	Loss
DEL00052178	chr13	14,251,639	14,252,199	560	Loss
DEL00052184	chr13	14,389,572	14,389,864	292	Loss
DEL00052185	chr13	14,392,735	14,393,480	745	Loss
DEL00052188	chr13	14,489,026	14,489,588	562	Loss
DEL00052195	chr13	14,653,557	14,653,750	193	Loss
DUP00052204	chr13	15,076,368	15,076,484	116	Gain
DEL00052212	chr13	15,297,874	15,298,438	564	Loss
DEL00052235	chr13	15,512,559	15,512,898	339	Loss
DEL00052237	chr13	15,524,225	15,541,909	17,684	Loss
DEL00052243	chr13	15,578,700	15,578,753	53	Loss
DEL00052249	chr13	15,687,068	15,687,136	68	Loss
DEL00052251	chr13	15,710,264	15,710,334	70	Loss
DEL00052258	chr13	15,842,429	15,842,771	342	Loss
DEL00052260	chr13	15,904,158	15,904,281	123	Loss
DEL00052262	chr13	15,930,602	15,930,824	222	Loss
DEL00052267	chr13	16,077,243	16,077,295	52	Loss
DEL00052272	chr13	16,155,418	16,155,666	248	Loss
DEL00052285	chr13	16,588,895	16,588,953	58	Loss
DEL00052292	chr13	16,738,105	16,739,672	1,567	Loss
DEL00052298	chr13	16,801,868	16,802,001	133	Loss
DEL00052300	chr13	16,824,207	16,824,296	89	Loss
DEL00052315	chr13	17,056,371	17,056,514	143	Loss
DEL00052319	chr13	17,131,119	17,131,521	402	Loss
DEL00052321	chr13	17,188,964	17,189,029	65	Loss
DEL00052323	chr13	17,228,861	17,228,996	135	Loss

DEL00052326	chr13	17,415,668	17,415,725	57	Loss
DUP00052334	chr13	17,572,747	17,572,805	58	Gain
DEL00052337	chr13	17,718,378	17,719,711	1,333	Loss
DEL00052348	chr13	17,939,202	17,939,657	455	Loss
DEL00052391	chr14	27,449	29,030	1,581	Loss
DUP00052398	chr14	29,162	30,004	842	Gain
DEL00052411	chr14	259,247	259,739	492	Loss
DEL00052412	chr14	286,318	286,583	265	Loss
DEL00052413	chr14	330,217	330,865	648	Loss
DEL00052427	chr14	769,261	769,936	675	Loss
DEL00052431	chr14	828,689	828,784	95	Loss
DEL00052433	chr14	890,232	890,346	114	Loss
DEL00052436	chr14	938,697	938,760	63	Loss
DEL00052440	chr14	999,912	1,000,012	100	Loss
DEL00052442	chr14	1,065,566	1,065,859	293	Loss
DEL00052450	chr14	1,363,164	1,363,240	76	Loss
DEL00052451	chr14	1,385,892	1,386,023	131	Loss
DEL00052457	chr14	1,396,785	1,397,091	306	Loss
DUP00052461	chr14	1,484,186	1,484,249	63	Gain
DEL00052463	chr14	1,540,452	1,541,034	582	Loss
DEL00052465	chr14	1,573,984	1,574,532	548	Loss
DEL00052472	chr14	1,707,039	1,707,122	83	Loss
DEL00052478	chr14	1,864,786	1,865,949	1,163	Loss
DEL00052480	chr14	2,128,082	2,128,144	62	Loss
DEL00052489	chr14	2,303,562	2,303,922	360	Loss
DEL00052508	chr14	2,684,669	2,684,921	252	Loss
DEL00052510	chr14	2,754,031	2,772,906	18,875	Loss
DEL00052512	chr14	2,779,182	2,781,840	2,658	Loss
DEL00052513	chr14	2,861,157	2,861,209	52	Loss
DEL00052515	chr14	2,897,281	2,897,335	54	Loss
DEL00052522	chr14	3,051,649	3,052,190	541	Loss
DEL00052524	chr14	3,083,679	3,083,807	128	Loss
DEL00052527	chr14	3,269,430	3,269,507	77	Loss
DEL00052543	chr14	3,479,452	3,481,434	1,982	Loss
DEL00052545	chr14	3,549,095	3,549,412	317	Loss
DEL00052546	chr14	3,573,092	3,573,368	276	Loss
DEL00052564	chr14	3,947,900	3,948,012	112	Loss
DEL00052567	chr14	4,151,735	4,151,929	194	Loss
DEL00052572	chr14	4,177,486	4,177,558	72	Loss
DEL00052573	chr14	4,233,044	4,233,607	563	Loss
DEL00052576	chr14	4,289,006	4,289,249	243	Loss
DEL00052579	chr14	4,327,771	4,327,834	63	Loss
DEL00052589	chr14	4,624,521	4,624,585	64	Loss
DEL00052601	chr14	5,118,722	5,118,857	135	Loss
DEL00052605	chr14	5,205,414	5,205,734	320	Loss
DEL00052607	chr14	5,267,053	5,267,584	531	Loss
DEL00052609	chr14	5,318,399	5,318,829	430	Loss
DEL00052618	chr14	5,536,413	5,536,468	55	Loss
DEL00052620	chr14	5,548,080	5,548,667	587	Loss

DEL00052630	chr14	6,070,337	6,070,601	264	Loss
DEL00052637	chr14	6,237,237	6,237,304	67	Loss
DEL00052683	chr14	6,547,232	6,549,066	1,834	Mixed
DEL00052717	chr14	6,549,634	6,550,885	1,251	Mixed
DEL00052729	chr14	6,613,002	6,613,075	73	Loss
DEL00052730	chr14	6,616,786	6,616,935	149	Loss
DEL00052731	chr14	6,723,564	6,723,626	62	Loss
DEL00052752	chr14	7,205,422	7,205,528	106	Loss
DEL00052756	chr14	7,289,393	7,289,450	57	Loss
DEL00052757	chr14	7,335,202	7,335,257	55	Loss
DEL00052765	chr14	7,471,201	7,471,322	121	Loss
DUP00052774	chr14	7,484,951	7,486,139	1,188	Gain
DEL00052788	chr14	7,543,808	7,544,426	618	Loss
DEL00052798	chr14	7,913,386	7,914,498	1,112	Loss
DEL00052801	chr14	8,064,240	8,064,429	189	Loss
DEL00052807	chr14	8,153,391	8,153,703	312	Loss
DEL00052809	chr14	8,179,456	8,179,926	470	Loss
DEL00052816	chr14	8,287,652	8,287,714	62	Loss
DEL00052819	chr14	8,390,314	8,390,575	261	Loss
DEL00052824	chr14	8,504,771	8,505,004	233	Loss
DEL00052829	chr14	8,585,050	8,585,692	642	Loss
DEL00052830	chr14	8,645,664	8,645,776	112	Loss
DEL00052836	chr14	8,753,699	8,753,762	63	Loss
DEL00052844	chr14	8,867,549	8,868,532	983	Loss
DEL00052846	chr14	8,971,912	8,971,965	53	Loss
DEL00052848	chr14	8,991,581	8,991,901	320	Loss
DEL00052850	chr14	9,012,420	9,012,509	89	Loss
DEL00052855	chr14	9,083,563	9,085,655	2,092	Loss
DEL00052856	chr14	9,113,724	9,113,786	62	Loss
DEL00052858	chr14	9,132,628	9,132,866	238	Loss
DEL00052860	chr14	9,148,746	9,148,970	224	Loss
DEL00052861	chr14	9,158,481	9,159,328	847	Loss
DEL00052862	chr14	9,169,160	9,169,591	431	Loss
DEL00052864	chr14	9,222,307	9,222,377	70	Loss
DEL00052870	chr14	9,484,432	9,484,916	484	Loss
DEL00052871	chr14	9,485,969	9,486,424	455	Loss
DEL00052873	chr14	9,496,807	9,496,879	72	Loss
DEL00052877	chr14	9,615,735	9,616,109	374	Loss
DEL00052880	chr14	9,649,221	9,649,279	58	Loss
DEL00052882	chr14	9,674,473	9,674,539	66	Loss
DEL00052883	chr14	9,693,267	9,693,346	79	Loss
DEL00052887	chr14	9,902,940	9,903,506	566	Loss
DEL00052896	chr14	10,201,034	10,201,277	243	Loss
DEL00052901	chr14	10,472,452	10,472,503	51	Loss
DEL00052909	chr14	10,608,120	10,608,284	164	Loss
DEL00052917	chr14	10,771,683	10,771,755	72	Loss
DEL00052920	chr14	10,801,899	10,805,696	3,797	Loss
DEL00052921	chr14	10,850,891	10,850,955	64	Loss
DEL00052922	chr14	10,887,603	10,887,654	51	Loss

DEL00052935	chr14	11,103,211	11,104,046	835	Loss
DEL00052939	chr14	11,176,457	11,176,522	65	Loss
DEL00052946	chr14	11,399,399	11,399,577	178	Loss
DEL00052949	chr14	11,417,570	11,417,676	106	Loss
DEL00052951	chr14	11,511,447	11,511,740	293	Loss
DEL00052956	chr14	11,600,323	11,600,847	524	Loss
DEL00052957	chr14	11,613,323	11,613,504	181	Loss
DEL00052964	chr14	11,703,141	11,703,309	168	Loss
DEL00052969	chr14	11,872,553	11,872,624	71	Loss
DEL00052980	chr14	12,116,380	12,116,441	61	Loss
DEL00052990	chr14	12,299,829	12,299,881	52	Loss
DEL00052996	chr14	12,404,037	12,404,096	59	Loss
DEL00053002	chr14	12,481,353	12,481,470	117	Loss
DUP00053018	chr14	13,000,074	13,000,417	343	Gain
DEL00053028	chr14	13,152,804	13,152,963	159	Loss
DEL00053032	chr14	13,251,287	13,251,553	266	Loss
DUP00053036	chr14	13,252,441	13,252,682	241	Gain
DEL00053069	chr14	13,787,227	13,787,278	51	Loss
DEL00053070	chr14	13,811,018	13,812,167	1,149	Loss
DEL00053076	chr14	13,834,553	13,834,861	308	Loss
DEL00053080	chr14	13,845,386	13,845,456	70	Loss
DEL00053081	chr14	13,853,475	13,853,612	137	Loss
DEL00053083	chr14	13,856,341	13,858,213	1,872	Loss
DEL00053091	chr14	13,930,894	13,931,091	197	Loss
DEL00053093	chr14	13,964,377	13,964,640	263	Loss
DEL00053095	chr14	14,030,926	14,031,015	89	Loss
DEL00053097	chr14	14,036,047	14,036,597	550	Loss
DEL00053103	chr14	14,056,319	14,056,518	199	Loss
DEL00053112	chr14	14,125,776	14,125,835	59	Loss
DEL00053121	chr14	14,257,186	14,257,237	51	Loss
DEL00053127	chr14	14,349,330	14,349,561	231	Loss
DEL00053135	chr14	14,381,510	14,382,089	579	Loss
DEL00053142	chr14	14,452,312	14,454,559	2,247	Loss
DEL00053149	chr14	14,518,900	14,519,434	534	Loss
DEL00053155	chr14	14,602,570	14,602,949	379	Loss
DEL00053174	chr14	14,791,478	14,792,077	599	Loss
DEL00053176	chr14	14,798,947	14,799,591	644	Loss
DEL00053178	chr14	14,813,509	14,819,342	5,833	Loss
DUP00053191	chr14	14,959,845	14,959,951	106	Gain
DEL00053204	chr14	15,202,057	15,202,977	920	Loss
DUP00053208	chr14	15,327,004	15,337,672	10,668	Mixed
DEL00053321	chr15	21,320	21,379	59	Loss
DEL00053325	chr15	22,342	22,454	112	Loss
DEL00053328	chr15	22,767	23,021	254	Loss
DUP00053339	chr15	24,041	29,517	5,476	Mixed
DEL00053383	chr15	356,243	356,763	520	Loss
DUP00053386	chr15	472,242	472,311	69	Gain
DEL00053391	chr15	578,338	578,465	127	Loss
DEL00053398	chr15	960,266	960,354	88	Loss

DEL00053408	chr15	1,088,143	1,091,385	3,242	Loss
DEL00053412	chr15	1,163,234	1,163,968	734	Loss
DEL00053413	chr15	1,197,430	1,197,562	132	Loss
DEL00053415	chr15	1,275,534	1,277,250	1,716	Loss
DEL00053419	chr15	1,434,235	1,434,295	60	Loss
DEL00053423	chr15	1,808,126	1,808,294	168	Loss
DEL00053427	chr15	1,921,231	1,921,301	70	Loss
DEL00053428	chr15	1,963,735	1,963,813	78	Loss
DEL00053438	chr15	2,167,620	2,168,208	588	Loss
DEL00053441	chr15	2,234,303	2,234,846	543	Loss
DEL00053448	chr15	2,410,095	2,410,581	486	Loss
DUP00053450	chr15	2,527,749	2,542,138	14,389	Gain
DEL00053471	chr15	2,824,937	2,825,145	208	Loss
DEL00053476	chr15	2,994,264	2,994,864	600	Loss
DEL00053478	chr15	3,046,824	3,047,827	1,003	Loss
DEL00053479	chr15	3,072,706	3,075,367	2,661	Loss
DEL00053482	chr15	3,308,022	3,308,186	164	Loss
DEL00053487	chr15	3,535,337	3,535,503	166	Loss
DUP00053503	chr15	3,780,702	3,780,830	128	Gain
DEL00053509	chr15	4,086,380	4,086,495	115	Loss
DEL00053517	chr15	4,510,722	4,510,799	77	Loss
DEL00053541	chr15	4,860,797	4,862,676	1,879	Loss
DEL00053556	chr15	4,975,558	4,976,145	587	Loss
DEL00053558	chr15	5,036,609	5,037,061	452	Loss
DEL00053565	chr15	5,328,105	5,328,271	166	Loss
DEL00053566	chr15	5,404,693	5,404,839	146	Loss
DEL00053613	chr15	6,457,480	6,457,531	51	Loss
DEL00053619	chr15	6,543,343	6,544,265	922	Loss
DEL00053621	chr15	6,740,461	6,740,528	67	Loss
DUP00053652	chr15	7,337,809	7,348,648	10,839	Gain
DEL00053663	chr15	7,442,800	7,442,853	53	Loss
DEL00053670	chr15	7,555,262	7,555,332	70	Loss
DEL00053671	chr15	7,572,956	7,573,177	221	Loss
DEL00053677	chr15	7,654,915	7,655,125	210	Loss
DEL00053678	chr15	7,665,544	7,665,723	179	Loss
DEL00053688	chr15	7,719,980	7,720,197	217	Loss
DEL00053689	chr15	7,722,937	7,723,035	98	Loss
DEL00053728	chr15	8,033,941	8,034,040	99	Loss
DEL00053732	chr15	8,182,166	8,182,262	96	Loss
DEL00053738	chr15	8,591,533	8,591,620	87	Loss
DEL00053750	chr15	8,727,897	8,727,965	68	Loss
DEL00053754	chr15	8,807,730	8,807,902	172	Loss
DEL00053755	chr15	8,821,500	8,822,280	780	Loss
DEL00053769	chr15	9,074,889	9,075,308	419	Loss
DEL00053776	chr15	9,351,776	9,351,827	51	Loss
DEL00053779	chr15	9,360,896	9,372,145	11,249	Loss
DEL00053785	chr15	9,376,073	9,376,196	123	Loss
DEL00053786	chr15	9,407,611	9,407,697	86	Loss
DUP00053807	chr15	9,907,780	9,908,132	352	Gain

DUP00053810	chr15	10,015,606	10,026,627	11,021	Mixed
DEL00053816	chr15	10,132,526	10,132,613	87	Loss
DEL00053823	chr15	10,305,669	10,306,054	385	Loss
DEL00053848	chr15	10,571,032	10,571,157	125	Loss
DEL00053849	chr15	10,606,919	10,607,011	92	Loss
DEL00053871	chr15	10,966,842	10,967,330	488	Loss
DEL00053888	chr15	11,389,769	11,390,056	287	Loss
DEL00053904	chr15	11,665,643	11,668,345	2,702	Loss
DEL00053906	chr15	11,730,668	11,730,730	62	Loss
DEL00053908	chr15	11,813,451	11,814,484	1,033	Loss
DEL00053914	chr15	11,851,873	11,852,040	167	Loss
DEL00053920	chr15	12,031,995	12,032,060	65	Loss
DEL00053926	chr15	12,169,073	12,169,141	68	Loss
DEL00053930	chr15	12,244,414	12,244,677	263	Loss
DUP00054265	chr16	449,719	991,794	542,075	Mixed
DEL00054880	chr16	1,048,122	1,048,585	463	Loss
DEL00054926	chr16	1,196,385	1,197,246	861	Loss
DEL00054927	chr16	1,201,753	1,201,826	73	Loss
DUP00054936	chr16	1,221,270	1,222,026	756	Gain
DEL00054966	chr16	1,231,705	1,232,110	405	Loss
DUP00054969	chr16	1,232,181	1,235,153	2,972	Gain
DEL00054989	chr16	1,245,283	1,245,346	63	Loss
DUP00055049	chr16	1,314,838	1,381,552	66,714	Gain
DUP00055231	chr16	1,480,577	1,481,184	607	Gain
DUP00055246	chr16	1,586,928	1,587,009	81	Gain
DEL00055265	chr17	33,080	33,371	291	Loss
DEL00055266	chr17	34,285	35,000	715	Loss
DUP00055283	chr17	199,775	199,885	110	Gain
DEL00055293	chr17	341,572	342,337	765	Loss
DEL00055311	chr17	790,001	790,574	573	Loss
DEL00055314	chr17	798,954	799,012	58	Loss
DEL00055321	chr17	841,957	842,189	232	Loss
DEL00055322	chr17	872,938	872,990	52	Loss
DEL00055324	chr17	907,803	907,910	107	Loss
DEL00055337	chr17	1,089,728	1,090,067	339	Loss
DEL00055341	chr17	1,093,071	1,093,139	68	Loss
DEL00055345	chr17	1,133,116	1,133,167	51	Loss
DEL00055346	chr17	1,140,911	1,141,305	394	Loss
DEL00055371	chr17	1,639,446	1,639,583	137	Loss
DEL00055375	chr17	1,667,879	1,668,401	522	Loss
DEL00055383	chr17	1,777,272	1,778,027	755	Loss
DEL00055384	chr17	1,849,045	1,849,098	53	Loss
DEL00055394	chr17	2,144,419	2,144,618	199	Loss
DEL00055413	chr17	2,378,364	2,378,437	73	Loss
DEL00055416	chr17	2,465,083	2,466,239	1,156	Loss
DEL00055428	chr17	2,560,442	2,560,874	432	Loss
DEL00055429	chr17	2,644,678	2,644,799	121	Loss
DEL00055455	chr17	3,525,542	3,525,595	53	Loss
DEL00055458	chr17	3,680,671	3,680,747	76	Loss

DEL00055470	chr17	4,150,135	4,155,031	4,896	Loss
DEL00055472	chr17	4,187,888	4,187,946	58	Loss
DEL00055477	chr17	4,219,535	4,219,699	164	Loss
DEL00055480	chr17	4,241,596	4,241,678	82	Loss
DEL00055504	chr17	4,835,195	4,835,410	215	Loss
DEL00055510	chr17	5,076,061	5,076,234	173	Loss
DEL00055513	chr17	5,147,030	5,147,132	102	Loss
DEL00055523	chr17	5,431,891	5,432,374	483	Loss
DEL00055531	chr17	5,599,328	5,599,517	189	Loss
DEL00055536	chr17	5,765,418	5,765,540	122	Loss
DEL00055537	chr17	5,788,606	5,788,771	165	Loss
DEL00055539	chr17	5,833,133	5,833,191	58	Loss
DUP00055551	chr17	6,172,785	6,173,081	296	Gain
DEL00055564	chr17	6,516,476	6,516,620	144	Loss
DEL00055573	chr17	6,709,376	6,709,927	551	Loss
DEL00055576	chr17	6,738,279	6,738,425	146	Loss
DUP00055583	chr17	6,976,711	6,976,840	129	Gain
DEL00055587	chr17	7,060,718	7,060,769	51	Loss
DEL00055592	chr17	7,097,139	7,097,265	126	Loss
DEL00055595	chr17	7,341,027	7,341,095	68	Loss
DEL00055597	chr17	7,449,053	7,449,124	71	Loss
DEL00055598	chr17	7,504,329	7,504,438	109	Loss
DEL00055606	chr17	7,751,113	7,751,226	113	Loss
DEL00055613	chr17	8,035,361	8,035,946	585	Loss
DUP00055616	chr17	8,094,253	8,094,313	60	Gain
DEL00055620	chr17	8,202,020	8,202,460	440	Loss
DEL00055628	chr17	8,359,609	8,359,684	75	Loss
DEL00055635	chr17	8,474,438	8,474,977	539	Loss
DEL00055649	chr17	9,027,831	9,027,891	60	Loss
DEL00055664	chr17	9,311,523	9,311,684	161	Loss
DEL00055666	chr17	9,366,384	9,366,713	329	Loss
DEL00055686	chr17	10,168,474	10,172,029	3,555	Mixed
DEL00055719	chr17	10,228,908	10,229,675	767	Loss
DUP00055749	chr18	466,334	468,539	2,205	Gain
DEL00055764	chr18	469,793	471,960	2,167	Loss
DUP00055774	chr18	472,153	472,746	593	Gain
DUP00055777	chr18	473,183	475,555	2,372	Gain
DEL00055793	chr18	767,541	768,586	1,045	Loss
DEL00055804	chr18	903,558	908,243	4,685	Loss
DEL00055838	chr18	1,036,616	1,036,941	325	Loss
DEL00055840	chr18	1,063,761	1,063,818	57	Loss
DEL00055853	chr18	1,153,400	1,153,645	245	Loss
DEL00055862	chr18	1,204,917	1,205,132	215	Loss
DEL00055863	chr18	1,235,934	1,236,176	242	Loss
DEL00055864	chr18	1,255,266	1,255,326	60	Loss
DEL00055865	chr18	1,261,679	1,261,840	161	Loss
DEL00055869	chr18	1,373,629	1,373,683	54	Loss
DEL00055870	chr18	1,379,923	1,381,703	1,780	Loss
DEL00055871	chr18	1,385,083	1,385,425	342	Loss

DEL00055872	chr18	1,432,998	1,433,502	504	Loss
DEL00055884	chr18	1,532,764	1,532,875	111	Loss
DEL00055890	chr18	1,625,375	1,625,454	79	Loss
DEL00055896	chr18	1,718,031	1,718,303	272	Loss
DEL00055898	chr18	1,745,814	1,745,880	66	Loss
DEL00055910	chr18	1,833,615	1,834,303	688	Loss
DEL00055931	chr18	2,064,845	2,065,245	400	Loss
DEL00056026	chr18	2,303,735	2,339,506	35,771	Mixed
DUP00056365	chr18	2,394,840	2,406,218	11,378	Gain
DEL00056409	chr18	2,855,680	2,855,750	70	Loss
DEL00056414	chr18	2,927,033	2,927,222	189	Loss
DEL00056425	chr18	3,165,089	3,165,231	142	Loss
DEL00056430	chr18	3,193,718	3,196,951	3,233	Loss
DEL00056446	chr18	3,341,346	3,341,720	374	Loss
DEL00056458	chr18	3,678,522	3,678,837	315	Loss
DEL00056473	chr18	3,902,868	3,902,926	58	Loss
DEL00056475	chr18	3,957,707	3,958,153	446	Loss
DEL00056488	chr18	4,174,944	4,175,029	85	Loss
DEL00056492	chr18	4,249,067	4,249,497	430	Loss
DEL00056502	chr18	4,395,557	4,395,671	114	Loss
DEL00056508	chr18	4,481,925	4,482,030	105	Loss
DEL00056509	chr18	4,499,942	4,500,041	99	Loss
DEL00056519	chr18	4,652,075	4,652,149	74	Loss
DEL00056522	chr18	4,681,517	4,682,792	1,275	Loss
DEL00056536	chr18	5,140,090	5,140,141	51	Loss
DEL00056538	chr18	5,242,331	5,242,401	70	Loss
DEL00056553	chr18	5,532,876	5,532,939	63	Loss
DEL00056558	chr18	5,572,646	5,572,725	79	Loss
DEL00056561	chr18	5,920,832	5,920,981	149	Loss
DEL00056567	chr18	6,032,871	6,033,028	157	Loss
DEL00056571	chr18	6,160,533	6,161,051	518	Loss
DEL00056572	chr18	6,194,993	6,195,075	82	Loss
DEL00056576	chr18	6,256,069	6,256,194	125	Loss
DEL00056578	chr18	6,280,343	6,280,394	51	Loss
DEL00056580	chr18	6,295,267	6,295,326	59	Loss
DEL00056585	chr18	6,327,642	6,328,764	1,122	Loss
DEL00056586	chr18	6,350,026	6,351,356	1,330	Loss
DEL00056587	chr18	6,393,809	6,394,229	420	Loss
DEL00056596	chr18	6,526,633	6,526,755	122	Loss
DEL00056597	chr18	6,605,075	6,605,192	117	Loss
DEL00056619	chr18	7,235,707	7,235,769	62	Loss
DEL00056624	chr18	7,257,104	7,257,367	263	Loss
DUP00056625	chr18	7,284,722	7,303,508	18,786	Mixed
DEL00056640	chr18	7,881,086	7,881,254	168	Loss
DEL00056642	chr18	8,000,412	8,001,400	988	Loss
DEL00056651	chr18	8,152,819	8,152,921	102	Loss
DEL00056657	chr18	8,395,324	8,395,488	164	Loss
DEL00056659	chr18	8,447,113	8,447,186	73	Loss
DUP00056676	chr18	8,766,751	8,766,884	133	Gain

DEL00056681	chr18	8,902,142	8,902,889	747	Loss
DEL00056694	chr18	9,310,705	9,310,766	61	Loss
DEL00056697	chr18	9,407,335	9,407,846	511	Loss
DEL00056704	chr18	9,450,341	9,450,405	64	Loss
DEL00056707	chr18	9,456,443	9,456,557	114	Loss
DEL00056725	chr18	9,822,619	9,822,671	52	Loss
DEL00056746	chr18	10,344,727	10,344,827	100	Loss
DEL00056753	chr18	10,549,897	10,549,989	92	Loss
DEL00056757	chr18	10,667,101	10,667,194	93	Loss
DEL00056758	chr18	10,683,934	10,683,997	63	Loss
DEL00056762	chr18	10,817,575	10,817,628	53	Loss
DEL00056766	chr18	10,987,591	10,987,647	56	Loss
DEL00056775	chr18	11,425,686	11,427,181	1,495	Mixed
DEL00056781	chr19	230,021	230,086	65	Loss
DUP00056792	chr19	279,373	289,629	10,256	Mixed
DUP00056810	chr19	289,901	297,061	7,160	Mixed
DUP00056825	chr19	300,251	305,115	4,864	Mixed
DEL00056833	chr19	307,602	307,664	62	Loss
DEL00056843	chr19	478,767	480,696	1,929	Loss
DEL00056849	chr19	517,355	517,625	270	Loss
DEL00056871	chr19	649,915	650,075	160	Loss
DEL00056899	chr19	1,154,069	1,154,622	553	Loss
DEL00056904	chr19	1,291,455	1,291,734	279	Loss
DEL00056909	chr19	1,313,037	1,313,100	63	Loss
DEL00056918	chr19	1,421,157	1,421,592	435	Loss
DEL00056926	chr19	1,493,167	1,494,074	907	Loss
DEL00056931	chr19	1,554,764	1,554,977	213	Loss
DEL00056940	chr19	1,705,710	1,705,857	147	Loss
DEL00056943	chr19	1,767,395	1,767,934	539	Loss
DEL00056950	chr19	1,870,634	1,873,761	3,127	Loss
DEL00056956	chr19	2,018,447	2,018,675	228	Loss
DEL00056958	chr19	2,042,212	2,042,489	277	Loss
DEL00056964	chr19	2,085,550	2,085,681	131	Loss
DEL00056965	chr19	2,152,748	2,152,940	192	Loss
DEL00056971	chr19	2,254,906	2,254,957	51	Loss
DEL00056972	chr19	2,257,574	2,257,650	76	Loss
DEL00056980	chr19	2,347,831	2,347,990	159	Loss
DEL00056982	chr19	2,392,930	2,401,047	8,117	Loss
DEL00056983	chr19	2,408,608	2,408,679	71	Loss
DEL00056985	chr19	2,474,851	2,480,684	5,833	Loss
DEL00056989	chr19	2,486,786	2,486,840	54	Loss
DEL00056995	chr19	2,549,858	2,549,933	75	Loss
DEL00056998	chr19	2,623,224	2,623,466	242	Loss
DEL00057001	chr19	2,638,727	2,638,801	74	Loss
DEL00057006	chr19	2,726,107	2,726,175	68	Loss
DEL00057008	chr19	2,843,583	2,844,317	734	Loss
DEL00057016	chr19	2,881,392	2,881,636	244	Loss
DEL00057019	chr19	2,898,811	2,899,006	195	Loss
DUP00057021	chr19	2,903,935	2,916,700	12,765	Mixed

DEL00057025	chr19	2,984,462	2,984,589	127	Loss
DEL00057029	chr19	3,034,337	3,034,390	53	Loss
DEL00057051	chr19	3,341,786	3,341,938	152	Loss
DEL00057063	chr19	3,613,380	3,613,651	271	Loss
DEL00057067	chr19	3,755,094	3,755,655	561	Loss
DEL00057072	chr19	3,873,623	3,873,757	134	Loss
DEL00057078	chr19	4,002,904	4,002,956	52	Loss
DEL00057083	chr19	4,096,026	4,096,106	80	Loss
DEL00057093	chr19	4,255,332	4,255,744	412	Loss
DEL00057101	chr19	4,373,595	4,373,665	70	Loss
DEL00057108	chr19	4,620,391	4,620,864	473	Loss
DEL00057109	chr19	4,635,175	4,635,237	62	Loss
DEL00057144	chr19	5,521,808	5,522,002	194	Loss
DEL00057146	chr19	5,581,654	5,581,739	85	Loss
DEL00057154	chr19	5,741,450	5,741,514	64	Loss
DEL00057159	chr19	5,916,340	5,916,426	86	Loss
DEL00057168	chr19	6,146,245	6,146,302	57	Loss
DEL00057176	chr19	6,348,359	6,348,517	158	Loss
DEL00057185	chr19	6,632,624	6,632,754	130	Loss
DEL00057207	chr19	7,696,218	7,696,275	57	Loss
DEL00057208	chr19	7,823,353	7,823,548	195	Loss
DUP00057209	chr19	7,853,986	7,854,043	57	Gain
DEL00057210	chr19	7,909,373	7,909,426	53	Loss
DEL00057216	chr19	8,010,018	8,010,190	172	Loss
DEL00057219	chr19	8,095,109	8,095,246	137	Loss
DEL00057224	chr19	8,262,952	8,263,006	54	Loss
DEL00057227	chr19	8,311,771	8,312,134	363	Loss
DEL00057240	chr19	8,895,759	8,895,811	52	Loss
DEL00057250	chr19	9,237,887	9,237,950	63	Loss
DEL00057281	chr19	10,397,148	10,397,563	415	Loss
DUP00057284	chr20	12,265	13,368	1,103	Gain
DUP00057302	chr20	28,840	28,970	130	Gain
DEL00057304	chr20	34,456	35,755	1,299	Loss
DEL00057307	chr20	37,078	37,129	51	Loss
DEL00057317	chr20	580,653	580,726	73	Loss
DEL00057320	chr20	664,440	664,520	80	Loss
DEL00057321	chr20	667,898	668,125	227	Loss
DEL00057335	chr20	921,306	921,402	96	Loss
DEL00057354	chr20	1,138,790	1,139,290	500	Loss
DEL00057355	chr20	1,162,603	1,162,689	86	Loss
DEL00057356	chr20	1,193,615	1,199,156	5,541	Loss
DEL00057369	chr20	1,424,452	1,426,397	1,945	Loss
DEL00057374	chr20	1,567,723	1,567,882	159	Loss
DEL00057375	chr20	1,588,482	1,594,315	5,833	Loss
DEL00057390	chr20	1,732,403	1,732,559	156	Loss
DEL00057406	chr20	2,051,162	2,051,601	439	Loss
DUP00057408	chr20	2,061,326	2,061,458	132	Gain
DEL00057443	chr20	2,209,339	2,210,067	728	Loss
DEL00057445	chr20	2,223,748	2,224,034	286	Loss

DEL00057446	chr20	2,272,687	2,272,767	80	Loss
DEL00057450	chr20	2,350,882	2,350,964	82	Loss
DEL00057451	chr20	2,370,860	2,371,327	467	Loss
DEL00057476	chr20	2,552,392	2,552,461	69	Loss
DEL00057480	chr20	2,674,898	2,674,999	101	Loss
DEL00057483	chr20	2,832,581	2,832,639	58	Loss
DEL00057485	chr20	2,899,812	2,900,362	550	Loss
DEL00057494	chr20	3,028,034	3,028,428	394	Loss
DEL00057495	chr20	3,033,070	3,033,202	132	Loss
DEL00057497	chr20	3,060,070	3,060,279	209	Loss
DEL00057499	chr20	3,085,374	3,085,566	192	Loss
DEL00057533	chr20	3,311,969	3,312,111	142	Loss
DEL00057540	chr20	3,434,866	3,435,068	202	Loss
DEL00057558	chr20	3,678,545	3,679,025	480	Loss
DEL00057565	chr20	3,760,613	3,760,835	222	Loss
DEL00057566	chr20	3,786,320	3,786,406	86	Loss
DEL00057568	chr20	3,808,380	3,808,537	157	Loss
DEL00057571	chr20	3,891,808	3,891,964	156	Loss
DEL00057572	chr20	3,928,479	3,928,888	409	Loss
DEL00057582	chr20	4,094,748	4,097,503	2,755	Loss
DEL00057590	chr20	4,206,281	4,206,339	58	Loss
DEL00057593	chr20	4,273,489	4,274,090	601	Loss
DEL00057595	chr20	4,281,065	4,283,023	1,958	Loss
DEL00057599	chr20	4,311,040	4,311,172	132	Loss
DEL00057608	chr20	4,412,715	4,413,218	503	Loss
DEL00057615	chr20	4,452,787	4,453,119	332	Loss
DEL00057618	chr20	4,540,211	4,540,304	93	Loss
DEL00057622	chr20	4,669,028	4,669,277	249	Loss
DEL00057642	chr20	5,038,591	5,038,722	131	Loss
DEL00057647	chr20	5,138,697	5,139,769	1,072	Loss
DEL00057657	chr20	5,247,844	5,248,103	259	Loss
DEL00057681	chr20	5,700,841	5,701,258	417	Loss
DEL00057682	chr20	5,712,784	5,712,887	103	Loss
DEL00057684	chr20	5,758,924	5,758,983	59	Loss
DEL00057687	chr20	5,876,950	5,877,366	416	Loss
DEL00057690	chr20	5,945,556	5,946,974	1,418	Loss
DEL00057691	chr20	5,959,871	5,959,991	120	Loss
DEL00057701	chr20	6,146,942	6,156,712	9,770	Loss
DEL00057709	chr20	6,280,130	6,280,211	81	Loss
DEL00057711	chr20	6,500,862	6,500,950	88	Loss
DEL00057713	chr20	6,539,964	6,540,021	57	Loss
DEL00057714	chr20	6,556,214	6,556,273	59	Loss
DEL00057718	chr20	6,579,665	6,579,723	58	Loss
DEL00057719	chr20	6,592,715	6,593,762	1,047	Loss
DEL00057722	chr20	6,611,112	6,611,528	416	Loss
DEL00057729	chr20	6,708,094	6,708,192	98	Loss
DEL00057754	chr20	6,970,709	6,971,216	507	Loss
DEL00057767	chr20	7,396,768	7,396,943	175	Loss
DEL00057768	chr20	7,425,558	7,429,792	4,234	Loss

DEL00057780	chr20	7,614,059	7,614,172	113	Loss
DEL00057785	chr20	7,805,554	7,805,792	238	Loss
DEL00057797	chr20	8,265,042	8,266,087	1,045	Loss
DEL00057798	chr20	8,326,300	8,326,374	74	Loss
DEL00057801	chr20	8,356,478	8,356,560	82	Loss
DEL00057807	chr20	8,484,434	8,484,592	158	Loss
DEL00057819	chr20	8,630,476	8,630,561	85	Loss
DEL00057823	chr20	8,661,574	8,662,225	651	Loss
DEL00057826	chr20	8,755,756	8,755,823	67	Loss
DEL00057828	chr20	8,860,388	8,860,527	139	Loss
DEL00057833	chr20	8,986,817	8,986,882	65	Loss
DEL00057840	chr20	9,180,551	9,180,623	72	Loss
DEL00057843	chr20	9,188,550	9,190,324	1,774	Loss
DEL00057848	chr20	9,284,667	9,284,839	172	Loss
DEL00057853	chr20	9,329,169	9,329,238	69	Loss
DEL00057857	chr20	9,502,952	9,503,056	104	Loss
DUP00057878	chr20	9,901,624	9,901,877	253	Gain
DEL00057896	chr20	10,571,818	10,571,938	120	Loss
DEL00057897	chr20	10,588,736	10,588,803	67	Loss
DEL00057902	chr20	10,836,281	10,836,448	167	Loss
DUP00057903	chr20	10,935,413	10,951,434	16,021	Mixed
DEL00057906	chr20	11,103,013	11,103,520	507	Loss
DEL00057909	chr20	11,176,638	11,176,763	125	Loss
DEL00057918	chr20	11,412,161	11,412,765	604	Loss
DEL00057921	chr20	11,624,800	11,625,109	309	Loss
DEL00057926	chr20	11,707,341	11,707,426	85	Loss
DEL00057934	chr20	11,813,271	11,813,329	58	Loss
DEL00057937	chr20	11,931,891	11,932,044	153	Loss
DEL00057942	chr20	12,121,451	12,121,531	80	Loss
DEL00057943	chr20	12,153,354	12,153,435	81	Loss
DEL00057946	chr20	12,181,566	12,181,653	87	Loss
DEL00057948	chr20	12,233,687	12,233,809	122	Loss
DEL00057951	chr20	12,252,807	12,253,043	236	Loss
DEL00057959	chr20	12,407,483	12,407,601	118	Loss
DEL00057967	chr20	12,566,986	12,567,660	674	Loss
DEL00057968	chr20	12,646,055	12,646,125	70	Loss
DUP00057981	chr20	12,951,894	12,952,156	262	Gain
DEL00057983	chr20	12,966,595	12,966,648	53	Loss
DEL00057984	chr20	12,974,816	12,977,287	2,471	Loss
DEL00057985	chr20	13,015,536	13,015,606	70	Loss
DEL00057986	chr20	13,039,179	13,039,263	84	Loss
DEL00057994	chr20	13,429,199	13,429,289	90	Loss
DEL00058038	chr21	423,814	424,009	195	Loss
DEL00058053	chr21	713,549	713,759	210	Loss
DEL00058059	chr21	778,694	778,816	122	Loss
DEL00058060	chr21	791,930	791,991	61	Loss
DEL00058066	chr21	973,551	974,381	830	Loss
DEL00058087	chr21	1,371,093	1,371,183	90	Loss
DEL00058089	chr21	1,388,419	1,388,591	172	Loss

DEL00058092	chr21	1,804,654	1,804,819	165	Loss
DEL00058094	chr21	1,880,821	1,881,178	357	Loss
DUP00058097	chr21	2,074,556	2,101,830	27,274	Mixed
DEL00058116	chr21	2,559,323	2,559,431	108	Loss
DEL00058117	chr21	2,562,127	2,562,292	165	Loss
DEL00058118	chr21	2,568,255	2,568,328	73	Loss
DEL00058120	chr21	2,611,693	2,611,826	133	Loss
DUP00058133	chr21	2,765,276	2,765,449	173	Gain
DEL00058136	chr21	2,770,321	2,770,399	78	Loss
DEL00058160	chr21	3,159,288	3,159,571	283	Loss
DEL00058167	chr21	3,421,815	3,421,882	67	Loss
DEL00058173	chr21	3,536,050	3,536,124	74	Loss
DEL00058193	chr21	4,068,350	4,068,431	81	Loss
DEL00058202	chr21	4,550,257	4,550,380	123	Loss
DEL00058203	chr21	4,555,490	4,555,616	126	Loss
DUP00058207	chr21	4,599,099	4,599,213	114	Gain
DEL00058213	chr21	4,730,698	4,731,012	314	Loss
DUP00058215	chr21	4,751,835	4,751,975	140	Gain
DEL00058228	chr21	4,839,485	4,839,543	58	Loss
DEL00058230	chr21	4,864,890	4,866,487	1,597	Mixed
DEL00058243	chr21	4,899,611	4,899,681	70	Loss
DEL00058260	chr21	5,023,584	5,023,641	57	Loss
DEL00058282	chr21	5,277,440	5,278,173	733	Loss
DEL00058288	chr21	5,436,656	5,436,712	56	Loss
DEL00058300	chr21	5,559,075	5,559,202	127	Loss
DEL00058303	chr21	5,597,549	5,597,624	75	Loss
DEL00058304	chr21	5,633,348	5,633,435	87	Loss
DEL00058338	chr21	5,999,300	5,999,352	52	Loss
DEL00058341	chr21	6,057,395	6,057,454	59	Loss
DEL00058350	chr21	6,073,550	6,073,688	138	Loss
DEL00058367	chr21	6,255,462	6,255,539	77	Loss
DUP00058385	chr21	6,453,148	6,453,292	144	Gain
DEL00058398	chr21	6,592,087	6,592,155	68	Loss
DEL00058403	chr21	6,657,739	6,658,379	640	Loss
DEL00058406	chr21	6,744,024	6,744,584	560	Loss
DEL00058437	chr22	239,179	240,499	1,320	Loss
DEL00058441	chr22	325,357	326,210	853	Loss
DEL00058450	chr22	636,263	636,338	75	Loss
DEL00058451	chr22	748,439	748,492	53	Loss
DEL00058452	chr22	771,018	771,908	890	Loss
DEL00058463	chr22	1,329,548	1,329,928	380	Loss
DUP00058474	chr22	1,458,023	1,458,571	548	Gain
DEL00058485	chr22	2,192,334	2,192,391	57	Loss
DEL00058551	chr22	3,165,030	3,165,085	55	Loss
DEL00058560	chr22	3,229,043	3,229,463	420	Loss
DUP00058609	chr22	3,300,551	3,300,718	167	Gain
DEL00058613	chr22	3,300,796	3,301,278	482	Loss
DEL00058618	chr22	3,301,445	3,302,080	635	Loss
DUP00058641	chr22	3,303,351	3,303,517	166	Gain

DUP00058660	chr22	3,340,036	3,340,745	709	Gain
DEL00058676	chr22	3,353,684	3,353,887	203	Loss
DEL00058780	chr22	3,456,424	3,457,122	698	Loss
DEL00058887	chr22	3,950,889	3,951,775	886	Loss
DEL00058888	chr22	3,963,307	3,963,426	119	Loss
DEL00058891	chr22	4,092,233	4,093,572	1,339	Loss
DEL00058905	chr22	4,272,148	4,272,633	485	Loss
DEL00058913	chr22	4,335,055	4,339,718	4,663	Loss
DEL00059044	chr22	4,517,986	4,518,197	211	Loss
DEL00059134	chr23	412,947	413,379	432	Loss
DEL00059141	chr23	492,822	493,280	458	Loss
DEL00059145	chr23	537,751	537,824	73	Loss
DEL00059154	chr23	632,879	633,247	368	Loss
DEL00059169	chr23	853,341	853,536	195	Loss
DEL00059170	chr23	921,304	921,437	133	Loss
DEL00059171	chr23	926,182	926,332	150	Loss
DEL00059179	chr23	1,044,174	1,044,634	460	Loss
DEL00059182	chr23	1,119,538	1,119,659	121	Loss
DEL00059192	chr23	1,195,291	1,195,366	75	Loss
DEL00059211	chr23	1,379,997	1,380,059	62	Loss
DEL00059213	chr23	1,411,907	1,411,970	63	Loss
DEL00059216	chr23	1,455,801	1,455,897	96	Loss
DEL00059217	chr23	1,492,751	1,492,929	178	Loss
DEL00059219	chr23	1,508,224	1,508,445	221	Loss
DEL00059220	chr23	1,515,547	1,515,721	174	Loss
DEL00059221	chr23	1,522,482	1,522,580	98	Loss
DEL00059228	chr23	1,616,280	1,616,430	150	Loss
DEL00059237	chr23	1,701,907	1,702,701	794	Loss
DEL00059240	chr23	1,727,405	1,728,524	1,119	Loss
DEL00059246	chr23	1,803,680	1,803,752	72	Loss
DEL00059248	chr23	1,834,755	1,834,831	76	Loss
DEL00059250	chr23	1,892,021	1,892,093	72	Loss
DEL00059292	chr23	2,005,871	2,005,937	66	Loss
DEL00059307	chr23	2,058,405	2,058,498	93	Loss
DEL00059312	chr23	2,065,919	2,066,277	358	Loss
DEL00059314	chr23	2,084,862	2,084,932	70	Loss
DEL00059318	chr23	2,167,459	2,167,533	74	Loss
DEL00059319	chr23	2,194,879	2,194,945	66	Loss
DEL00059329	chr23	2,250,442	2,250,511	69	Loss
DEL00059334	chr23	2,276,693	2,276,745	52	Loss
DEL00059336	chr23	2,277,073	2,277,155	82	Loss
DEL00059341	chr23	2,339,625	2,339,725	100	Loss
DEL00059348	chr23	2,436,351	2,442,715	6,364	Loss
DUP00059361	chr23	2,548,640	2,548,802	162	Gain
DUP00059365	chr23	2,550,245	2,550,321	76	Gain
DEL00059381	chr23	2,920,918	2,921,174	256	Loss
DEL00059384	chr23	3,009,592	3,010,105	513	Loss
DEL00059386	chr23	3,073,913	3,074,033	120	Loss
DEL00059400	chr23	3,261,832	3,261,893	61	Loss

DEL00059404	chr23	3,392,123	3,392,311	188	Loss
DEL00059411	chr23	3,574,485	3,574,647	162	Loss
DEL00059412	chr23	3,602,997	3,603,155	158	Loss
DEL00059418	chr23	3,733,417	3,733,481	64	Loss
DUP00059457	chr23	4,302,504	4,302,678	174	Gain
DEL00059465	chr23	4,423,927	4,424,407	480	Loss
DEL00059466	chr23	4,428,100	4,428,207	107	Loss
DEL00059472	chr23	4,591,792	4,591,934	142	Loss
DEL00059473	chr23	4,667,403	4,667,455	52	Loss
DEL00059485	chr23	4,897,079	4,897,222	143	Loss
DEL00059487	chr23	4,912,972	4,913,252	280	Loss
DEL00059514	chr23	5,339,041	5,339,196	155	Loss
DEL00059515	chr23	5,452,952	5,453,940	988	Loss
DEL00059543	chr24	468,025	468,118	93	Loss
DEL00059545	chr24	529,159	529,218	59	Loss
DEL00059557	chr24	806,897	807,016	119	Loss
DEL00059561	chr24	836,231	836,366	135	Loss
DUP00059564	chr24	932,231	932,395	164	Gain
DEL00059566	chr24	951,737	951,835	98	Loss
DEL00059577	chr24	1,188,744	1,189,317	573	Loss
DEL00059582	chr24	1,321,582	1,327,415	5,833	Loss
DEL00059592	chr24	1,762,088	1,762,168	80	Loss
DEL00059609	chr24	2,275,674	2,281,762	6,088	Loss
DEL00059615	chr24	2,414,139	2,414,199	60	Loss
DEL00059617	chr24	2,519,534	2,519,740	206	Loss
DEL00059621	chr24	2,688,578	2,688,929	351	Loss
DEL00059622	chr24	2,724,844	2,724,929	85	Loss
DEL00059623	chr24	2,766,907	2,767,042	135	Loss
DEL00059631	chr24	2,899,255	2,899,308	53	Loss
DUP00059640	chr24	3,297,279	3,297,374	95	Gain
DEL00059656	chr24	3,681,334	3,681,398	64	Loss
DEL00059665	chr24	3,791,327	3,791,381	54	Loss
DEL00059676	chr24	3,946,871	3,946,970	99	Loss
DEL00059678	chr24	3,976,722	3,976,793	71	Loss
DEL00059679	chr24	4,052,734	4,052,817	83	Loss
DEL00059683	chr24	4,133,406	4,133,484	78	Loss
DEL00059693	chr24	4,263,336	4,263,417	81	Loss
DEL00059694	chr24	4,298,034	4,298,131	97	Loss
DEL00059697	chr24	4,335,662	4,335,798	136	Loss
DEL00059703	chr24	4,446,411	4,446,514	103	Loss
DEL00059708	chr24	4,451,424	4,451,623	199	Loss
DEL00059717	chr24	4,723,605	4,723,674	69	Loss
DEL00059725	chr24	4,846,718	4,846,864	146	Loss
DEL00059732	chr24	4,995,912	4,996,197	285	Loss
DEL00059748	chr24	5,088,056	5,088,245	189	Loss
DEL00059759	chr24	5,099,250	5,099,369	119	Loss
DEL00059762	chr24	5,142,379	5,142,505	126	Loss
DEL00059765	chr24	5,193,047	5,193,175	128	Loss
DEL00059782	chr24	5,323,969	5,324,050	81	Loss

DEL00059887	chr24	5,982,343	5,982,416	73	Loss
DEL00059953	chr25	14,318	14,641	323	Loss
DEL00059982	chr25	29,494	30,156	662	Loss
DUP00059992	chr25	45,338	45,745	407	Gain
DEL00059997	chr25	46,524	47,604	1,080	Mixed
DUP00060021	chr25	83,911	84,434	523	Gain
DUP00060030	chr25	85,483	87,071	1,588	Mixed
DEL00060058	chr25	99,595	99,951	356	Loss
DUP00060103	chr25	156,585	156,893	308	Mixed
DEL00060133	chr25	237,169	237,445	276	Loss
DEL00060147	chr25	274,449	275,802	1,353	Loss
DUP00060170	chr25	369,229	372,550	3,321	Gain
DUP00060193	chr25	438,819	441,244	2,425	Gain
DEL00060205	chr25	443,220	444,938	1,718	Loss
DEL00060211	chr25	450,742	450,794	52	Loss
DEL00060213	chr25	453,076	457,353	4,277	Loss
DUP00060259	chr25	633,762	633,957	195	Gain
DUP00060271	chr25	808,022	811,684	3,662	Gain
DUP00060291	chr25	909,936	910,305	369	Gain
DEL00060299	chr25	990,045	991,074	1,029	Loss
DEL00060361	chr25	1,171,520	1,172,065	545	Loss
DUP00060384	chr25	1,199,744	1,205,614	5,870	Mixed
DEL00060450	chr25	1,365,190	1,365,269	79	Loss
DEL00060453	chr25	1,372,525	1,372,586	61	Loss
DUP00060456	chr25	1,379,236	1,379,723	487	Gain
DUP00060526	chr25	1,457,879	1,458,340	461	Gain
DUP00060550	chr25	1,479,632	1,479,927	295	Gain
DEL00060565	chr25	1,485,658	1,486,150	492	Loss
DUP00060572	chr25	1,520,870	1,521,470	600	Gain
DEL00060586	chr25	1,585,341	1,585,483	142	Loss
DEL00060628	chr25	2,046,213	2,046,270	57	Loss
DEL00060640	chr25	2,078,111	2,078,566	455	Loss
DEL00060644	chr25	2,088,497	2,088,556	59	Loss
DEL00060654	chr25	2,144,381	2,144,771	390	Loss
DEL00060683	chr25	2,338,640	2,349,428	10,788	Loss
DEL00060704	chr26	345,064	345,222	158	Loss
DEL00060707	chr26	392,376	392,443	67	Loss
DEL00060715	chr26	460,815	461,072	257	Loss
DEL00060721	chr26	623,522	623,593	71	Loss
DEL00060726	chr26	705,755	705,845	90	Loss
DEL00060731	chr26	790,141	790,719	578	Loss
DEL00060746	chr26	935,143	935,916	773	Loss
DEL00060748	chr26	1,035,188	1,035,483	295	Loss
DEL00060798	chr26	1,487,335	1,487,467	132	Loss
DEL00060800	chr26	1,519,472	1,519,570	98	Loss
DEL00060835	chr26	1,976,094	1,976,328	234	Loss
DEL00060846	chr26	2,192,294	2,192,736	442	Loss
DEL00060849	chr26	2,215,851	2,215,904	53	Loss
DEL00060850	chr26	2,242,064	2,242,176	112	Loss

DEL00060858	chr26	2,472,179	2,472,316	137	Loss
DEL00060865	chr26	2,613,855	2,613,970	115	Loss
DEL00060876	chr26	2,751,486	2,752,033	547	Loss
DEL00060881	chr26	2,831,352	2,831,430	78	Loss
DEL00060895	chr26	3,017,060	3,017,307	247	Loss
DEL00060916	chr26	3,318,375	3,318,544	169	Loss
DEL00060926	chr26	3,385,759	3,385,810	51	Loss
DEL00060932	chr26	3,425,847	3,425,934	87	Loss
DEL00060944	chr26	3,543,768	3,544,090	322	Loss
DEL00060980	chr26	4,210,511	4,210,955	444	Loss
DEL00060998	chr26	4,673,739	4,673,963	224	Loss
DEL00061018	chr26	4,955,082	4,955,190	108	Loss
DEL00061024	chr26	5,050,613	5,063,657	13,044	Loss
DEL00061052	chr26	5,257,353	5,259,792	2,439	Loss
DUP00061064	chr26	5,265,219	5,266,525	1,306	Gain
DUP00061071	chr26	5,277,546	5,278,147	601	Gain
DEL00061085	chr27	27,863	31,393	3,530	Loss
DEL00061101	chr27	126,197	127,312	1,115	Loss
DEL00061106	chr27	172,165	174,180	2,015	Loss
DEL00061113	chr27	176,106	198,281	22,175	Loss
DEL00061612	chr27	422,822	478,741	55,919	Mixed
DEL00061733	chr27	535,223	535,447	224	Loss
DEL00061857	chr27	585,409	897,118	311,709	Mixed
DEL00062323	chr27	1,380,612	1,380,695	83	Loss
DEL00062327	chr27	1,428,772	1,429,055	283	Loss
DEL00062332	chr27	1,458,062	1,458,443	381	Loss
DEL00062340	chr27	1,547,038	1,547,091	53	Loss
DEL00062345	chr27	1,581,837	1,582,505	668	Loss
DEL00062347	chr27	1,599,561	1,599,725	164	Loss
DEL00062356	chr27	1,673,052	1,673,153	101	Loss
DEL00062363	chr27	1,681,678	1,682,007	329	Loss
DEL00062364	chr27	1,695,490	1,695,683	193	Loss
DEL00062372	chr27	1,746,398	1,746,502	104	Loss
DEL00062373	chr27	1,774,687	1,774,791	104	Loss
DEL00062385	chr27	1,842,862	1,842,917	55	Loss
DEL00062417	chr27	1,884,716	1,885,110	394	Loss
DUP00062431	chr27	1,894,283	1,894,720	437	Gain
DEL00062455	chr27	1,943,821	1,943,949	128	Loss
DEL00062482	chr27	2,064,147	2,064,947	800	Loss
DEL00062486	chr27	2,078,692	2,078,838	146	Loss
DUP00062487	chr27	2,081,989	2,082,216	227	Gain
DEL00062488	chr27	2,111,228	2,111,312	84	Loss
DEL00062491	chr27	2,152,549	2,152,648	99	Loss
DEL00062494	chr27	2,201,858	2,202,385	527	Loss
DEL00062496	chr27	2,240,238	2,240,332	94	Loss
DEL00062500	chr27	2,313,831	2,313,927	96	Loss
DEL00062505	chr27	2,331,535	2,331,624	89	Loss
DEL00062508	chr27	2,439,439	2,440,413	974	Loss
DEL00062517	chr27	2,568,743	2,568,805	62	Loss

DEL00062537	chr27	2,730,631	2,730,958	327	Loss
DEL00062558	chr27	2,844,673	2,844,741	68	Loss
DEL00062562	chr27	2,866,141	2,867,702	1,561	Loss
DEL00062585	chr27	2,959,389	2,959,449	60	Loss
DEL00062594	chr27	3,068,451	3,068,537	86	Loss
DEL00062598	chr27	3,176,104	3,176,164	60	Loss
DEL00062654	chr27	3,461,070	3,461,187	117	Loss
DUP00062655	chr27	3,472,195	3,486,546	14,351	Gain
DEL00062663	chr27	3,523,515	3,523,598	83	Loss
DEL00062668	chr27	3,579,573	3,580,015	442	Loss
DEL00062679	chr27	3,703,012	3,703,195	183	Loss
DEL00062685	chr27	3,822,753	3,822,912	159	Loss
DEL00062691	chr27	3,908,042	3,908,339	297	Loss
DUP00062695	chr27	3,948,809	3,948,901	92	Gain
DEL00062699	chr27	4,060,937	4,061,028	91	Loss
DEL00062710	chr27	4,184,216	4,184,271	55	Loss
DEL00062725	chr27	4,328,984	4,329,050	66	Loss
DEL00062815	chr27	5,774,570	5,774,969	399	Loss
DUP00062820	chr27	5,775,512	5,776,646	1,134	Mixed
DEL00062854	chr27	5,816,715	5,816,946	231	Loss
DEL00062858	chr27	5,828,451	5,828,756	305	Loss
DEL00062867	chr27	5,856,906	5,856,985	79	Loss
DUP00062908	chr27	5,899,515	5,901,756	2,241	Mixed
DEL00062985	chr28	72,629	72,780	151	Loss
DEL00062987	chr28	88,950	91,008	2,058	Loss
DEL00062994	chr28	94,923	95,006	83	Loss
DEL00062999	chr28	103,480	103,596	116	Loss
DEL00063001	chr28	104,027	104,153	126	Loss
DEL00063002	chr28	109,660	109,812	152	Loss
DEL00063071	chr28	380,788	380,851	63	Loss
DEL00063080	chr28	520,609	520,738	129	Loss
DEL00063085	chr28	569,477	569,588	111	Loss
DEL00063092	chr28	746,276	749,654	3,378	Loss
DUP00063107	chr28	751,522	763,936	12,414	Gain
DUP00063209	chr28	1,014,505	1,015,214	709	Gain
DUP00063229	chr28	1,040,762	1,040,932	170	Gain
DEL00063234	chr28	1,047,701	1,048,196	495	Loss
DEL00063246	chr28	1,146,774	1,146,997	223	Loss
DEL00063248	chr28	1,176,379	1,176,444	65	Loss
DEL00063270	chr28	1,462,038	1,462,126	88	Loss
DEL00063275	chr28	1,584,397	1,584,488	91	Loss
DEL00063281	chr28	1,700,493	1,701,581	1,088	Loss
DEL00063288	chr28	1,846,682	1,846,944	262	Loss
DEL00063289	chr28	1,852,470	1,852,534	64	Loss
DUP00063308	chr28	1,951,686	1,951,931	245	Gain
DEL00063383	chr28	3,052,039	3,052,655	616	Loss
DEL00063391	chr28	3,461,965	3,462,155	190	Loss
DUP00063400	chr28	3,653,185	3,653,243	58	Gain
DEL00063426	chr28	3,887,647	3,888,164	517	Loss

DEL00063430	chr28	3,933,372	3,933,510	138	Loss
DEL00063437	chr28	3,995,033	3,995,093	60	Loss
DEL00063451	chr28	4,089,912	4,089,995	83	Loss
DEL00063502	chr28	4,423,933	4,424,029	96	Loss
DEL00063503	chr28	4,430,123	4,430,255	132	Loss
DEL00063509	chr28	4,452,318	4,452,538	220	Loss
DEL00063510	chr28	4,463,662	4,463,714	52	Loss
DEL00063514	chr28	4,465,569	4,465,981	412	Loss
DEL00063516	chr28	4,467,595	4,467,823	228	Loss
DEL00063517	chr28	4,468,180	4,469,156	976	Loss
DEL00063523	chr28	4,508,669	4,509,053	384	Loss
DEL00063538	chr28	4,630,405	4,630,628	223	Loss
DEL00063545	chr28	4,720,813	4,721,368	555	Loss
DEL00063560	chr28	4,932,506	4,932,611	105	Loss
DEL00063642	chr28	5,175,771	5,175,921	150	Loss
DEL00063668	chr28	5,177,980	5,180,222	2,242	Loss
DEL00064138	chr30	92,455	92,574	119	Loss
DUP00064140	chr30	92,724	93,863	1,139	Mixed
DUP00064275	chr30	309,706	310,173	467	Gain
DUP00064293	chr30	362,257	362,565	308	Gain
DEL00064315	chr30	432,443	432,763	320	Loss
DUP00064347	chr30	492,220	492,719	499	Gain
DEL00064354	chr30	493,311	494,271	960	Loss
DEL00064428	chr30	553,892	553,946	54	Loss
DUP00064433	chr30	574,549	574,906	357	Mixed
DUP00064437	chr30	580,838	581,092	254	Gain
DEL00064439	chr30	581,326	581,536	210	Loss
DUP00064446	chr30	622,337	622,894	557	Gain
DEL00064454	chr30	665,789	666,557	768	Loss
DEL00064461	chr30	673,493	673,883	390	Loss
DEL00064470	chr30	689,399	689,481	82	Loss
DEL00064471	chr30	711,468	711,539	71	Loss
DUP00064504	chr30	899,941	900,125	184	Gain
DEL00064526	chr30	942,629	942,995	366	Loss
DEL00064531	chr30	949,634	950,730	1,096	Mixed
DUP00064547	chr30	956,151	956,462	311	Gain
DUP00064549	chr30	972,703	973,121	418	Gain
DEL00064568	chr31	420,550	420,750	200	Loss
DUP00064572	chr31	441,876	442,001	125	Gain
DUP00064668	chr31	598,970	792,135	193,165	Mixed
DEL00065016	chr31	800,983	801,294	311	Loss
DEL00065017	chr31	811,527	811,678	151	Loss
DUP00065021	chr31	823,673	880,148	56,475	Mixed
DEL00065431	chr31	927,196	927,302	106	Loss
DEL00065958	chr31	1,026,458	1,026,516	58	Loss
DEL00066070	chr31	1,131,404	1,131,799	395	Loss
DEL00066080	chr31	1,131,897	1,133,187	1,290	Loss
DEL00066109	chr31	1,152,598	1,152,793	195	Loss
DEL00066304	chr31	1,341,258	1,395,307	54,049	Mixed

DUP00066325	chr31	1,400,045	1,402,510	2,465	Mixed
DEL00066480	chr33	121,262	121,369	107	Loss
DUP00066656	chr33	206,644	206,887	243	Gain
DUP00067066	chr33	622,111	827,872	205,761	Mixed
DUP00067558	chr33	1,084,756	1,101,347	16,591	Mixed
DUP00067650	chr33	1,189,834	1,277,288	87,454	Mixed
DUP00068049	chr33	1,279,506	1,783,896	504,390	Mixed
DEL00071454	chr33	1,942,510	1,981,841	39,331	Loss
DEL00072822	chr33	2,225,075	2,225,543	468	Loss
DEL00073333	chr33	2,362,091	2,362,677	586	Loss
DUP00073694	chr33	2,432,304	2,549,005	116,701	Mixed
DEL00074944	chr33	2,800,731	2,800,804	73	Loss
DEL00075205	chr33	2,873,772	2,873,953	181	Loss
DEL00075709	chr33	3,077,041	3,077,109	68	Loss
DEL00075923	chr33	3,173,496	3,173,589	93	Loss
DEL00076000	chr33	3,207,997	3,208,049	52	Loss
DEL00076059	chr33	3,258,670	3,258,895	225	Loss
DEL00076121	chr33	3,302,202	3,303,876	1,674	Loss
DUP00076158	chr33	3,322,422	3,490,687	168,265	Gain
DEL00076308	chr33	3,513,377	3,513,998	621	Loss
DEL00076429	chr34	35,174	35,829	655	Loss
DUP00076494	chr34	41,814	42,180	366	Gain
DUP00076562	chr34	122,970	123,550	580	Gain
DEL00076615	chr34	660,080	660,421	341	Loss
DUP00076634	chr34	813,745	814,600	855	Gain
DEL00076709	chr34	1,338,933	1,339,266	333	Loss
DUP00076716	chr34	1,355,981	1,356,068	87	Gain
DEL00076717	chr34	1,367,808	1,367,998	190	Loss
DEL00076728	chr34	1,441,300	1,441,533	233	Loss
DUP00076731	chr34	1,445,805	1,446,255	450	Gain
DUP00076733	chr34	1,446,650	1,447,012	362	Gain
DEL00076739	chr34	1,471,136	1,471,544	408	Loss
DUP00076752	chr34	1,481,659	1,482,088	429	Gain
DEL00076763	chr34	1,493,265	1,493,392	127	Loss
DUP00076779	chr34	1,629,775	1,630,204	429	Mixed
DEL00076793	chr34	1,631,056	1,631,143	87	Loss
DUP00076820	chr34	1,777,846	1,794,562	16,716	Gain
DEL00076835	chr34	1,995,945	1,996,076	131	Loss
DEL00076842	chr34	2,068,933	2,069,006	73	Loss
DEL00076848	chr34	2,076,047	2,076,356	309	Loss
DUP00076852	chr34	2,091,134	2,091,754	620	Gain
DEL00076871	chr34	2,095,047	2,095,498	451	Loss
DEL00076881	chr34	2,107,498	2,107,575	77	Loss
DEL00076893	chr34	2,112,595	2,114,053	1,458	Loss
DUP00076907	chr34	2,126,387	2,126,831	444	Gain
DUP00076912	chr34	2,132,568	2,133,016	448	Gain
DEL00076922	chr34	2,135,601	2,135,771	170	Loss
DUP00076956	chr34	2,144,838	2,145,399	561	Gain
DEL00076971	chr34	2,150,907	2,151,437	530	Loss

DEL00076978	chr34	2,170,942	2,171,076	134	Loss
DUP00077327	chr35	227,002	228,798	1,796	Gain
DEL00077337	chr35	229,488	244,839	15,351	Loss
DEL00077952	chr36	286,904	287,410	506	Loss
DUP00077956	chr36	290,818	291,284	466	Gain
DEL00077959	chr36	291,968	293,025	1,057	Mixed
DEL00078107	chr36	323,495	324,954	1,459	Loss
DEL00078135	chr36	347,000	350,094	3,094	Loss
DEL00078145	chr36	366,859	367,580	721	Loss
DUP00078209	chr36	431,550	431,776	226	Gain
DEL00078331	chr38	8,995	9,472	477	Loss
DUP00078425	chr38	180,696	181,061	365	Gain
DUP00078435	chr38	201,468	201,884	416	Gain
